# BOOK OF POSTERS

**DOI:** 10.1080/24740527.2024.2366132

**Published:** 2024-10-30

**A large-language-model (LLM)-ready, extended SECI model framework for guiding creation of narrative stories concerning living with chronic neuroplastic pain (NPC) and useful in guiding co-creation of clinical care change-way scenarios**

Peter Pennefather^a, b^, West Suhanic^b^, Claudia Lai^c^ and S. Fatma Lakha^d^

^a^University of Toronto; ^b^gDial; ^c^University of Victoria; ^d^Pain and Wellness Center

**Introduction/Aim**: Chronic-neuroplastic-pain (CNP) is a persistent condition associated with cognitive misinterpretation of safe sensory signals as potentially hazardous. Large-language-models (LLMs) are being applied to support a broad spectrum of individuals in generating decision-support responses. A comprehensive framework is essential for adapting LLMs to ensure accurate interpretations and responses appropriate to the complexities of a given context. We are applying a hybrid [Bio-Psycho-Social-Ecological]-[Socialization-Externalization-Combination- Internalization-(SECI)] conceptual-framework for structuring personal-real-world data relevant in training patient-centered LLMs for supporting personalized clinical-care for persons-living-with-NPC.

**Methodology**: We are adapting gDial’s patented, OpenSSL v.3.0 compliant, technology for securely registering, atomizing, materializing, and encapsulating unstructured data, in a license-protected encrypted manner. We have named our hybrid framework the MCOA (Materiality-Cognitive-Organizational-Affordance) conceptual framework.

**Results**: The framework organizes unstructured personal-digital-data associated with person-centered real-world data, linked to particular persons-living-with-CPN, within a patient-owned data repository owned by the person-data-is-about. This then can be augmented with other patient-linked data generated within their “digital-first” world. Rapidly evolving digital data privacy legislation is providing increasing accessibility by all to machine-readable data-objects representing structured “data-about-them.”

**Discussion/Conclusions**: The framework will structure clinical-records and patient-reported-outcomes for training patient-centered LLMs. Making stories generated by the LLMs for their Circles-of-Care will help them deal with the clinical perplexity associated with changing patient circumstances. The approach should help using LLMs in articulating comprehensive stories, constrained by NPC-linked real-world data, that are useful in guiding the cocreation of clinical care change-way scenarios and presenting patient-reported outcomes in generally understandable formats.

**Chronic noncancer pain best practices: Developing clinical tools to support primary care clinicians with chronic noncancer pain management**

Arun Radhakrishnan, Cameron Ross, Kelly Lang-Robertson, Vipusaayini Sivanesanathan, Anne Dabrowski, Katie Hagel, Pippy Scott-Meuser, Dersha Uthayakumaran, Valentina Gnanapragasam

Center for Effective Practice

**Introduction/Aim**: Chronic noncancer pain (CNCP) affects 20% of Canadians and is recognized as a complex disease, with a wide scope in management and treatment. Due to limited access to specialist care, many patients rely on primary care providers (PCP) for CNCP management. To support PCPs and patients, we developed national Best Practices for the development of effective CNCP clinical decision support systems (CDSSs).

**Methodology**: The Best Practices were developed through literature searches and eScans to outline the clinical and technical standards for appropriate, effective, efficient, and compassionate CNCP management which were assessed by a national advisory committee. These Best Practices were leveraged to design a point-of-care electronic medical record (EMR) CDSS for CNCP using an iterative design process. The CDSS development process aligned with technical best practices including incorporating key design features and clinical information elements to support CNCP management in primary care, and early and sustained engagement of clinicians and patients through clinical codesign, usability testing and clinical pilot testing to refine and evaluate the CDSS for implementation.

**Results**: Likert scale results from survey responses and interview-based feedback on clinician experience and satisfaction with the CDSS during on-site pilot testing were analyzed; majority of clinicians reported satisfaction with the CDSS, with emphasis on the design features of multimodal management options and patient-centered education components.

**Discussion/Conclusions**: The national Best Practices identified practical, and actionable principles to apply when developing CDSS tools for CNCP management in primary care. These best practices can serve as a reference as the work expands to additional disease states and CDSS platforms.

**Sex differences in the prevalence of chronic pain in children and adolescents: Sub-analysis of an updated systematic review and meta-analysis**

Annemarie Dedek^a^, Christine T Chambers^a, b, c^, Justine Dol^b^, Jennifer A Parker^b^, Perri R Tutelman^a, b^, Charlotte L Langley^b^, Brittany T Cormier^b^, Gary J Macfarlane^d^, Gareth T Jones^d^, Darlene Chapman^e^, Nicole Proudfoot^b^, Amy Grant^f^, and Justina Marianayagam^g^

^a^Department of Psychology and Neuroscience, Dalhousie University; ^b^IWK Health Center, Center for Pediatric Pain Research; ^c^Department of Pediatrics, Dalhousie University; ^d^Epidemiology Group and Aberdeen Center for Arthritis and Musculoskeletal Health, University of Aberdeen; ^e^Health Sciences Library, IWK Health Center; ^f^Maritime SPOR Support Unit, ^g^Patient Partner and Medical Student, Northern Ontario School of Medicine

**Introduction/Aim**: Children and adolescents are disproportionately impacted by chronic pain, which is persistent or recurring pain lasting longer than three months. A 2011 systematic review by King and colleagues uncovered sex differences in the prevalence of pain between sexes, however, updated analysis is required. Therefore, as part of an updated systematic review on the prevalence of chronic pain in children and adolescents, the objective was to compare the prevalence of chronic pain in females and males across multiple pain types.

**Methodology**: EMBASE, PubMed, CINAHL, and PsychINFO were searched for papers published between 01/01/2009, and 30/06/2023. Papers reporting population-based estimates of chronic non-disease-related pain prevalence in populations aged 19 years or less were included. The number of participants, sex, and type of pain (headache, abdominal, back, musculoskeletal, multisite/combined, other) were extracted.

**Results**: 119 studies with 1,043,878 children in total were included. Overall, a statistically significant higher prevalence of pain was reported in females (18.3%) than males (12.7%, *p* < .001). By pain type, females reported significantly more pain with headache (23.5% vs 15.1%), abdominal pain (11.3% vs 7.1%), multisite/combined pain (24.5% vs 15%), and other pain (9.7% vs 5.1%) than males. No significant difference was found in musculoskeletal and back pain. Data showed high heterogeneity (I.^1^ 999%).

**Discussion/Conclusions**: Females ≤19-years experience a greater prevalence of chronic pain overall as well as higher chronic headache, abdominal, multisite/combined, and other pain than males. Future studies should expand on the binary categorization of sex and measure the prevalence of chronic pain across gender in children and adolescents.

**Expectant parents’ beliefs and perspectives of involvement in newborns’ pain management**

Ligyana Korki de Candido^a^, Paula Forgeron^b^, Janet E. Squires^b, c^, Wendy Peterson^b^ and Denise Harrison^a^

^a^Department of Nursing, University of Melbourne; ^b^School of Nursing, University of Ottawa; ^c^Ottawa Hospital Research Institute

**Introduction/Aim**: Parents are underutilized in newborns’ pain management (NPM), (e.g., breastfeeding, skin-to-skin care (SSC), sucrose administration). Little is known about expectant parents’ beliefs about pain and involvement in their NPM during routine painful procedures.

**Methodology**: Cross-sectional survey targeting Canadian expectant parents recruited through social media. The Theoretical Domains Framework guided data analysis to assess factors associated with intentions to be involved in NPM. Online prenatal pain education (video) was provided. Data was analyzed using descriptive statistics.

**Results**: 134 mothers and 4 fathers completed the survey. Prevideo, 93% believed newborns feel pain during procedures, identified breastfeeding (84%) and SSC (93%) as effective analgesic strategies. Less than half (43%) agreed that sucrose was effective. Nearly all (98%) believed participating in NPM is part of their parenting role, and 89% intended to discuss their involvement with healthcare providers. Most intended to use breastfeeding (80%) and SSC (90%) to relieve pain, but only 38% intended to use sucrose. Parents felt confident (97%) and optimistic (94%) about participating in NPM, believing in short- (94%) and long-term (90%) benefits for their newborn’s health. Parents did not foresee feeling stressed (72%) or anxious (65%) during their participation and felt supported by their cultural beliefs (80%), peers (97%), family (96%), and partners (98%).

**Discussion/Conclusions**: Parents hold positive beliefs about their capabilities and benefits of their involvement in NPM, reporting a preference for using breastfeeding and SSC. Investigating factors hindering or facilitating parental involvement in NPM is essential to support their parenting role and improve pain care.

**Les disparités dans l’expérience de la douleur chronique au sein de la diversité sexuelle et de genre**

Philippe Trudel, Marianne McNally, Dominique Trottier, and Stéphanie Cormier

Université du Québec en Outaouais

**Introduction/Objectif**: La douleur chronique est une condition de santé qui ne se répartit pas de manière égale dans la population canadienne. De récentes études ont montré qu’elle toucherait davantage certains groupes de la société, dont la communauté LGBT+. Peu de recherches se sont toutefois intéressées aux disparités dans l’expérience de la douleur auprès de ce groupe d’individus. Cette étude comparative transversale propose donc d’examiner la douleur, le fonctionnement et la détresse psychologique au sein de la communauté LGBT+.

**Méthodologie**: Un échantillon préliminaire de 90 adultes a été réparti en trois groupes, soit un groupe *cishétéro* (*n* = 40), un groupe *cishomo* (*n* = 25), et un groupe *autre* composé de gens non monosexuels, transgenres ou non binaires (*n* = 25). Les sujets ont rempli des questionnaires en ligne mesurant l’intensité de la douleur, le fonctionnement et la détresse psychologique.

**Résultats**: Les analyses révèlent que le groupe *autre* rapporte des douleurs d’intensité plus élevée que les autres groupes, ainsi qu’une interférence fonctionnelle plus élevée que le groupe *cishomo*. Une tendance à dramatizer la douleur plus élevée a aussi été rapportée chez le groupe *autre* comparativement aux autres groupes, mais aucune différence n’a été décelée entre les trois groupes dans les symptômes dépressifs.

**Discussion/Conclusions**: Cette étude illustre le fardeau supplémentaire que portent les gens transgenres, bisexuels ou pansexuels vivant avec des douleurs chroniques. Elle réitère aussi l’importance de cibler les défis uniques auxquels sont confrontés les groupes marginalisés de la société aux prises avec des douleurs persistantes dans le but de promouvoir des pratiques plus inclusives et équitables.

**Hybrid cognitive behavior therapy for insomnia and chronic pain in adolescent: Assessment and intervention strategy illustrated by a case study**

Adam Newton, Sarah Naraine, Melissa Baidoobonso, Joshua Lee, Raju Poolacherla, and Abirami Kandasamy

Children’s Hospital at London Health Sciences Center

**Introduction/Aim**: Over 50% of adolescents with chronic pain experience significant insomnia. Pain and sleep have a bidirectional relationship; however, recent research suggests that nighttime sleep has a more substantial impact on daytime pain, than the inverse. Yet, there are few established protocols for the assessment and treatment of insomnia symptoms for these adolescents.

**Methodology**: We conducted a narrative review of the literature on assessment and intervention processes for co-occurring chronic pain and insomnia symptoms. We implemented the findings of this review to the assessment and intervention practices in a Pediatric Chronic Pain Program and highlight how quantitative and qualitative sleep data informs clinical decision making for sleep-pain interventions.

**Results**: An assessment and intervention strategy was developed for the clinic. This process included the integration of standardized sleep questionnaires and a structured sleep interview. Recommended sleep-related interventions included a group CBT for chronic pain and sleep intervention and an individual hybrid CBT for insomnia (CBT-I) treatment. In the case study with a 17-year-old female with chronic visceral pain and insomnia, 6-session hybrid CBT-I was associated with an increase in sleep diary-reported sleep efficiency, sleep duration, and sleep quality, as well as clinically significant decreases in self- and parent-reported symptoms of insomnia and daytime somnolence. The intervention was well-received by the youth and parent.

**Discussion/Conclusions**: Sleep assessments can inform clinical decision making in adolescents with chronic pain. Brief sleep interventions hold promise for the treatment of co-occurring sleep and chronic pain in adolescents. Further studies are required to valid this approach in controlled samples.

**Test-retest reliability of homeostatic plasticity induced and assessed in the primary motor cortex using TBS**

Emma Tassinari^a, b^, Phivos Phylactau^b, c^ and Siobhan Schabrun^b, c^

^a^Graduate Program in Neuroscience, Faculty of Health Sciences, University of Western Ontario, London, ON, Canada; ^b^The Gray Center for Mobility and Activity, Parkwood Institute, London, ON, Canada; ^c^School of Physical Therapy, University of Western Ontario, London, ON, Canada

**Introduction/Aim**: Homeostatic plasticity regulates synaptic plasticity to maintain an optimal level of neuronal activity by preventing excessive neuronal excitability or silencing. Aberrant homeostatic plasticity is thought to play a role in chronic pain, hence a reliable approach to induce homeostatic plasticity is needed. Recently, it has been demonstrated that transcranial magnetic theta burst stimulation (TBS), can be used to induce homeostatic plasticity in the human primary motor cortex (M1), by implementing a priming-test TBS paradigm. However, the reliability of TBS-induced homeostatic plasticity has yet to be investigated. Our aim was to test the reliability of inducing homeostatic plasticity in the M1across days using TBS.

**Methodology**: We investigated the test-retest reliability of TBS induced homeostatic plasticity in the M1 in 11 healthy individuals, across days 0, 2, and 7. Using a crossover design, homeostatic plasticity was investigated in response to both an excitatory and an inhibitory priming-test paradigm. To assess homeostatic plasticity, 20 MEPs were recorded from the first dorsal interossei (FDI) muscle at baseline, between the priming and test blocks, and at 0-, 10-, and 20-minutes poststimulation.

**Results**: Homeostatic plasticity was successfully induced for both the excitatory and inhibitory paradigm (*F*= 3.72, *p*= .045). Overall, cortical excitability was highly consistent across days (ICC = .89). Reliability for the inhibitory priming paradigm was slightly higher (ICC = .86) compared to the excitatory paradigm (ICC = .82).

**Discussion/Conclusions**: Our findings show that the priming-test TBS paradigm could serve as a dependable noninvasive tool to investigate homeostatic plasticity, paving a novel way for studying the neural underpinnings of chronic pain.

**Using graph theory to evaluate functional network properties of the brain in persons with surgically confirmed endometrioses**

Clara Moon, Claire Lunde, David Borsook, Christine Sieberg, and Scott Holmes

Boston Children’s Hospital

**Introduction/Aim**: Endometriosis is a highly prevalent gynecological condition wherein endometrial tissue exists outside of the uterus resulting in significant pelvic pain that is often chronic in nature. Research to date has shown using both structural and functional brain imaging that brain health is compromised in persons with surgically confirmed endometriosis (SCE), which may have a basis in chronic pain exposure. There has been little research in terms of functional network organization, which may provide insight into adaptive reorganization. Our hypothesis is that exposure to chronic pain in persons with SCE will result in less efficient, more condensed functional networks.

**Methodology**: We performed a cross-sectional (45 SCE; 39 Controls) and in a cohort with visits before and after surgical confirmation (7 SCE; 8 Controls – No Surgery). Each individual completed a 2-hour study visit including behavioral testing and a 1-hour MRI visit. Graph theory was applied using the CONN toolbox.

**Results**: Cross-sectional data showed higher eccentricity in controls compared to SCE participants in regions including the right postcentral gyrus, left superior parietal lobule, and the precuneus cortex. Current analyses are focused on understanding differences between pre- and postsurgical MRI scans. Results for persons in the SCE cohort demonstrated a decrease in eccentricity in left thalamus, right caudate, and right cerebellum 3 after surgical confirmation.

**Discussion/Conclusions**: Findings of decreased eccentricity points toward a reduction in the extent of brain regions involved in functional networks. These findings may point toward changes in white matter pathways and underscore the impact of prolonged pain exposure in persons with SCE.

**Decoding pain management discourse: A social media analytics approach to the Canadian Armed Forces community online**

Brent Davis^a, b^, Dominic Gargala^b^, Anthony Nazarov^b^ and Don Richardson^a, b, c^

^a^Western University; ^b^MacDonald Franklin OSI Research Center; ^c^OSI Clinic London

**Introduction/Aim**: The prevalence of chronic pain among Canadian Armed Forces (CAF) personnel and Veterans has prompted increasing interest in alternative and emerging pain management strategies. This study aims to analyze discussions related to pain and pain treatment methods within the /r/Canadian Forces subreddit on Reddit, focusing on key terms such as “Ketamine,” “Mushrooms” (for Psilocybin), and “Pain,” to gauge interest and sentiment toward these topics over time.

**Methodology**: Utilizing the complete historical data of the /r/Canadian Forces subreddit up to the end of 2023, we conducted a frequency analysis of the specified key terms to identify trends. Additionally, a Latent Dirichlet Allocation (LDA) topic model was applied to a subset of posts directly mentioning “pain,” aiming to uncover underlying topics and associated phenomena, with a particular focus on “tinnitus” as indicated by the model.

**Results**: The frequency analysis revealed a growing interest in alternative treatments, notably Ketamine and Psilocybin, alongside a consistent concern for pain management. The topic modeling highlighted several pain-related discussions, with “tinnitus” emerging as one concern. A subsequent trend analysis of “tinnitus” mentions corroborated its increasing relevance within the community discussions.

**Discussion/Conclusions**: This study offers insights into evolving perspectives of CAF personnel and Veterans on pain and its treatment, emphasizing an openness to emerging therapies. The identification of tinnitus as a notable topic is curious in the context of “long covid” related causation. This analysis method proves effective in elucidating complex dynamics of military-related health discussions on social media, paving the way for future investigations into specific interests and concerns regarding chronic pain management.

**Lateralization of pain induced facial grimacing**

Alicia Zumbusch, Elodie Nickner, Susana Sotocinal, Gabriel Firanescu, Paula Sanchez, Lilian Yoffe, Janik Falcarek-Hope, and Jeffrey Mogil

McGill University

**Introduction/Aim**: Facial expressions are adaptive for communicating an organism’s state to others in its surroundings. Research shows that facial expressions of emotion (e.g., fear) are stronger on the left side of the face. Whether pain expression is similarly lateralized has never been assessed. This study investigated whether facial expression of pain, as measured by the Mouse Grimace Scale (MGS), is lateralized and whether the pain model or pain location alters the lateralization of grimacing.

**Methodology**: Mice received intraperitoneal injections of acetic acid (AA), injection of complete Freund’s adjuvant (CFA), zymosan or carrageenan in the left or right hind paw or received a spared nerve injury on the left or right leg. Subsequently, we video-recorded grimacing for 30 min, and videos were sampled to obtain left- or right-side-only image types. These were: 1) left and right profile images, 2) symmetrical front-facing images of only the left and only the right half of the face, and 3) facial chimeras produced by mirroring hemiface images. Images were then scored using the MGS. We hypothesized that the left side of the face would show higher MGS scores than the right.

**Results**: Grimacing was significantly stronger on the *right* side of the face than on the left for all image types and all pain assays tested. We observed this lateralization regardless of the location of the pain itself.

**Discussion/Conclusions**: We are currently investigating the underlying neurobiology of grimace asymmetry using chemogenetic inhibition of areas associated with pain expression and assessing if the same effect is observed in humans.

**Social stories to prepare autistic children for needle procedures: An online environmental scan**

Olivia Dobson^a^, Carter Janssen^a^, Anna Taddio^b^, Frank Symons^c^and C. Meghan McMurtry^a^

^a^University of Guelph; ^b^University of Toronto; ^c^University of Minnesota Twin Cities

**Introduction/Aim**: Needle procedures are a painful experience and information provision is an important pain management strategy. Standard information provision may not fit the needs of children with autism spectrum disorder and may need to be customized to their needs. Preparation is critical for autistic children given this group commonly struggles with needles and unpredictability. Social Stories teach autistic children about situations and there are many Social Stories about needle procedures online; however, research has not examined whether their content is consistent with clinical practice guidelines for managing pediatric needle-related pain (e.g., distraction).

**Methodology**: An online environmental scan of Social Stories about needle procedures was conducted via Google using search terms including Vaccine and Social Story. Following screening for inclusion, two researchers used deductive content analysis with 80+ codes derived from clinical practice guidelines for needle-related pain and fear and research specific to autistic children. Analysis is underway and will conclude by March 2024.

**Results**: Frequency statistics and quotes will be presented to describe the content of 82 eligible Social Stories. Preliminary findings indicate: a focus on the COVID-19 vaccine; a lack of customizability options; certain helpful strategies are depicted often (e.g., distraction) while others more rarely (e.g., topical anesthetics); and some unhelpful strategies are commonly depicted (e.g., uninformative reassurance).

**Discussion/Conclusions**: To minimize harm and maximize benefit for families, we must understand whether online resources convey helpful, evidence-based information. Social Stories appear to convey some helpful and unhelpful information. The results will inform future research on using Social Stories to help autistic children cope with needles.

**Sex-based differences in pain reporting in clinician notes: A natural language processing approach**

Brent Davis^a, b^, Dominic Gargala^b^, Yuan Du^b^, Anthony Nazarov^b^ and Don Richardson^a, b, c^

^a^Western University; ^b^MacDonald Franklin OSI Research Center; ^c^OSI Clinic London

**Introduction/Aim**: Chronic pain management and documentation practices within healthcare settings are pivotal for patient care. This study leverages natural language processing (NLP) techniques to examine clinician notes from an operational stress injury clinic chronic, aiming to identify patterns and linguistic habits in chronic pain descriptions and explore the impact of patient sex on documentation practices.

**Methodology**: Employing a bag of words approach, our analysis encompassed a dataset of 3669 clinician notes which were analyzed for mentions of pain. A key methodological step involved extracting patient sex from medical records, facilitating a sex-stratified analysis to investigate disparities in pain documentation.

**Results**: The analysis unveiled notable differences in pain documentation, with references to pain being twice as prevalent in notes pertaining to female patients as opposed to male patients. This finding suggests potential gender biases in clinical assessments or reporting, emphasizing the need to consider sex as a critical variable in medical documentation and research. Furthermore, our study began to uncover additional linguistic patterns, indicating the potential for broader applications of NLP in understanding chronic pain documentation.

**Discussion/Conclusions**: This research highlights significant disparities in how pain is documented in clinical notes based on patient sex, pointing to underlying gender biases in pain perception and reporting. The application of NLP techniques in this context not only reveals clinician documentation habits but also opens avenues for further studies aimed at developing more equitable and informed pain management strategies. Through continued exploration and expansion of our dataset, we anticipate uncovering more insights that could enhance chronic pain documentation and treatment practices.

**Stress, depressive mood and fatigue and their associations with pain intensity: an ecological momentary assessment study**

Karen Ghoussoub^a, b^, Mael Gagnon-Mailhot^a, b^, Élise Develay^b^, Sonia Lupien^a^, Pierre Rainville^a^, Mathieu Roy^c^, Étienne Vachon-Presseau^c^ and M. Gabrielle Pagé ^a, b^

^a^Université de Montréal; ^b^Research Center of the Center hospitalier de l’Université de Montréal; ^c^McGill University

**Introduction/Aim**: The biopsychosocial framework is used to capture the multidimensional pain experience. Multiple psychosocial factors are associated with pain intensity, including depressive mood, fatigue, and stress. However, it is unclear how intra-individual stress and psychological state fluctuations are associated with concurrent pain intensity. This study aimed to examine the associations between the momentary fluctuations of stress, depressive mood, fatigue and their interactions and momentary pain intensity.

**Methodology**: In this ecological momentary assessment study, participants (*n* = 181) living with chronic low back pain completed electronic diaries three times a day during seven days. Diaries included assessments of pain intensity, stress intensity, depressive mood and fatigue levels using the Numeric Rating Scale-11. Baseline sociodemographic and averaged pain intensity were also collected prior to the diary completion.

**Results**: Participants had an average age of 49.5 years (*SD* = 12.5) and the majority were women (58.3%), with an average pain intensity of 6.0 (*SD* = 1.7). Results of multilevel modeling showed that initial averaged pain intensity (β = 0.51) and fluctuations in levels of stress (β = 0.09), depressive mood (β = 0.18) and fatigue (β = 0.23) were significantly associated with concurrent pain intensity (*p* < .001). No significant interactions between fluctuations in stress, mood, or fatigue were found to predict pain intensity.

**Discussion/Conclusions**: Results show that higher levels of stress, mood and fatigue predict higher concurrent pain intensity, but these factors do not have an interactive effect. The evidence presented in this study highlights the importance of examining intra-individual variability in psychosocial factors to better understand dynamic fluctuations in the pain experience.

**La stigmatization de la douleur chronique chez les adultes de la communauté: Étude de son occurrence et de ses facteurs associés**

Marianne Mc Nally, Vanessa Prud’Homme, and Stéphanie Cormier

Université du Québec en Outaouais

**Introduction/Objectif**: La douleur chronique affecte environ le quart de la population canadienne, mais le manque de compréhension à son sujet expose les personnes concernées à la stigmatization. Un nombre croissant d’études illustre d’ailleurs la prévalence élevée et les conséquences néfastes de la stigmatization de la douleur chronique, mais peu d’écrits scientifiques se sont intéressés à son occurrence et aux facteurs associés chez les personnes de la population générale. Cette étude propose donc d’explorer la stigmatization de la douleur chronique telle que perçue par des adultes de la communauté.

**Méthodologie**: Les données de 291 personnes ont été colligées à l’aide de questionnaires électroniques autorapportés. En plus du recueil de données sociodémographiques, l’adaptation française du *Stigma Scale for Chronic Illnesses*, le Questionnaire concis sur les douleurs et l’Indice de détresse psychologique ont été administrés.

**Résultats**: Des niveaux modérés de stigmatization ont été rapportés par notre échantillon composé d’adultes vivant avec des douleurs chroniques. Des corrélations significatives (p ≤ .05) ont été décelées entre la stigmatization perçue et diverses variables cliniques et sociodémographiques, dont l’intensité de la douleur, la localization de la douleur, le soulagement en lien aux traitements, le revenu familial, et le statut professionnel. La stigmatization perçue s’est également avérée être un prédicteur significatif (p ≤ .001) de l’interférence de la douleur et de la détresse psychologique.

**Discussion/Conclusions**: Cette étude fournit des preuves convaincantes de l’occurrence et des corrélats de la stigmatization de la douleur chronique perçue par les adultes de la communauté, et souligne l’importance de renforcer les initiatives éducatives visant à démystifier la condition.

**Dissemination and implementation of Project ECHO for Chronic Pain and Opioid Stewardship across Canada**

Yalnee Shantharam^a^, Quinn Cassucio-Treen^a^, Rebecca Binik^b^, Mark Ware^b^, Patricia Poulin^c^, Andrew Koscielniak^c^, Alex Falcigno^c^, Cathy Scrimshaw^d^, Lori Montgomery^d^, Jennifer Stinson^e^, Chitra Lalloo^e^, Lauren Harris^e^, Susan Tupper^f^, Cathy Jeffery^f^, Selene Daniel-Whyte^f^, Rebecca Titman^g^, John Flannery^a^ and Andrea Furlan^a^

^a^Toronto Rehabilitation Institute- University Health Network; ^b^McGill University; ^c^St. Joseph’s Care Group; ^d^Alberta College of Family Physicians; ^e^Hospital for Sick Children; ^f^University of Saskatchewan; ^g^Mount Sinai Hospital

**Introduction/Aim**: Project ECHO (Extensions of Community Health Outcomes) has been successfully used as an educational platform in chronic pain and opioid stewardship in Ontario since 2014. Using the Project ECHO Ontario Chronic Pain and Opioid Stewardship model, this research project expanded the model to develop pilot sites for ECHO in other provinces and special populations across Canada.

**Methodology**: New ECHO programs were piloted across the following sites: McGill University (Quebec), Alberta College of Family Physicians (Alberta), St. Joseph’s Care Group (Northern Ontario), Mount Sinai Hospital (Ontario), Hospital for Sick Children (Ontario), College of Physicians and Surgeons Saskatchewan (Saskatchewan). Each pilot site developed a hub team of experts, a curriculum of 8–12 sessions and collected pre and post test data.

**Results**: Seven new pilot ECHO programs in chronic pain and opioid stewardship were developed and piloted during January 2021 to March 2024. Four of these pilot programs included special populations including Indigenous communities, pregnant women, and pediatric populations. Data collection is ongoing and final results will be analyzed by March 2024. Preliminary data collected on pre- and postknowledge tests and satisfaction surveys for two sites have shown improvements in scores. Full data analysis will be available March 2024.

**Discussion/Conclusions**: Seven pilot ECHO Chronic Pain and Opioid Stewardship programs ran successfully with lessons learned and areas of improvement recorded. Pre and post data analysis once completed is expected to show an improvement in knowledge tests and high satisfaction scores for attendance of ECHO pilot programs. Seeking ongoing funding will be a priority to continue the work done thus far.

**Considerations for ketamine in the effective management of chronic pain and mental health for Veterans: A systematic review and meta-analysis**

Jenny Liu, Natalie Ein, Julia Gervasio, Anthony Nazarov, andJ. Don Richardson

MacDonald Franklin OSI Research Center

**Introduction/Aim**: Ketamine has emerged as a promising treatment alternative for the management of chronic pain. Despite encouraging findings in civilian populations and favorable results from trials examining its efficacy in military populations, there is still a dearth of information for chronic pain and mental health conditions in military populations. To address this gap, we sought to conduct a systematic review and meta-analysis synthesizing evidence on the effectiveness, tolerability, and feasibility of ketamine in the management of chronic pain and mental health conditions in Veterans.

**Methodology**: The preregistered review was conducted following PRISMA and Cochrane guidelines. A total of 1020 studies were reviewed, and 11 studies were retained for data analysis. From each study, the following information was extracted: study characteristics, study data, and potential moderator/study rigor information.

**Results**: Ketamine led to significant reductions in mental health symptoms and pain, with moderate-to-large effects. These improvements were observed in both active-duty and Veteran groups, and for different routes of ketamine administration, frequencies of ketamine administration, duration of ketamine treatments, dosage, study design, and allowance for concurrent treatments.

**Discussion/Conclusions**: The evidence suggests that ketamine may be a promising treatment for mental health and chronic pain in military populations.

**Investigating the comorbidity of PTSD and chronic pain: Prescription patterns and treatment considerations in OSI clinics across Canada**

Jenny Liu, Anthony Nazarov, and J. Don Richardson

MacDonald Franklin OSI Research Center

**Introduction/Aim**: We examine the characteristics and prescribed medications of patients with PTSD and chronic pain comorbidity.

**Methodology**: We overview findings from a two-part survey on the prescription practices of clinicians in treating posttraumatic stress disorder (PTSD) in Operational Stress Injury (OSI) clinics across Canada.

**Results**: Findings indicate distinct differences in characteristics of patients with PTSD, including PTSD specifier and mental health comorbidity. We also noted commonly prescribed medications in the management of chronic pain comorbid PTSD.

**Discussion/Conclusions**: Taken together, these findings illuminate the current practices in OSI clinics while emphasizing the importance of considering chronic pain as a significant factor in the treatment of military-related PTSD. These insights will aid in guiding future policies and research aimed at refining and personalizing treatment strategies for PTSD in military personnel and Veterans, especially those living with chronic pain.

**Sustaining CARD (Comfort, Ask, Relax, Distract) practices to continue positive patient experiences in university-based pop-up vaccination clinics**

Victoria Gudzak^a^, Anna Taddio^a, b^, Charlotte Logeman^b^, Natalie Crown^a^, Lucie Bucci^c^, Mike Folinas^a^, Michael Chan^a^, Rachel Kuruvilla^a^, Lisa Dolovich^a^ and C. Meghan McMurtry^d, e^

^a^University of Toronto; ^b^SickKids Hospital; ^c^Bucci-Hepworth Health Services Inc; ^d^University of Guelph; ^e^McMaster Children’s Hospital

**Introduction/Aim**: CARD^TM^ (Comfort, Ask, Relax, Distract) is a vaccination delivery framework that reduces adverse reactions (fear, pain). It was introduced into university-based vaccination pop-up clinics led by the Leslie Dan Faculty of Pharmacy (LDFP) in fall, 2022. 64% of clients reported a better vaccination experience compared to their last vaccination after CARD compared to 34% before CARD. Integration was supported on-site by CARD developers and LDFP preceptors. This study examined the benefit one year later, after sustained CARD implementation overseen by LDFP preceptors.

**Methodology**: LDFP hosted four influenza and COVID-19 vaccination clinics in November 2023. Sustainment strategies included: 1) CARD education for doctor of pharmacy student vaccinators; 2) ‘CARD lead’ staff role to oversee CARD implementation, 3) standard operating procedures, and 4) client and staff feedback to continuously improve vaccination delivery. A rate of ≥50% of clients reporting a more positive vaccine experience compared to their last vaccination was considered significant.

**Results**: Doctor of pharmacy students were involved in all aspects of the clinics (set up, vaccine preparation and administration). CARD environmental interventions included directional and CARD-related signage, separate waiting and vaccination areas and private rooms for vaccination. 59% of 547 clients reported a more positive experience compared to their last vaccination.

**Discussion/Conclusions**: This study demonstrated a sustained benefit of implementing CARD in vaccination clinics. Continued tailoring of practices according to client and staff feedback is recommended to further improve vaccination delivery in pop-up clinic settings.

**Perceptions of pharmacy students after introducing the CARD system (Comfort-Ask-Relax-Distract) to the injection curriculum**

Anna Taddio^a, b^, Natalie Crown^a^, Victoria Gudzak^a^, Charlotte Logeman^b^, Lucie Bucci^c^ and C. Meghan McMurtry^d^

^a^University of Toronto; ^b^SickKids Hospital; ^s^Bucci-Hepworth Health Services Inc; ^d^University of Guelph

**Introduction/Aim**: Due to an expanded scope of practice, the proportion of vaccinations delivered by pharmacists is steadily increasing. Adequate preparation of pharmacy students is required to ensure patients have positive vaccination experiences. The CARD system (Comfort-Ask-Relax-Distract) identifies interventions which reduce pain, fear and fainting. CARD was introduced in the requisite injections course for second year Doctor of Pharmacy students (*n* = 240) at the University of Toronto’s Leslie Dan Faculty of Pharmacy. We evaluated perceptions about CARD education in two consecutive student cohorts.

**Methodology**: Two rounds of 3 focus groups were held after course completion, including 18 students in the 2022 cohort and 14 in the 2023 cohort. In 2022, CARD training included an e-module and video assignment. 2023 curriculum changes followed student feedback, including modifying the e-module and integrating CARD ‘practice’ in the certification workshop, whereby students inject a peer. Feedback was analyzed deductively using the Consolidated Framework for Implementation Research.

**Results**: Both cohorts endorsed that fear is part of vaccination in patients across the lifespan. They learned ways to improve patient vaccination experiences which would strengthen their patient-provider relationship and increase role satisfaction. However, some described community pharmacies as ‘rushed’ and largely unfamiliar with CARD, introducing implementation barriers. 2022 cohort students reported insufficient education about how CARD “works” and described offering patients selected CARD interventions. 2023 cohort students highlighted patient autonomy in choosing interventions. Both cohorts recommended more practice opportunities.

**Discussion/Conclusions**: This study identified pharmacy student perceptions of the CARD curriculum that informed revisions. Future research will examine perspectives in other vaccinator groups.

**Playing CARD (Comfort, Ask, Relax, Distract) during hospital-based needle procedures in children: Effect on clinical and experience outcomes**

Victoria Gudzak^a^, Mandy Kohli^b^, Charlotte Logeman^b^, Christine Shea^a^, Lucie Bucci^c^, C. Meghan McMurtry^d, e^, Vibhuti Shah^f^ and Anna Taddio^a, b^

^a^University of Toronto; ^b^SickKids Hospital; ^c^Bucci-Hepworth Health Services Inc; ^d^University of Guelph; ^e^McMaster Children’s Hospital; ^f^Mount Sinai Hospital

**Introduction/Aim**: Fear and pain are primary concerns for children undergoing needle procedures in hospital. The CARD™ system (Comfort, Ask, Relax, Distract) is an evidence-based framework that systematically integrates interventions to reduce fear and pain. CARD was implemented in the Nuclear Medicine Department of The Hospital for Sick Children, Toronto, Canada. We evaluated impact on clinical and experience outcomes.

**Methodology**: This before-and-after quality improvement study included patients aged ≥5 years that were undergoing needle procedures and able to self-report symptoms (fear, pain, dizziness) and their caregivers. CARD interventions were introduced using Plan, Do, Study, Act cycles. Following the first procedure attempt, patients and caregivers reported symptoms and experiences compared to the patient’s last needle procedure.

**Results**: 118 patients and caregivers participated (September 2022-December 2023). Patient characteristics did not differ between study phases. CARD interventions included changes to the environment (e.g., distraction items), patient and caregiver engagement (e.g., communication strategies), onsite patient and caregiver education (e.g., CARD coping checklist), and evaluation (e.g., feedback surveys). Patients’ self-reported mean dizziness scores (0–10) were lower after CARD compared to before (0 vs. 0.7, *p* = .01) and fewer patients reported high levels of fear (≥7 out of 10); 13% vs 28% (*p* = .05). Caregivers reported lower fear (0–10) in themselves (2.8 vs. 1.5, *p* = .01). A higher percentage of caregivers reported more positive needle experiences compared to the previous needle procedure (65% vs 46%, *p* = .05).

**Discussion/Conclusions**: CARD demonstrated benefits in clinical and experience outcomes. CARD continues to be used in the department and will be expanded to other areas of the hospital.

**Co-occurring opioid use disorders and chronic pain: Expert perspectives to support research and clinical management**

Léonie Archambault^a^, Mélanie Bérubé^b^ and Michel Perreault^c^

^a^Institut universitaire sur les dépendances du CIUSSS du-Center-Sud-de-l’île-de-Montréal; ^b^Université Laval; ^c^Douglas Institute and McGill University

**Introduction/Aim**: Opioid use disorder (OUD) and chronic pain (CP) often co-occur, which can complicate treatment, worsen symptoms and hinder quality of life for patients. High quality scientific evidence for managing these co-occurring conditions is scarce. This project aimed to develop expert consensus around clinical management and research needs for co-occurring OUD and CP.

**Methodology**: A Delphi survey comprising 42 statements was based on a scoping review of the literature to identify areas of consensus between addiction experts, pain experts and people with lived experience of OUD and CP.

**Results**: Clinical expert recruitment proved to be difficult, as many of the targeted experts did not consider themselves sufficiently knowledgeable to participate. We aimed to recruit 20 experts, but only five agreed to take the survey. Three had stronger affiliations with pain medicine, and two were mostly involved in the addiction field. Twenty-nine statements reached consensus between clinical experts. Most of the statements that did not reach consensus highlight distinct perspectives between disciplines. In terms of participants with lived experience of OUD and CP, 10 were recruited, as expected. Only four statements reached consensus, underlining the heterogeneity of experiences. Only one round of the survey was administered because of the small sample size and the nature of the conflicts.

**Discussion/Conclusions**: Results highlight the importance of supporting interdisciplinary collaboration and training between the fields of pain and addiction. Future steps should mobilize an expert panel and support a deliberative process to develop recommendations for the management of co-occurring OUD and CP.

**Risk of infections among persons treated with opioids for chronic pain: A systematic review and meta-analysis**

Irina Kudrina^a, b, c^, M. Gabrielle Pagé^d^, Manon Choinière^d^, Yoram Shir^a^, Mark J. Eisenberg^b, e^, Maayan BenSasoon^a^, Bertrand Lebouché^f, g^ and Svetlana Puzhko^h^

^a^Anesthesia Department, Faculty of Medicine and Health Sciences, Alan Edwards Pain Management Unit; ^b^McGill University; ^c^Research Institute of the McGill University Health Center, ^d^Department of Anesthesiology and Pain Medicine, Faculty of Medicine, Université de Montréal; ^e^Center for Clinical Epidemiology, Lady Davis Institute for Medical Research, Jewish General Hospital; ^f^Faculty of Medicine and Health Sciences, Family Medicine Department, McGill University; ^g^Centre for Outcomes Research and Evaluation, Research Institute of the McGill University Health Center; ^h^Department of General Practice and Family Medicine, University of Bielefeld, Germany

**Introduction/Aim**: Millions of persons with chronic cancer and noncancer pain (CP) worldwide use opioids. Immunosuppressive properties of opioids are associated with avoidable risks of infection-related complications. There is no guidance on how to limit infection risks and optimize opioid therapy for patients with CP. We conduct a systematic review (SR) to identify the evidence on the association of opioid use with increased risks for infections, re-infections, and negative infectious outcomes among persons with CP using opioids.

**Methodology**: SR methodology is based on PRISMA statement, the MOOSE Guidelines for Meta-Analyses and SRs of Observational Studies, and the Cochrane Handbook for SR of Interventions. We systematically search seven databases, gray literature (inception date to December 2023) in English, French, Spanish, German. We include primary studies evaluating measurable infectious outcomes in adults with CP using opioids of all types in all settings (except self-injection of illicit opioids).

**Results**. Pilot search (PubMed, Google Scholar, backward citation) and preliminary screening yielded 25 primary studies (18 retrospective cohorts, 7 case control). Race/ethnicity, daily opioid doses >50 MEDD, long-acting formulations, immunosuppressive opioids, use of >90 days were independently associated with risks of serious infections (pneumonia, meningitis/encephalitis, septic arthritis/osteomyelitis, endocarditis, pyelonephritis, cellulitis, septic shock), negative outcomes for COVID-19 infection, related hospitalizations, ICU admissions, or mortality.

**Discussion/Conclusions**: Studies reported clinically significant risks of negative infectious outcomes among persons with CP using opioids, especially immunosuppressive, in higher doses, long-term. Meta-analysis will be conducted. Deliberative dialogue group discussions with stakeholders and decision makers will help to contextualize study results and inform policy developments. PROSPERO (CRD42023402812)

**Exploring the impacts of sex and gender on the chronic pain management experiences of Canadian Veterans**

Joy MacDermid^a^, David Walton^a^, Shannon Killip^b^, Margaret Lomotan^b^and Pavlos Bobos^a^

^a^University of Western Ontario; ^b^McMaster University

**Introduction/Aim**: Although sex and gender can impact the injuries sustained and responses to treatments for military personnel, little research has been done to understand how sex and gender impact pain management among Veterans with chronic pain. The purpose of our study was to explore the unique experiences of Canadian men and women Veterans in managing their chronic pain.

**Methodology**: Twenty-six Canadian Veterans (11 women, 15 men) with chronic pain participated in semi-structured interviews. Interpretive description was used to analyze the transcripts. One coder performed the thematic analysis, while confirming codes and themes with the research team.

**Results**: We identified 4 themes: 1. Differences in expectations for men and women in the military influenced their likelihood of chronic pain, 2. The ‘macho mentality’ in the military impacts men and women differently, 3. Healthcare providers have a lack of understanding of chronic pain, especially in women, which impacted the treatment they received, 4. Veterans experience other forms of discrimination which impact their chronic pain.

**Discussion/Conclusion**: Women Veterans explained that training exercises developed for men, and feeling like they needed to prove themselves increased their risk of injuries and chronic pain. Men Veterans believed that they were expected to push through pain and were given more physically demanding tasks, leading to chronic pain. Both men and women Veterans felt that their chronic pain symptoms were often ignored and were frustrated by the lack of treatments they received. Veterans explained discrimination due to the stigmas associated with chronic pain also impacted their access to treatments.

**Exploring the influence of brief cognitive behavioral therapy on central sensitization: A replication study**

Aleiia Asmundson^a^, Shokouh Abolhosseini^a^, Richard Harrison^b^, Greig Adams^b^, Carien Van Reekum^b^ and Tim Salomons^a^

^a^Queen’s University; ^b^University of Reading

**Introduction/Aim**: Painful experiences involve a complex interplay of biopsychosocial factors. One such factor, central sensitization (CS), amplifies nociceptive signaling in the central nervous system, resulting in heightened perceptions of pain in response to noxious stimuli. A previous report (Salomons, et al., 2014) found that cognitive behavioral therapy (CBT), a psychological intervention known for altering thinking patterns, has demonstrated efficacy in reducing CS during acute pain studies, even in brief sessions. Here we attempt to replicate that study in a larger sample.

**Methodology**: This study examines the impact of brief CBT on secondary hyperalgesia (SH), a proxy for CS. Participants were randomized into two groups: brief CBT for pain (n = 48), and an active intervention (i.e., interpersonal effectiveness training) control group (n = 48). Both groups underwent baseline assessments and completed 8 sessions of thermal pain induction and brief intervention. SH was measured after the first and last sessions.

**Results**: Analyses of variance were conducted for SH ratings between groups and across time. There was a significant time effect (F = 31.67, *p* = < .001) for SH reduction for both groups; however, there was no significant difference between the two groups (F = 2.42, *p*= .123).

**Discussion/Conclusions**: While there was a notable reduction in SH over time, the difference between interventions did not reach statistical significance. These findings suggest that psychological interventions can reduce CS, but the effects may not be specific to CBT.

**Pain Sensitivity Questionnaire as a clinical proxy for identifying central sensitization symptoms in endometriosis?**

Avonae Gentles, Sarah Wong, Natasha Orr, Heather Noga, Catherine Allaire, Christina Williams, Mohamed Bedaiwy, Caroline Lee, and Paul Yong

University of British Columbia

**Introduction/Aim**: Some people with endometriosis experience recurring pain after endometriosis surgery, with up to 50% requiring reoperation within 5 years. Central sensitization (CS), characterized by heightened pain sensitivity and allodynia, may provide an explanation for posttreatment pain. The Pain Sensitivity Questionnaire Minor (PSQ-M) is a 7-item questionnaire that assesses generalized pain sensitivity and is associated with experimental pain ratings and postoperative pain scores in chronic pain groups. However, there is limited evidence linking the PSQ-M to CS or endometriosis. This study investigated the associations between baseline PSQ-M scores and postsurgical quality of life (QoL) and pain scores in endometriosis patients.

**Methodology**: We analyzed data from patients in the Endometriosis Pelvic Pain Interdisciplinary Cohort Data Registry (EPPIC) with a clinic visit (baseline) between January 2020–2022 and underwent endometriosis surgery. We conducted multivariable regression analyses between baseline PSQ-M scores and QoL and postsurgical patient-reported pain scores while controlling for baseline QoL and pain scores (No pain/Mild 0–3; Moderate 4–6; Severe 7–10). ORs and CIs were reported.

**Results**: We included 345 participants with endometriosis with a mean age was 34 years. Baseline PSQ-M scores were not significantly associated with postoperative QoL (β = −0.34, [−2.76–2.08]), or pain scores for dysmenorrhea (OR = 1.02, [0.65–1.04]), deep dyspareunia (OR = 0.94, [0.75–1.16]), superficial dyspareunia (OR = 0.99 (0.97–1.01)), dysphasia (OR = 0.95 95% CI: 0.78–1.16) and chronic pelvic pain (OR = 1.12 [0.84–1.49]).

**Discussion/Conclusions**: Baseline PSQ-M was not significantly associated with postsurgical QoL or pain scores, suggesting that the PSQ may not be an adequate tool for predicting outcomes after endometriosis surgery.

**Using automated patch clamp for high throughput characterization of sodium and potassium channels in human induced pluripotent stem cell-derived sensory neurons**

Mei Zhang^a^, Vincent Truong^b^, Daniel Sauter^a^, Patrick Walsh^b^ and Kyle Jensen^a^

^a^Sophion; ^b^Anatomic Incorporated

**Introduction/Aim**: Dorsal root ganglion (DRG) neurons transfer sensory signals from the peripheral to the central nervous system. Understanding the electrophysiological properties of DRG neurons has a significant application for pain research and potential drug development. Given the limited availability of human DRG neurons, the development of human-induced pluripotent stem cell (hiPSC)-derived sensory neurons (SNs) that contain DRG neuron electrophysiological properties provides a promising way to perform in vitro pain research. In addition, the latest expansion of automated patch clamp (APC) into a 384-well format provides the possibility of a high throughput screening (HTS) for thousands of compounds.

**Methodology**: In this study, RealDRG™ hiPSC-derived SNs were produced at massive scale and consistency to meet the demands of high-throughput APC studies. These SNs bear similarities to human DRG from a whole-transcriptome perspective and have been previously shown to possess functional voltage and ligand-gated channels important for nociception via manual patch clamp. To show the utility of nociceptors in HTS APC, we investigated the electrophysiological properties of hiPSC derived SNs on the Qube 384 APC system over time spans of 9-, 14-, 21-, and 28-day cultures.

**Results**: Using an optimized cell dissociation protocol to obtain healthy cell membranes for patch-clamp, we obtained a whole-cell success rate of 40.69–53.19%.

**Discussion/Conclusions**: Among these cells, 90.54–97.22% expressed KV currents, and 59.76–77.48% expressed Nav currents. For the Nav current group, 30.7–37.5% of cells carried a detectable TTX-resistant component. Furthermore, under current clamp mode, action potential firings were recorded from 60.12–66.76% of the cells that passed the success filter criteria.

**The impact of socioeconomic status and the COVID-19 pandemic on access to chronic pain management in Nova Scotia**

Nicole Atkins^a^, Alexandra MacNeil^b^ and Karim Mukhida^c^

^a^Department of Anesthesia, McMaster University; ^b^Dalhousie University, ^c^Department of Anesthesia, Pain Management and Perioperative Medicine, Dalhousie University

**Introduction/Aim**: Socioeconomic status (specifically employment status and income) often impacts chronic pain management, and this has likely been further exacerbated by the COVID-19 pandemic. Our objective of the study was to better understand the impact of an individual’s socioeconomic status on access to pain management services in Nova Scotia, and whether the COVID-19 pandemic impacted access to treatments.

**Methodology**: A retrospective review was completed using electronic medical records at the Pain Management Unit in the Queen Elizabeth II Health Sciences Center in Nova Scotia. The patients included in this study had their initial visit in May-June 2019 (prior to the pandemic) or in May-June 2020 (following the pandemic onset). Data was collected from each patient’s initial appointment and any follow-up appointments within one year.

**Results**: There were 95 patients who had their first appointment at the pain clinic in May-June of 2019, and there were 27 patients who had their first appointment between May-June of 2020. Patients who were unemployed were more likely to be offered opioid prescriptions to manage their chronic pain. Following the onset of the COVID-19 pandemic, patients were also more likely to be prescribed opioids at the first appointment.

**Discussion/Conclusions**: These results suggest that individuals who are unemployed may not receive equal therapeutic opportunities for chronic pain management. However, these results could be due to different factors, such the affordability of alternative therapies, health literacy, and prescribing biases. Future research studies are needed to identify interventions to improve equitable access to chronic pain therapies.

**Behind the screens: Perspectives of healthcare professionals to inform an equitable, patient-oriented decision aid for in-person versus virtual pediatric chronic pain care**

Mica Marbil^a, b^, Justin Bonhomme^c^, Alexandra Neville^d^, Nicole MacKenzie^e, f^, Vina Mohabir^g, h^, Katherine Wynne-Edwards^i^, Isabel Jordan^j^, Melanie Noel^a^, Fiona Webster^k^, Krista Baerg^l^, Fiona Campbell^m^, Tim Oberlander^n^, Nivez Rasic^c, o^, Jennifer Stinson^f, m, p^, Karine Toupin-April^q, r, s^, Tracy Wasylak^t, u^, Kathryn Birnie^a, c^

^a^Department of Psychology, University of Calgary, Alberta, Canada; ^b^Alberta Children’s Hospital Research Institute, University of Calgary, Alberta, Canada; ^c^Department of Anesthesiology, Perioperative and Pain Medicine, University of Calgary, Alberta, Canada; ^d^Department of Anesthesiology, Perioperative, and Pain Medicine, Stanford University School of Medicine, California, USA; ^e^Department of Psychology and Neuroscience, Dalhousie University, Nova Scotia, Canada; ^f^Centre for Pediatric Pain Research, IWK Health, Nova Scotia, Canada; ^g^Child Health Evaluative Sciences, Research Institute, The Hospital for Sick Children, Ontario, Canada; ^h^Patient and Family Partner, Ontario, Canada; ^i^Patient and Family Partner, Alberta, Canada; ^j^Patient and Family Partner, British Columbia, Canada; ^k^Arthur Labatt Family School of Nursing, Western University, Ontario, Canada; ^l^Department of Pediatrics, University of Saskatchewan, Saskatchewan, Canada; ^m^Department of Anesthesia and Pain Medicine, The Hospital for Sick Children, University of Toronto, Ontario, Canada; ^n^Department of Pediatrics, University of British Columbia, British Columbia, Canada; ^o^Vi Riddell Children’s Pain and Rehabilitation Center, Alberta Children’s Hospital, Alberta, Canada; ^p^Lawrence S. Bloomberg, Faculty of Nursing, University of Toronto, Ontario, Canada; ^q^School of Rehabilitation Sciences and Department of Pediatrics, University of Ottawa, Ontario, Canada; ^r^Children’s Hospital of Eastern Ontario Research Institute, Ontario, Canada; ^s^Institut du savoir Montfort, Ontario, Canada; ^t^Strategic Clinical Networks, Alberta Health Services, Alberta, Canada; ^u^Faculty of Nursing, University of Calgary, Alberta, Canada

**Introduction/Aim**: Shared decision-making (SDM), a collaborative decision-making process between patients, caregivers, and healthcare professionals, could improve pediatric chronic pain (CP) treatment outcomes. As part of a multiphase project, we gathered experiences of tertiary care healthcare professionals (HCPs) to inform the development of an equitable, patient-oriented decision aid for in-person versus virtual pediatric CP management.

**Methodology**: Using a mixed-methods approach, up to 15 HCPs across Canadian multidisciplinary tertiary pediatric CP programs will complete an online demographic survey and semi-structured interview. Derived from the Ottawa Decision Support Framework, interview questions capture HCP virtual care experiences and desired decision aid features. Data will be analyzed using reflexive thematic analysis.

**Results**: To date, 10 (2 physicians, 2 nurses, 3 psychologists, 2 physiotherapists, 1 recreation therapist) have participated. Preliminary analyses revealed that HCPs emphasized patient-centered care, whether in-person or virtual, that reduces CP burden among families. Perspectives in decision-making, optimal content (e.g., risks and benefits of treatment, patient trajectories) and format (e.g., website or paper) of a decision aid varied according to HCP role and clinic resources and practices. Nevertheless, HCPs placed importance on the decision aid’s accessibility and applicability. Three more HCPs (1 physician, 1 nurse, 1 psychologist) have consented to interviews and will be included in final analyses.

**Discussion/Conclusions**: HCPs underscore the importance of patient-centered pediatric CP intervention regardless of delivery. Implications for the development of the decision aid include ensuring availability and generalizability to clinics. Next steps include interviewing diverse youth with CP and caregivers to further inform the decision aid.

**Mind the curve: Exploring the curvilinear relationship between hyperarousal and chronic pain in Canadian Veterans**

Martine Southall, Ian Kyte, Chloe McLeod, Taylor Linsenmeier, Spenser Martin, and Pamela Holens

University of Manitoba

**Introduction/Aim**: Despite the strong link between posttraumatic stress disorder (PTSD) and chronic pain in military and veteran populations, there is limited research examining how PTSD impacts pain in these populations using the Diagnostic and Statistical Manual of Mental Disorders-Fifth Edition’s criteria. Additionally, few studies examine PTSD from a multidimensional, symptom cluster level.

**Methodology**: We conducted a retrospective chart analysis of 153 Canadian Armed Forces veterans. A hierarchical multiple regression analysis of PTSD symptom clusters on pain was used to examine which symptom clusters of PTSD predicted pain. For symptom cluster(s) that were found to predict pain, we conducted additional regressions to test for potential curvilinear relationships to better understand the nature of the relationship between the cluster(s) and pain. Finally, we performed post-hoc correlations of pain intensity and each symptom within these symptom cluster(s).

**Results**: Our initial hierarchical regression found that only hyperarousal predicted pain. Subsequent regressions revealed significant cubic and quadratic (i.e., curvilinear) relationships indicating that hyperarousal decreased after a certain point of pain intensity. The post-hoc correlations revealed that specific symptoms (sleep difficulties, hypervigilance, and heightened startle response) were correlated with pain intensity while others were uncorrelated.

**Discussion/Conclusions**: The three correlated symptoms within the hyperarousal symptom cluster relate to the construct of anxiety sensitivity, which has been proposed as an underlying mechanism explaining the link between PTSD and chronic pain. Our findings thus offer further support for this theory and suggest that treatments targeting pain and PTSD may see greater improvement if hyperarousal symptoms are prioritized.

**Classical conditioning confounds preclinical pain testing**

Oakley B Morgan, Lianfang Liang, Aleksandrina Skvortsova, Susana Sotocinal, and Jeffrey Mogil

McGill

**Introduction/Aim**: Research shows that the testing environment can produce hypersensitive states, with pairing of painful stimulus to context leading to an exacerbated pain response via classical conditioning. Given that most behavioral preclinical pain studies involve multiple testing sessions in the same room, we wondered whether conditioning hypersensitivity might be conflated with hypersensitivity from an injury, thus confounding the interpretation of results.

**Methodology**: Mechanical withdrawal thresholds of mice were assessed using von Frey fibers pre- and postchronic constriction injury (CCI) in the same context (Room A), alternating contexts (A/B) or with a switch in contexts at day 10 (A-to-B). Separate groups of mice were baselined and tested at single time points along the course of the testing paradigm, in room A. A review was conducted using a manual inspection of preclinical papers published in *PAIN* between 2016–2020, featuring multiple postinjury testing days.

**Results**: CCI mice tested in room A presented with hypersensitivity lasting up to 35 days. Alternating context A/B and an A-to-B switch produced a shortened allodynic state and a return to baseline at 21 days. Single time point testing reduced the CCI-induced hypersensitivity, with a return to baseline from day 7 onward. Analysis of pain literature revealed a significant positive correlation between number of postinjury trials and time taken to return to baseline sensitivity.

**Discussion/Conclusions**: Our results suggest conditioning confounds in preclinical pain testing exist. We therefore propose that a portion of pain hypersensitivity is not attributable to the injury, but to a classically conditioned response to the testing context associated with repeated pain-inducing stimulation.

**Sex and strain differences in injury-induced mechanical and thermal hypersensitivity for two common models of chronic neuropathic pain**

Damien Boorman and Loren Martin

University of Toronto Mississauga

**Introduction/Aim**: Preclinical animal models are essential for identifying the complex neurobiological adaptations that underlie chronic pain states. However, the behavioral consequences of the two most common models, chronic constriction injury (CCI) and spared nerve injury (SNI), which were originally developed in rats, have yet to be fully characterized in mice. Therefore, the aim of this study was to compare sex and strain differences in the time courses of thermal and mechanical hypersensitivity of each injury model.

**Methodology**: 24 male and 24 female CD1 and C57BL/6 mice either received CCI (*n* = 16), SNI (*n* = 16), sham (*n* = 8) or no surgery (*n* = 8). Hypersensitivity to heat, cold and mechanical stimuli was assessed regularly over the following 30 days using the hot plate test, cold plate test and Von Frey test respectively.

**Results**: SNI induced stronger and more rapid mechanical allodynia, but weaker cold allodynia, compared to CCI. Subtle sex differences and moderate strain differences were observed within each model. Female CCI mice developed quicker mechanical allodynia and stronger cold allodynia than males, while CD1 mice developed stronger cold allodynia than C57BL/6 mice. Neither model induced strong heat hyperalgesia in any of the mice.

**Discussion/Conclusions**: Consistent with previous studies, CCI and SNI produced strong mechanical allodynia, albeit with different time courses. However, both models only produced weak heat and cold hypersensitivity when compared to rats. As such, the use of these models in mice may have limited utility when investigating the neurobiology of pain conditions that are characterized by strong changes to thermal sensitivity.

**Analgesic benefits of sleep interventions for adults with fibromyalgia: Preliminary findings from a systematic review and meta-analysis**

Shokouh Abolhosseini^a^, Aleiia J. N. Asmundson^a^, Ava Malatesta^a^, Ian Gilron^b^ and Tim V. Salomons^a^

^a^Department of Psychology, Queens University, Kingston, ON, Canada; ^b^Department of Anesthesiology and Perioperative Medicine, Queen’s University, Kingston, ON, Canada

**Introduction/Aim**: Fibromyalgia is a chronic musculoskeletal pain condition affecting 2–8% of the population. Most people with fibromyalgia experience poor sleep, and sleep problems consistently predict pain severity. Research also suggests that sleep problems may precede the onset of fibromyalgia symptoms in healthy adults. Given the bidirectional relationship between sleep and pain, improving sleep may reduce fibromyalgia pain. This review identified trials of sleep-focused pharmacological and nonpharmacological interventions to evaluate whether they improved pain outcomes for adults with fibromyalgia.

**Methodology**: A systematic search of 7 electronic databases retrieved 4764 records. To be included, trials had to investigate an intervention used for sleep problems in adults with fibromyalgia, collecting both sleep-related and pain-related outcomes. Interventions that targeted both sleep and pain simultaneously were excluded. Three independent reviewers completed study screening and data extraction. Eighteen unique studies met the inclusion criteria.

**Results**: Preliminary meta-analyses were conducted for two interventions (4.5 g sodium oxybate [SXB] and cognitive behavioral therapy for insomnia [CBT-I]) compared to inactive control groups. Three studies were included in the SXB analysis, which showed a significant improvement in both sleep (MD = 2.50, 95%CI [0.71, 4.29]) and pain outcomes (MD = −8.98, 95%CI [−12.00, −5.96]) compared to placebo. Four studies were included in the CBT-I analysis, which showed no significant difference between groups for either outcome.

**Discussion/Conclusions**: This review provides preliminary support for the analgesic benefits of sleep interventions for adults with fibromyalgia, even if such interventions have no primary effect on pain. Findings may inform fibromyalgia treatment plans and self-management efforts by clarifying the relationship between two debilitating fibromyalgia symptoms.

**Project Extension for Community Healthcare Outcomes (ECHO) Indigenous Chronic Pain and Substance Use (IPCandSU): A braided approach to understanding in a good way**

Andrew Koscielniak^a^, Alex Falcigno^a^, Paul Francis^a^, Teresa Trudeau-Magiskan^a^, Chris Mushquash^b^, Donna Garstin^a^, Marinna Read^a^, Andrew Smith^c^, Virginia McEwen^d^, Yaadwinder Shergill^e^, Yalnee Shantharam^f^, Andrea Furlan^f^, Natalie Zur Nedden^e^, Alycia Benson^a^, Patricia Poulin^e^

^a^St. Josephs Care Group; ^b^Lakehead University; ^c^CAMH; ^d^NOSM; ^e^OHRI; ^f^UHN

**Introduction/Aim**: Indigenous Peoples stories are understood in relation to their worldviews. Through a braided approach bringing together multiple ways of knowing we discuss and give space to Indigenous and western conceptualizations of wellness as it relates to chronic pain (CP). The ICPandSU ECHO is a continuing professional development program aiming at improving health care providers’ (HCPs) ability to provide culturally safer care for Indigenous Peoples. This project focuses on sharing the development, implementation, and evaluation of ICPandSU ECHO in a good way.

**Methodology**: We use a Braided Approach weaving together Indigenous storytelling, quantitative and qualitative evaluation, as well as knowledge sharing through a 10-hour process of Ceremonies, Elder’s Teachings, Talking Circles, and presentations aimed at embodying a culturally safer approach to evaluation (Sharing Day).

**Results**: The development of ICPandSU culminates in the implementation of two 10-session series covering trauma, history of opioids in Indigenous communities, assessment through an Indigenous lens, psychological pain, and traditional healing. The program reached 57 HCPs, 77.8% to 94.1% indicated moderately or greatly improved knowledge and appreciation for the Medicine Wheel as a framework to address CP. The Sharing Day fostered reflections, an appreciation of different worldviews, and challenged program evaluators to re-think what.

**Discussion/Conclusions**: Project ECHO must understand the history of Indigenous Peoples and of the land on which it convenes its sessions. It must also recognize the value of Traditional Healing Systems to achieve its goal of improving wellbeing/healing outcomes for all. We discuss two-eyed seeing approaches for future Project ECHO development.

**Item reduction of the Pediatric PainSCAN© – a novel self-report screening tool for pediatric neuropathic pain and complex regional pain syndrome**

Giulia Mesaroli^a, b^, Fiona Campbell^c, d^, Kristen Davidge^e^, Aileen Davis^f^, Rachel Kelly^g^, Naiyi Sun^c^, Jennifer Tyrrell^c, h^, Anthony V. Perruccio^f, I^ and Jennifer Stinson^f, h^

^a^ Department of Rehabilitation Services,The Hospital for Sick Children; ^b^ Department of Physical Therapy and Institute of Health Policy, Management, and Evaluation, University of Toronto; ^c^ Department of Anesthesia and Pain Medicine, The Hospital for Sick Children; ^d^ Temerty Faculty of Medicine, University of Toronto; ^e^ Division of Plastic Surgery, The Hospital for Sick Children; ^f^ Institute of Health Policy, Management, and Evaluation, University of Toronto; ^g^ Child Health Evaluative Sciences, The Hospital for Sick Children; ^h^ Lawrence S. Bloomberg Faculty of Nursing, University of Toronto; ^i^ Schroeder Arthritis Institute, Krembil Research Institute, University Health Network

**Introduction/Aim**: The Pediatric PainSCAN© is the first self-report screening tool for pediatric neuropathic pain (NP) and complex regional pain syndrome (CRPS). The tool has been developed through a phased approach. Phases 1–3 developed an initial item pool (37 items) and determined item comprehensibility. To reduce patient burden and enable timely administration and interpretation, the aim of this study (phase 4) was item reduction.

**Methodology**: A cross-sectional survey was administered to patients with NP or CRPS and to health care professionals (HCPs) with pediatric pain expertise. Participants rated the frequency and importance of each item in the tool on a 5-point Likert scale (0–4). Data were analyzed using descriptive statistics. First, mean importance and frequency scores were calculated per item. Second, items were ranked according to mean importance and frequency scores. Finally, the highest-ranking items were retained in the tool (maximum 21 items).

**Results**: Patient participants (*n* = 43) ranged from 11–18 years old (median age 15) and were predominantly female (77%). HCP participants (*n* = 74) were predominantly female (72%) and practiced in North America (70%). From the initial item pool, 19 items were retained (mean importance scores ranged from 2.24–3.48; mean frequency scores ranged from 1.90–3.38), 3 items were labeled “grey zone,” and 15 items were deleted.

**Discussion/Conclusions**: The Pediatric PainSCAN© aims to identify patients with potential NP and CRPS to facilitate referrals for specialist assessment and treatment. The retained items were highly ranked by participants, suggesting the tool may be effective in the clinical setting. Further consultation is needed with key partners to determine the final item pool.

**Women’s pain on TikTok: Analysis of video content**

Kelly Mazzocca^a^, Tori Langmuir^a^, Michelle Gagnon^b^ and Nicole Alberts^a^

^a^Concordia University; ^b^University of Saskatchewan

**Introduction/Aim**: Social media platforms can provide insight into the public’s experiences and attitudes toward health topics, yet little is known about the narrative surrounding women’s pain on these platforms, including experiences with chronic pain conditions (e.g., dysmenorrhea) and procedure related pain (e.g., intrauterine device insertion).

**Methodology**: “Women’s pain” was searched using the most relevant video function on TikTok, and the first 50 videos were reviewed by two coders, with discrepancies resolved by a third reviewer. Video tone and pain type were coded, and video content was analyzed thematically. Metadata for all videos was extracted.

**Results**: From our search, 40 videos were relevant and had a total of 3.3 million likes, 21562 comments, and 30034 shares. More than half the videos had a negative tone (*n* = 22, 55%), followed by neutral (*n* = 7, 17.5%), ambiguous (*n* = 6, 15%), and positive tones (n = 5, 12.5%). Regarding pain type, most videos discussed endometriosis (35%) or pain during penetrative sex (35%), followed by vaginismus (15%), dysmenorrhea (10%) and stomach pain (10%). Of the videos with a negative tone, common themes included experiencing distress due to pain, feeling dismissed by a healthcare professional, hope for pain alleviation, and uncertainty around medical advice.

**Discussion/Conclusions**: Women are frequently using TikTok to share their personal experiences with pain, with a large proportion of videos describing negative experiences with pain management and healthcare. Understanding the social media content created by women regarding their pain can provide important insights for those involved in both pain care and research.

**Exploring the relationship between empathy and the perceptual evaluation of pain facial expressions: Insights from a high-definition transcranial direct current stimulation research**

Marie-Claude Desjardins^a^, Daniel Fiset^a^, Daphnée Sénécal^a^, Arthur R. Chaves^b, c^, Sara Tremblay^a, c, d, e^ and Caroline Blais^a^

^a^Department of Psychoeducation and Psychology, University of Quebec in Outaouais; ^b^Faculty of Health Sciences, University of Ottawa; ^c^University of Ottawa Institute of Mental Health Research at The Royal; ^d^Department of Neuroscience, Carleton University; ^e^Department of Cellular and Molecular Medicine, University of Ottawa

**Introduction/Aim**: Efficient pain communication is crucial to humans’ survival, yet individuals often underestimate others’ pain and struggle to detect its subtle variations (called sensitivity). As empathy and pain rely on shared neural processes, this social competence is proposed to mediate pain evaluation adequacy. The neurostimulation of the right inferior frontal gyrus (rIFG), a key region of both networks, could temporarily alter empathic abilities. However, the impact of such manipulation on the parameters that underlie the evaluation of pain facial expressions (the bias and sensitivity) remains unclear.

**Methodology**: High-definition transcranial direct current stimulation (HD-tDCS: Anodal-Cathodal-Sham) was applied over the rIFG of 25 participants for 20 min before completing three tasks: visual expectations, pain evaluation, emotional and cognitive empathy.

**Results**: Our sample exhibited a generalized tendency to underestimate and a suboptimal sensitivity to others’ pain, while these parameters did not correlate [*M_bias_ *= −1.47, *M_sensitivity_ *= −.40, *r*= −.22, *p*= .30]. Extracted visual expectations of pain facial expressions further confirmed that participants’ percept was composed of facial features typically associated with painful experiences [*T_crit_ *= 2.3, *k_crit_ *= 452, *p*<.05]. Using the clustering approach, we additionally revealed three distinctive response patterns to HD-tDCS for emotional empathy and estimation bias. Most interestingly, stimulating the rIFG selectively impacted the bias [*F*(2,22) = 21.32, *p*<.001, *n^1^* = .68] but not the sensitivity to pain variations [*F*(2,22) = 0.01, *p*= .38] in one cluster, and this positively correlated with changes in participants’ empathic resources [*F*(2,22) = 8.83, *p*= .002, *n^1^*=.72].

**Discussion/Conclusions**: Our findings emphasize the importance of considering interindividual variability in response to stimulation and suggest the bias is a malleable factor that can be influenced by external manipulations.

**Distraction from pain depends on cognitive demand and motivation**

Sophie Desjardins^a^, Todd A. Vogel^b^ and Mathieu Roy^a^

^a^McGill University; ^b^University of Birmingham

**Introduction/Aim**: Although pain automatically captures our attention, engaging in a demanding cognitive task can reduce concurrent pain. At the same time, expending cognitive effort is subjectively felt as aversive and costly. Therefore, engaging in cognitive effort may help reduce pain but only up to a certain point. Our study aimed to identify the limits of cognitive demand in distraction and to use monetary rewards to alleviate the aversiveness of cognitive effort.

**Methodology**: 10 pain-free adults were recruited to participate in our study. Participants performed a demanding cognitive task at three levels of difficulty (low, medium, and high) while receiving painful thermal stimulations for a chance of obtaining a monetary reward ($0 or $20) if they performed well on the task.

**Results**: We found a significant main effect of difficulty on pain ratings (*F*(2, 354) = 14.02, *p*< .001). Post-hoc tests revealed that pain ratings were significantly lower for medium compared to low difficulty (*p* < .001), but there was no significant difference in pain ratings between medium and high difficulty (*p* = .78). We also found a significant main effect of reward on pain ratings (*F*(1, 354) = 5.12, *p* = .02), revealing that pain ratings were significantly lower on rewarded ($20) compared to nonrewarded trials ($0).

**Discussion/Conclusions**: Our findings suggest that performing a demanding cognitive task can help alleviate pain even when difficulty is very high. This suggests that the effectiveness of distraction may be driven by the amount of effort invested into the competing task.

**The diminished capacity to recognize pain expressions among Black individuals appears to be associated more with a stringent decision-making criterion rather than a lack of sensitivity to visual cues**

Daphnée Sénécal^a^, Camille Saumure^b^, Marie-Pier Plouffe-Demers^c^, Daniel Fiset^a^, Frédéric Gosselin^d^ and Caroline Blais^a^

^a^University of Quebec in Outaouais; ^b^University of Fribourg; ^c^University of Quebec in Montreal; ^d^University of Montreal

**Introduction/Aim**: Studies reveal that the pain experienced by individuals of Black ethnicity is underestimated in countries where most individuals are of White ethnicity. Moreover, when it comes to detecting pain based on facial expressions, White observers have more difficulty with Black faces compares to White faces. This detection of pain in facial expressions involves at least two processes: the sensitivity to the visual information embedded in pain facial expressions and the criteria used to ascertain that a face genuinely conveys pain. The present study aims at verifying if the difficulty at detecting pain in faces of Black ethnicity is associated with an altered sensitivity, an altered criterion, or both.

**Methodology**: We conducted a series of four experiments where participants saw either Black or White faces depicting pain facial expressions or neutrality. On each trial, participants had to determine whether the face displayed pain or not (Exp. 1,3,4) or they were asked to decide which of two faces was expressing pain (Exp. 2a and 2b). For experiments 1, 2a and 2b, White participants (*n* = 150) from predominantly White countries were recruited on Prolific. In experiments 3 and 4, White participants (*n* = 100) coming from predominantly white countries, and Black participants (*n* = 100) coming from predominantly Black countries were recruited.

**Results**: Overall, the impairment in detecting pain in Black faces is mostly associated with an altered criterion.

**Discussion/Conclusions**: Consequently, further studies should explore the theoretical implications of these results, specifically considering the potential contribution of our mental representations and expectations concerning the pain experienced by Black ethnicity.

**Les caractéristiques de la douleur chronique en tant que déterminants de la stigmatization: Une étude par vignettes**

Stéphanie Cormier, Marianne Mc Nally and Alexandra Lévesque-Lacasse

Université du Québec en Outaouais

**Introduction/Objectif**: Les personnes atteintes de douleur chronique sont souvent confrontées à des réactions stigmatisantes, entraînant des répercussions sur leur bien-être physique, mental et social. Pourtant, les déterminants de la stigmatization de la douleur chronique demeurent largement méconnus. Cette étude propose donc d’explorer si certaines caractéristiques de la douleur chronique sont plus susceptibles de générer des réactions stigmatisantes.

**Méthodologie**: 160 participants issus de la population générale ont été assignés à l’une des quatre vignettes décrivant un personnage fictif souffrant de douleur. Trois vignettes illustraient des caractéristiques de la douleur chronique (étiologie inconnue, invisibilité, usage d’opioïdes), tandis que la quatrième décrivait une douleur aiguë. Les réponses immédiates à l’égard du personnage ont été évaluées à l’aide de questionnaires auto-rapportés, dont le *Social Distance Scale* (SDS). La familiarité avec la douleur chronique en général a été mesurée à l’aide du *Level of Contact Report* (LCR) et du *Chronic Pain Myth Scale* (CPMS).

**Résultats**: Les réponses stigmatisantes diffèrent significativement selon les caractéristiques de la douleur. Notamment, les participants ont rapporté vouloir une plus grande distance sociale (SDS) avec le personnage souffrant de douleur chronique d’étiologie inconnue, comparativement à celui présentant une douleur aiguë. La distance sociale (SDS) était aussi significativement prédite par les connaissances, croyances et attitudes à l’égard de la douleur chronique (CPMS), mais pas par l’âge, le genre ou le niveau de contact avec la douleur chronique (LCR).

**Discussion/Conclusions**: Cette étude révèle des schémas distincts de stigmatization selon les caractéristiques de la douleur chronique et réitère la nécessité de démystifier cette affection au sein de la population générale.

**Identification of analgesic bioactive lipids following green light therapy in osteoarthritic rats**

Melissa O’Brien^a^, Heather Bradshaw^b^ and Jason McDougall^a^

^a^Dalhousie University; ^b^Indiana University

**Introduction/Aim**: We have recently discovered that treating osteoarthritic (OA) rats with ambient green light significantly attenuated pain behavior but had no effect on joint nociceptor activity. The aim of the present study was to determine potential bioactive lipids which may contribute to green light analgesia.

**Methodology**: Knee joint OA was induced in male and female Wistar rats (200–320 g) by intra-articular injection of sodium monoiodoacetate (MIA; 3 mg/50 μl). On days 9–14 following OA induction, rats were exposed to ambient green light (8 hr/day: wavelength: 455 nm; luminance: 20Lux). Control animals were treated with white light. Following five days of light treatment, serum samples were then taken and the concentration of various biolipids (N-acyl-ethanolamines, glycines, and 2-acyl-glycerols) were measured using HPLC MS/MS.

**Results**: Compared to white light, exposure to green light caused an upregulation of biolipid mediators with potential analgesic properties. In female rats, green light caused a significant increase in serum palmitoylethanolamine (P = .019; *n*= 4 rats), arachidonyl glycine (P = .022), palmitoyl glycine (P = .024), and stearoyl glycine (P = .023). In male rats, green light caused elevated levels of oleoyl glycine (P = .021), arachidonic glycine (P = .025), palmitoyl glycine (P = .021), linoleoyl glycine (P = .037), and stearoyl glycine (P = .034). None of the glycerols tested were different between the treatment groups.

**Discussion/Conclusions**: Treatment of OA rats with green light caused an upregulation in circulating bioactive lipids with putative analgesic properties. These data suggest that green light therapy produces a centrally driven analgesic effect which may in part be mediated by elevated levels of analgesic endolipids.

**Understanding the impact of wait times on individuals with chronic neuromusculoskeletal pain referred to the chronic pain center: Opportunity to improve care**

Magali Robert^a^, Karen Moffat^a^, Melissa Urquhart^b^, Rebecca Job^b^ and Tina Samuel^b^

^a^University of Calgary; ^b^Alberta Health Services

**Introduction/Aim**: As health is known to deteriorate as wait times increase,^2^ the impact on human suffering is significant. A wait time of longer than 6 months is considered unacceptable.^2^ The Calgary Chronic Pain Center (CPCC) is a public funded ambulatory clinic with wait times exceeding 12-months. To improve access to care, exploring the needs and understanding of people on the waitlist is the first step in addressing how to manage waitlist.

**Methodology**: A 15-minute, anonymous, voluntary, single-paged web-based survey sent to waitlisted patients. Basic demographics were collected. Other questions explored the pain experience, understanding of what the Center offers, what their expectations were, impact of being on the waitlist, and proposed solutions.

**Results**: Data collection in ongoing. Presently, out of 77 contacted patients, 24 answered the invitational call. Completed survey response rate was 83%. Respondents were mostly Caucasian (75%), female (90%), average age of 47.6 years, and unable to work (60%). 85% had not been told what the program offered. Expectations from the program:

- Don’t know (6, 33.3%)
- Education and/or management strategies (11, 61.1%)
- Medications and/or interventions (10, 55.5%)
- Free services (5, 27.8%)
- Diagnosis (9, 50.0%)

What would benefit from:

- understand what the CPCC offers (15, 75.0%)
- community resources (11, 55.0%)
- online resources (9, 45.0%)
- “updates regularly on where I am on the wait list” (15, 75.0%).

**Discussion/Conclusions**: Results from this cohort suggest that people on a chronic pain center waitlist need information on the program, existing pain resources and an understanding of chronic pain (PNE).

**Celebrating ten years of ECHO Ontario Chronic Pain and Opioid Stewardship**

Andrea Furlan^a^, Jane Zhao^a^, Everton Smith^b^, Paul Taenzer^c^, Leslie Carlin^b^, Ralph Fabico^a^, Rhonda Mostyn^a^, Kiera Morgan^a^, Andrew Smith^d^ and John Flannery^a^

^a^Toronto Rehab, University Health Network, ^b^University of Toronto, ^c^Queens University, ^d^Centre for Addiction and Mental Health

**Introduction/Aim**: In 2014, ECHO (Extensions for Community Healthcare Outcomes) was introduced to Canada by a collaboration between the University Health Network (UHN) in Toronto and Queen’s University in Kingston, ON. ECHO-Ontario Chronic Pain offered weekly videoconference sessions about chronic pain management and opioid stewardship in Ontario’s rural, remote, and underserved areas. The ECHO strategy employs a hub-and-spoke model that includes both (de-identified) case-based discussions and short didactic presentations. In this presentation, we review the implementation, dissemination, and impact of ECHO Chronic Pain at UHN/Queen’s across a decade and consider future directions.

**Results**: Since 2014, ECHO Chronic Pain has completed 20 cycles for a total of 419 sessions, including 924 participants, 22,600+ hours of Continuing Professional Development (CPD) credits, and 573 case presentations. Participants’ professions were 271 physicians, 240 nurses, 413 others (pharmacy, rehabilitation, psychology, etc). Participants came from all geographical areas of Ontario and attended, on average, 12 sessions. We also organized 11 in-person events, which were attended by 300+ people. Over the past 10 years, we received funding from a variety of sources for program planning (CIHR planning grant), implementation (Ontario’s Ministry of Health, Health Canada SUAP), dissemination (Ontario Medical Association), and evaluation (CIHR PHSI, NOAMA, CIHR Opioid Crisis, and CIHR THINC). Our group published 10 peer-reviewed scientific papers demonstrating the impact and effects of ECHO on healthcare providers’ knowledge, confidence, competence, self-efficacy, and opioid prescribing behaviors.

**Discussion/Conclusions**: ECHO chronic pain and opioid stewardship is a sustainable platform for knowledge mobilization in Canada.

**Validation of the Borsook Stress Assessment Tool (BSAT) in preoperative hernia patients**

Marguerite Mainprize^a^, Anthony Vaccarino^b^, Heather Lumsden-Ruegg^c^, Anton Svendrovski^d^ and Joel Katz^c^

^a^Shouldice Hospital; ^b^indoc Systems; ^c^York University; ^d^UZIK Consulting Inc.

**Introduction/Aim**: The BSAT was developed to measure stress based on life events and emotions. The present study aimed to assess the validity of the BSAT in preoperative hernia patients and determine whether BSAT scores were associated with postoperative pain severity.

**Methodology**: The BSAT is a 13-item self-report measure of stress (past 6 months). Items are rated on a 7-point Likert scale (1-very rarely to 7-very often). The BSAT was administered to 1140 patients the day before surgery. Postoperative measures included the Brief Pain Inventory, assessed at 2-weeks and 3-months after surgery. Rasch Measurement Theory (RMT) was used to assess the measurement properties of the BSAT, including goodness-of-fit and reliability. The study was approved by the Research Ethics Board at York University and patient consent obtained.

**Results**: The BSAT showed good internal consistency (Cronbach’s alpha = .82), with most of the BSAT items showing favorable goodness-of-fit characteristics. However, some items did not fit the Rasch model and thus added unwanted noise. Furthermore, preliminary analysis showed that patients with higher BSAT scores had higher levels of postoperative pain at 2-weeks and 3-months, as compared to those with low BSAT scores (*p* <.05).

**Discussion/Conclusions**: The BSAT generally showed acceptable goodness-of-fit properties, with RMT providing indications of where the scale could be improved. Stress, as measured by the BSAT, was related to postoperative pain. Scale improvement and analysis are underway to assess the relationship between BSAT scores and demographic/clinical variables. The identification of variables, such as stress, that influence postoperative pain is valuable in developing approaches to minimize pain after surgery.

**General and orthopedic surgery residents’ knowledge and attitudes of pain management**

Adam J. Burcheri^a^, Claire Galvin^a^, Nelson Piché^b^, Michael J. Frett^c^, Kevin Alschuler^d^ and Nicole M. Alberts^a^

^a^Concordia University, Montréal, Canada, ^b^Centre Hospitalier Universitaire Sainte-Justine, Montréal, Canada; ^c^St. Jude Children’s Research Hospital, Memphis, USA; ^d^University of Washington, Seattle, USA

**Introduction/Aim**: Appropriate knowledge in pain management is essential for surgery residents who must continually provide pain treatment for patients. Despite this, no studies have examined pain knowledge and attitudes among orthopedic and general surgery residents.

**Methodology**: General (*n* = 73) and orthopedic (*n* = 37) surgery residents (*N* = 110, median [*SD*] age = 29.9 [3.5] years, 56.4% female) recruited from 27 accredited residency programs across Canada completed a sociodemographic/background survey, and the Knowledge and Attitudes Survey Regarding Pain (KASRP), a validated 41-item measure of pain knowledge and attitudes in health care providers. Descriptive statistics examines level of pain knowledge (% correct on the KASRP), while a multiple linear regression analysis examined associations among pain knowledge and attitudes and resident factors (e.g., sex, specialty).

**Results**: Overall, surveyed residents demonstrated adequate pain knowledge, scoring 75.1% (*SD *= 8.6, range = 43.9–95.1) on average on the KASRP. Performance on pediatric and adult pain content also appeared adequate with residents scoring 86.5% (*SD* = 17.3, range = 11.1–100.0), and 74.0% (*SD*-8.9, range = 41.0–94.9), respectively on these domains. The two items most often answered incorrectly pertained to opioid tolerance (87.3% incorrect) and opioid administration (84.6% incorrect). Personal experience with postsurgical pain (*β* = .24, *p*= .01) was significantly associated with greater pain knowledge.

**Discussion/Conclusions**: Overall, Canadian orthopedic and general surgery residents appear to possess adequate knowledge of pain management when assessed using a validated survey. Nonetheless, gaps and a large amount of variability in knowledge were observed, especially for pediatric pain. Future research is needed to further examine these gaps, and to better characterize how pain knowledge impacts clinical practice.

**Single nuclei transcriptomic analysis in the prefrontal cortex in a neuropathic pain mouse model**

Peter Lee, Taylor Yeater, Maureen Riedl, Lucy Vulchanova, and Laura Stone

University of Minnesota

**Introduction/Aim**: The prefrontal cortex (PFC) plays a crucial role in the processing and modulation of pain processing. We previously reported changes in gene expression in mouse PFC following nerve injury; however, the cell phenotypes driving these changes have not been studied. *The goal of this study was to identify nerve injury-induced cell type-specific changes in gene expression using a single nuclei RNA sequencing approach.*

**Methodology**: Spared nerve injury (SNI) was induced in 10–12-week-old C57BL6 male mice to model neuropathic pain by ligating the common peroneal and tibial nerves. Sham surgery consisted of exposing the nerve. Mechanical and cold sensitivity on the ipsilateral hind paws were monitored for 6 weeks. PFC was collected for single nuclei RNA sequencing and data was analyzed by the R package Seurat (v4.3). Pathway enrichment was assessed via GO database.

**Results**: SNI induced persistent hypersensitivity to mechanical and cold stimuli. Multiple excitatory (*Slc17a7*+) and inhibitory (*Gad 1, 2*+) neuronal subpopulations and nonneuronal cell populations (i.e., microglia, astrocytes, oligodendrocytes) were identified. The majority of differentially expressed genes were detected in neuronal populations. *Oprm1* was downregulated in excitatory subpopulations while *Vgf* was upregulated in inhibitory neurons following SNI. Pathway analysis revealed enrichment in pathways related to calcium ion transport in excitatory neurons and macromolecule biosynthetic processes in inhibitory neurons.

**Discussion/Conclusions**: Cell type-specific changes in gene expression in PFC following nerve injury could contribute to the establishment and persistence of chronic neuropathic pain. Identifying these genes and pathways may contribute to the identification of new therapeutic targets.

**Strategies for improving pain management and minimizing opioid harm along the patient surgical journey**

Aaron Sihota^a, b^, Alan Bell^c^, Laz Klein^d, e, f^, Maxwell Slepian^g^, Valia Lestou^h^ and Hance Clarke^g, i, j, k^

^a^Faculty of Pharmaceutical Sciences, University of British Columbia, Vancouver, BC, Canada; ^b^Primary Care Pharmacist, Urgent Primary Care Center Vancouver, BC, Canada; ^c^Department of Family and Community Medicine, University of Toronto, Toronto, ON, Canada; ^d^Department of Surgery, University of Toronto, Toronto, ON, Canada; ^e^Family medicine, Humber River Regional Hospital, Toronto, ON, Canada; ^f^General Surgery, Humber River Regional Hospital, Toronto, ON, Canada; ^g^Department of Anesthesia and Pain Management, Toronto General Hospital, Toronto, ON, Canada; ^h^Emergent Biosolutions Inc., Gaithersburg, MD, USA; ^i^Transitional Pain Service, Department of Anesthesia and Pain Management, Toronto General Hospital, Toronto, ON, Canada; ^j^Department of Anesthesiology and Pain Medicine, University of Toronto, Toronto, ON, Canada; ^k^University of Toronto Center for the Study of Pain, University of Toronto, Toronto, ON, Canada

**Introduction/Aim**: The opioid crisis is a major public health concern, and it is vital to minimize the risk of harm from postsurgical opioid prescriptions. The roles that different healthcare professionals (HCP) play throughout the patient surgical journey and how these HCPs interact with the patient remain under-characterized within the context of mitigating potential harms of persistent opioid use and potential dependence. This aspect needs addressing in a detailed framework. The aims of this initiative are to 1) characterize the Canadian pre-, peri-, and postoperative patient pain journey, 2) identify the key patient-HCP touchpoints and define a collaborative, multidisciplinary model of care to mitigate opioid misuse and overdose, and 3) provide guidance on the potential strategies for harm reduction across the patient journey.

**Methodology**: We identified the challenges, unmet needs, and strategies at key patient-HCP touchpoints by participating in independent, standardized interviews and group reviews. Information obtained in interviews was distilled, synthesized, and corroborated with literature in a multi-faceted approach.

**Results**: This novel information proposes a collaborative framework for establishing an effective care model to mitigate the development of postsurgical opioid use disorder. Strategies include improving education, communication, and follow-up for HCPs and patients at all stages of the patient journey.

**Discussion/Conclusions**: Our proposed framework can be implemented in practice to address substance misuse before it happens and may help prevent patients from developing opioid dependence. Effective integration of preventive practices and interventions across healthcare systems along the patient-surgical journey will help mitigate postsurgical opioid harm. 

**Remote live vs. prerecorded physical exercise training for the treatment of chronic low back pain: a pilot randomized controlled trial**

Carlos Gevers-Montoro^a^, Maxime Bergevin^b, c^, Florian Bobeuf^c^, Benjamin Pageaux^b, c^ and Mathieu Roy^a^

^a^Psychology Department, McGill University; ^b^École de kinésiologie et des sciences de l’activité physique (EKSAP), Université de Montréal; ^c^Centre de Recherche de l’Institut Universitaire de Gériatrie de Montréal

**Introduction/Aim**: Remote exercise training offers a promising alternative for managing chronic low back pain (CLBP), making treatment more accessible. However, the effectiveness of various remote delivery methods is unclear for this population. This pilot randomized controlled trial compares live group videoconference with individual prerecorded remote training sessions in patients with CLBP.

**Methodology**: Seventy-one patients with CLBP were randomly assigned to a control wait-list group or a 12-week training program delivered by a certified kinesiologist, either through live group videoconference or prerecorded video sessions. Depending on performance during baseline assessment, participants were allocated to one of three levels of training intensity for three weekly one-hour sessions. As primary outcome, participants completed the NIH minimal dataset (NIHMD) before and after the training program (or wait-list). The NIHMD was proposed to standardize assessments of impact, function, and emotional distress in CLBP.

**Results**: In our preliminary analyses, 44 patients (average age 50.3 years, 27 women) had completed the study. Those in both exercise groups experienced significant reductions in pain intensity ratings compared to the wait-list group (*p*= .003). However, live and prerecorded training sessions yielded similar results (*p*= .3). No between-group differences were observed in other NIHMD measures. Both exercise methods resulted in similar levels of satisfaction, effort, fatigue, pain, and affect (*p*>.05).

**Discussion/Conclusions**: Live group videoconference or prerecorded video sessions effectively reduced pain intensity in CLBP patients. These findings highlight the potential of remote exercise training as an effective intervention for CLBP management. Further research should explore the mechanisms behind these results and optimize remote training for better pain relief.

**A new addition to the pain curriculum stable**

Norm Buckley^a^, David Mercer^b^, Paul Taenzer^c^, Eleni Hapidou^a, d^ and Greg Tippin^a, d^

^a^McMaster University; ^b^Canadian Psychological Association; ^c^Queens University, ^d^Hamilton Health Sciences Corp.

**Introduction/Aim**: The Canadian Pain Care Forum [CPCF] is a national group at which diverse individuals and organizations with common interest in pain-care, advocacy, education, research and policy come together to share information.

https://pain-institute.healthsci.mcmaster.ca/about-us/canadian-pain-care-forum/

The group has been in operation since 2016, supported by the Michael G. DeGroote Institute for Pain Research and Care at McMaster University [IPRC]. One series of presentations focused on the pain content of healthcare professional curricula. A presentation from the Canadian Psychological Association [CPA] identified the fact that there is no formal inclusion of pain in graduate curricula or clinical psychology training and certification. It is highly dependent upon the local interests and availability of clinical practice settings in the training center.

**Methodology**: Following the CPA presentation to the CPCF, a working group was struck to develop a pain program for clinical psychologists. Sponsored jointly by the CPA and the IPRC, the authors are creating a series of recorded online presentations with pre- and postself-assessments.

**Results**: The content includes the biopsychosocial model of pain, the interdisciplinary approach to treatment and the role of each profession in the team, the role of the psychologist in pain care, the pain assessment, theoretical principles underlying psychological interventions, evidence supporting these interventions, use of standardized assessment tools, description of specific techniques, and discussion of the creation of “virtual interdisciplinary teams” for the practitioner in community practice who may be collaborating in the management of complex patients. We expect the program to be available during the second quarter of 2024.

**Programme de mentorat pour accompagner les professionnels de soins primaires: prendre soin des Québécois atteints de douleur chronique, de problème de santé mentale et de dépendance !**

Anne Marie Pinard^a^, Orlane Ballot^a^ and Marylie Martel^b^

^a^Université Laval; ^b^Université de Sherbrooke

**Introduction/Objectif**: Les conditions de douleur, santé mentale et dépendance sont fréquentes. Un projet pilote de mentorat est mis en place au Québec pour fournir des soins bienveillants et de qualité.

**Méthodologie**: Ce programme permettra la discussion de cas complexes, le partage d’expériences et la réflexion sur la pratique clinique entre mentors et mentorés, via une plateforme en ligne et des rencontres aux six semaines. Des questionnaires et entrevues documenteront leurs expériences. Les mentors participeront à une formation en présentiel, et les mentorés compléteront un module en ligne avant le début du programme.

**Résultats**: Sept mentors experts de diverses professions en réadaptation, santé mentale et dépendance des RUISSS-UL et Estrie-CHUS soutiendront 20 professionnels de première ligne durant une année. La formation des mentors permet de revoir les fondements du mentorat, les qualités, rôles et responsabilités de chacun, en théorie et en atelier. 100% des mentors sont satisfaits du contenu, de la forme et des méthodes pédagogiques utilisées. Les commentaires sont positifs: *“Tout a été mis en place pour faire naître un sentiment d’engagement et de solidarité au sein de l’équipe*.”

**Discussion/Conclusions**: Les mentors sont très enthousiastes de participer à ce programme, et la formation offerte a permis d’assoir les bases du programme. Les rencontres et l’utilization de la plateforme vont débuter incessamment. Nous allons documenter la satisfaction et les impacts du programme pour les mentors et les mentorés. À long terme, ce programme permettra d’améliorer la qualité des soins des patients vivant avec ces difficultés, réduisant ainsi les listes d’attentes.

**Becoming a data detective: Bots, scammers and sham participants in online research recruitment and data collection**

Michelle Lamont^a^, Rachel Kelly^b^, Ligyana Candido^a^, Tristan Phair^a^, Paula Forgeron^a^, Bruce Dick^c^, Jennifer N. Stinson^b^, Kathryn A. Birnie^d^, G. Allen Finley^e^, Abbie Jordan^f^, Pamela Qualter^g^, Cassidy Bradley^h^, Delane Linkiewich^h^, Trinity Lowthian^h^, Samuel McNally ^h^ and Natasha Trehan^h^

^a^University of Ottawa; ^b^The Hospital for Sick Children; ^c^University of Alberta; ^d^University of Calgary; ^e^Dalhousie University; ^f^University of Bath; ^g^University of Manchester; ^h^Patient Partner

**Introduction/Aim**: Social media recruitment is popular, especially since the COVID-19 pandemic when most research resorted to online recruitment and data collection. However, researchers have seen an increase in fraudulent participation (e.g., bots, scammers and sham participants), Our study, designed to understand the types and impact of peer loneliness amongst adolescents with chronic pain in the context of the COVID-19 pandemic was not immune to this problem. The prevalence of all types of fraudulent participants led our team to streamline and develop new methods to identify and exclude these participants. We aim to share collaborative methods of detecting and removing data from fraudulent participants.

**Methodology**: Due to the mixed-method nature of our study, both quantitative and qualitative strategies were developed to deter, detect, and exclude fraudulent participants. Quantitative strategies included use of a *CAPTCHA*, double data verification questions, and data surge screening (e.g., 10 surveys completed within 10 minutes of each other). Qualitative strategies included mandatory use of Zoom cameras, data verification questions, and verification with photo identification.

**Results**: During the quantitative survey phase 1, 279 survey results were excluded by screening for data surges, 103 from invalid postal codes, 882 from data surges *and* invalid postal codes and 53 from double data verification questions. During the qualitative survey phase, two interviews were excluded due to suspected double participation with their corresponding survey data removed from the study.

**Discussion/Conclusions**: To ensure research data quality from social media recruitment, education, and development of detection strategies to identify and remove fraudulent participant data is essential.

**The impacts of patient engagement at the Chronic Pain Network: Results of an exploratory evaluation case study**

Delane Linkiewich^a, b^, Laura Tripp^c^, Dawn P. Richards^a, d^, Jennifer Daly-Cyr^a^, Therese Lane^a^, Kimberly N. Begley^a^, Norman Buckley^a, e^, Maria Hudspith^a, f^, Patricia Poulin^g, h, I^ and Julia Abelson^c^

^a^Chronic Pain Network, McMaster University, Hamilton, Ontario, Canada; ^b^Department of Psychology, University of Guelph, Guelph, Ontario, Canada; ^c^Public and Patient Engagement Collaborative, Department of Health Research Methods, Evidence and Impact, McMaster University, Hamilton, Ontario, Canada; ^d^Five02 Labs Inc, Toronto, Ontario, Canada; ^e^Department of Anesthesia, Faculty of Health Sciences, McMaster University, Hamilton, Ontario, Canada; ^f^Pain BC, Vancouver, British Columbia, Canada; ^g^The Ottawa Hospital Pain Clinic, Ottawa, Ontario, Canada; ^h^The Ottawa Hospital Research Institute, Ottawa, Ontario, Canada; ^i^Department of Anesthesiology and Pain Medicine, Faculty of Medicine, University of Ottawa, Ottawa, Ontario, Canada

**Introduction/Aim**: The Chronic Pain Network (CPN) is a Canadian research network funded by CIHR’s Strategy for Patient-Oriented Research. A main tenet of the CPN is to collaborate with patient partners across Network activities (e.g., research, governance). The CPN’s Patient Engagement (PE) Committee is mandated to oversee PE across the network and wished to evaluate the CPN’s PE strategy. McMaster University’s Public and Patient Engagement Collaborative (PPEC) acted as external evaluators. The positive and negative, intended, and unintended impacts of the CPN’s PE strategy, on those engaged in the Network, and the broader Canadian pain research community were evaluated.

**Methodology**: PPEC team members conducted semi-structured interviews via Zoom or telephone with CPN staff, committee/governance members (i.e., patient partners and researchers), and funded researchers at three time points. Content analysis was conducted at each time point. A final round of deductive content analysis was conducted, informed by the Engage with Impact Toolkit, to determine the impacts of the CPN’s PE efforts.

**Results**: Impacts were identified in seven areas: building community; developing knowledge, skills and resources; increasing confidence; influencing priorities and decisions; enabling additional opportunities; promoting culture change; and coping with experiences of chronic pain. Following the evaluation, the PE Committee identified key knowledge mobilization messages from the evaluation to implement in Phase 2 of the CPN.

**Discussion/Conclusions**: The CPN’s PE efforts had wide-reaching impacts from the individual and Network levels to the broader pain research community. Although there were challenges, the beneficial impacts highlighted in this study demonstrate the value of engaging patient partners in a research network.

**Interprofessional learner impacts of a Project ECHO® pilot series focused on opioid management and substance use/misuse for pediatric pain**

Chitra Lalloo^a, b^, Lauren Harris^a^, Naiyi Sun^c, d^, Sara Klein^d^, Marco Battaglia^e, f^, Karen Leslie^g, h^, Andrea Furlan^i, j^, Jane Zhao^i^, Yalnee Shantharam^i^, Quinn Casuccio-Treen^i^ and Jennifer Stinson^a, k^

^a^Child Health Evaluative Sciences, The Hospital for Sick Children; ^b^Institute for Health Policy, Management and Evaluation, University of Toronto; ^c^Department of Anesthesia and Pain Medicine, University of Toronto; ^d^Department of Anesthesia and Pain Medicine, The Hospital for Sick Children; ^e^Cundill Center for Child and Youth Depression, Center for Addiction and Mental Health; ^f^Department of Psychiatry, University of Toronto; ^g^Division of Adolescent Medicine, The Hospital for Sick Children; ^h^Department of Pediatrics, University of Toronto; ^i^Toronto Rehabilitation Institute, University Health Network; ^j^Department of Medicine, University of Toronto; ^k^Lawrence Bloomberg Faculty of Nursing, University of Toronto

**Introduction/Aim**: There are gaps in healthcare provider (HCP) knowledge and confidence related to opioids and controlled substances for pediatric pain management. To address these gaps, we aimed to leverage the Project ECHO® model to pilot and evaluate a specialized virtual education series for interprofessional HCPs.

**Methodology**: The pilot series consisted of 10-weekly online sessions delivered using the infrastructure of Pediatric Project ECHO for Pain®. Each 60-minute session included a didactic presentation followed by a de-identified case presentation. Pre- and postseries outcomes were collected using REDCap. Self-efficacy in 15 relevant skills was captured using 7-point Likert scales ranging from “strongly disagree” to “strongly agree.” Knowledge was assessed using an investigator-developed quiz and the KnowPain-12. Each knowledge tool yielded a sum score ranging from 0–100%. Paired *T*-tests examined pre/postchanges in self-efficacy and knowledge (alpha = 0.05).

**Results**: Sample included *N* = 43 HCPs (84% female identity; mean 17.4 ± 11 years of practice). Represented professions included nursing (47%), medicine (37%), pharmacy (2%), rehabilitation therapy (2%), social work (2%), and other (10%). Most learners (54%) were new to Project ECHO® and attended an average of 3.3 ± 2.5 sessions. Significant improvements in self-efficacy were observed across all (15; 100%) assessed skills. Similarly, significant improvements in knowledge were demonstrated for the series-specific tool [*t*(40) = 2.801, *p* = .008] and KnowPain-12 [*t*(40) = 4.870, *p* < .001].

**Discussion/Conclusions**: This pilot series was impactful in improving self-efficacy and objective knowledge among interprofessional HCPs with an interest in opioid management and substance use assessment for pediatric pain. Series curriculum topics will be integrated into the ongoing Pediatric Project ECHO® for Pain program.

**Sex and gender differences in health information needs for arthritis patients**

Tania Al-Jilawi^a^, Joy Christine^a^, Katherine Salter^b^ and Armaghan Dabbagh^c^

^a^Western University; ^b^Dalhousie University; ^c^University of Toronto

**Introduction/Aim**: The onset and progression of both degenerative and inflammatory arthritis can be affected by sex and gender (Maranini et al., 2022) and both age and gender can affect people’s decisions about their health (Xie et al. 2014). Men, for instance, may employ fewer, and less varied coping mechanisms than women (Englbrecht et al., 2012).

To focus on the health information needs of arthritis patients and investigate the experiences of arthritis patients, and how they think their sex and/or gender might influence their health information needs.

**Methodology**: This study followed interpretive description, where 13 participants were recruited from HULC at St. Joseph’s Health Center. The inclusion criteria were patients with confirmed arthritis aged 18–75, who could speak and understand English, and were able to consent to participate. Thematic analysis was conducted

**Results**: Ten overarching themes were identified with various subthemes. These themes were: positive therapeutical alliance with physicians, need for online resources, men are more reluctant to seek help, systemic challenges to accessing healthcare, patients’ health information needs, perceived facilitators, level of satisfaction with the information/services provided by physicians, gender affects information needs but not the ability to establish a therapeutic alliance, dire need for more access to arthritis information programs, and mixed understanding about the meaning of sex/gender.

**Discussion/Conclusions**: The demands of patients for health information are influenced by their gender and/or sexual orientation. Given the challenges patients have while accessing health information, it is critical to adopt a patient-centered strategy that focuses on their needs and allows them to express their opinions.

**The role of fear of movement and pain catastrophizing in Complex Regional Pain Syndrome (CRPS)**

Maryam Farzad^a^, Joy MacDermid^a^ and Tara Packham^b^

^a^Western University; ^b^McMaster University

**Introduction/Aim**: Complex Regional Pain Syndrome (CRPS) is recognized for its severe pain and functional limitations, often initiated by trauma or surgical interventions. The uncertainty surrounding CRPS’s pathophysiology points toward psychological factors, like fear of movement and pain catastrophizing, as potential contributors to its development and chronicity. The study sought to evaluate the psychometric characteristics of the Persian version of the Tampa Scale for Kinesiophobia-11 (TSK-11) among individuals with upper limb CRPS, focusing on the scale’s ability to reflect the original model of “activity avoidance” and “somatic focus.”

**Methodology**: Engaging 142 upper limb CRPS sufferers (average age 42, 54% female), this research utilized the TSK-11 to measure fear of movement. The process included testing for internal consistency, test-retest reliability (via intraclass correlation), and construct validity, alongside confirmatory and exploratory factor analyses for structural validity.

**Results**: The TSK-11 showed high internal consistency (Cronbach’s alpha = 0.93) and excellent test-retest reliability (ICC = 0.93). Despite strong criterion validity (*r* = 0.81), the confirmatory factor analysis did not support the original two-factor model. Instead, an exploratory factor analysis suggested a revised two-factor structure, capturing Fear Avoidance beliefs and Magnification/Helplessness, explaining a significant variance portion and maintaining high internal consistency.

**Discussion/Conclusions**: While confirming the TSK-11ʹs reliability for assessing fear of movement in upper limb CRPS patients, the study reveals a discrepancy with the original model, introducing a novel two-factor structure of Fear Avoidance and Magnification/Helplessness. This finding necessitates further research to delineate these constructs’ distinct roles in CRPS, aiming to enhance diagnostic and treatment approaches for this complex condition, thereby improving patient outcomes.

**The effect of aquatic therapy on paraspinal and gluteal muscle strength and psychological outcomes in individuals with chronic low back pain: Preliminary findings**

Chanelle Montpetit^a^, Nicolas Vaillancourt^a^, Brent Rosenstein^a^, Geoffrey Dover^a, b^, Najmeh Khalini-Mahani^c^, Christina Weiss^b^, Lee Ann Papula^b^, Antonys Melek^b^ and Maryse Fortin^a, b, d^

^a^Department of Health, Kinesiology and Applied Physiology, Concordia University, Montreal, Canada; ^b^School of Health, Concordia University, Montreal, QC, Canada; ^c^McGill Center for Integrative Neuroscience, Montreal Neurological Institute, Montreal, QC, Canada; ^d^CRIR – Center de réadaptation Constance‐Lethbridge du CIUSSS COMTL, Montreal, QC, Canada

**Introduction/Aim**: Chronic low back pain (cLBP) is associated with impaired paraspinal and gluteal muscle function. Despite exercise therapy being a first-line conservative treatment for cLBP, individuals often experience fear-avoidance beliefs hindering their physical activity engagement. Aquatic therapy, by reducing spinal loading, promotes movement capacity and facilitates exercises otherwise challenging on land. To our knowledge, no studies have explored the effects of aquatic therapy on paraspinal/gluteal strength and psychological outcomes.

To investigate the effects of an aquatic therapy (AT) exercise intervention versus standard care (SC) on **1)** paraspinal and gluteal strength; **2)** patient-oriented outcomes (pain, disability, and psychological factors).

**Methodology**: This pilot study is part of a larger, ongoing randomized controlled trial; preliminary results are presented. Thirty-four participants with nonspecific cLBP were randomly assigned to each group (AT *n* = 18; SC *n* = 16) groups. Both groups completed a 10-week supervised intervention program (2 sessions/week). Baseline and postintervention assessments included lumbar extension (MedEx) and gluteal (hand-held dynamometer) strength tests, and self-reported questionnaires. Two-way repeated measures ANOVA were used to assess changes in muscle and patient-oriented outcomes within and between-groups.

**Results**: Both groups significantly increased lumbar extensor strength (AT *p* <.001; SC *p* = .003), gluteus maximus strength (both *p*<.001) and gluteus medius strength (both *p* <.001) across timepoints. The AT and SC groups saw a significant decrease in pain (*p* <.001), disability (*p*<.001), and pain catastrophizing (*p* <.001), with no difference across groups for all self-reported outcomes.

**Discussion/Conclusions**: The AT group demonstrated a significant increase in strength and patient-oriented outcomes, highlighting its potential for improving musculoskeletal health and well-being in individuals with cLBP.

**Psychometric evaluation of the Hamilton Inventory to evaluate signs and symptoms in patients with CRPS**

Maryam Farzad^a^, Joy MacDermid^a^ and Tara Packham^b^

^a^Western University; ^b^McMaster University

**Introduction/Aim**: Complex Regional Pain Syndrome (CRPS) is a debilitating condition with profound physical and psychological impacts, necessitating comprehensive assessment tools for effective evaluation.

This study aimed to validate the Persian version of the Hamilton Inventory for Complex Regional Pain Syndrome (HI-CRPS) and assess its reliability and validity in individuals diagnosed with CRPS.

A cross-sectional clinical measurement study evaluated the Persian version of HI-CRPS.

**Methodology**: A sample of 64 individuals diagnosed with CRPS from pain and hand surgeon clinics completed the Persian versions of the PR-HI-CRPS and CB-HI-CRPS. Test-retest reliability was assessed after one week, and responsiveness was measured after three months. Baseline scores, ceiling effects, internal consistency (Cronbach’s alpha), and construct validity (correlations with related measures) were examined. Effect sizes and standardized response means (SRM) were calculated to gauge responsiveness.

**Results**: Baseline scores for PR-HI-CRPS and CB-HI-CRPS were 77.8 and 14.9, respectively; 18% of PR-HI-CRPS and 16% of CB-HI-CRPS respondents exhibited ceiling effects. Internal consistency for PR-HI-CRPS (Cronbach’s alpha: 0.71–0.91) and CB-HI-CRPS (alpha: 0.90) was satisfactory. PR-HI-CRPS (ICC: 0.86) and CB-HI-CRPS (ICC: 0.97) showed robust test-retest reliability. Construct validity was confirmed by significant correlations between PR-HI-CRPS subscales and related measures (*p* <.01). PR-HI-CRPS displayed an effect size of 0.79 and a standardized response mean (SRM) of 0.88.

**Discussion/Conclusions**: The Persian version of the HI-CRPS demonstrated satisfactory internal consistency, test-retest reliability, construct validity, and responsiveness. It can be relied upon to assess CRPS symptoms, functional limitations, and psychosocial impacts.

**Pathways of military sexual trauma and other forms of abuse affecting chronic pain in men and women active military members and Veterans**

Joy C. MacDermid^a^, Dimitra V. Pouliopoulou^a^, David M. Walton^a^, Angel Kibble^b^ and Pavlos Bobos^a^

^a^Western University; ^b^Veteran with Lived Experience

**Introduction/Aim**: This study evaluated gendered risk and pathways between psychological distress, military sexual trauma and other forms of abuse, and severe pain in active military personnel and Veterans in Canada.

**Methodology**: A cross-section of 328 men and women active military members and Veterans with chronic pain responded to confidential surveys including the Brief Pain Inventory, Patient Health Questionnaire (depression/ anxiety), and Single Assessment Numeric Evaluation (function). Unadjusted and adjusted odds ratios [OR] described the gendered risks of trauma exposures. Structural Equation Modeling quantified pathways between psychological distress, sexual trauma, and pain intensity levels.

**Results**: BPI scores (6.8 or 6.9/10), PHQ scores (6.6 or 6.2/12), and SANE scores (11% or 10%) indicate high burden and were similar for women and men. Women had high exposure to sexual harassment (69%), emotional abuse (76%), and repeated forced sexual activity (60%) compared to men (8, 13, and 9%); and a higher risk of experiencing sexual harassment (OR 28.6, 95%CI 13.6 to 60.2), emotional abuse (OR 24.8, 95%CI 11.9 to 51.7), verbal abuse/harassment (OR 2.1, 95%CI 1.3 to 3.6), and physical abuse (OR 2.2, 95%CI 1.3 to 3.8). Sexual assault was reported by 12% of men and 6% of women (OR 0.48, 95%CI 0.19 to 1.22). Sexual harassment and sexual assault were directly associated with psychological distress levels as a pathway to severe pain (R^1^ = 0.40).

**Discussion/Conclusions**: Women report much higher risk of experiencing sexual harassment and emotional abuse during military service. Harassment and discrimination are a pathway to severe chronic pain.

**A multivariable approach to defining acute low back pain**

Rachael Osagie^a, b^, Iulia Tufa^c,d^, Adriana Angarita-Fonseca^e, f^, M. Gabrielle Pagé^e^, Anaïs Lacasse^f^, Laura S. Stone^g^, Pierre Rainville^h^, Mathieu Roy^i^, Pascal Tétreault^j^, Maryse Fortin^k^, Guillaume Léonard^l^, Hugo Massé-Alarie^m^, Jean-Sébastien Roy^i^ and Carolina B. Meloto^a, c^

^a^Faculty of Dental Medicine and Oral Health Sciences, McGill University, QC, Canada; ^b^The Alan Edwards Center for Research on Pain, McGill University, QC, Canada; ^c^McGill University Health Center, QC, Canada; ^d^Quebec Pain Research Network, QC, Canada; ^e^Centre de recherche du Center hospitalier de l’Université de Montréal (CRCHUM); Département d’anesthésiologie et de médecine de la douleur, Université de Montréal, Montréal, QC, Canada; ^f^Département des sciences de la santé, Université du Québec en Abitibi-Témiscamingue (UQAT), Rouyn-Noranda, QC, Canada; ^g^Department of Anesthesiology, University of Minnesota, Minneapolis, MN, U.S.A; ^h^Faculté de médecine dentaire – Département de stomatologie, Université de Montréal, Montréal, QC, Canada; ^i^Department of Psychology, McGill University, Montreal, QC, Canada; ^j^Centre de Recherche du Center Hospitalier Universitaire de Sherbrooke (CHUS) – Department of Anesthesiology, Université de Sherbrooke, Sherbrooke, QC, Canada; ^k^Department of Health, Kinesiology and Applied Physiology, Concordia University, Montreal, QC, Canada; ^l^Research Center on Aging, CIUSSS de l’Estrie-CHUS; School of Rehabilitation, Université de Sherbrooke, Sherbrooke, QC, Canada; ^m^School of Rehabilitation Sciences – Université Laval and Center for Interdisciplinary Research in Rehabilitation and Social Integration, Quebec City, QC, Canada

**Introduction/Aim**: The NIH Task Force for Research Standards for Chronic Low Back Pain recommends defining cLBP as a back pain problem that has persisted at least 3 months and has resulted in pain on at least half the days in the past 6 months. The lack of consistency of acute low back pain (aLBP) definition across studies has likely contributed to observed inaccuracies in predicting chronification. The goal was to evaluate aLBP definitions using a multivariable approach.

**Methodology**: We composed 3 aLBP groups using baseline data from the Quebec Low Back Pain Study. 1) nonchronic: individuals not meeting the NIH recommendation for cLBP (*n* =788); 2) acute: individuals with LBP that had begun < 3 months ago (*n* = 230); 3) new episode: individuals experiencing a new occurrence that had begun < 3 months ago and was preceded by a pain-free period (n =182). At 6-months, individuals meeting the NIH criteria for cLBP were reclassified as cLBP, others as transient LBP (tLBP). We used mRMRe R package to rank variables and built logistic regression models using 10-fold cross-validation with caret R package. We then estimated the area under the receiver operating characteristic curve (AUROC) and confidence interval (CI).

**Results**: Top 4 variables were as follows: 1) nonchronic: physical function, sex, age, health-related quality of life; 2) acute: physical function, sex, educational attainment, health-related quality of life; 3) new-episode: health-related quality of life, age, sex, physical function. AUROC (CI) values were: 0.704 (0.66–0.75), 0.732 (0.66–0.81), and 0.765 (0.69–0.84), respectively.

**Discussion/Conclusions**: A more stringent aLBP definition enhances the predictive performance for predicting chronification.

**Loneliness and pain-related outcomes: Are friendships protective?**

Paula Forgeron^a^, Ligyana Candido^a^, Bruce Dick^b^, Jennifer Stinson^c^, Kathryn Birnie^d^, G. Allen Finley^e^, Abbie Jordan^f^, Pam Qualter^g^, Cassidy Bradley^h^, Delane Linkiewich^h^, Trinity Lowthian^h^, Samuel McNally^h^, Natasha Trehan^h^, Michelle Lamont^a^

^a^School of Nursing, Faculty of Health Sciences, University of Ottawa; ^b^Departments of Anesthesiology and Pain Medicine, Psychiatry and Pediatrics, Faculties of Medicine and Dentistry and Rehabilitation Medicine, University of Alberta; ^c^Child Health Evaluative Sciences (CHES) in Research Institute, SickKids Pain Center, The Hospital for Sick Children; ^d^Department of Anesthesiology, Perioperative and Pain Medicine, and Department of Community Health Sciences, University of Calgary; ^e^Department of Anesthesia, Pain Management and Perioperative Medicine, Faculty of Medicine, Dalhousie University; ^f^Department of Psychology and Center for Pain Research, University of Bath; ^g^Manchester Institute of Education, University of Manchester; ^h^Patient Partner

**Introduction/Aim**: Enduring adolescent peer loneliness is linked to poor physical and mental health. Adolescents with chronic pain (ACP) experience more loneliness compared to peers, which persists over time. Healthy friendships are protective for social well-being, but little is known about the impact of friendships on loneliness and pain outcomes. This study examined if loneliness is a driver of worse pain-related outcomes amongst ACP and whether friendships are protective.

**Methodology**: A cross-sectional survey completed by 127 ACP (aged 12–18 years, experiencing pain >3 months) captured demographics, pain characteristics, loneliness, friendship factors, and pain outcomes. Regression and mediation analyses were conducted to determine loneliness effects on pain outcomes and if friendship factors mediated these relationships.

**Results**: Peer loneliness significantly predicted worse mental/social health outcomes beyond demographics (age, sex, SES, ethnicity) and pain interference (additional variance explained by loneliness: 31.1% depression, 26.6% anxiety, 39.7% self-esteem). School attendance was predicted by lower SES and greater pain interference, not loneliness. Friendship circle size mediated the relationship between loneliness and self-esteem regardless of sex. Supportive friendships and friendship circle size did not mediate the relationship between loneliness and other outcomes.

**Discussion/Conclusions**: Peer loneliness predicted worse pain-related outcomes. Friendship support did not mediate these relationships. Having more friends was helpful, suggesting companionship matters. Previous studies report that ACP did not find most friends understand chronic pain, suggesting unmet peer needs. Pain-peer support interventions targeting unmet peer needs may be helpful in reducing loneliness. Given the positive effects of friendships on adolescent loneliness further research to understand friends’ role in ACP loneliness is warranted.

**The impact of face ethnicity on the discrimination of facial expressions of pain**

Alex Cousineau^a^, Gabrielle Kealey^a^, Daphnée Sénécal^a^, Diego Leblanc^a^, Marie-Pier Plouffe-Demers^a, b^, Daniel Fiset^a^ and Caroline Blais^a^

^a^Department of Psychoeducation and Psychology, University of Québec in Outaouais; ^b^Department of Psychology, University of Québec in Montréal

**Introduction/Aim**: Previous findings indicate that White participants perceive the intensity of facial expressions differently when assessing White versus Black faces. However, existing studies often rely on subjective tasks that are susceptible to response biases. To address this limitation, our study employs an experimental paradigm to investigate whether perceptual differences contribute to disparities in discriminating intensities of facial expressions among White participants toward Black faces. In addition to pain, we extend our examination to include facial expressions of anger and joy for comprehensive insights.

**Methodology**: White participants (*N* = 50 per emotion) completed a task in which they identified the strongest expression (anger, joy or pain) among two faces. The task included two conditions of intensity: low (ranging from 14 to 49%) and high (ranging from 54 to 89%). In each trial and under both conditions, the intensity gap between the two faces varied between 7% and 35%.

**Results**: Results for the low-intensity condition reveal that across all three emotions, participants exhibit superior discrimination abilities when evaluating facial expressions on White faces compared to Black faces. However, for the high-intensity condition, the discrimination performance for Black and White faces varies inversely across intensity gaps. For larger intensity gaps, participants show greater discrimination ability for Black faces, while for smaller gaps, they perform better with White faces.

**Discussion/Conclusions**: Results suggest that the alteration in the capacity to interpret the intensity of facial expressions, encompassing not only pain but also anger and joy, in faces of Black ethnicity, has a perceptual component.

**Publication trends related to the prevalence of chronic pain in children and adolescents from 2009 to 2023**

Justine Dol^a^, Jennifer Parker^a^, Perri Tutelman^a, b^, Brittany Cormier^a^ and Christine Chambers^a, b, c^

^a^IWK Health Center, Center for Pediatric Pain Research, Halifax, Canada; ^b^Dalhousie University, Department of Psychology and Neuroscience, Halifax, Canada; ^c^Dalhousie University, Department of Pediatrics, Halifax, Canada

**Introduction/Aim**: There has been significant growth in the number of publications on pain in children and adolescents. As part of an updated systematic review on the prevalence of pediatric chronic pain, the aim of this study was to conduct a bibliometric analysis of publications trends.

**Methodology**: Studies published between 2009 and 2023 on chronic, nondisease-related pain prevalence in individuals under 19 years of age were eligible. EMBASE, PubMed, CINAHL and PsycINFO were searched from January 1, 2009 to June 30, 2023. Data were analyzed in Excel and CitationChaser.

**Results**: 119 studies with 1,043,878 children (52% female, mean age 13.4 yrs [SD 2.4]) were included. Number of publications per year ranged from 4 (2023) to 11 (2014, 2021) with an average of 8/year. There were 74 unique journals, with most publishing one (*n* = 52, 70%) or two articles (n =13, 18%), with “Cephalalgia” publishing the most (*n* = 9, 12%). 70 different countries were represented, with the highest number of data points coming from Finland and Germany (*n* = 19, 4.3%), with 15 countries only having one data point. There were 109 different corresponding authors, with only one author who had more than two published articles. The studies were cited on average 35 times (*SD* =57, range 0–392) for a total of 3,193 unique citations.

**Discussion/Conclusions**: Despite a steady number of publications over a period of 14 years, the literature on the prevalence of pediatric chronic pain remains disjointed. Future work should focus on bringing together this important topic in pediatric pain and expanding evidence in underrepresented areas.

**Answering the call: The Family Medicine Enhanced Skills Chronic Pain Residency at the University of Calgary**

Ted Findlay

University of Calgary

**Introduction/Aim**: An estimated 7.6 million, or one in five people live with chronic pain, many of whom report it adversely affecting some or most daily activities. Pain impacts all demographics in Canada, although not equally. It is more common as we age, with approximately one in three people over 65 experiencing chronic pain. As Canada’s population ages, the prevalence of chronic pain and its impacts are expected to grow.

In response, the National Pain Strategy established GOAL #3: People living with pain and health professionals have the knowledge, skills, and educational supports to appropriately assess and manage pain based on population needs. The broader community understands pain as a legitimate, biopsychosocial condition and stigma is reduced.

- Empower leadership from University and College programs to create sub-specializations in pain management across all relevant health care professions.
- Expand postgraduate positions (e.g., residencies, postdoctoral fellowships) to train health professionals as pain specialists.

**Discussion/Conclusions**: In response to the Canadian Pain Task Force Report and its call for postgraduate training positions for pain specialists, and in keeping with physician requirements anticipated by the Alberta Pain Strategy, the Department of Family Medicine at the Cumming School of Medicine (University of Calgary) has established an Enhanced Skills program for family medicine residency graduates. This is a one-year program that will be based at the Calgary Chronic Pain Center. The first resident will begin training in July 2025.

Ref: Canadian Pain Task Force Reports 2019, 2020, 2021

**Symptom and function profiles of children and adolescents with cancer in China**

Danyu Li^a^, Jennifer Stinson^b, c^, Lindsay Jibb^b, c^, Wen Zhang^a^ and Changrong Yuan^a^

^a^School of Nursing, Fudan University, Shanghai, China; ^b^Child Health Evaluation Sciences, Research Institute, The Hospital for Sick Children, Toronto, ON, Canada; ^c^Lawrence S. Bloomberg Faculty of Nursing, University of Toronto, Toronto, ON, Canada

**Introduction/Aim**: Unpleasant symptoms and limited functions are common in children and adolescents with cancer. However, research identifying subgroups of children and adolescents with cancer who experience similar levels of distress and functional limitations in China is limited. This study aimed to: (1) classify the symptom and function profiles of children and adolescents with cancer and (2) detect the possible predictors of the profiles.

**Methodology**: A total of 148 parents of patients aged 5–17 years completed the Chinese version of the PROMIS-Parent/Proxy-25 to report mobility, anxiety, depressive symptoms, fatigue, peer relationships, pain interference, and pain intensity. Latent profile analysis was used to identify profiles. Binary logistic regression was used to examine predictors of profile membership.

**Results**: The best fit was a 2-profile model: less suffering and more suffering group (Fig. 1). Children who have parents without college degrees are more likely to be in the more suffering group. Other variables like children’s gender, age, ethnicity, religion, complication, parents’ education level, monthly family income, and parents’ marital status showed insignificant predictors of profile membership.

**Discussion/Conclusions**: Children with cancer are heterogeneous in their experience. The family characteristic of parental education level predicts membership. *Implications for Practice*: This study identified distinct groups of patients who predictably experience higher symptoms distress and more limited functions and their predictors, which could help to place patients within a profile and perhaps allow nurses to use adapting communication strategies or provide cancer care navigating to match children’s specific profile.

**Determining the feasibility, tolerability and safety of combining Repetitive Transcranial Magnetic Stimulation (rTMS) and Steroid Joint Injections (SJIs) for Chronic Spinal Pain (CSP): A pilot randomized controlled trial**

Anike Alarape^a^, Thivya Sivarajan^a^, Siobhan Schabrun^b^ and Eldon Loh^c^

^a^Graduate Program in Neuroscience, Faculty of Health Sciences, University of Western Ontario, London, ON, Canada and The Gray Center for Mobility and Activity, Parkwood Institute, London, ON, Canada; ^b^The Gray Center for Mobility and Activity, Parkwood Institute, London, ON, Canada, Lawson Health Research Institute, Parkwood Institute, London, ON, Canada, and School of Physical Therapy, Western University, London, On, Canada; ^c^ Lawson Health Research Institute, Parkwood Institute, London, ON, Canada

**Introduction/Aim**: Traditional treatments of chronic spinal pain (CSP) have only short-term analgesic effects. This study explores the feasibility, tolerability, and safety of combining repetitive transcranial magnetic stimulation (rTMS) and steroid joint injections (SJIs) as a novel approach to overcome the limitations of conventional interventions.

**Methodology**: In this ongoing study, 40 participants (≥18 years old, receiving SJIs) will be recruited. Eligible patients are randomized (1:1) to undergo 11 active or sham rTMS sessions within 12 weeks and are followed-up until week 24 or their next SJI. Drop-out rate, session completeness, and screening-to-recruitment ratio serve as measures of feasibility and tolerability. Safety is measured through adverse effects reports.

**Results**: To date 264 patients have been prescreened, of which 112 (58%) were eligible and 15 (13.4%) were recruited (8 female; mean age = 64.3, *sd* = 9.3). Of the 97 (86.6%) not enrolled, 74 (66.1%) were not interested due to distance (6.3%), time commitment (11.6%), or lack of interest in research (31.3%). Eighteen patients (16.1%) were not approached and five (4.5%) are undecided. Of the 15 enrolled, two (13.3%) withdrew. Session completeness is adequate for rTMS (90.5%, *SD *= 20.3%) and assessments (86.0%, *SD* = 28.9%). All patients tolerated the stimulation well, with no adverse effects reported.

**Discussion/Conclusions**: Our preliminary findings support the feasibility, tolerability, and safety of combining rTMS with SJIs for CSP patients, paving the way for a larger clinical trial. If successful, this approach may offer an effective, long-term intervention for individuals with CSP.

**Reliability of pain outcome measures**

Nathaniel De Vera, Spencer Abssy, Natalie Osborne and Massieh Moayedi^a^

Center for Multimodal Sensorimotor and Pain Research, University of Toronto

**Introduction/Aim**: Self-report pain intensity and pain unpleasantness ratings are common measures of intervention outcomes. The area of secondary hyperalgesia in response to an experimental noxious stimulus is thought to reflect central sensitization. There is a need to determine the test-retest reliability of these measures across multiple sessions of an identical intervention in pain-free individuals. Here, we aim to determine the test-retest reliability of subjective and objective pain outcome measures across multiple sessions over several day in pain-free individuals.

**Methodology**: Twenty-four healthy participants consented to procedures approved by the local Ethics Board. Participants attended three sessions, where they received a heat familiarization protocol (three 10s noxious stimuli: 43°C, 45°C, 47°C). Pain intensity and pain unpleasantness ratings on 0–100 verbal numerical rating scale (vNRS) were collected. Next, a phasic heat pain (PHP) protocol was performed. Ratings and the area of secondary hyperalgesia area were measured. Intraclass correlations (ICC), and repeated measures ANOVA were used to assess test-retest reliability.

**Results**: Pain intensity ratings were moderately reliable (ICC = 0.738–0.741). Pain unpleasantness ratings had good reliability (ICC = 0.750–0.811). Secondary hyperalgesia had good reliability (ICC = 0.845). We found significant differences for pain intensity and pain unpleasantness across sessions (*p* <.05), but not for secondary hyperalgesia across sessions in PHP (*p* >.05).

**Discussion/Conclusions**: Together, these data suggest multiple outcome measures are more reliable than a single outcome measure. Future studies should investigate factors that may contribute to differences in pain intensity across sessions (e.g., time of day, mood, etc). Furthermore, our data indicates the importance of counterbalancing sessions across participants to wash out any session effects.

**Improving individualized self-management: A 12-week online education intervention for chronic pain patients on waitlists in Canada**

Nina Gregoire^a^, Kimberley Kaseweter^a^, Mark Nazemi^b^, Madison Eagle^a^, Paul G. Davies^a^ and W. Francois Louw^a^

^a^University of British Columbia; ^b^Thrive Health

**Introduction/Aim**: In Canada, the scarcity of pain clinics, coupled with median wait times of up to four years often leads to patient mental health deterioration. Promisingly, pain education has demonstrated effectiveness in relieving mental health symptoms among individuals with chronic pain (CP). Although eHealth strategies enhance self-management, their implementation for supporting waitlisted patients is often overlooked or underutilized. Further, limited uptake may be partially attributed to the burden placed on patients in navigating available resources. A pilot study was conducted investigating a brief, 12-week online educational waitlist intervention.

**Methodology**: The sample comprised 10 CP patients who were waitlisted at a pain clinic in British Columbia. Participants completed a brief online baseline survey assessing knowledge of aspects of CP self-management (visual analogue scales), as well as anxiety and depression symptomatology (PROMIS-29), followed by 12 one-week educational modules and a follow-up survey at week 12. Modules were designed to take approximately 5 minutes per week. Additional resources were available within each module (e.g., supplemental podcasts/articles).

**Results**: Participants’ self-reported knowledge of CP management (*t*= 4.17, *p*= .002) and mental health

(*t*= 2.83, *p*= .02) significantly increased following the waitlist intervention. Additionally, 70% of participants exhibited no deterioration in anxiety and depression symptoms during the waitlist period, either staying at baseline level or experiencing reductions in symptomatology.

**Discussion/Conclusions**: Preliminary data on this brief online educational program is promising to help prevent mental health deterioration while waitlisted. Further examination is required with a larger sample and follow-up beyond the 12 weeks to determine the longevity of the effect.

**Prolonged opioid use after surgery: A scoping review**

Monakshi Sawhney^a^, Irina Nestor^b^ and Aryan Bawa^c^

^a^School of Nursing, Queen’s University; ^b^Mount Sinai Hospital, Toronto; ^c^University of Western Ontario

**Introduction/Aim**: The opioid misuse epidemic is a public safety crisis with personal prescriptions for opioids for pain following surgical or dental procedures are reported to account for a third of misuse or nonmedicinal drug use. Although patients are prescribed opioids after surgery, they are not routinely screened for risk of prescription opioid misuse. The challenge associated with prescription drug misuses is that opioids have a therapeutic effect in the management of surgical pain. The aim of this scoping review was to examine the risk factors for prolonged opioid prescription following surgery included published studies from 2018–2020.

**Methodology**: This scoping review was conducted following the JBI methodology. The study population included adults who underwent any surgical procedure who were opioid-naïve prior to their surgery. Our review considered research articles exploring the postoperative use of prescription opioids beyond three months. We performed a search in MEDLINE and EMBASE, using the keywords: “Risk assessment, Risk factors, Opioids*, Pain, and “Pain, postoperative.” All screening and extraction occurred by two independent researchers (MS and IN).

**Results**: A total of 78 studies were included. Up to 10% of people continue to fill a prescription for an opioid analgesic 90 days after surgery. Common risk factors for prolonged opioid used included being diagnosed with multiple comorbid medical conditions, having a cancer diagnosis, being diagnosed with a mental health issue such as anxiety, history of a substance use disorder.

**Discussion/Conclusions**: It is important that patients be assessed for the appropriateness of an opioid prescription past the acute pain phase.

**Gender-specific distinctions in encoding and decoding pain through facial expressions**

Arianne Richer^a^, Camille Saumure^b^, Daniel Fiset^a^, Zoé Glardon^b^ and Caroline Blais^a^

^a^University of Quebec in Outaouais; ^b^University of Fribourg

**Introduction/Aim**: Facial expressions play a crucial role in assessing others’ affective states. However, pain facial expressions (PFE) face challenges in recognition, often being confused with other negative affective states and being less easily perceived in women’s faces. Studies have identified various configurations of PFE, but it remains unclear whether some of these configurations are more easily recognizable, potentially explaining disparities in perceived pain based on face gender. This study investigates potential gender differences in the configurations of PFE (encoding) as well as their perception by external observers (decoding).

**Methodology**: We used the Delaware Pain Database (DPD), containing 225 pictures of White individuals posing PFE. To explore potential differences in PFE encoding between men and women, we used OpenFace to measure activation levels of 17 action units (AUs) in those pictures.

**Results**: Principal component analysis revealed five main groups of AUs with correlated activations. Most importantly, a component including AUs typically associated with PFE prominently emerged in men’s PFE. To assess whether PFE are decoded differently based on face gender, we used ratings of perceived intensity of six basic emotions and pain openly available within the DPD. A mixed ANOVA 7 (affective states) by 2 (genders) indicated significant main effects of both factors, as well as an interaction. *T*-tests revealed higher perceptions of fear and sadness in women’s PFE and a higher perception of pain in men’s.

**Discussion/Conclusions**: These findings emphasize gender-specific disparities in PFE, their overlap with other affective states, and underscore the potential contribution of both encoding and decoding in observed gender differences.

**Presurgical fMRI left hippocampal functional connectivity is associated with acute postsurgical pain in youth after major surgery**

Anna Waisman^a^, Karen Cobos^b^, Xiangyu Long^b^, Catherine Lebel^b^, Joel Katz^a^, Melanie Noel^b^ and Jillian Miller^a^

^a^York University; ^b^University of Calgary

**Introduction/Aim**: The incidence of chronic postsurgical pain in youth ranges from ~10–60%. Recent research has found greater regional global efficiency of the right hippocampus in youth with chronic headache pain. However, the temporal relationship between hippocampal functional connectivity and postsurgical pain remains unknown. To examine whether presurgical functional connectivity of the hippocampus is associated with pain in youth after surgery.

**Methodology**: 38 youth (10–18 years) undergoing major surgery were recruited at Alberta Children’s Hospital. Participants completed questionnaires assessing pain 7 days before, and 2 weeks and 4 months after surgery. Resting-state fMRI (rsfMRI) was collected before surgery. rsfMRI data was preprocessed using Analysis of Functional NeuroImages and the Oxford Center for fMRI of the Brain Software Library. Graph theory was used to derive nodal and modular network matrices for left and right hippocampus using the GRETNA toolbox.

**Results**: A 3-step hierarchical linear regression was conducted to evaluate the association between presurgical betweenness centrality (Step 3) and 2-week postsurgical pain intensity after controlling for age, sex, and presurgical pain intensity. In Step 3, presurgical betweenness centrality of the left hippocampus *t*(31) =2.15, *p*=.04 explained 10% of the variance in 2-week pain intensity scores after controlling for age (Step 1: *t*(34) =0.28, *p*=.78), sex (Step 1: *t*(34) =−0.08, *p*=.94), and presurgical pain intensity (Step 2: *t*(32) =3.32, *p*=.002).

**Discussion**/**Conclusions**: Efficient information transfer of the left hippocampus, a brain region central to episodic memory, was a risk factor for acute postsurgical pain. Future research should investigate the role of memory and emotion as possible factors in this relationship.

**The role of patient characteristics on invalidation in chronic pain**

Madison Eagle (B.A. Student), Nina Gregoire (Ph.D./LL.B Student), Kimberley Kaseweter (Ph.D.), Paul G. Davies (Ph.D)

University of British Columbia Okanagan

**Introduction/Aim**: Although highly prevalent amongst Canadians, chronic pain (CP) remains a highly stigmatized condition. Perceptions of those with CP can be affected by unrelated external factors; therefore, elucidating these factors is crucial, as discriminatory treatment has been shown to worsen pain symptoms. The present study examined how manipulating a patient’s weight, history of depression, and origin of pain in a vignette would influence third-party illness invalidation.

**Methodology**: Undergraduate students (*N* = 576) recruited from the University of British Columbia Okanagan engaged in a 2 (“obese” BMI vs. “healthy” BMI) × 2 (history of depression vs. no history of depression) × 2 (specific vs. nonspecific origin of pain) between-subjects vignette design. Following the exposure to one of eight vignettes, participants completed an online survey, which included an altered version of the Illness Invalidation Inventory.

**Results**: A 3-way factorial ANOVA found a marginally significant main effect for weight, *F*(1, 568) = 3.30, *p* = .07, η_p_^1^ = .006, and origin of pain, *F*(1, 568) = 3.79, *p* = .052, η_p_^1^ = .007. There was no main effect for history of depression, *F*(1, 568) = 0.004, *p* = .95, η_p_^1^ < .001, nor were there any significant interactions, all *p*-values > .05.

**Discussion/Conclusions**: These findings suggest that those with CP who live with obesity or nonspecific pain sources are more likely to incur invalidation. Conversely, depression history did not influence invalidation. This study emphasizes the systematic issue of discriminatory behavior toward persons with CP by showcasing biases existent among the lay public.

**Portrait de la pratique des ergothérapeutes du Québec en gestion de la douleur chronique**

Émilie Lagueux^a^, Julie Masse^b^ and Yannick Tousignant-Laflamme^a^

^a^Université de Sherbrooke; ^b^Université de Montréal

**Introduction/Objectif**: L’état actuel des connaissances concernant la contribution de l’ergothérapie en gestion de la douleur chronique (DC) a évolué dans la dernière décennie, mais cela a-t-il été transféré à la pratique clinique? Le but de l’étude était de décrire l’état actuel de la pratique ergothérapique en gestion de la DC au Québec (Canada).

**Méthodologie**: Un sondage en ligne a été envoyé aux ergothérapeutes membres de l’Ordre des ergothérapeutes du Québec.

**Résultats**: Des 90 répondants, 42,2% travaillaient principalement en soins primaires et 52,2% en soins secondaires. Leur rôle principal visait à promouvoir l’occupation et la réadaptation au travail. Le Modèle canadien du rendement et de l’engagement occupationnels (87,8%), l’entrevue semi-structurée (86,7%) et l’éducation sur la conservation d’énergie (65,6%) et l’hygiène posturale (60,0%) étaient les plus fréquemment cités.

**Discussion/Conclusion**: Les résultats illustrent la diversité de la pratique ergothérapique actuelle et suggèrent des possibilités d’amélioration en mettant l’accent sur une vision de la santé et du bien-être fondée sur l’occupation. Les actions futures doivent s’appuyer sur des preuves scientifiques robustes afin d’améliorer certains aspects du processus clinique, comme l’utilization d’évaluations standardisées et la mise en œuvre d’interventions ciblant directement des ajustements de modes de vie.

**Delivering chronic noncancer pain management in family medicine: A nurse-led program**

Metasebia Assefa^a^, Isabelle LeClerc^b^, Elizabeth Muggah^b^, Raywat Deonandan^c^, Charles Godbout^d^ and Hillel Finestone^b^

^a^Children’s Aid Society; ^b^Bruyère Continuing Care; ^c^University of Ottawa; ^d^Bruyère Research Institute

**Introduction/Aim**: The gold standard for the management of chronic noncancer pain is multidisciplinary care. Medical, physical, psychological and social factors are incorporated into a treatment plan that enables the patient to better manage their chronic painful conditions. Too often, opioids and other physical modalities, e.g., injections, are offered as first-line therapies, frequently hospital-based and not integrated into primary care. This poster describes an effective chronic noncancer pain program in primary care, led by a registered nurse (RN) who counsels within a family practice.

**Methodology**: Patients 18 years of age or older with chronic noncancer pain (*n* =111) were referred by family physicians or nurse practitioners within the family health team (outpatient, multidisciplinary clinic) in Ottawa, Ontario.

**Results**: Arthritis, back and neck pain, and fibromyalgia were frequent diagnoses. The RN used the Pain Explanation and Treatment Diagram (developed by a physiatrist, Dr. Hillel Finestone) with patients, taught self-management skills (related to habits [smoking, consumption of alcohol, diet], exercise, sleep, ergonomics, and psycho-social factors). The physiatrist was available for consultation. The RN referred patients to relevant resources in the community. Outcomes related to pain intensity, pain interference with daily living, and opioid use were improved (published in *Canadian Family Physician*, March 2023).

**Discussion/Conclusions**: The program, initiated without extra funding, was successfully integrated into a primary care setting. This program can serve as a model for improving chronic noncancer pain management in primary care and society.

**Overwhelming experiences of pain: A phenomenological study of the disrupted self**

Mael Gagnon-Mailhot^a^, Peter Stilwell^b^, Anne Hudon^c^, Virginia McIntrye^d^, Keith Meldrum^e^, M. Gabrielle Pagé^a^ and Timothy Wideman^b^

^a^Department of Psychology, Université de Montréal, Montreal, Quebec, Canada; ^b^School of Physical and Occupational Therapy, McGill University, Montreal, Quebec, Canada; ^c^School of Rehabilitation, Université de Montréal, Montreal, Quebec, Canada; ^d^Coldbrook, Nova Scotia, Canada, ^e^Kelowna, British Columbia, Canada

**Introduction/Aim**: Disruption to one’s sense of self is believed to be an integral part of pain-related suffering. Past research has established the self as having two core interrelated aspects: *narrative-self* and *minimal-self*. Narrative-self involves self-reflection and consists of the self-identity we cultivate over time. Minimal-self is in-the-moment experiences that shape one’s stream of consciousness and involves a sense of agency and ownership over one’s experiences. Although past suffering research has focused on narrative-self disruptions, this study examined minimal-self disruptions in overwhelming pain experiences.

**Methodology**: We conducted a theoretically driven phenomenological interview study with 12 adults reporting pain that significantly disrupted their lives. Transcripts were analyzed using a hybrid (deductive-inductive) approach to coding and theme development.

**Results**: Disruptions to the minimal-self involved loss of agency over actions, thoughts, sensations, and emotions. Participants reported feeling controlled by pain, experiencing dehumanization, powerlessness, hopelessness, helplessness, and a strong urge to escape. Time distortions and altered bodily ownership**/**awareness were also described, including out-of-body experiences. Contextual factors like isolation, stigmatization, and pain novelty/uncertainty were often related to these experiences and highlighted interface with the narrative-self. The extent to which people experienced disruptions to their minimal and narrative selves fluctuated over time.

**Discussion/Conclusions**: This study is the first to characterize pain-related disruptions to the minimal-self, contributing to a more comprehensive model of pain-related suffering. Pain-related suffering can take many forms, as different aspects of the self interact and are threatened, lost, or altered. Our findings have important implications for developing more individualized pain management strategies that target different aspects of suffering.

**Effectiveness of Ketamine for management of chronic noncancer pain: a systematic review and meta-analysis of randomized controlled trials**

Sara Moradi^a^, Mojdeh Daneshmand^b^, Ahmad Sofi Mahmudi^a^, Geoff Elder^a^, Jason W. Busse^a^ and Behnam Sadeghirad^a^

^a^McMaster University; ^b^Shahid Beheshti University

**Introduction/Aim**: Chronic pain is a prevalent health condition, imposing a significant economic burden on healthcare systems. Ketamine shows promise in managing chronic pain. We conducted a systematic review and meta-analysis of randomized trials to evaluate Ketamine’s benefits and harms in adults with chronic noncancer pain (CNCP).

**Methodology**: We searched MEDLINE, Embase, CINAHL, and Cochrane CENTRAL up to July 2023 for trials that: (1) enrolled adults (≥ 16 years old) diagnosed with CNCP, and (2) randomized to ketamine, placebo/no-treatment. We performed a random-effects meta-analysis for patient-important outcomes at immediate [1-to-3 hours], short-term [3–7 days], and medium-term [3–5 weeks] follow-up and assessed certainty of evidence using GRADE.

**Results**: We identified 1890 records and found 35 eligible randomized trials [1805 patients]. Results from meta-analysis of 11 trials showed that Ketamine reduced pain at immediate follow-up by 1.48 points on a 10-point Visual Analogue Scale (VAS) (95%CI: −2.12 to −0.84; I^1^ = 0.0%, moderate certainty) compared to Placebo. A similar reduction was observed at the short-term follow-up (9 trials; MD −1.46 [on a 10-point VAS], 95%CI: −1.90 to −1.01; I^1^ = 41.6%, low certainty). Meta-analysis from six trials showed a 1.22 points reduction of pain on a 10-point VAS (95%CI: −1.22 to −1.77; I^1^ = 37.7%%, low certainty) at medium-term follow-up. Sensitivity analysis excluding studies with co-intervention yielded similar results for all time-points favoring Ketamine over placebo.

**Discussion/Conclusions**: Ketamine is likely to reduce pain in patients with CNCP at immediate follow-up time and may reduce pain in patients with CNCP at short- and medium-term follow-up time.

**Co-occurrence of anxiety and depressive symptoms among persons with chronic pain: Insights from a symptom network approach**

Leon Tourian^a,b^, Gabriella Spiegler^c^, Yilin Zhang^d^, Louis-Phillipe Langlois^e^, Nesrine Mesli^e^, Masha Verner^a^, Sabrina Mitrovic^a^, Mark Ware^a, f^, M. Gabrielle Pagé^a, g, h^ and Marc Martel^a, i, j^

^a^Alan Edwards Pain Management Unit, McGill University Health Center; ^b^Department of Psychiatry, McGill University; ^c^Department of Epidemiology, Biostatistics, and Occupational Health, McGill University; ^d^Faculty of Medicine and Health Sciences, McGill University; ^e^Department of Psychology, McGill University; ^f^Department of Family Medicine, McGill University; ^g^Research Center of the Center Hospitalier de l’Université de Montréal; ^h^Department of Anesthesiology and Pain Medicine, Université de Montréal; ^i^Faculty of Dental Medicine and Oral Health Sciences, McGill University, ^j^Department of Anesthesiology, McGill University

**Introduction/Aim**: Chronic pain can exert a deleterious impact on mental health. Anxiety and depressive disorders are particularly prevalent among persons with chronic pain, and can be accompanied by a host of neurovegetative, cognitive, and affective symptoms. However, questions remain concerning the symptoms that play the most predominant (i.e., central) role in the anxiety and depressive symptomatology of persons with chronic pain. The first objective of this study was to explore the relative centrality (i.e., importance) of specific anxiety and depressive symptoms in persons with chronic pain. We also explored the co-occurrence and interrelations among these symptoms.

**Methodology**: This study included 721 persons with chronic pain from a tertiary pain management unit who were referred for a mental health assessment. Structured clinical interviews were conducted based on the Diagnostic and Statistical Manual of Mental Disorders (DSM-5) to assess the presence/absence of symptoms associated with Generalized Anxiety Disorder (GAD) and Major Depressive Disorder (MDD).

**Results**: Network analyses using the *Bootnet* package in R revealed that excessive worry, lack of control over worry, and restlessness were the most central symptoms of the network, followed by a cluster of neurovegetative symptoms (i.e., sleep problems, lack of energy, and poor concentration). The strongest interrelations involved worry, lack of control, and restlessness (std. edge weights: 1.69–3.78) as well as sleep problems and anhedonia (EW = .94).

**Discussion/Conclusions**: Our findings provide insights into the specific cognitive-affective and neurovegetative symptoms that most often co-occur and play a predominant role in the anxiety and depressive symptomatology of persons with chronic pain.

**Pain management services among persons with high-impact chronic pain and comorbid mental health problems**

Sabrina Mitrovic^a^, Nesrine Mesli^b^, Louis-Phillipe Langlois^b^, Maria Verner^a^, Amanda Sirois^a, c^, Regina Visca^d^, Mark A. Ware^a, d^, Marc O. Martel^a,c, e^ and Leon Tourian^a, f^

^a^Alan Edwards Pain Management Unit, McGill University Health Center; ^b^Department of Psychology, McGill University; ^c^Faculty of Dental Medicine and Oral Health Sciences, McGill University; ^d^Department of Family Medicine, McGill University, Montréal, QC, Canada; ^e^Department of Anesthesiology, McGill University, ^f^Department of Psychiatry, McGill University

**Introduction/Aim**: Many patients experience high-impact chronic pain (HICP), which is characterized by severe restrictions in daily life or occupational activities due to pain. For many patients, chronic pain can also be accompanied by mental health problems. There is reason to believe that HICP and mental health could influence pain management services received by patients, but this needs to be further explored. The first objective was to examine the association between HICP and pain management services received by patients with chronic pain. We also examined the association between mental health problems and pain management services.

**Methodology**: This study included 301 patients with chronic pain from a tertiary care setting. Mental health assessments were conducted based on the DSM-5 and included diagnoses of generalized anxiety disorder (GAD) and major depressive disorder (MDD). Self-reports of functional limitations were used to assess HICP. Information on pain management services (i.e., number of clinic visits, nursing services, interventional pain management procedures, and physical therapy visits) was retrieved from electronic health records.

**Results**: Poisson regression analyses indicated that HICP was associated with a significantly greater number of nerve blocks (*p* < .05). HICP was not associated with any other types of services received. MDD was associated with significantly more physical therapy visits (*p* < .001), and GAD was associated with significantly more nursing services as well as infusions (*p*’s < .001).

**Discussion/Conclusions**: Our findings suggest that certain types of pain management services are more frequently offered to patients with HICP and mental health problems.

**Alberta Pain Strategy five years after implementation: Successes and challenges of the opioid use and management stream**

Magali Robert^a^, Tracy Wasyluk^b^, Robert Tanguay^a^, Tracey Geyer^b^, John Pereira^a^, Elena Lopatina^a^, Nivez Rasic^a^ and Susan Sobey-Fawcett^b^

^a^University of Calgary; ^b^Alberta Health Services

**Introduction/Aim**: The Alberta Pain Strategy^2^ (APS) was conceptualized in 2017 as a partnership between the Pain Society of Alberta (PSA), AHS’ Strategic Clinical Networks™ (SCNs), and 360 Albertans to develop the strategy that launched in October 2019.

**Methodology**: Through comprehensive engagement including the Pain Society of Alberta executive, SCNs, people with lived experience (PWLE), patient advocates and other stakeholders identified opioid use in pain management as a focus area. Important topic areas in each stream were identified.

**Results**: The key priority areas and midpoint status update identified in Table 1

Some challenges included:

- the SARS-Cov2 pandemic
- Declining primary care work force
- Worsening of the opioid overdose crisis
- Member attrition
- Successes as of October 2023:
- Agreement in Principle: Alberta’s Care Plan for Chronic Pain Patients on Opioid Therapy
- Rural Opioid Outreach Program
- Opioid crisis support for Indigenous People

Table 1: Progress to April 2022

Addressing Stigma with Special Reference to “Legacy” Opioid Patients

Develop a pathway for treatment for those individuals who have been using opioids long term

Establish peer navigators within the system

Implement *Green Zones* or *Urgent Opioid Pain Clinics* across the province.

Evidence – Informed Options and Current Guidelines

Access to active physical therapy, exercise training and psychological therapy free of cost to those with chronic pain.

Indigenous Populations and Response

Develop an Indigenous-specific pain program.

Create a mobile access team that can provide treatment directly in community.

**Discussion/Conclusions**: The present work accomplished by the other streams of the APS have informed this stream. The changing landscape has shifted some priorities.

**Comparative effectiveness of internet-based psychotherapies for management of chronic noncancer pain: a network meta-analysis of randomized controlled trials**

Behnam Sadeghirad^a^, Shiva Shahabi^a^, Azin Khosravirad^a^, Sara Moradi^a^, Shahrzad Motaghi^a^, Randi McCabe^a^, Brittany Rosenbloom^b^ and Jason Busse^a^

^a^McMaster University; ^b^Toronto Academic Pain Medicine Institute

**Introduction/Aim**: Almost all pharmacotherapies for the management of chronic noncancer pain (CNCP) have small-to-modest efficacy and are associated with important side effects. Remotely delivered psychotherapies are suggested as the less costly alternative or additive interventions. We conducted a systematic review and network meta-analysis of randomized trials to assess the comparative effectiveness of remotely delivered psychotherapies in adults with CNCP.

**Methodology**: We searched MEDLINE, Embase, CINAHL, PsycINFO, and Cochrane CENTRAL up to October 2023 for trials that: (1) enrolled adults (≥ 16 years old) diagnosed with CNCP, and (2) randomized to any psychotherapy intervention delivered remotely from the therapist (e.g., Internet, smartphone application), waitlist or active control, or treatment as usual. We performed a random-effects network meta-analysis using frequentist approach for patient-important outcomes at postintervention time, medium-term follow-up [3–6 months], and long-term follow-up [6–12 months] and assessed certainty of evidence using GRADE.

**Results**: We identified 2,307 records through our electronic searches. We included 35 randomized trials involving 5,126 individuals diagnosed with CNCP. Median of participant average age was 49.1 years with duration of psychotherapies ranging from 3 to 26 weeks (median 8 weeks). Most frequent psychotherapy was internet-based cognitive behavioral therapy [CBT] (18 trials), CBT+treatment as usual (7 trials) followed by acceptance and commitment therapy [ACT] (6 trials). Both CBT and ACT alone or in combination with treatment as usual showed improvement in pain intensity at postintervention and medium-term follow-ups. Psychotherapies may have little to no benefit on physical functioning.

**Discussion/Conclusions**: Internet-based psychotherapies are likely to have small benefit in management of CNCP.

**Alberta Pain Strategy: Successes and challenges of the Acute Pain Stream**

Magali Robert^a^, Tracy Wasyluk^b^, Tracey Geyer^b^, Nivez Rasic^a^, John Pereira^a^, Elena Lopatina^a^, Robert Tanguay^a^ and Susan Sobey-Fawcett^b^

^a^University of Calgary; ^b^Alberta Health Services

**Introduction/Aim**: The Alberta Pain Strategy^1,2^ (APS) was conceptualized in 2017 with the support of the Pain Society of Alberta and Alberta Health Services (AHS) Strategic Clinical Networks™ (SCNs). The strategy was launched in October 2019. Overall, 360 Albertans actively participated.

**Methodology**: The Pain Society of Alberta executive, SCNs in collaboration with people with lived experience (PWLE), patient advocates, and other stakeholders decided on three focus streams: acute pain, chronic pain, and opioid use in pain management. Important topic areas in each stream were identified and each topic area had a committee balanced to include all stakeholders and geographical distribution to ensure representation and diversity as seen within and across the province.

**Results**: The key priority areas are identified in Table 1 with a report card at the midway point (2.5 years). Table 2 lists the accomplishment near completion of the strategy.

Some barriers included:

- adoption of the Alberta Surgical Initiative^1^ mandated by the Alberta Minister of Health
- the SARS-Cov2 pandemic
- a shortage of anesthesiologists in the operating rooms
- attrition of some committee members

**Discussion/Conclusions**: A provincial strategy for acute pain care is possible when all stakeholders are involved. It requires the ability to pivot with changes in the pain landscape. The Alberta Pain Strategy will continue to complete its vision and build on its successes.

**References**

1. Alberta Pain Strategy | Alberta Health Services (Nov 7, 2023).

2. Alberta Surgical Initiative | Alberta Health Services (Nov 7, 2023).

**Understanding the impact of demographic and psychological factors on pain variability in adults with chronic pain: The CircaPain project**

Elisabeth Lamoureux^a^, Hailey Gowdy^b^, Doriana Taccardi^b^, Amanda Zacharias^b^, Mitra Knezic^b^, Lesley Norris Singer^c^, Jennifer Daly-Cyr^c^, Zihang Lu^d^, Manon Choinière^e^, Nader Ghasemlou^b, f, g^ and M. Gabrielle Pagé^a, e^

^a^Department of Psychology, Université de Montréal, Canada; ^b^Department of Biomedical and Molecular Sciences, Queen’s University, Canada; ^c^Chronic Pain Network, Canada; ^d^Department of Public Health Sciences, Queen’s University, Canada; ^e^Department of Anesthesiology and Pain Medicine, Université de Montréal, Canada; ^f^Department of Anesthesiology and Perioperative, Medicine, Queen’s University, Canada; ^g^Centre for Neuroscience Studies, Queen’s University, Canada

**Introduction/Aim**: Chronic pain is a complex experience marked by substantial inter- and intra-individual pain variability. Although chronic pain is commonly associated with symptoms of psychological distress including anxiety and depression, little research has examined the impact of demographic and psychological factors on pain fluctuations among this population.

**Methodology**: Canadian adults living with chronic pain (*n* = 555, age = 57.0 ±13.0 years, 84.5% woman) were recruited via partner advocacy organizations, social media, and clinic outreach to participate in the CircaPain Project. Participants completed a baseline questionnaire measuring pain type and duration, pain intensity, anxiety, and depression, and an electronic diary tracking their pain intensity 3 times per day for 7 days. Multiple linear regression models were used to test if age, gender, anxiety, depression, and pain catastrophizing significantly predicted pain variability (SD of pain scores) among participants who had completed at least 80% of the diary entries.

**Results**: Among the examined variables, anxiety was the only significant variable negatively associated with pain variability (B = −0.03, *p* = .01). Sensitivity analyses showed that results remained unchanged if only those with 50% of diary completion were considered.

**Discussion/Conclusions**: The current findings provide evidence that individuals who report fewer anxiety symptoms experience greater pain variability (for example, pain that follows a circadian rhythm) than individuals whose pain remains more constant over time. This is in line with previous reports showing that those with a circadian pain rhythm report less depression and opioid consumption than those who report stable pain intensity. Further research is needed to examine the temporality of this relationship.

**The Alberta Pain Strategy: Chronic Pain Stream report card**

Magali Robert^a^, Tracy Wasyluk^b^, Tracey Geyer^b^, Robert Tanguay^a^, Lori Montgomerty^a^, John Pereira^a^, Elena Lopatina^a^, Nivez Rasic^a^ and Susan Sobey-Fawcett^b^

^a^University of Calgary; ^b^Alberta Health Services

**Introduction/Aim**: The Alberta Pain Strategy^1^ (APS) conceptualized in 2017 as a partnership between the Pain Society of Alberta (PSA), AHS’ Strategic Clinical Networks™ (SCNs), and 360 Albertans to develop the strategy that launched in October 2019.

**Methodology**: Through comprehensive engagement, including people with lived experience (PWLE), chronic pain was identified as an area of focus.

**Results**: The key priority areas and mid-point status are identified in Table 1.

Identified barriers/hurdles:

- the SARS-Cov2 pandemic
- declining primary care work force
- Member attrition
- launch of a Provincial EMR, shifting workforce and focus

Priority Access

Develop and disseminate a catalog of pain resources. Create a sustainable model for funded access to evidence-informed interdisciplinary service. Patient, Provider, and Public Education. Establish pain competencies and curricula. Establish a provincial repository of pain assessment tools, treatment guidelines, and other useful resources. Develop a chronic pain public education campaign.

Performance Outcomes

Identify common provincial outcome measures. Provincial Approaches Develop and implement an interdisciplinary hub and spoke. Develop a provider mentorship model for chronic pain and addictions.

Create navigation pathways for use by Health Link. Connect Care (Epic) infrastructure and web-based platforms making chronic pain tools and pathways available

**Discussion/Conclusions**: Strong leadership and committed stakeholders responded nimbly, adjusting priorities to reorganize in the face of change resulting in grant funding to launch an Alberta Virtual Pain Program.

**References**

1. Alberta Pain Strategy | alberta Health Services (Nov 7, 2023).

**Toward a computational understanding of chronic pain**

Mégane Lacombe-Thibault^a, b^ and Michel-Pierre Coll^a, b^

^a^Université Laval; ^b^Centre interdisciplinaire de recherche en réadaptation et intégration sociale (CIRRIS)

**Introduction/Aim**: According to a learning account of pain, pain should be modulated to optimize learning. The aim of this study was to test this hypothesis by assessing the influence of the informational value of pain on the subjective perception of pain in healthy participants. We expected that the participants’ pain perception would be greater when pain was surprising and the environment uncertain.

**Methodology**: Fifty healthy participants (28 F, 22 M; mean age of 24,38 years old) took part in an experimental task in which they had to learn to predict the associations between high or low tones and high or low painful electric stimuli. At each trial, a tone was presented and was followed by a painful stimulus. Participants had to indicate as quickly as possible if the pain was high or low. The probabilities related to the tone-pain association changed throughout the task and remained stable for several trials or changed quickly to create periods of low and high environmental uncertainty.

**Results**: Model-free analyses confirmed that participants took longer to answer when pain was unexpected and that expectations had a significant effect on subjective pain perception. Model-based analyses using a hierarchical bayesian learning model also suggested that trialwise computational estimates of expectations and uncertainty estimates were significantly related to pain perception.

**Discussion/Conclusions**: This research reinforces the established link between pain and learning, demonstrating that pain is adaptively adjusted to enhance learning efficiency. These findings offer a basis for exploring how learning about pain could be altered in the context of pathological pain conditions.

**The most effective treatment according to individuals living with chronic pain in a real-life setting**

Meriem Zerriouh, Claudie Audet, Gwenaelle De Clifford-Faugere and Anaïs Lacasse

Département des sciences de la santé, Université du Québec en Abitibi-Témiscamingue (UQAT), Rouyn-Noranda, Québec, Canada

**Introduction/Aim**: Chronic pain is a complex condition that necessitates the integration of various treatments, including pharmacological, physical, or psychological approaches. Although evidence regarding the efficacy of individual treatments is often available through clinical trial data, the question arises as to which treatments are perceived as the most effective in the real-world. This study aimed to gather patients’ perceptions of the most effective treatments among those currently used to manage chronic pain.

**Methodology**: This exploratory cross-sectional web-based study analyzed data from 1,263 individuals living with pain for more than 3 months. Participants were recruited from the community (Quebec). After responding to various questions about medications, as well as physical and psychological treatments currently used, participants were asked an open-ended question about which treatment they considered the most effective. Responses were carefully reviewed to develop a standardized coding system.

**Results**: 36.6% identified nonpharmacological treatments as the most effective, compared to 28.8% medications obtained from community pharmacies, and 18.7% a combination of pharmacological and nonpharmacological treatments. Additionally, 2.8% reported cannabis, 2.2% clinic-based injections, 2.2% reported not knowing, and 8.6% stated that no treatment is effective (mutually exclusive categories). When examining specific nonpharmacological approaches, responses varied widely, and no single approach emerged as highly prevalent in terms of perception. The two most frequently mentioned were exercise, reported as the most effective by 10.0% of participants, and massage therapy, reported by 8.4% (nonmutually exclusive categories).

**Discussion/Conclusions**: Our results emphasize that one size does not fit all in terms of perceptions regarding the effectiveness of chronic pain treatments.

**Identifying subtypes and pain trajectories in chronic overlapping pain conditions**

Christophe Tanguay-Sabourin^a, b, c^, Azin Zare^b, d^, Pierre Rainville^b, c, e^ and Etienne Vachon-Presseau^b, d, f^

^a^Faculty of Medicine – University of Montreal; ^b^AECRP – McGill University; ^c^CRIGUM – University of Montreal; ^d^Faculty of Dental Medicine and Oral Health Sciences – McGill University; ^e^Faculty of Dental Medicine – University of Montreal; ^f^Anesthesia Research Unit – McGill University

**Introduction/Aim**: Chronic pain often coexists with other forms of pain and comorbid conditions, referred to as Chronic Overlapping Pain Conditions (COPCs). These conditions are challenging to study due to their diverse etiologies and clinical presentations. This study aimed to identify homogeneous subtypes of COPCs and understand their distinct pain spread trajectories using unsupervised machine learning.

**Methodology**: We analyzed data from 81,600 individuals with chronic pain who completed the UK Biobank online pain questionnaire including pain ratings across 12 distinct body sites. To characterize pain spread, we used the unsupervised machine learning algorithm merging disease progression with clustering to obtain probabilistic spatiotemporal partitioning. Subtypes were then compared based on their pain spread trajectories, diagnoses, and nonpain symptomatology.

**Results**: We identified four different subtypes (S1-4) associated with distinct putative trajectories of pain spread. These trajectories were characterized based on the spread (Number of Pain Sites, *R^2^ *= 37–38%) and intensity (Worst Pain Rating; *R^2^ *= 32–40%) of pain but could also capture the impact of pain (Brief Pain Interference; *R^2^ *= 25–34%). These subtypes exhibited varying predominant body sites, combinations of pain diagnoses (nociceptive, neuropathic, and nociplastic), and multisystem symptomatology (e.g., cardiological, respiratory, GI among others). Importantly, across all subtypes, the trajectories of pain spread were associated with greater signs of neuropathic symptoms localized at their most bothersome pain site (DN4; *r*= 0.23–0.40).

**Discussion/Conclusions**: In conclusion, our data-driven model shows distinct patterns of pain spread and accompanying symptomatology within COPCs. Understanding these trajectories holds the potential to inform the etiology of chronic pain and provide insights into effective pain management strategies.

**Treated versus self-reported prevalence of chronic pain and costs of patients’ health services utilization: A population-based study of health administrative databases**

Magali Robert^a^, Nguyen Xuan Thanh^b^, Elena Lopatina^a^, Lori Montgomery^a^, Robert Tanguay^a^ and Tracy Wasyluk^b^

^a^University of Calgary; ^b^Alberta Health Services

**Introduction/Aim**: Understanding how patients with chronic pain access health care services is important for policy decisions, planning and addressing needs.

**Methodology**: Physician billing codes were used to identify individuals with chronic pain treated during the year 2021/22. This prevalence estimate was compared to self reported prevalence estimates.^1,2^ Alberta Health Administrative databases were linked to identify health service utilization and to estimate costs. Services accessed included: visits to family practitioners, specialists, inpatient, emergency department, outpatient clinic services, and prescription drugs.

**Results**: The treated prevalence of patients with chronic pain was 6.0% representing only 30–41% of the self-reported prevalence. The average cost per patient per year was $5096; most of the which was related to inpatient hospitalizations. Sex differences were observed. The total cost of care of chronic pain patients for the health system in Alberta was $1.37 billion (7% of total health expenditure).

**Discussion/Conclusions**: Many individuals with chronic pain do not access public health care. However, the economic burden is substantial.

**References**

1. Thanh N, Lopatina E, Montgomery L, Robert M, Tanguay R, Wasylak T. Treated versus Self-reported Prevalence of Chronic Pain and Costs of Patients. Health Serv Util. 2023; Nov 7, 2023. doi:10.1177/20494637231209928.

2. Thanh NX, Tanguay RL, Manhas KJP, Kania-Richmond A, Kashuba S. Economic burden of chronic pain in Alberta, Canada. PLOS ONE. 2022;17(8):e0272638. doi:10.1371/journal.pone.0272638.

**Scoping review of virtual vulvar pain and chronic pelvic pain programs**

Magali Robert, Maryam Nasr-Esfahani and Caitlin Jago

University of Calgary

**Introduction/Aim**: Pelvic pain affects 27% of the female population. Virtual health options have increased access to services which could help address prolonged wait lists and meet the needs population sectors.

**Methodology**: The PRISMA with extension for scoping reviews approach was used. Medline, Embase and CINHal databases were searched up to July 13, 2023, using multiple search terms and incorporating virtual health terms. Titles and abstracts were independently searched by two researchers. Using an overinclusive approach, all possible papers were reviewed for further inclusion. Data extraction was individualized by paper due to the paucity of publications.

**Results**: After removal of duplicates, 795 citations were screened for chronic pelvic pain. Of those, 36 papers were assessed. Only two papers met criteria for inclusion. One randomized controlled trial of 8 week modules on an eHEALTH platform for women who had penetrative pain with intercourse compared to controls. This showed initial improvement but no statistical differences between groups at 6 months. The other small pilot study evaluated women with pain related to endometriosis who attended one virtual supervised group exercise session compared to a single virtual reality session and control group. Both modalities showed favorable outcomes over controls but not themselves.

A similar search to identify virtual vulvar pain programs identified 31 citations, non of which met criteria

**Discussion/Conclusions**: There is a dearth of publications on the use of virtual care for vulvar and pelvic pain programs. There is a need to address this vacuum in care.

**Impact of chronic pain amongst medical students in central and western Canada**

Alexandra O. MacNeil^a^ and Karim Mukhida^a, b^

^a^Faculty of Medicine, Dalhousie University; ^b^Department of Anesthesia, Pain Management and Perioperative Medicine, Dalhousie University

**Introduction/Aim**: University students with chronic pain are more likely to experience academic challenges and worse quality of life compared to their peers without chronic pain. To date, there have been no studies focusing on the chronic pain experiences of medical students in Western and Central Canada. Therefore, the purpose of our study was to address this knowledge gap by looking at the prevalence of chronic pain in this population, students’ perceived impact of chronic pain on their lives, and their attitudes surrounding disclosure of their pain to their institutions.

**Methodology**: An online, anonymous survey was developed. Information was collected regarding students’ quality of and impact of their pain. The study was distributed via administration, student societies and/or social media to medical students at seven different schools in Central and Western Canada, and by the Canadian Federation of Medical Students via newsletter.

**Results**: There were 193 study participants, 80 participants indicated that they had chronic pain. Of those, 67.5% identified as women. Participants indicated that pain most impacted their overall quality of life (21.2%), mood (18.8%), and daily activities (16.3%). Only 20% of affected participants had disclosed having chronic pain to their institution.

**Discussion/Conclusions**: These findings suggest that chronic pain impacts medical students’ quality of life and that their institutions are largely not aware of this. Further research is required to better understand the barriers that medical students face or perceive to disclosing their chronic pain, as well as potential supports for them.

**Patient reported wait times to multidisciplinary pain clinics in Ontario, Quebec, and Manitoba and the impact of living with chronic pain on their quality of life**

Clare Liddy^a^, Jennifer Anthonypillai^b^, Geoff Bellingham^b^, Norman Buckley^c^, Lynn Cooper^d^, Tracy Deyell^e^, Rola Hashem^f^, Pablo Ingelmo^g^, Gabrielle Logan^h^, Tess McCutcheon^i^, Melissa Milc^j^, Isabella Moroz^d^, Patricia Poulin^j^, Zahra Sepehri^h^, Alexander Singer^h^, Marie Vigouroux^g^, Regina Visca^f^ and Amin Zahrai^a^

^a^University of Ottawa; ^b^McMaster University; ^c^Western University; ^d^Chronic Pain Network; ^e^Bruyere Research Institute; ^f^Children’s Hospital of Eastern Ontario Research Institute; ^g^McGill University; ^h^University of Manitoba; ^i^University of Toronto; ^j^Ottawa Hospital Research Institute

**Introduction/Aim**: Wait times at Canadian multidisciplinary pain clinics often exceed benchmark recommendations. The goal of this study was to gain insight into the patient experience of waiting for chronic pain specialty care.

**Methodology**: A cross-sectional survey of new patients waiting to attend a chronic pain appointment was conducted in 6 multidisciplinary pain clinics in Ontario, Quebec, and Manitoba between February 2020 and October 2022. One of the clinics was pediatric. Participants were asked about the length of time they waited for their appointment since being referred, their quality-of-life, healthcare professionals seen while waiting, and an open-ended question “Is there anything else you’d like to tell us?”

**Results**: Among the 484 adult and 99 pediatric respondents, 53% of adults and 82% of children reported wait times under 6 months, whereas 22% of adults and 4% of children waited longer than a year. Between 52–63% of adults and 29–48% of children reported being affected by chronic pain “quite a bit” or “extremely” on measures of quality of life. The most visited healthcare professionals while waiting for a pain clinic appointment were family doctors/nurse practitioners for adults and physiotherapists for children. Qualitative analysis of open-ended question responses revealed 8 themes: system navigation issues, administrative issues, decreased quality of life, distress, self-advocacy, coping strategies, communication, and distrust.

**Discussion/Conclusions**: Our findings provide real-time regional snapshots into the impact of long wait times experienced by Canadians living with chronic pain. There is an urgent need to better support patients during the waiting period. Expanding technologies such as electronic consultation hold great promise.

**Expression and localization of 5-HT receptors in the dorsal horn of rat and human spinal cords**

Clare Murray-Lawson^a, b^, Laurence David^a^, Gordia Fathi^a^, Newton Martin^a^, Katherine Griffiths^a, b^, Jessica Parnell^a, b^, Santina Temi^a^, Annemarie Dedek^a, b^, Eve Tsai^b^ and Michael Hildebrand^a, b^

^a^Carleton University; ^b^The Ottawa Hospital Research Institute Department of Neuroscience

**Introduction/Aim**: Serotonin, also known as 5-HT, is a promising potential target system for treating chronic pain. Although rodent studies and preclinical investigations on psychedelics, as well as 5-HT receptor agonists and antagonists, suggest a connection between serotonergic signaling and spinal pain processing, the specific mechanisms underlying this relationship remain largely unknown. The superficial laminae of the dorsal horn are pivotal in nociception; however, the distribution of 5-HT receptors in the spinal cord remains unexplored in human samples, with no consideration given to potential sex differences.

**Methodology**: This study employs a multidimensional approach to map the distribution of 5-HT receptors in rat and human spinal pain circuits. Through immunohistochemistry, qRT-PCR, and single-cell/nuclei RNA sequencing, we systematically investigated the spinal expression of all 5-HT receptor subtypes as well as localization of 5-HT2C in dorsal horn circuits, with analyses across spinal cord region, sex, and species.

**Results**: Our findings suggest that the 5-HT2C receptor is highly expressed and preferentially localized to the superficial laminae of the dorsal horn in both rat and human spinal cord and other serotonin receptor subtypes are expressed densely within the dorsal horn. Furthermore, we are investigating potential sex differences and variations in receptor expression and localization across thoracic and lumbar spinal cord sections.

**Discussion/Conclusions**: The 5-HT2C receptor, along with other 5-HT receptor subtypes, exhibit a dense expression in the superficial laminae of the dorsal horn in both rat and human spinal cords. This pattern suggests an involvement of spinal serotonin transmission in the modulation of pain.

**Conversion rations between conventional opioids and methadone in pediatrics: A 13 year retrospective review**

Elise Druon, Marie-Joëlle Doré-Bergeron, Kathryn DeKoven, Catherine Corriveau, Tanya Santella, Edith Villeneuve, Jean-François Delisle, and Flaviu Adrian Mosora

CHU Sainte-Justine

**Introduction/Aim**: Methadone has multiple benefits in pediatrics due to its unique pharmacokinetic profile, multiple administration forms and pharmacodynamic profile which makes it active against nociceptive and neuropathic pain. No established conversion ratios for switching between conventional opioids and methadone exist in pediatrics. The aim of this retrospective study is to present the conversion ratios used in a large sample of pediatric patients at baseline and at methadone steady-state.

**Methodology**: This study includes a heterogenous patient population ages 3 months to 18 years treated with methadone at the Center Hospitalier Universitaire Sainte-Justine from January 1^st^ 2011 to March 31^st^ 2023. Patients treated for substance abuse and neonates were excluded.

**Results**: 65 patients, aged 10,6 years, were identified, the majority of which were treated for neuropathic or mixed type pain. Treatment duration was 184 days on average (median 79 days). Methadone doses ranged from 0,1 to 300 mg/day (0,01 to 10,72 mg/kg/day). Initial conversion ratios from oral morphine equivalent doses to methadone ranged from 0,64 to 162:1. No standardized conversion ratios could be derived from the collected data when age, weight, or conventional opioid doses were categorized. Methadone doses were kept stable or increased in 60/65 patients at steady-state.

**Discussion/Conclusions**: This review reflects the difficulty of establishing pediatric standardized conversion ratios similar to the ones available for the adult population. Data scarcity and heterogeneity, as well as covariables affecting the initial conversion ratio underline the importance of establishing a pediatric guide for methadone prescription by experienced centers.

**Characteristics of interprofessional rehabilitation programs for patients with chronic low back pain evaluated in the literature: A scoping review**

Sintayehu Daba Wami^a^, Mulugeta Bayisa Chala^b^, Sara Yirgalem Wolde^c^, Catherine Donnelly^a^, Kasahun Aelmu Gelaye^c^, Abdul Pullatayil^a^, Esayas Adefris^c^, and Jordan Miller^a^

^a^Queen’s University, ^b^Western University, ^c^University of Gondar

**Introduction/Aim**: Interprofessional rehabilitation programs are effective at improving pain, health-related quality of life, function, and work abilities for patients with chronic low back pain (CLBP). This scoping review aimed to describe the characteristics of interprofessional rehabilitation programs for patients with CLBP evaluated in the literature to date.

**Methodology**: Our scoping review was guided by the framework developed by Arksey and O’Malley, further enhanced by Levac et al. and the Joanna Briggs Institute (JBI). Electronic databases, including Ovid MEDLINE, EMBASE, CINAHL, PsycINFO, SCOPUS, and Web of Science, were searched to identify all studies that assessed interprofessional rehabilitation programs for patients with CLPB.

**Results**: Out of 13370 articles identified, 79 studies fulfilled our eligibility criteria. In the majority of the studies (*n* = 75), interprofessional rehabilitation programs consisted of two or more of the following interventions: physical activity and exercise (*n* = 68), education (*n* = 61), psychotherapy (*n* = 52), and vocational support/advice (*n* = 31). The commonly represented professionals in the delivery of the interprofessional treatment included physiotherapists (*n* = 73), physicians (*n* = 62), psychologists (*n* = 48), and occupational therapists (*n* = 35). Descriptions of interprofessional rehabilitation programs, components/content of the programs, health professionals involved, settings, duration, frequency and intensity of the interventions, and health outcomes assessed varied widely across studies.

**Discussion/Conclusions**: This scoping review provides a comprehensive overview of how interprofessional rehabilitation programs for CLBP have been conceptualized in the existing literature. Although it is unclear what components of the interventions are most effective and which healthcare professionals should be involved, the results of this scoping review will guide the development of interprofessional rehabilitation programs in new settings.

**Microglial activation is regulated by circadian rhythms in chronic neuropathic pain**

Ciara O’Connor, Olivia Smith, and Nader Ghasemlou

Department of Biomedical and Molecular Sciences, Queen’s University, Kingston, Ontario Canada

**Introduction/Aim**: Microglia have been shown to be drivers of pain hypersensitivity in the spared nerve injury (SNI) model of neuropathic pain in mice. Following SNI, microglia in the dorsal horn of the spinal cord proliferate and transition from a homeostatic phenotype to a pro-inflammatory phenotype. These cellular changes are accompanied by behavioral changes as mice develop mechanical and thermal (cold) allodynia. Clinical studies have shown that pain can be rhythmic in various chronic pain states. However, it remains unknown whether microglia exhibit circadian rhythmicity in chronic pain states.

**Methodology**: To investigate this gap in knowledge, male and female C57BL/6 mice received spared nerve injury, with tissues collected at 3, 7, 10, 14, 28, and 84 days following injury. Animals were sacrificed at ZT2 and ZT14, corresponding to 2 hours after the start of the light- or dark- phases. The spinal cord was stained for markers of homeostatic and pro-inflammatory microglia, and images were taken to create 3D renderings of the cells.

**Results**: We found changes in both microglial morphology and activation state (using markers Iba1, P2RY12, CD68) in both the naïve and injured state. During peak periods of microglial activation following SNI, which occur between 7 and 14 days following injury, microglia in the dorsal horn took on a more pro-inflammatory phenotype at ZT2, and a more homeostatic phenotype at ZT14.

**Discussion/Conclusions**: Further understanding of microglial activation states across male and female mice, and during the circadian cycle, may provide new insight into mechanisms regulating their activity and function in the pathophysiology of chronic neuropathic pain.

**Achieving consensus on the characteristics of an interprofessional rehabilitation program for patients with chronic low back pain tailored to the Ethiopian context: A modified Delphi study**

Sintayehu Wami^a^, Jordan Miller^a^, Catherine Donnelly^a^, Kassahun Alemu Gelaye^b^ and Esayas Adefris^b^

^a^Queen’s University; ^b^University of Gondar

**Introduction/Aim**: Practice guidelines recommend interprofessional rehabilitation programs for people with chronic low back pain (CLBP). However, interprofessional rehabilitation programs vary greatly in the literature, making it challenging to implement the evidence in new settings. The aim of this study was to reach a consensus on the characteristics of an interprofessional rehabilitation program for people with CLBP in Ethiopia.

**Methodology**: A two-round modified Delphi was used to reach a consensus amongst experts in CLBP, interprofessional rehabilitation, and the Ethiopian health service delivery. Findings from a scoping review were used to develop the round one survey question. Participants rated each item on a 5-point Likert scale (strongly disagree to strongly agree) and provided comments. Consensus was defined as > 70% of panel members agreeing/strongly agreeing or disagreeing/strongly disagreeing. Items that did not reach consensus were included (with comments) in a second round.

**Results**: Of the 30 experts invited to participate, 24 and 20 completed the first and second-round Delphi survey, respectively. Participants reached a consensus on 86/95 items (90.5%) in the first-round and 9/17 items (52.9%) in the second-round. The remaining eight items were discussed amongst an expert panel and not included in plans for the program. Consensus was reached related to setting, delivery mode, duration, intervention components, and theoretical foundations.

**Discussion/Conclusions**: The findings from this Delphi study form the foundation for the development of an interprofessional rehabilitation program for patients with CLBP tailored to the Ethiopian context. The methodology used may provide a valuable roadmap for the development of interprofessional rehabilitation programs tailored to other contexts.

**Randomized controlled trial investigating the effectiveness of the Curable Inc mobile application for the treatment of chronic pain**

Cynthia Thomson^a^, Hanna Pahl^a^ and Luisa Giles^a^

^a^University of the Fraser Vallely

**Introduction/Aim**: Treatments for persistent pain commonly rely on physical interventions. Digital programs focusing on the psychosocial aspects of pain may provide low-barrier alternatives. Through a randomized controlled trial, we investigated the effectiveness of a mind-body mobile application (Curable Inc.) on pain outcomes.

**Methodology**: Participants (*n* = 198; 82% women, mean age = 46.7 (13.1) years; mean pain duration = 13.6 (11.2) years) with nonmalignant persistent pain were randomized to either a 6-week intervention (*n* = 98) or wait-listed usual care (*n* = 100). The intervention involved regular engagement with a user-guided mobile application informed by the biopsychosocial model of pain that included pain education, mindfulness training, cognitive behavioral therapy, and expressive writing. The coprimary outcomes were the Brief Pain Inventory (BPI) intensity and interference at 6 weeks, analyzed using mixed model repeated measures (intention to treat analysis).

**Results**: We observed significant reductions in pain severity (−0.67; 95% CI: −1.04 to −0.29) and interference (−0.60 (95% CI: −1.18 to −0.03) in the intervention group compared to the control group following the intervention (BPI intensity: *F*(1, 176) = 12.45, *p* = .001; BPI interference: *F*(1, 181) = 4.35, *p* = .038). There were significant improvements in secondary outcomes (PROMIS pain interference *p* = .015; pain catastrophizing *p* = .005; anxiety *p* = .001; depression *p* = .008; stress *p* = .009). Frequency of app use correlated with lower BPI pain interference (*p* < .001) and pain catastrophizing (*p* = .018), and changes from baseline persisted in the intervention group at 12 weeks (*p* < .05).

**Discussion/Conclusions**: A short-term mobile app intervention resulted in improvements across pain-related physical and mental health outcomes compared to usual care.

**Virtual reality experiences and neural responses: Unraveling the dynamics of empathy and placebo effects**

Jewel Clark^a^, Carmen-Édith Belleï-Rodriguez ^a^, Roni Shafir^a^, Lakota Watson^a^, Yang Wang^a^ and Luana Colloca^a^

^a^University of Maryland Baltimore

**Introduction/Aim**: Observing another person receiving pain relief from a treatment can influence one’s behavioral response to the same treatment. However, the behavioral and brain mechanisms underlying observational hypoalgesia remain unclear. We examined the effects of immersive Virtual Reality (VR) versus non-VR and observational hypoalgesia, with human versus avatar demonstration, in inducing pain empathy and placebo effects.

**Methodology**: Using high-resolution electroencephalography (EEG), we investigated the neural individual differences underlying observational hypoalgesia and empathy in immersive VR and non-VR. Peak alpha frequency (PAF) from resting-state activity was recorded for 47 healthy adult participants. Participants underwent a social observation paradigm where they observed a demonstrator, avatar or human, experiencing pain under control and “treatment” conditions. Following the observation phase, state empathy measurements were obtained before each participant underwent a self-experience phase.

**Results**: Participants reported higher pain empathy for a human demonstrator than an avatar. The difference in empathy scores between the control and placebo conditions was greater in the non-VR setting compared to VR. During the testing phase, participants reported less pain unpleasantness in the placebo condition than in the control condition, suggesting that observational learning significantly induced placebo effects. Faster PAF was significantly associated with higher self-reported empathy for pain severity and unpleasantness, lower demonstrator empathy ratings, and greater observationally induced placebo effects.

**Discussion/Conclusions**: These results suggest that for most conditions, observation of a human demonstrator in pain induced greater pain empathy than an avatar in pain, independent of immersion levels. Further, individual neural activity differences in the somatosensorial region may predict an individual’s response to a placebo manipulation.

**Development and validation of pain classification models in EEG using convolutional neural networks**

Alyson Champagne^a^, Mathilda Buschmann^b^, Mathieu Royc^3^ and Michel-Pierre Coll^a^

^a^Université Laval, ^b^Universität Osnabrück, ^c^McGill University

**Introduction/Aim**: Pain is experienced differently by each person, depending on their previous experiences, emotions, and mood. In research contexts, pain is assessed by scales or verbal reports, measures with several limitations. Thus, the scientific community has invested considerable effort in creating pain biomarkers based on brain activity recorded by electroencephalography (EEG) that could provide a complementary way of measuring pain. The aim of this project is to leverage recent developments in machine learning to create models capable of identifying the presence of pain in people based on their EEG activity.

**Methodology**: EEG data was collected from 43 healthy individuals under three conditions: resting state (five minutes), experiencing tonic thermal pain for 8 minutes, with or without providing an intensity rating, and listening to an unpleasant auditory stimulus for 8 minutes, with or without rating its unpleasantness. The EEG data was used to train deep and shallow convolutional neural networks (CNN) to classify data segments across participants in three categories (resting, pain, auditory). Nested cross-validation was used to optimize hyperparameters and the model’s performance was then assessed with new data from 23 healthy individuals experiencing the same conditions.

**Results**: The shallow CNN showed the best classification performance compared to the deep CNN. The accuracy of the shallow CNN was significantly above chance in both cross-validation (mean test accuracy = 0.518) and the new data (mean test accuracy = 0.483).

**Discussion/Conclusions**: Results confirm the ability of convolutional models to distinguish the EEG signal associated with pain from other conditions using cross-validation and in new data.

**User-centered design of telehomecare in a continuum of care for chronic pain: Patient and provider needs assessment**

Regina Visca^a, b, c^, Krista Brecht^b, c^, June Litowski^d^, Martine Leroux^d^, Rona Fleming^e^, Flavie Laliberté^e^ and Yoram Shir^a, b, c^

^a^McGill University; ^b^McGill University Health Center; ^c^McGill RUISSS Center of Expertise in Chronic Pain; ^d^CIUSSS Center-Ouest-de-l’Île-de-Montréal; ^e^Patient Partner

**Introduction/Aim**: Telehomecare monitoring (TM) is a promising tool to support chronic pain (CP) management through routine and timely data transmission, enabling clinicians to identify symptom exacerbations and intervene early. However, the limited research on TM for CP does not address users’ needs. The aim of this study was to determine the perceived needs of patients and clinicians with respect to using TM to support CP management.

**Methodology**: Semi-structured focus groups were conducted with patients and clinicians using user-centered design principles. A descriptive qualitative research design was used to identify the perceived needs of users, TM features that support those needs, and perceived benefits. Participants were recruited from pain clinics between April 2023 to September 2023.

**Results**: Participants (12 clinicians/6 patients) identified barriers to care at the patient, health systems and societal levels that could be addressed by TM. Five themes focusing on design features were identified: 1) Biopsychosocial domains to monitor and manage; 2) Real-time information sharing between patients and providers; 3) Actionable monitoring to alert clinicians to signs of regression in patient health; 4) TM features to support behavioral activation including improved adherence to medication and tailored coping strategies; and 5) Human connection and compassionate care. Participants recognized the potential value of TM in supporting continuity and coordination of care, building therapeutic relationships, and enabling behavior change.

**Discussion/Conclusions**: From the participants’ perspective, TM that addresses patient- and provider-identified needs and supports theory-based functionalities including symptom monitoring, behavioral activation and patient-provider interactions could be beneficial to CP patients. Next steps include development, usability testing and impact/implementation evaluation.

**Prolonged pain in premature neonates hospitalized in neonatal intensive care units: A scoping review**

Alexandra Breton-Piette^a, b^, Gwenaelle De Clifford-Faugère^c^ and Marilyn Aita^a, b, d^

^a^Faculty of Nursing, Université de Montréal; ^b^Research Center, CHU Sainte-Justine; ^c^Université du Québec en Abitibi-Témiscamingue; ^d^Quebec Network on Nursing Intervention Research (RRISIQ)

**Introduction/Aim**: Procedural pain in premature neonates is routinely studied, while prolonged pain remains understudied and ambiguous among clinicians and researchers. Lacking a definition and an agreed upon taxonomy of prolonged pain, this concept is under recognized, under evaluated and under treated, leading to significant long-term pain consequences in premature neonates.

To determine the scope, extent, and nature of the available literature on prolonged pain in premature neonates hospitalized in the neonatal intensive care unit (NICU).

**Methodology**: An electronic search was conducted in CINAHL, PubMed, Medline, Web of Science, and the gray literature.

**Results**: 86 articles met the inclusion criteria and were included in the scoping review. Key concepts of neonatal prolonged pain identified were definitions (*n* = 26). Although definitions of neonatal prolonged pain remain large in their scope, a time criterion was identified as an important element in defining it. The context of hospitalization, such as mechanical ventilation, necrotizing enterocolitis, and the postoperative period, was identified as being the most indicative of a prolonged pain state in premature neonates. Furthermore, tools commonly used to measure prolonged pain included the EDIN, COMFORTneo, and the N-PASS, while very limited interventions were shown to be effective specifically for alleviating prolonged pain in premature neonates.

**Discussion/Conclusions**: Prolonged pain can originate from different contexts routinely found in the NICU and the most current contexts of hospitalization identified in this scoping review should guide the health care professional to perform frequent pain evaluations with a tool validated for prolonged pain.

**Weathering pain – allostatic load and pain in the UK biobank**

Jax Norman^a^, Matthew Fillingim^a^, Gianluca Guglietti^a^, Azin Zare^a^, Christophe Tanguay Sabourin^b^, Ronrick Da-ano^a^ and Etienne Vachon-Presseau^a^

^a^McGill University; ^b^Université de Montréal

**Introduction/Aim**: The weathering hypothesis posits that racial and ethnic minority groups are disproportionately susceptible to adverse health outcomes, such as chronic pain, owing to prolonged exposure to socioeconomic challenges and systemic disparities. This research aims to quantify the prevalence of chronic pain across different racial and ethnic populations and to elucidate the determinants of disparities in chronic pain.

**Methodology**: This investigation utilized initial anthropometric, metabolic, cardiovascular, and immunological metrics, along with self-reported sociodemographic information, from the UK Biobank dataset. We computed the prevalence rates of chronic pain and widespread chronic pain within each demographic segment, employing chi-square tests to evaluate the statistical significance of variations in these rates across groups.

**Results**: Our findings indicate a markedly higher occurrence of chronic pain in Black and Asian females (52.3% and 54.3%, respectively) compared to their White counterparts (45.5%; *p* < .001). Furthermore, widespread chronic pain showed greater prevalence among Black and Asian subjects (Females: 4.0% and 4.5%; Males: 1.7% and 2.5%, respectively) than in White individuals of corresponding gender (Females: 1.4%, *p* < .001; Males: 1.0%, *p* < .05).

**Discussion/Conclusions**: The analysis revealed that White participants exhibited a lower prevalence of widespread chronic pain compared to Black and Asian participants, and White females had a lower incidence of chronic pain in general than Black or Asian females. Subsequent investigations will aim to ascertain the role of allostatic load in the differential prevalence of pain observed in racial and ethnic minority groups and to identify additional factors contributing to these observed disparities.

**The CPOT-Neuro for pain assessment in critically ill adults with a brain injury: Interrater agreement between bedside and video raters**

Céline Gélinas^a^, Vivienne Nguyen^a^ and Melissa Richard-Lalonde^a^

^a^McGill University

**Introduction/Aim**: Pain assessment in critically ill adults with a brain injury is challenging as their level of consciousness may fluctuate. The Critical-Care Pain Observation Tool (CPOT), an alternative reference standard measure for the critically ill unable to self-report, was recently adapted for this subpopulation (5-item CPOT-Neuro with scores 0–8). This study aimed to examine interrater agreement between bedside and video raters.

**Methodology**: A methodological design with two methods of rating were used (bedside versus video) during standard care (i.e., noninvasive blood pressure [NIBP] and bed turning). Bedside raters were research staff and two video raters had different background (health and nonhealth disciplines). All raters received a standardized 45-minute training by the principal investigator.

**Results**: Videos of 56 patients with a brain injury (trauma related or not) at different levels of consciousness (31 conscious, 17 altered LOC, 8 unconscious) were included. Patients were middle-aged (57.5 years), most were male (66%) and Caucasian (75%). Raters provided low and high CPOT-Neuro scores during NIBP and bed turning, respectively. Interrater agreement of CPOT-Neuro scores was supported with intraclass correlation coefficients (ICCs) > 0.65 for each procedure. Interrater agreement was highest during bed turning in the conscious group with ICCs ranging from 0.79 to 0.90.

**Discussion/Conclusions**: Video technology was useful to achieve acceptable interrater agreement of CPOT-Neuro scores between bedside and video raters for research purposes. The use of video was challenging for some behaviors (i.e., tearing, face flushing) which were influenced by lighting and the camera angle. Ventilator alarms were challenging to identify for the video rater from a nonhealth discipline.

**Closing the gap: Effectively using knowledge translation to improve quality of surgical pain relief**

Salima S. J. Ladak^a^, Diana Tamir^b^, Nicholas Vaccariello^b^ and Hance Clarke^c^

^a^University Health Network and Lawrence S. Bloomberg Faculty of Nursing; ^b^University Health Network, Toronto General Hospital; ^d^University Health Network Department of Anesthesia and Pain Management, University of Toronto

**Introduction/Aim**: Challenges of timely integration of health research into clinical practice has been well described. Despite surgical guidelines recommending use of intravenous (IV) lidocaine and ketamine as efficacious treatment options for postsurgical pain, adoption of these medications in the clinical setting have been challenging. This poster will describe the integration, evaluation and sustained practice of using IV lidocaine and ketamine as viable options for multimodal postoperative pain management in the surgical program of a quaternary academic health science center care in Canada.

**Methodology**: The Dignan and Carr Health Program Implementation Model was most feasible for the structures and processes of our hospital and was used to guide systematic integration of IV lidocaine ketamine as postoperative analgesics.

**Results**: Initial adoption was undertaken on a general and thoracic surgery ward and the program was monitored for a 3-month period. No adverse events were reported during this time. Following this initial uptake and sustainability, both medications have been successfully integrated across 6 surgical subspecialties.

**Discussion/Conclusions**: Key success factors that enabled knowledge translation and program sustainability included: 1) focused education for inter-professional nursing, pharmacy and medicine teams; 2) the development of easily accessible reference materials required for clinical practice; and, 3) the establishment of a mechanism for program implementation based on ward-specific structure and operation, management and evaluation, as well as providing educational updates at specific time intervals. We have refined this process within our organization and support the use of this knowledge translation model for future pain management therapies.

**Strategic approaches to research impact in chronic pain: A focus on equitable approaches to knowledge mobilization**

Megan MacNeil^a, b^, Jenny Olson^c^, Justin Presseau^c^, Alex Haagaard^d^, Norman Buckley^e^, Brandon Van Dam^e^ and Kathryn Birnie^b^

^a^University of Alberta; ^b^University of Calgary; ^c^University of Ottawa; ^d^Chronic Pain Network; ^e^McMaster University

**Introduction/Aim**: Chronic pain affects 1 in 5 Canadians. Lack of timely diagnosis and treatment leads to disability and reduced quality of life. This can be exacerbated for equity-denied groups and when people experience pain-related stigma. There is a need to ensure the right research findings impact clinical care and policy for populations disproportionally affected by barriers to pain care. Knowledge mobilization (KM) approaches can be leveraged to bridge this research-to-practice gap.

**Methodology**: The Chronic Pain Network (CPN) has generated actionable evidence to improve pain care in partnership with people with lived experience. The CPN has a national infrastructure to facilitate KM, including leadership from healthcare professionals, researchers, organizations, and people with lived experience. We are using an equity-focused, evidence-informed approach to KM (e.g., Knowledge to Action, Participatory Action Research, Epistemic Solidarity) to: 1) facilitate collaboration toward a consistent approach to pain management across Canada; 2) advanced pain education and awareness and training capacity; and, 3) co-design evidence-informed resources and tools using tailored and impactful strategies for multiple audiences. Our KM activities are informed by priority-setting dialogs led by the McMaster Health Forum and Health Canada’s “Action Plan for Pain in Canada” (2021).

**Results**: By maintaining a hub of chronic pain research, evidence is being scaled to ensure quality, equitable pain care. Tailored KM is resulting in the cocreation of evidence synthesis and new tools/resources for diverse audiences.

**Discussion/Conclusions**: A patient-oriented and systematic approach to KM-IS leads to actionable evidence for the scale-up of innovations or adaptation of existing knowledge.

**Assessing the feasibility of a clinical trial to evaluate an advanced practice physiotherapy model of care in chronic pain management: a feasibility study**

Jordan Miller^a^, Abey Abebe^a^, Tom Doulas^b^, Etienne Bisson^b^, Mulugeta Chala^c^, Chad McClintock^a^, Kevin Varette^a^, Kyle Vader^a^, Francois Desmeules^d^, Kadija Perreault^e^, Catherine Donnelly^a^, Randy Booth^a^, Andrews Tawiah^c^ and Scott Duggan^b^

^a^Queen’s University; ^b^Kingston Health Sciences Center; ^c^Western University; ^d^Universite de Montreal; ^e^Universite Laval

**Introduction/Aim**: Practice guidelines recommend interprofessional chronic pain management, but chronic pain clinics often have lengthy waitlists. Advanced practice physiotherapists (APP) in orthopedic clinics and emergency departments have provided effective care and reduced wait times. The purpose of this study was to determine the feasibility of a clinical trial to evaluate the effects of integrating an APP into an interprofessional chronic pain clinic.

**Methodology**: A single-arm feasibility study with embedded qualitative interviews exploring contextual factors influencing implementation from the perspectives of patients and providers was conducted. Patients with chronic musculoskeletal pain referred to an interprofessional chronic pain clinic were invited to participate. The APP model of care involved participants seeing a physiotherapist as the first point of contact within the team and supporting the patient by coordinating care. Feasibility outcomes were recruitment, retention and assessment completion rates, and fidelity of the model of care. Interviews were completed and analyzed using interpretive description.

**Results**: Forty-five participants (21 men; 24 women) with chronic musculoskeletal pain and a mean age of 51.47 (*SD*:13.45) years were recruited over 18 weeks. Thirty-four (76%) and 28 (62.2%) participants completed assessments at 3 and 6 months, respectively. Over 90% of assessment items were completed. The APP model of care was carried out with high fidelity and interviews identified organizational and provider level contextual factors that could be addressed to improve implementation.

**Discussion/Conclusions**: The APP model of care was carried out with high fidelity; however, alternate strategies to address attrition challenges are needed prior to proceeding with a clinical trial.

**Healthcare professionals’ perspectives on a self-management electronic education resource (E-Resource) for adults with high needle fear: A qualitative content analysis**

Emma Truffyn^a^, Kaytlin Constantin^a^, Anna Taddio^b^ and C. Meghan McMurtry^a, c, d^

^a^Department of Psychology, University of Guelph, Guelph, ON, Canada; ^b^Leslie Dan Faculty of Pharmacy, University of Toronto, Toronto, ON, Canada; ^c^Pediatric Chronic Pain Program, McMaster Children’s Hospital, Hamilton, ON, Canada; ^d^Department of Pediatrics, Schulich School of Medicine and Dentistry, Western University, London, ON, Canada

**Introduction/Aim**: Needles are painful and highly fear-inducing for ~10% of adults. Clinical practice guidelines exist on the management of pain due to immunizations and interventions for individuals with high needle fear. High needle fear in adults presents a challenge for clinician immunizers (CIs) to carry out procedures and manage pain effectively. Although mental health clinicians (MHCs) can treat high needle fear, they are rarely present in the procedural setting. A stepped-care approach, including self-guided educational resources, is recommended to address the gap between treatment demand and the availability of relevant professionals. Our objective was to elicit HCP feedback on a self-guided e-resource designed to address high needle fear in adults.

**Methodology**: An e-resource was drafted based on CPGs, extant literature, and team expertise. 18 HCPs (*n* = 10 CIs, *n* = 8 MHCs) shared their perceptions of the e-resource via interviews/focus groups. A qualitative content analysis was used to analyze the data.

**Results**: HCPs provided insight into what they liked about the content (e.g., coping statements, exposure, fainting, and information) and design/organization of the e-resource and proposed changes (e.g., chunking material, adding visuals and supporting tools, managing expectations and setbacks). HCPs noted that the e-resource was accessible and provided ideas for dissemination. HCPs recommended simplified language and highlighted barriers for clients (e.g., motivation) using the e-resource.

**Discussion/Conclusions**: HCPs were satisfied with the e-resource and would recommend it to both clients and other HCPs. Results highlight initial strengths of the e-resource, provide guidance for updates, and will inform future acceptability testing and dissemination efforts.

**Adaptive mentoring networks: Provider engagement in building primary care capacity for the management of chronic pain, substance use and mental health**

Marielle Darnley^a^, Stéphanie Lalande^b^, Joshua A. Rash^c^, Laura M. Visentin^a, d^, Diana Marsilio-Apostoli^e^, Sara Davidson^f^, David Flusk^g^, Joseph Rasimas^h^, Samuel Hickcox^i^, Helena Daudt^j^, Paula Hutchinson^k^ and Arun Radhakrishnan^a, l^

^a^Department of Family Medicine, University of Ottawa; ^b^Clinical Psychology, University of Ottawa; ^c^Department of Psychology, Memorial University of Newfoundland; ^d^The Ottawa Hospital Academic Family Health Team; ^e^Centre for Effective Practice; ^f^Riverstone Recovery Center; ^g^Memorial University of Newfoundland; ^h^Dalhousie University; ^i^Nova Scotia Health Authority; ^j^PainBC Society; ^k^Horizons Community Development Associates; ^l^Bruyere Research Institute

**Introduction/Aim**: Adaptive Mentorship Networks (AMNs) are an educational innovation used in Canada to improve primary care capacity and provide compassionate, quality care for those with chronic pain (CP), mental health (MH), and substance use concerns (SU). AMNs create mentoring communities to: 1) support wellbeing of providers; 2) increase knowledge and confidence managing clinical complexity; and, 3) enhance the delivery of compassionate care.

**Methodology**: Survey data as of 2023 was collected from volunteer mentees from AMNs in BC, NB, NL, NS, and PEI (*N* = 390), to capture participation and engagement. Descriptive statistics were used to characterize mentees, and content analysis to characterize responses to open-ended questions about their motivations.

**Results**: 390 mentees completed the surveys, representing 20 healthcare professions, including nurses (21%), physicians (28%), physiotherapists, pharmacists, and social workers. Practice location data placed 40% in large urban areas, 46% in rural, 7% in medium urban, and 5% in small rural centers. 48% were in early career, 35% in midcareer, and 5% in late career. The most frequent motivations to join included: 1) building interprofessional bridges; 2) building a community of practice; 3) desire to improve care; and 4) previous enjoyment learning from mentors.

**Discussion/Conclusions**: AMNs are engaging a range of healthcare professions at varying career stages, practicing across diverse regions. Participants are motivated to engage in interprofessional communities of practice that enhance care for the management of CP, MH, and SU in Canada. Evaluation data being collected from each AMN will provide details about their impact on mentees over time.

**Translation and validation of the Pain Coping Questionnaire in French-Canadian adolescents with musculoskeletal pain**

Janick Carmel^a^, Laurence Lessard^a^, Raíssa Passos dos Santos^a^, Stéphany Cara-Slavich^a^, Annie Sylfra^b^, An Kateri Vu^b^, Pascale Ouimet^a^, Isabelle Turgeon^a^, Soraya Barchi^a^, Julie Joncas^a^, Stefan Parent^a^ and Sylvie Le May^a^

^a^Centre de recherche du CHU Sainte-Justine; ^b^Université de Montréal

**Introduction/Aim**: Musculoskeletal (MSK) conditions are painful and affect 1 in 4 children, yet little is known on how these children cope with pain. The Pain Coping Questionnaire (PCQ) is a self-reported measure (39 items, 8 subscales) used to assess pain coping strategies in adolescents suffering from MSK pain. However, no French version of the PCQ is available. This research aimed to validate a French-Canadian version of PCQ (PCQ-F) with a population of adolescents suffering from painful MSK conditions.

**Methodology**: The PCQ was translated from English to French with a forward-backwards translation process, then it was reviewed by experts for relevance and clarity. This translated version was administered for validation to patients aged 11 to 18 years who experienced significant MSK pain in a pediatric hospital in Montreal. Construct validity was verified using a Principal Component Analysis (PCA), divergent validity using a Pearson correlation between the Child Post-Traumatic Stress Index (CPTS) and the PCQ-F, and reliability using Cronbach’s alphas.

**Results**: A total of 163 participants completed the questionnaire. The PCQ-F contains 37 items distributed in a seven-factor structure. Cronbach’s alpha was 0.88 for the global scale and between 0.58 and 0.67 among the subscales. Correlation between the CPTS and the PCQ-F was *r*=0.45, *p*=.008.

**Discussion/Conclusions**: Construct validity was supported by the PCA and reliability by the global Cronbach’s alpha which was considered good. Divergent validity was partially supported by the moderate positive correlation between the PCQ-F and the CPTS. PCQ-F is a valid and reliable questionnaire to assess pain coping strategies in adolescents with painful MSK conditions.

**Acceptability and feasibility of a Patient-Oriented Music Intervention (POMI) for use in the adult intensive care unit: A crossover pilot randomized controlled trial**

Melissa Richard-Lalonde^a, b^, Nancy Feeley^a, b^, Sylvie Cossette^c, d^, Linda Chlan^e^ and Céline Gélinas^a, b^

^a^McGill University; ^b^Jewish General Hospital; ^c^Université de Montréal; ^d^Montreal Heart Institute; ^e^Mayo Clinic

**Introduction/Aim**: Music is suggested as a complementary pain management intervention in the intensive care unit (ICU). Evidence on the acceptability and feasibility of music interventions in the ICU is scarce. A patient-oriented music intervention (POMI) was developed using a music streaming service to generate individualized playlists. This study aimed to evaluate acceptability and feasibility of POMI, feasibility of research methods, and POMI preliminary efficacy to reduce pain.

**Methodology**: In this crossover pilot randomized controlled trial, three categories of participants were recruited: ICU patients able (*n* = 12) and unable to self-report (*n* = 12); family members (*n* = 12); and nurses (*n* = 12). Patients were randomized to sequence 1 (POMI period, then control period without music) or sequence 2 (control, then POMI), with a 4-hour washout period between the two. POMI and control intervention lasted at least 20 minutes, before turning procedure. Outcomes included acceptability and feasibility of POMI, feasibility of research methods and pain scores. Pain was measured at four timepoints (T0: pre-intervention; T1: postintervention; T2: during turning; T3: 30-minutes postturning).

**Results**: Of 347 patients screened, 53 were eligible and 24 consented to participate. Participants evaluated POMI as acceptable. POMI was feasible (>80% patients) although turning occurred 0–101 minutes after POMI, making the timing of the POMI challenging. Self-reported pain and behavioral scores were lower (*p* <.10) after POMI compared to control period.

**Discussion/Conclusions**: POMI was acceptable and feasible as complementary pain management intervention. POMI duration could be tailored to better synchronize with unpredictable turning. Eligibility rate was low and further research is warranted to determine POMI efficacy to reduce ICU pain.

**Pharmacological prophylaxis for chronic migraine: A systematic review and network meta-analysis of randomized controlled trials**

Malahat Khalili^a^, Amin Liaghatdar^b^, Fatemeh Mahdian^c^, Tal Levit^d^, Sara Moradi^e^, Ehsan Hedayati^f^, Kian Torabiardakani^d^, Farzaneh Ahmadi^g^, Sahar Khademioore^e^, Ahmad Sofi-Mahmudi^e^, Tariq Atkin-Jones^h^, Vivek Patil^i^, Fatemeh Mirzayeh Fashami^e^, Soheil Mehmandoost^j^, Harjind Kahlon^d^, Rachel J. Couban^k^, Mohammad Fereshtehnejad^l^, Sangita Sharma^i^, Kameshwar Prasad^m^, Jason W. Busse^k^ and Behnam Sadeghirad^k^

^a^Michael G. DeGroote Institute for Pain Research and Care, ^b^Isfahan University of Medical Sciences, Isfahan, Iran, ^c^Mazandaran University of Medical Sciences, Mazandaran, Iran, ^d^Faculty of Health Sciences, McMaster University, ON, Canada, ^e^Department of Health Research Methods, Evidence, and Impact, McMaster University, Hamilton, Ontario, Canada, ^f^Ahvaz Jundishapur University of Medical Sciences, Ahvaz, Iran, ^g^Faculty of Dentistry, Islamic Azad University Shiraz Branch, Shiraz, Iran, ^h^Faculty of Health Sciences, McMaster University, Ontario, Canada, ^i^McMaster University, Ontario, Canada, ^j^Department of Biostatistics and Epidemiology, Faculty of Public Health, Kerman University of Medical Sciences, Kerman, Iran, ^k^Department of Anesthesia, McMaster University, Hamilton, Ontario, Canada, ^l^Division of Neurology, Department of Medicine, University of Toronto, Toronto, Ontario, Canada, ^m^ Rajendra Institute of Medical Sciences, Jharkhand, India

**Introduction/Aim**: Chronic migraine is a common cause of disability for which several interventions are available to prevent the onset of symptoms or mitigate their severity. Understanding the comparative effectiveness of current pharmacological prophylactics is crucial for informed clinical decisions and to guide future research. We performed a network meta-analysis of randomized controlled trials to assess the comparative effectiveness of available pharmacological prophylaxis for migraines.

**Methodology**: We searched MEDLINE, EMBASE, Web of Science, Scopus, PsycINFO, and Cochrane CENTRAL up to October 2023 for trials that: (1) enrolled adults diagnosed with chronic migraine, and (2) randomized them to any prophylactic medication vs. another medication or placebo. We performed a random-effects frequentist network meta-analysis for all patient-important outcomes.

**Results**: We included 193 randomized trials. Compared to placebo, CGRP monoclonal antibodies (mean difference [MD] −1.7, 95%CI: −1.1 to −2.2), injection of botulinum toxin (MD −1.8, 95%CI: −0.7 to −2.9), calcium channel blockers (MD −1.8, 95%CI: −0.5 to −3.0), beta-blockers (MD −1.4, 95%CI: −0.2 to −2.6), and anticonvulsants (MD −1.1, 95%CI: −0.4 to −1.8) were among the most effective treatments in reducing average number of headache days per months. Anticonvulsants (Risk Ratio [RR] 2.3, 95%CI: 1.8 to 3.0), calcium channel blockers (RR 1.8, 95% CI: 1.1 to 3.1), and tricyclic antidepressants (RR 2.3, 95% CI: 1.3 to 3.8) showed the highest risk of discontinuation due to adverse events.

**Discussion/Conclusions**: Our findings suggest that CGRP inhibitors, botulinum toxin, and beta-blockers may provide the greatest benefit, and tolerability, for reducing the frequency of migraine headaches.

**Biomarkers for specific etiologies, pathophysologies and symptoms of chronic pain**

Matt Fillingim^a^, Christophe Tanguay-Sabourin^b^, Jax Norman^a^, Gianluca Gugliette^a^ and Etienne Vachon-Presseau^a^

^a^McGill University; ^b^University of Montreal

**Introduction/Aim**: Chronic pain’s diverse etiology poses a challenge to finding a universal biomarker. This study aims to identify and test biomarkers for pain-associated medical conditions, with the hypothesis that distinct pathophysiologies will express through different, unrelated biomarkers. The study also postulates that a biological signature predicting medical conditions would partly reflect chronic pain’s clinical classifications (i.e., nociceptive, nociplastic, and neuropathic).

**Methodology**: The study used data from 493,211 UK Biobank participants. We trained machine learning models to predict 39 pain-associated medical conditions (i.e., derive biomarkers) based on brain imaging, blood immunoassays, and genome-wide associations.

**Results**: Our study findings underline the fact that different biological factors predict various medical conditions. For instance, blood immunoassays proved most effective in predicting gout and Crohns Disease, while diffusion-weighted imaging (DWI) was superior in forecasting conditions such as joint pain and soft tissue inflammation. Further, we found that medical conditions characterized by similar clinical classifications of pain (nociplastic, nociceptive, neuropathic) tended to be predicted by similar biological indicators. For example, resting-state functional magnetic resonance imaging (rsfMRI) proved most effective in predicting nociplastic pain conditions such as widespread pain, fibromyalgia, and chronic fatigue syndrome. The top 18 biomarkers, chosen based on their highest ROC-AUC scores in predicting medical conditions are presented below.

**Discussion/Conclusions**: Our findings indicate that various biological markers could potentially predict diverse types of chronic pain conditions, furthering our understanding of their etiology and potentially guiding targeted treatment strategies. Additionally, it emphasizes the importance of characterizing pain conditions based on etiology and biological factors to identify clinically useful biomarkers.

**Association of gender role expectations of pain and measures of pain sensitization in people with knee osteoarthritis: A cross-sectional study**

YV Raghava Neelapala^a^, Saurab Sharma^b^ and Lisa Carlesso^c^

^a^PhD Candidate, School of Rehabilitation Science, McMaster University, Canada, ^b^Postdoctoral Research Fellow, School of Health Sciences, Faculty of Medicine and Health, University of New South Wales; and Center for Pain IMPACT, Neuroscience Research, Australia, ^c^Associate Professor, School of Rehabilitation Science, McMaster University, Canada

**Introduction/Aim**: As opposed to sex, the association of gender and its stereotypic roles with pain sensitivity has not been explored previously in people with knee osteoarthritis (KOA). We, therefore, examined the association between Gender Role Expectations of Pain (GREP), and measures of pain sensitization in KOA. We also explored whether the association differed by level of GREP items (high vs low scores).

**Methodology**: Participants aged above 40 years, diagnosed with KOA with an initial orthopedic surgeon consult were included in this secondary analysis from a multi-center cohort study in Montreal. GREP pain sensitivity and pain endurance items for the typical man or woman were rated by males and females respectively. Psychophysical tests included pressure pain thresholds (PPTs), temporal Summation (TS), and conditioned pain modulation (CPM). Multiple linear regression models for males and females were conducted with GREP scores and psychophysical tests adjusting for age, BMI, pain catastrophizing, anxio-depressive symptoms, and radiographic severity. Next models stratified on the median split of GREP scores were examined.

**Results**: 280 participants (57% females; age (SD): 63.9 (9.6) and BMI (SD): 31.3 (8.40)) were included. GREP pain sensitivity scores in males were associated with CPM values (b: 95% CI: 0.09 (0.01 to 0.17)). Males with low GREP pain sensitivity or pain endurance had very small to small positive associations with PPT and CPM values.

**Discussion/Conclusions**: This first exploration of gendered pain sensitivity and pain endurance in people with KOA has small and clinically unimportant associations with measures of pain sensitization requiring further study.

**A machine learning algorithm aids in identifying predictors for acute pain and chronic postoperative pain: A prospective cohort study**

Leann Lac^a, b^, Karen Ferreira^c^, Berlant AlSabbagh^d, f^, Pingzhao Hu^a, g^ and Ana Velly^d, e, h^

^a^Department of Computer Science, University of Manitoba, ^b^Department of Statistics, University of Manitoba, ^c^Hôpital du Suroit, CISSS de la Montérégie Ouest, Québec, Canada, ^e^Department of Dentistry, Jewish General Hospital, ^f^Lady Davis Institute for Medical Research, ^g^Department of Biochemistry, Schulich School of Medicine and Dentistry, Western University, ^h^Faculty of Dentistry, McGill University

**Introduction/Aim**: To identify the contributors of persistent pain 7 days, and at 3–6 months after breast cancer surgery.

**Methodology**: Female participants (above 18 years of age) with breast cancer who underwent breast cancer surgery (BCS) were recruited from Jewish General Hospital, Montreal. The putative risk factors (i.e., anxiety, depression, pre-operative pain, and medical conditions) were assessed on the day of enrollment and type of surgery after the surgery. Pain intensity (0–10 numerical rating scale NRS) assessments were conducted by telephonic interview using the Brief Pain Inventory scale before surgery, 7 days after and at 3–6 months after BCS. The logistic least absolute shrinkage and selection operator (LASSO) was used to assess the contributors to composite pain index.

**Results**: 268 participants (mean age = 56.3, standard deviation = 14.2) participated in this study. Logistic LASSO analysis showed that segmental mastectomy (SM) (odds ratio [OR] =0.26), pain before surgery (OR = 1.54), depression (OR = 1.15), pain hours after surgery (OR = 1.71), infiltration (OR = 1.45), painful comorbidities (OR = 1.36), and diabetes (OR = 1.24) were associated with the pain seven days after surgery. Pain before surgery (OR = 1.76), depression (OR = 1.45), SM (OR = 0.95), infiltration (OR = 1.16), and pain 7 days after surgery (OR = 1.6) were related to pain 3-month after surgery. Furthermore, severity of pain at 6 months was associated with pain before surgery (OR = 1.03), pain 7 days after surgery at rest (OR = 1.02), and movement (OR = 1.55).

**Discussion/Conclusions**: Pain severity is associated with persistence of pain during all follow-ups. Other risk factors have a shorter impact. This result indicates that effective management of pain intensity is crucial for preventing chronic pain persistence following surgery.

**Building capacity in primary care using a collaborative approach: Comprehensive education for healthcare professionals in primary care on pain management in adults**

Regina Visca^a, b, c^, Claire Nehme^d^, Sindiane Bouchaala^e^, Mylène Laberge-Homsy^e^, Sabrina Mitrovic^c^, Sandra Gaudet-Menard^b^ and Krista Brecht^b, c^

^a^McGill University, ^b^McGIll University Health Center, ^c^McGill RUISSS Center of Expertise in Chronic Pain, ^d^CIUSSS du Nord-de-l’île de Montréal, ^e^Centre intégré universitaire de santé et de services sociaux de l’Ouest-de-l ’Île-de-Montréal

**Introduction/Aim**: In Canada, chronic pain (CP) patients are mainly treated by primary care clinicians (PCC). However, the clinical management of CP is complicated by inadequate continuing professional development (CPD). With the release of Canadian/provincial recommendations, there is an impetus to understand the knowledge gaps faced by PCCs. The aim of this study is to elicit PCCs views about enhancing knowledge to provide effective CP care.

**Methodology**: An explanatory sequential mixed-methods design was used to identify knowledge gaps and determine learning objectives for online learning modules. Using a provincial database of PCCs, we conducted a cross-sectional online survey of CPD needs and preferred learning objectives. Results from the survey were used to inform focus-group interviews (3) conducted with a convenience sample of PCCs (N =20). Data were analyzed using content analysis (April 2023 – September 2023).

**Results**: Respondents (*N* =450) were primarily nurses (43%), physiotherapists (13%) and social workers (22%), university educated (90%), working in primary care clinics (92%). Most respondents (80%) had not received any form of training in the last 3 years, with over 50% self-identifying as beginners in the areas of neuroplasticity, organization of service delivery, management of patient trajectories, and continuity and coordination of care. Five CPD themes were identified: whole-person care; interdisciplinary integrated care; digitally enabled care; patients’ roles; and vulnerable populations.

**Discussion/Conclusions**: Quebec PCCs require greater access to CPD to build knowledge and confidence in pain management using culturally responsive approaches. The goal is to co-design, with patients and PCCs, asynchronous modules in interprofessional CP management that provide comprehensive knowledge aligned with best practices.

**The link between self-reported bruxism and increased severity of pain-related temporomandibular disorders: A cohort study**

Sherif ElSaraj^a, b^, Neha Thakur^a^, Berlant AlSabbagh^b, c^and Ana Velly^a, b, c^

^a^Faculty of Dentistry, McGill University, ^b^Department of Dentistry, Jewish General Hospital, ^c^Lady Davis Institute for Medical Research

**Introduction/Aim**: Individuals experiencing pain-related temporomandibular disorders (PTMD) often report instances of clenching and grinding teeth. Earlier research has identified a tentative connection between these behaviors and the likelihood of developing TMD as a result. This prospective cohort study was designed to explore the association between oral parafunctional behaviors and the severity of pain intensity over a 6-month follow-up period.

**Methodology**: Individuals with PTMD were recruited from four participating sites across Canada, and they were diagnosed through the Diagnostic Criteria for Temporomandibular Disorders (DC/TMD). Pain intensity characteristics (CPI) were measured using the Graded Chronic Pain Scale (GCPS) at baseline and during 3–6-month follow-ups. The Oral Behavior Checklist was utilized to evaluate oral parafunctional behaviors at baseline. The association between oral parafunctional behaviors and PTMD was examined through linear regression analysis.

**Results**: Out of the 456 subjects enlisted from 2015 to 2021, 378 successfully completed the follow-up. The findings revealed that self-reported clenching or grinding was linked to the severity of CPI over the 6-month follow-up period (β = 0.17, 0.02). These associations persisted for both evening (β = 0.87, P = .02) and daytime (β = 0.17, P = .03) bruxism.

**Discussion/Conclusion**s: The self-reported occurrence of bruxism is related to a higher severity of pain-related temporomandibular disorders (TMD) during follow-up. Importantly, this association remains unaffected by demographic characteristics and psychological symptoms. This outcome suggests that addressing these behaviors should be essential to treating PTMD.

**Neurocomputational dynamic of pain value-based decision making**

Léane Beaulieu-Laliberté^a^, Mathieu Roy^a^ and Michel-Pierre Coll^a^

^a^CIRRIS

**Introduction/Aim**: Pain is a salient learning signal, allowing the organism to avoid future dangers by assigning a negative value to potentially harmful actions. However, we still know little about how the brain assigns value to pain to guide behavior. In this study, we aim to investigate how the brain dynamically represents the prospect of pain in economic contexts and how this representation is integrated with external rewards to guide behavior.

**Methodology**: Fifty healthy adults (27 females, mean age = 24,72) took part in an experimental session during which electroencephalography (EEG) was recorded. During the first phase of the session, activity was recorded during the anticipation of painful electrical shocks and monetary rewards. In the main task, participants were asked to accept or reject offers, combining the various levels of pain and money shown in the first phase. If they accepted the offer, they received a painful shock and the opportunity to obtain the money. If they refused the offer, they did not receive any pain or money.

**Results**: Behavioral results replicate previous studies using a similar approach and show that participants’ decisions and deliberation times were significantly impacted by both the pain and money levels offered. Ongoing EEG analyses will investigate the time course of the processes underlying the decision to voluntarily approach pain in exchange for a reward.

**Discussion/Conclusions**: The current study will contribute to our understanding of the brain mechanisms involved in decision making about potential future pain. This research could contribute to our comprehension of disorders marked by either excessive or inadequate avoidance of pain.

**An #ItDoesntHaveToHurt needs assessment for a tool for parents to improve their child’s procedural pain**

Meredith Otley^a, b^, Justine Dol^a^, Jennifer A. Parker^a^ and Christine T. Chambers^a, b, c, d^

^a^IWK Health Center, Center for Pediatric Pain Research, Halifax, NS Canada, ^c^Dalhousie University, Faculty of Medicine, Halifax, Canada, ^b^Dalhousie University, Department of Psychology and Neuroscience, Halifax, Canada, ^d^Dalhousie University, Department of Pediatrics, Halifax, Canada

**Introduction/Aim**: Procedural pain remains an undertreated source of pain in children. Parents, who are often with their children during these procedures, could benefit from interventions that aim to improve use of evidence-based strategies for procedural pain management. As a follow-up to the #ItDoesntHaveToHurt initiative, the present study’s objectives were to (1) assess parents’ need for a tool to help manage their child’s procedural pain and (2) identify barriers and facilitators to their use of such a tool.

**Methodology**: A cross-sectional study of Canadian parents with a child between the ages of 0 and 17 years was conducted through an online survey platform. Parents were asked questions regarding their children’s procedural pain management needs.

**Results**: Among the 110 respondents, 55% were female, and 51% had female children. When asked to rate the need for a tool to help manage children’s procedural pain and the usefulness of a tool for them personally on a 5-point (1 = not at all to 5 = very) scale, the mean responses were 3.8 (*SD* = 0.89) and 3.9 (*SD* = 0.96), respectively. The most useful types of tools that parents identified were a mobile-app or a website. Parents reported high cost and inaccessibility as the main barriers to use of a tool and low-cost and ease of use as facilitators.

**Discussion/Conclusions**: These findings suggest that the majority of parents see a need for a low-cost, accessible tool to help manage their children’s procedural pain. This study provides support for the development of a mobile-app or website targeted at parents to help improve pediatric procedural pain management.

**Narratives of chronic pain from Armed Forces Veterans**

Thomas Kersey

Veterans and Families Institute for Military Social Research

**Introduction/Aim**: Although there is a wealth of data exploring chronic pain in Armed Forces veterans, the existing literature overwhelmingly views chronic pain through a quantitative lens which does not explore the depth of chronic pain lived experience as a result of this; therefore, it is unclear how veterans live with and manage their chronic pain.

**Methodology**: The stories of 14 veterans living with chronic pain were collected and analyzed using dialogical narrative analysis, which detail veterans’ autobiographical stories of living with chronic pain, and how they use these stories to make sense of their pain experience in the context of their identity as a veteran.

**Results**: Analysis identified that the participants understood and made sense of their chronic pain experience through three distinct storytelling phases. The military body, which situated a career that was marked by physicality and the churn of life within the military institution. The stigmatized body which situates the attitudes toward pain and weakness within the military and the consequences of showing that the military body is infallible, which in turn inform stoic attitudes toward pain and pain management. And, the moving body, stories about the moving body were about making sense of a new body with pain, and how movement is used to understand, learn, and respond to and from their bodies on any given day.

**Discussion/Conclusions**: Stories about chronic pain were told in the context of military experience and how the culture of the military shapes attitudes toward chronic pain, and movement was a keyway in which participants live with and manage their chronic pain.

**Sahaj Samadhi Meditation versus a Health Enhancement Program for depression in chronic pain: a randomized controlled trial**

Darren Cheng^a^, Kawsari Abdulah^b^, Robert Simpson^c^, Joel Katz^d^, Rahim Moineddin^c^, Benoit Mulsant^c, e^, Akshya Vasudev^f^, Michelle Greiver^c, g^, Fardous Hosseiny^h^, Marco Inzitari^i, j^, Ronnie Newman^k, l^, Leon Rivlin^b^, Kirk Foat^m^, Andrea Furlan^n^, John Flannery^n^, Deanna Telner^o^, Rachael Bosma^p^, Michelle Naimer^c^, Chadwick Chung^q^, Andrew Pinto^r^, Michelle Nelson^a, c^, Ross Upshur^a, c^and Abhimanyu Sud^b, c^

^a^Lunenfeld-Tanenbaum Research Institute, ^b^Humber River Health, ^c^University of Toronto, ^d^York University, ^e^Centre for Addiction and Mental Health, ^f^ Medpoint Clinic, ^g^North York General Hospital, ^h^Royal Ottawa Mental Health Center, ^i^Parc Sanitari Pere Virgili and VHIR, Barcelona, ^j^Open University of Catalonia (UOC), ^k^Art of Living Foundation, ^l^Nova Southeastern University, ^m^Patient advisor, ^n^University Health Network, ^o^South East Toronto Family Health Team, ^p^Women’s College Hospital, ^q^Canadian Memorial Chiropractic College, ^r^St. Michael’s Hospital

**Introduction/Aim**: Meditation has demonstrated efficacy for both chronic pain and depression independently, yet few studies have examined its effectiveness when both conditions are present concurrently. Our aim was to assess the effectiveness of Sahaj Samadhi Meditation (SSM) in people living with chronic pain and depression.

**Methodology**: We conducted a randomized controlled trial comparing SSM with the Health Enhancement Program (HEP) in people living with chronic pain and moderate depressive symptoms. Patients were recruited from multiple primary to tertiary care sites in the Greater Toronto Area. Both 12-week programs were provided virtually in groups, SSM by certified meditation teachers, and HEP by trained healthcare professionals. Depressive symptoms (primary outcome) were assessed using the Patient Health Questionnaire (PHQ-9) at baseline, and 12 and 24 weeks later (FU1 and FU2). We calculated within- and between-group changes in PHQ-9 scores, adjusting for potential confounders (including age, gender, and ethnicity). A Generalized Estimation Equation (GEE) method was used to adjust for repeated measures.

**Results**: Of 108 participants enrolled, 89 were randomized to the SSM (*n* = 43) or HEP (*n* = 46) group. Significant within-group mean difference in PHQ-9 scores from baseline were found for the SSM group at both FU1 [3.92 (95% CI 2.06, 5.78), *p* ≤ .001] and FU2 [4.75 (95% CI 2.44, 7.07), *p* ≤ .001]. Mean difference for HEP were significant only at FU1 [2.38 (95% CI 0.44, 4.31), *p* = .017]. Between-group mean differences were not significant.

**Discussion/Conclusions**: The SSM group demonstrated prolonged statistically significant improvements in depressive symptoms. Future studies are needed to assess implementation of SSM in clinical.

**Changes in pain following bilateral intermittent theta-burst, transcranial magnetic stimulation for depression: A retrospective chart review**

Sawmmiya Kirupaharan^a^, Roumen Milev^b, d^, Joanne Bresee^d^, Sonya Kelso^d^, Scott Duggan^a, d^, Felicia Iftene^b, c^, Tim Salomons^e^, Wilma Hopman^d, f^ and Ian Gilron^a, c, d^

^a^Queen’s University, Department of Anesthesiology and Perioperative Medicine, ^b^Queen’s University, Department of Psychiatry, ^c^Providence Care Hospital, ^d^Kingston Health Sciences Center Chronic Pain Clinic, ^e^Queen’s University, Department of Psychology, ^f^Queen’s University, Department of Public Health Sciences

**Introduction/Aim**: Pain management in patients with chronic pain and comorbid depression is challenging and understudied. There is growing interest in studying intermittent theta-burst stimulation (iTBS), an emerging protocol for repetitive transcranial magnetic stimulation (rTMS), for the treatment of chronic pain and comorbid depression. This retrospective review describes changes in pain, anxiety and depression throughout iTBS treatment at the dorsolateral prefrontal cortex (DLPFC).

**Methodology**: A retrospective chart review was conducted of patients who underwent their first acute series of iTBS treatments at the DLPFC for depression at a single institution between 2020 and 2023. Data on depression, anxiety and pain were collected throughout iTBS treatment using the Beck Depression Inventory-II (BDI-II, higher scores indicate worse depression) and Visual Analogue Scale (VAS, 0–100, higher scores indicate worse pain, anxiety and depression).

**Results**: Of 104 patients, 52 (50.0%) reported moderate pain at baseline. Median BDI-II scores decreased from 38.0 [IQR 29.0–44.0] to 24.0 [IQR 9.0–36.0] from pre- to posttreatment (*p* <.001). Patients reported a median pretreatment VAS pain score of 41.0 [IQR 5.5–72.0] and a posttreatment score of 16.5 [IQR 4.5–5.3] (*p* = .04). In patients with at least moderate pain at baseline, pain scores decreased from 71.0 [IQR 55.0–80.0] to 20.0 [IQR 11.0–71.0] (*p* = .004). Ten of 32 patients (31.2%) with available pre- and posttreatment scores reported ≥30% reduction in pain scores.

**Discussion/Conclusions**: These preliminary results, suggesting decreases in pain following iTBS treatment, provide a rationale for future rigorous investigations to evaluate this intervention for depression and comorbid chronic pain.

**Parents’ use of pediatric procedural pain management strategies and health seeking behavior**

Justine Dol^a^, Meredith Otley^a, b^, Jennifer Parker^a^ and Christine Chambers^a, c, d^

^a^IWK Health Center, Center for Pediatric Pain Research, Halifax, NS Canada, ^b^Dalhousie University, Faculty of Medicine, Halifax, Canada, ^c^Dalhousie University, Department of Psychology and Neuroscience, Halifax, Canada, ^d^Dalhousie University, Department of Pediatrics, Halifax, Canada

**Introduction/Aim**: Parents are often present during childhood painful procedures and can provide and advocate for effective pain management. However, most parents are unaware of effective pain management strategies. The objective of this study is to explore (1) which pain management strategies parents engaged in and (2) where parents are seeking pain management health information, in relation to their child’s most recent painful procedure.

**Methodology**: A cross-sectional study of Canadian parents with a child between the ages of 0 and 17 years was conducted through an online survey platform. Parents were asked questions related to childhood procedural pain management behavior and needs.

**Results**: Among 110 respondents, 55% were female and 51% of the children were female. The top three strategies used during their child’s last painful procedures were: encouraging deep breathing (51%), distraction (47%), and reassuring child (47%). Many respondents (41%) did not seek out information to help manage their child’s last painful procedures. Of those who did (59%), they were most likely to use a healthcare provider (33%) and/or the Internet (15%). On a scale from 1 (not at all) to 5 (completely), parents rated available information as helpful (*M* = 4.1, *SD* = 0.9).

**Discussion/Conclusions**: Despite almost half of parents not seeking out health information related to their child’s most recent painful procedure, findings suggest that parents would still benefit from evidence-based information. Specifically, almost half of all parents provided their child reassurance, which has been linked to increases in children’s experience of pain. Evidence-based tools are needed to enhance parents’ knowledge around childhood procedural pain management.

**Cardiac and behavioral trajectories of pain-responding across the second year of life**

Sara Jasim^a^, Haleh Hashemi^a^, Dan Flanders^b^, Eitan Weinberg^b^, Deena Savlov^b^, Hartley Garfield^b^ and Rebecca Pillai Riddell^a, b, c^

^a^York University, ^b^University of Toronto, ^c^The Hospital for Sick Children

**Introduction/Aim**: To manage needle-related pain in young children, it is important to learn about pain-responding based on developmental stage. As the brain undergoes rapid changes early in life, children gain regulatory skills and may experience pain modulation. Thus, this study will assess developmental patterns in pain trajectories during routine immunizations across the second year of life via cardiac and behavioral measures on pain-related distress.

**Methodology**: Respiratory Sinus Arrhythmia (RSA) and Face, Legs, Activity, Crying, and Consolability (FLACC) scores were collected at 4 immunization appointment timepoints (Base, Needle, Post1, and Post2) during toddler’s 12-, 18-, and 24-month visits (*N* = 223). Multilevel modeling was used to examine age-related changes in RSA and FLACC trajectories.

**Results**: No RSA changes were found across ages. FLACC trajectories differed by age. Specifically, between 12 and 18 months, significant slope differences were found between Base and Needle (= −2.02, *p* < .001) and Post1 and Post2 (= 0.90, *p* = .01). Between 12 and 24 months, significant slope differences were found between Base and Needle (= −1.11, *p* = .02).

**Discussion/Conclusions**: Cardiac responses to pain did not change with age. However, behavioral pain responses revealed that greater reactivity to pain at 12 months becomes less pronounced over time and rate of recovery to baseline levels increases. By 18 months, pain behaviors return to baseline levels by the third minute whereas more time is required at 12 months. Thus, with increasing age, infants’ reactivity to pain decreases and their recovery rate of change increases, suggesting better-developed regulatory skills.

**The effect of targeted phantom motor execution on phantom limb pain in people with ‎unilateral transtibial amputation**

Kamiar Ghoseiri^a^ and Audrey Zucker-Levin^a^

^a^School of Rehabilitation Science, College of Medicine, University of Saskatchewan, Saskatoon, SK, Canada

**Introduction/Aim**: Phantom limb pain (PLP) is prevalent among individuals with limb amputation, negatively impacting quality of life and function. It has been hypothesized that actively exercising the phantom limb could decrease painful perception and enhance prosthetic control, ultimately improving overall physical function. Our study investigated the impact of a specific phantom motor execution program on PLP in individuals with unilateral transtibial amputation (uTTA).

**Methodology**: Ethics approval and informed consent was obtained prior to data collection. Participants were recruited from a free-standing prosthetic clinic and were randomized into one of two groups: exercise or control. The exercise group underwent three weeks of targeted phantom motor execution training, while the control group received no intervention. PLP was assessed in both groups at baseline and follow-up using the Survey of Phantom Limb Awareness and Control. Chi-square tests were used to compare results within and between groups.

**Results**: Twenty-three participants with uTTA aged 59.1 (±12.3) years, 16 males, participated. Participants in the exercise group (*n* = 13) experienced reduced PLP (X^2^ = 3.74, *p* = .05); no change was reported in the control group (*X*^2^ = 1.351, *p* = .245). There was no significant difference in PLP between exercise and control groups at the baseline (*X*^2^ = 0.087, *p* = .768) and follow-up (*X*^2^ = 0.787, *p* = .375).

**Discussion/Conclusions**: Targeted phantom motor execution training reduced PLP in individuals with uTTA. Long-term follow-up and a larger sample size are recommended to deepen understanding of the program’s efficacy and potential applications. Further, the development of a standardized, valid, and reliable outcome measure for measuring PLP is warranted.

**Living in a (Sometimes) painful body: Chronic pain as a dynamic experience and implications for treatment**

Leigha Comer

Arthur Labatt Family School of Nursing, Western University

**Introduction/Aim**: For many people with chronic pain, the pain they live with is experienced cyclically, periodically, or only occasionally. To better understand the lived experiences of people with chronic pain, there is an urgent need to attend to the experience of living in a body that is struck by pain intermittently and, often, unpredictably.

**Methodology**: This study draws on interviews with twenty-one people with chronic pain and analyzes lived experiences of chronic pain through the lens of critical disability studies. In particular, the concept of “dynamic disability” is taken up to explore chronic pain as it is experienced intermittently and unpredictably.

**Results**: For every person interviewed, the experience of pain was highly dynamic. The inconsistent nature of pain impacted their sense of self, as many struggled to determine whether they could “legitimately” identify as disabled. The unpredictability of chronic pain further complicated their engagement in activities related to paid work, caregiving, and so on. This inconsistency also troubled their ability to access care, as appointments with specialists and pain clinics were often made months in advance, and sufferers could not predict whether their symptoms would be present at the time of the appointment. Likewise, some people found that medication regimes did not account for the variable nature of their symptoms.

**Discussion/Conclusions**: The notion of chronic pain as a “dynamic experience” might allow for a more fulsome conceptualization of the experience of living in a (sometimes) painful body. Furthermore, the recognition of chronic pain as highly dynamic raises important questions for diagnosis and treatment.

**A survey of knowledge, attitudes, and behaviors of Canadian family physicians in response to the Canadian Guideline on Prescribing Opioids for Chronic Noncancer Pain (CNCP): Preliminary results**

Lillian Saberian^a, b^, Nagina Parmar ^a^, Mohammad Alavinia ^a^, Jenny Lau ^b, c, d^, Craig M. Dale^e, f, g^, Peter Selby^c, h^, Lydia Hatcher ^i^, Angela Carol ^i, j^, Najam Mian ^k^, Nicole Bootsman ^l^, Fizza Gilani ^m^ and Andrea Furlan^a, b, n^

^a^KITE-Toronto Rehabilitation Institute- University Health Network, ^b^Institute of Medical Science- University of Toronto, ^c^Department of Family and Community Medicine- University of Toronto, ^d^Princess Margaret Cancer Center- University Health Network, ^e^Lawrence S. Bloomberg Faculty of Nursing- University of Toronto, ^f^Interdepartmental Division of Critical Care Medicine- University of Toronto, ^g^Sunnybrook Health Sciences Center, ^h^Centre for Addiction and Mental Health, ^i^Department of Family Medicine- McMaster University, ^j^Hamilton Urban Core Community Health Center, ^k^Department of Medicine-University of British Columbia, ^l^College of Physicians and Surgeons of Saskatchewan, ^m^College of Physicians and Surgeons of Alberta, ^n^Department of Medicine, University of Toronto 

**Introduction/Aim**: We are currently conducting the 3^rd^ Canadian Survey of Family Physicians (FPs) practices and knowledge in prescribing opioids for chronic noncancer pain (CNCP). We aim to assess changes from the previous survey conducted in 2018.

**Methodology**: We invite FPs from all Canadian provinces and territories to participate in this cross-sectional online survey in English and French from July 2023 to January 2024. The survey contains questions about screening for eligibility (4), demographic characteristics (12), knowledge about opioids and CNCP (14), behaviors (30), and attitudes (38).

**Results**: From July to November 2023, there were 295 responses, with a 73% completion rate; most respondents were from Ontario (58%). The percentage of physicians not prescribing opioids for CNCP has increased from 11% in 2018 to 25% in 2023. Concerns about long-term adverse effects are a primary factor influencing the decision not to prescribe opioids (from 96% to 86.6%). The percentage of respondents who are “Very Confident” in prescribing opioids for CNCP declined from 16% to 12%. Canadian FPs’ adherence to guidelines shows a modest decline; notably, urine drug screening dropped by 13% before initiating and 17% during monitoring of opioid therapy in 2023 compared to 2018.

**Discussion/Conclusions**: The significant surge in FPs choosing not to prescribe opioids for CNCP, driven by enduring concerns about long-term adverse effects, could be a response to the ongoing opioid crisis. Concurrently, evolving opioid prescribing practices contribute to a subtle decrease in confidence among respondents. Strategies like targeted education may enhance guideline adherence, emphasizing the need for ongoing assessment to optimize patient care.

**An interpretive synthesis of patient engagement frameworks**

Leigha Comer^a^, Laura Connoy^a^, Megan Harley^a^ and Fiona Webster^a^

^a^Arthur Labatt Family School of Nursing, Western University

**Introduction/Aim**: Patient engagement (PE) has been promoted as a collaborative approach that involves patients in the research process. A number of frameworks have emerged in the literature with the aim of guiding PE. However, little is known about what models exist to guide researchers engaging patients in research, or what conceptualizations of PE underpin these frameworks.

**Methodology**: Drawing on the work of Dixon and colleagues, we conducted an interpretive synthesis of the literature to synthesize arguments in the literature, reveal the larger structures in which PE has been produced and in which it is embedded, and identify the assumptions underlying PE. This involved purposive sampling of the literature, the use of a data extraction table, and discussions through which analytical questions were considered.

**Results**: Our preliminary findings indicate that PE and associated frameworks are underpinned by assumptions regarding the role of researchers (e.g., as those who invite patients to the research, rather than vice versa), the value of PE (which is rarely explored or justified), conceptions of patients (e.g., as partners or as stakeholders), and connections between PE and evidence-based medicine.

**Discussion/Conclusions**: An interpretive synthesis of the literature allowed for the production of a critical analysis that identified assumptions about PE that are largely unrecognized or unquestioned. Our findings point toward the need for further exploration of the history of PE, assumptions embedded in how the process of engagement is described, and beliefs regarding the value of PE. Our work will continue to extend this line of inquiry.

**Subjective salience ratings are a reliable surrogate for physiological measures of arousal**

Georgia Hadjis^a, b^, Reem Mustafa^a, b^, Lauren Atlas^c, d^ and Massieh Moayedi^a, b^

^a^University of Toronto, ^b^Centre for Multimodal Sensorimotor and Pain Research, ^c^National Center for Complementary and Integrative Health, ^d^National Institutes of Health

**Introduction/Aim**: Behavioral and neuroimaging studies have long investigated what makes pain unique compared to other nonpainful aversive stimuli. Pain is often matched with auditory, visual, and/or somatosensory stimuli across many rating scales, namely salience, or attention-grabbing quality, to control for general aspects of arousal. Skin conductance responses (SCRs) are changes in the electrical conductivity in the skin in response to a stimulus and are a proxy of physiological arousal. The reliability of subjective salience ratings has not been tested compared to SCRs, and it remains unclear whether subjective ratings alone can in fact rule out confounds of arousal. Here, we investigated whether heat pain and innocuous somatosensory electric stimuli matched subjectively across multiple salience levels also produced matched physiological responses using SCRs.

**Methodology**: Healthy participants from the university environment provided informed consent to experience six levels of salient heat pain and electric stimuli calibrated to their sensitivity while their SCRs were recorded. Physiological data were collected and analyzed using BIOPAC Acknowledge 5.0 to obtain an area under the curve (AUC) for every trial. Average salience ratings and AUC for each salience level were compared across stimuli within subjects using a linear regression.

**Results**: The slopes and intercepts of AUCs did not differ between pain and electric stimuli across the salience levels (*p* > .1).

**Discussion/Conclusions**: Subjective salience ratings are reliable surrogates for a physiological measure of arousal to compare noxious and innocuous somatosensory stimuli, as ratings and their corresponding physiological responses do not differ. This method allows future studies to rule out general arousal as a confound.

**Cyroablation as a treatment for symptomatic Bertolotti’s syndrome: A case report**

Abeer Alomari^a^, Ken-Yi Lui^a^ and Philip Peng^a^

^a^Toronto Western Hospital

**Introduction/Aim**: Bertolotti’s syndrome is characterized by chronic lower back pain caused by transitional lumbosacral vertebrae with a reported incidence of 4–36%. Initial management is usually conservative including physical therapy and medical management. Should conservative management fail, surgical treatments are the mainstay of management. Intervention such as radiofrequency (RF) ablation may have a role but is rarely reported.

**Methodology**: Case description.

**Results**: A 44-year-old female with a 25-year history of intermittent lower back pain, which progressively worsened over the last 5 years, was referred to the pain clinic. CT scan confirmed the diagnosis of Bertolotti syndrome with partial sacralization of left L5 transverse process. A diagnostic block was performed, and complete pain relief lasted for a few hours. Subsequently, a radiofrequency ablation of the left iliolumbar ligament was performed but pain relief only lasted for a week. A cryoablation was performed and she reported initial flare-up pain for a few weeks and significant improvement for 4 months before gradually returning to baseline levels. A second cryoablation with a different approach, with the tip being directed over and below the iliolumbar ligament and the junction between the ligament and the ilium. This time she reported almost instant complete pain relief postprocedure and did not experience postprocedure flareup. The last follow-up was 8 weeks postprocedure, and she remains pain-free.

**Discussion/Conclusions**: There has not been any reported use of cryoablation for the management of symptomatic Bertolotti’s syndrome and we suggest that cryoablation is an effective option in cases not responding to RF ablation. Further investigation of this technique is warranted.

**Polytrauma and spinal cord injury: A novel mouse model for chronic pain research**

Gopeekrishnan Unnithan Radhakrishnan Unnithan^a, b^, Joshua Hazan Mea^b^, Tarek Klaylat^b, c^, Rahul Gawri^b, c, d^ and Chan Gao^a, b, c, d^

^a^Departments of Medicine, McGill University, Montreal, QC, CA, ^b^Faculty of Medicine and Health Science, McGill University, Montreal, QC, CA, ^c^Departments of Surgery, McGill University, Montreal, QC, CA, ^d^Research Institute – McGill University Health Center, Montreal, QC, CA

**Introduction/Aim**: Over 50% of the patients with spinal cord injury (SCI) have chronic pain. Our lab developed a new mouse model with simultaneous SCI and muscle-tendon injuries (SCI+MTI) and this model is being characterized as a model of SCI-associated chronic pain. We aim to determine the postoperative analgesic regimen optimized for pain-related behaviors assessment in this SCI+MTI model.

**Methodology**: Ten 12-week-old female C57B/L6 mice underwent spinal cord contusion injury at T9/10 followed by left quadriceps crush and tenotomy. They were then randomized to either 0.5 mg/kg or 1.0 mg/kg of BUP SR every 3 days until postoperative day 24. Pain-related assessments were conducted at baseline and every 6 days until the 28-day endpoint.

**Results**: Eight mice were included for analysis after excluding 2 mice due to human endpoints. Treatment with Bup SR at 0.5 mg/kg led to a trend of higher-level acute stress compared to 1.0 mg/kg as shown by facial expression and body language score. No significant differences were observed in Von Frey and acetone-evoked behavior tests between injured (left) and uninjured (right) limbs in either treatment group from days 6 to 24. On day 28, injured (left) limbs showed mechanical and cold allodynia in both treatment groups.

**Discussion/Conclusions**: Administration of 0.5 mg/kg BUP SR did not improve the sensitivity of evoked pain assessment. Evoked pain analysis should be conducted after allowing the resolution of acute painful stress and cessation of postoperative analgesic treatment This SCI+MTI mouse model is a useful tool for future research on SCI-associated pain.

**Acceptabilité culturelle des instruments de mesure de la douleur ? Entrevues cognitives auprès de personnes immigrantes au Québec**

Oumar Mallé Samb^a^, Emilie Gélinas^a^, Marimée Godbout-Parent^a^, Claudie Audet^a^, Joséanne Desrosiers^a^, Anaïs Lacasse^a^, Nancy Julien^a 1034^and Oscar Labra^a^

^a^Université du Québec en Abitibi-Témiscamingue

**Introduction/Objectif**: Les instruments utilisés en clinique et en recherche pour mesurer la douleur n’ont pas tous été validés dans plusieurs contextes culturels. La culture est pourtant reconnue comme ayant un impact sur l’expérience de douleur. Cette étude visait à évaluer l’acceptabilité culturelle de quatre instruments de mesure communément utilisés dans le domaine de la douleur.

**Méthodologie**: Des entretiens cognitifs (entrevues individuelles qualitatives) ont été menés auprès de 14 personnes immigrantes vivant avec de la douleur depuis plus de 3 mois et arrivées au Québec depuis moins de 5 ans (Algérie, Cameroun France, Guinée, Sénégal, Tunisie, Haïti). L’entretien cognitif permet d’analyzer les processus cognitifs impliqués dans la formulation d’une réponse (compréhension/récupération/jugement/réponse) ainsi que leur précision. Trois échelles d’intensité de la douleur (échelle de visages FPS-R, échelle numérique 0–10, échelle visuelle analogique), ainsi que l’échelle d’impression globale de changement ont été testées.

**Résultats**: De manière générale, les instruments étaient acceptables culturellement. S’ils étaient la plupart du temps bien compris, des personnes ont toutefois soulevé des problèmes de compréhension, de récupération et de jugement pouvant mener à des réponses imprécises. Contrairement à nos attentes, c’est l’échelle numérique qui a été la préférée pour sa simplicité et son côté pratique.

**Discussion/Conclusions**: Les instruments, bien qu’en général acceptés, ont fait l’objet de critiques quant à leur pertinence en contexte de chronicité, notamment à l’égard du manque de considération pour les facteurs contextuels (ex. normes sociales, médicaments). Comme ces facteurs ont de l’importance dans l’expérience de la douleur, il est nécessaire d’inclure ceux-ci lors de l’évaluation.

**Healthcare professionals’ perceptions of trauma-informed care in the context of chronic pain across Canada**

Catherine Paré^a^, Arthur Woznowski-Vu^b^, Nathan Augeard^b^, Geoff Bostick^c^, Melanie Noel^d, e^, Allen Steverman^f^, Peter Stilwell^b^, Sandra Woods^g^ and Timothy H. Wideman^b^

^a^Department of Psychology, McGill University, ^b^School of Physical and Occupational Therapy, McGill University, ^c^Department of Physical Therapy, University of Alberta, ^d^Department of Psychology, University of Calgary, ^e^Alberta Children’s Hospital Research Institute, ^f^Le Center de Gestion de la Douleur, Center Hospitalier de l’Université de Montréal (CHUM), ^g^Patient Partner

**Introduction/Aim**: A history of trauma is common in people living with chronic pain and can limit the effectiveness of chronic pain treatment. Although TIC was endorsed in the Canadian Pain Task Force’s 2021 Action Plan, perceptions on TIC remain unclear for healthcare professionals (HCP) working with chronic pain patients. As such, this study explored HCP knowledge, attitudes, behaviors, and competencies in relation to TIC.

**Methodology**: An anonymous, cross-sectional survey (French and English) was conducted using a convenience sample of individuals self-reporting as licensed HCP working ≥30% of their clinical hours with chronic pain patients. Descriptive, missing data, and inductive content analyses were conducted.

**Results**: The majority of participants (*n* = 30/59) were HCP in chiropractic and physiotherapy. Although most participants defined trauma as a psychological or multidimensional construct, some (*n* = 6) provided a biomedical definition of trauma. Participants’ definitions of TIC were varied, touching on the comprehensive nature of this approach, the multifaceted impact of trauma on a person’s well-being, and the interpersonal effect of TIC. Over 65% of participants were knowledgeable about, had positive attitudes toward, felt proficient in applying, and had recently used TIC principles. Notably, a systematic missing data pattern was identified: questions unrelated to TIC had 0–5% missing data, compared to 30–40% missing data for TIC-related questions.

**Discussion/Conclusions**: Although findings suggest a high degree of familiarity of Canadian HCP with TIC, the pattern of missing data suggests that the sample may have been biased toward HCP who endorse TIC. This has important implication for future research and knowledge translation initiatives on TIC.

**New-onset chronic and neuropathic pain in survivors of severe COVID-19: A secondary analysis of the PAIN-COVID trial**

Antonio Ojeda^a, b^, Tomas Cuñat^a^, Marylin Arias^a^, Oscar Comino^a^, Jorge Aliaga^a^ and Andrea Calvo^a^

^a^Department of Anesthesiology, Critical Care and Pain Medicine, Hospital Clínic de Barcelona, University of Barcelona, Spain., ^b^Alan Edwards Pain Management Unit. McGill University Health Center. Montreal. Canada.

**Introduction/Aim**: Survivors of critical illnesses, particularly COVID-19, often endure chronic pain post-ICU discharge. This study examines PAIN-COVID trial data to assess the progression of new-onset and neuropathic pain 6 months after discharge, evaluating pain intensity, functional impact, and quality of life compared to patients without such pain.

**Methodology**: We analyzed the prevalence and impact of new-onset and neuropathic pain in COVID-19 ICU survivors 6 months postdischarge, using propensity score matching to control for confounding risk factors. Pain intensity, functional impact, and quality of life were compared between groups.

**Results**: At 6 months, 86 severe COVID-19 survivors were evaluated, with 47.6% reporting new-onset pain. In matched cohorts, those with new-onset pain showed a significant decrease in quality of life, with EQ 5D/5 L index values at 0.705 [0.613 to 0.818] (*p* <.001) and EQ 5D/5 L VAS scores at 72.0 [60 to 80] (*p* = .019) compared to those without pain. Neuropathic pain prevalence was 36.5%, and the neuropathic pain group exhibited higher BPI-intensity and BPI-limitation scores (5.3 [2.5 to 6] (*p* = .002) and 4.5 [1.5 to 6.9] (*p* = .025), respectively). Quality of life assessments using the EQ 5D/5 L index were lower for the neuropathic pain group (0.68 [0.36 to 0.81], *p*= .29), with VAS scores also lower (60 [47.5 to 85], *p* = .032).

**Discussion/Conclusions**: Post-COVID-19 ICU survivors experience significant new-onset and neuropathic pain, markedly impacting their quality of life.

**Resources informed by women’s experiences with chronic pain and prescription opioids: Translating research to practice**

Olivia Schultz^a^, Lindsay Wolfson^a^, Nancy Poole^a^, Darby Whittaker^a^, Andreea Brabete^a^ and Lorraine Greaves^a^

^a^Centre of Excellence for Women’s Health

**Introduction/Aim**: Women report more chronic pain than men and are more likely to be prescribed opioids for pain management. Despite this, it is not well documented how women’s specific sex/gender experiences with prescription opioid use for pain management impact policy and practice. The Center of Excellence for Women’s Health led a two-year project to create sex, gender, and equity-informed resources for 1) service providers, educators, and policymakers and 2) women who experience chronic pain.

**Methodology**: A scoping review, a comprehensive literature review, and qualitative interviews were conducted. The reviews focused on 1) women’s experiences with prescription opioids for chronic pain and 2) the implications of sex/gender factors on chronic pain and efficacious treatment interventions. Twenty-two women from BC, NWT, and Yukon were interviewed to understand their lived experiences and information needs.

**Results**: The findings have been translated into a digital guide, infographics, information sheets, and discussion guides. These resources emphasize the need for codeveloped comprehensive pain management plans and for healthcare providers and policymakers to have better operational understanding of the benefits and harms of opioid use for women living with chronic pain. Resources developed for women respond to their expressed desire for greater information for themselves and their providers, such as understanding what opioids are, how they may affect women and alternative pain management strategies.

**Discussion/Conclusions**: Women have sex/gender-specific experiences with chronic pain. There is an urgent need for evidence-based policy, practice, and resources that are responsive to the lived realities of women with chronic pain in a nonstigmatizing and harm-reducing manner.

**Understanding the meanings of pain among immigrant individuals living with chronic pain**

Joséanne Desrosiers^a^, Oumar Mallé Samb^a^, Émilie Gélinas^a^, Marimée Godbout-Parent^a^, Claudie Audet^a^, Anaïs Lacasse^a^, Oscar Labra^a^ and Nancy Julien^a^

^a^Université du Québec en Abitibi-Témiscamingue

**Introduction/Aim**: In Canada, one person out of four is or has been a landed immigrant or a permanent resident. From 2016 to 2021, new immigrants who settled permanently in Canada represented just over 1.3 million people. Knowing that chronic pain disproportionately affects certain ethnic and racialized communities, and that culture can potentially influence the conceptualization and expression of pain, exploring the cultural meanings of pain among the immigrant population seems relevant to ensure equitable approaches and awareness in chronic pain management. This project was part of a larger qualitative study [multiple case study] aimed at evaluating the cultural relevance of pain measurement instruments. One of the objectives was to gain a better understanding into how immigrants describe pain in their respective culture.

**Methodology**: We conducted individual qualitative interviews with 14 immigrants living with chronic pain in the province of Quebec, who arrived in Canada less than 5 years ago. All discussions were audio-recorded and verbatim transcriptions were analyzed using a thematic analysis, with NVivo15© software for coding support.

**Results**: Regardless of their cultural backgrounds, immigrant individuals in the study shared a similar conceptualization of pain. They described that pain is an individual and subjective experience that can manifest both physically and psychologically. However, differences were found in the ways pain is communicated to others.

**Discussion/Conclusions**: Our findings suggest that the expression of one’s pain varies by cultural backgrounds. Clinicians should be mindful of these nuances for equitable care.

**Week-to-week reports of physical and cognitive side effects associated with opioids: Insights from a longitudinal study among persons with chronic pain**

Amanda Sirois^a, b^, Maria Verner^b^, Alice Bruneau^c^, Jiaqi Bi^d^, Jordi Perez^b, e^, Yoram Shir^b, e^ and Marc O. Martel^a, b, e^

^a^Faculty of Dental Medicine and Oral Health Sciences, McGill University, Montreal, Canada, ^b^Alan Edwards Pain Management Unit, McGill University Health Center, Montreal, Canada, ^c^Division of Experimental Medicine, McGill University, Montreal, Canada, ^d^Department of Epidemiology and Biostatistics, Schulich School of Medicine and Dentistry, Western University, London, Canada, ^e^Department of Anesthesia, McGill University, Montreal, Canada

**Introduction/Aim**: Despite the potential benefits of opioids, their regular use can be accompanied by side effects. It is unclear if side effects remain stable or whether they fluctuate over time among those on long-term opioid therapy. The first objective was to examine the degree of within-person fluctuations in opioid-related side effects, on a week-to-week basis for 5 consecutive weeks, among persons with chronic pain on long-term opioid therapy. We also examined if fluctuations in pain intensity and negative affect were linked to weekly reports of physical and cognitive side effects.

**Methodology**: Participants (*n* = 77) underwent baseline assessment and completed daily diaries for 14 consecutive days assessing pain, psychological, and opioid-related variables. Weekly reports were then collected over 5 consecutive weeks to assess the same variable domains, including reports of opioid-related physical and cognitive side effects.

**Results**: Multilevel analyses revealed significant within-person (week-to-week) variability in physical and cognitive side effects (both *p* < .05). Across weeks, higher levels of pain intensity and negative affect were linked to greater reports of physical and cognitive side effects (all *p* < .05).

**Discussion/Conclusions**: Our findings provide information on the stability/fluctuations in opioid-related side effects among persons with chronic pain. Our findings revealed considerable within-person fluctuations in physical and cognitive side effects associated with opioids, and opioid-related side effects were more pronounced on weeks when patients concurrently experienced higher levels of pain and negative affect. Our findings could point to ways of reducing opioid-related side effects among those using opioids for pain.

**Quantitative sensory testing and exercise-induced hypoalgesia protocols in low back pain: A scoping review**

Lee-Ran Goodman^a^, Ronessa Dass^a^, Eden Daniel^a^, Shirin Modarresi^a^, Lisa Carlesso^a^, Ada Tang^a^ and Luciana Macedo^a^

^a^School of Rehabilitation Sciences, Faculty of Health Sciences, McMaster University

**Introduction/Aim**: There are inconsistencies in the way that quantitative sensory tests (QST) have been implemented across the low back pain literature. Given the broad range of ways that QST have been implemented in practice, a synthesis of the literature is warranted. The objective of this scoping review was to summarize methodologies used to assess endogenous pain modulation (EPM) using QST (pain pressure threshold (PPT), temporal summation (TS), conditioned pain modulation (CPM)) or exercise-induced hypoalgesia (EIH) in adults with low back pain.  

**Methodology**: Databases Medline, Embase, CINAHL and AMED were searched on June 15, 2023, for articles that used QST or EIH protocols in back pain populations. Title and abstract screening, full text evaluation, and data extraction (participants, study design, setting, details on QST and EIH protocols) were performed by a pair of independent reviewers.

**Results**: Of the 194 studies included in the review, 173 used PPT, 54 used TS and 53 used CPM; only 5 studies investigated EIH. There was high variability in the type of equipment, timing, trials, and testing location for all QST measurements with many studies not reporting necessary details for replication.  

**Discussion/Conclusions**: This scoping review provides a summary of the most common protocols for QST and EIH in low back pain that may be used as a guide for assessment in future studies. However, these results demonstrate a need for the development of standardized protocols and reporting guidelines. 

**Mapping out marginalization within chronic pain scholarship: A scoping review**

Laura Connoy^a^, Michelle Solomon^a^, Riana Longo^a^, Joel Katz^b^, Abhimanyu Sud^c^, Craig Dale^c^ and Fiona Webster^a^

^a^University of Western Ontario; ^b^York University; ^c^University of Toronto

**Introduction/Aim**: Chronic pain scholarship is witnessing a shift toward equity, diversity, and inclusion (EDI) frameworks. Through these frameworks, researchers will be able to better account for the needs and knowledges of systemically and structurally marginalized groups of people living with chronic pain. We question how marginalization is accounted for, and how marginalized groups are discussed, within this scholarship.

**Methodology**: Guided by Arksey and O’Malley and Levac et al., and the PRISM-ScR checklist, we conducted a scoping review of scholarship focusing on chronic pain and marginalization. In total, 67 articles were included in our scoping review study.

**Results**: There were three primary purposes for the readings: 1) everyday experiences; 2) mental illness and/or substance use; and 3) health education, training, or pain management programs. Overall, we noted little representation of studies focusing on women, Indigenous, Black or People of Color. Furthermore, we noted varying considerations of social-political and socio-economic contexts; conceptual conflations between sex and gender; and differing approaches to how people living with chronic pain and marginalization were described.

**Discussion/Conclusions**: Our preliminary findings indicate that alongside the contributions being made by chronic pain scholarship toward EDI, there exist many fissures. From these, we offer the following suggestions in order to strengthen future research and work toward the adequate implementation of EDI in research: 1) engage with social science literature and critical scholarship to account for sociopolitical and socioeconomic contexts; 2) provide definitions and explanations of terminology, theoretical frameworks, and/or concepts; and, 3) engage in gender-based research that can also account for intersections of social categorizations.

**Clinical questions asked to addictions medicine providers by primary care providers through eConsult: A retrospective content analysis**

Grace Zhu^a^, Cynthia Chan^a^, Danica Goulet^b^, Erin Keely^b, c^, Clare Liddy^a, b, d^ and Arun Radhakrishnan^a, e^

^a^Department of Family Medicine, University of Ottawa, Ottawa, ON, Canada, ^b^Ontario eConsult Center of Excellence, The Ottawa Hospital, Ottawa, ON, Canada, ^c^Department of Medicine, University of Ottawa, Ottawa, ON, Canada, ^d^C.T. Lamont Primary Health Care Research Center, Bruyère Research Institute, Ottawa, ON, Canada, ^e^Bruyere Research Institute, Ottawa, ON, Canada

**Introduction/Aim**: Considering the opioid crisis and addiction prevalence of up to 50% among patients with chronic noncancer pain (CNCP), managing chronic pain in primary care has become increasingly complex. The Champlain Building Access to Specialists through eConsultation (BASE™) electronic consultation (eConsult) service allows primary care providers (PCPs) in the Champlain region to request rapid electronic support from specialists.

**Methodology**: To characterize major themes in clinical questions asked in Addiction Medicine and the PCP-perceived benefits of this service, we conducted content analysis of eConsult cases submitted to Addiction Medicine between January 1, 2020 to December 31, 2021. Descriptive statistics were generated for content and question type classification, and close-out survey responses by PCPs.

**Results**: Among 129 cases included in this study, PCPs mostly sought advice on opioid (38.0%) and alcohol use (34.9%). Many PCPs asked about pharmacological (75.5%) and nonpharmacological (65.1%) management for patients with substance use. Chronic pain (28.7%) and prescribing controlled substances (24.0%) were among the most common clinical topics discussed in these cases. Specialists answered within 7 calendar days in over 95% of eConsults. PCPs reported the responses were helpful, with nearly a third reporting avoiding a formal referral.

**Discussion/Conclusions**: Despite analyzing eConsults to Addiction Medicine, the most common questions were related to CNCP management with opioids. This finding may relate to challenges PCPs face in navigating opioid prescribing related to a focus on opioid harms (including addictions), regulatory policies and adherence to prescribing guidelines. However, support from eConsults helped reduce formal consultations related to questions around addictions and opioid prescribing for CNCP.

**Heme-Oxygenase-1 as a potential biomarker for chronic painful temporomandibular disorders**

Natalie Orlovetskie^a, b^, Berlant AlSabbagh^a, c^, Hyman M Schipper^a, b^, and Ana Velly^a, c, d^

^a^Lady Davis Institute for Medical Research, ^b^Department of Neurology and Neurosurgery, McGill University, ^c^Department of Dentistry, Jewish General Hospital, ^d^Faculty of Dentistry, McGill University

**Introduction/Aim**: Chronic Painful Temporomandibular disorders (PTMD) are a major public health problem, requiring the use of reliable biomarkers for accurate diagnosis, prognosis, and successful treatment. The potential of heme oxygenase-1 (HO-1) as a biomarker for chronic pain is investigated in this study, focusing on different age groups.

**Methodology**: Whole, unstimulated saliva samples were collected from three groups: chronic PTMD patients 65 and older (CP65+), chronic PTMD patients younger than 65 (CP65-), and healthy controls 65 and older (*n* =17/group). Extracellular vesicle (EV) particles were isolated using an EV purification kit, and HO-1 concentration and EV CD63 abundance were quantified with specific ELISA kits, enabling detailed analysis of HO-1 levels in various fractions. Analysis of variance (ANOVA) was used to compare the levels of the potential biomarker between groups (α = 0.05).

**Results**: CP65+ patients had significantly higher levels of HO-1 in fractions (Free, L1Cam, Glast, NLG) compared to controls (*p* < .005), and greater levels in fractions (Free, L1Cam, and Glast) fractions compared to CP65- (*p* < .004). In all fractions, CP patients had a greater HO-1/CD63 ratio (*p* < .03) than controls. Significantly elevated HO-1 levels were detected in the Free fraction of the CP65- group compared to other groups (*p* = .0002).

**Discussion/Conclusions**: The results show that HO-1 has the potential to be a useful biomarker for chronic PTMD, particularly in older individuals. The presence of higher HO-1 levels in certain fractions suggests that it may be a useful biomarker for diagnostic, prognostic, and therapeutic approaches in pain management.

**Autobiographical memory in acute perisurgical pain in youth undergoing spinal fusion**

Anna Waisman^a^, Maria Pavlova^b^, Chenyue Zhang^a^, Valeria Robles Vera^b^, Batsheva Weinberger^a^, Carmel Daskalo^a^, Melanie Noel^b^ and Joel Katz^a, c^

^a^York University, ^b^University of Calgary, ^c^Toronto General Hospital

**Introduction/Aim**: Young children who remember more emotions related to past surgery recall pain as less distressing. The specificity with which individuals recall pain-related events also predicts postsurgical pain up to 1 year later. Autobiographical memory for pain hasn’t been assessed in youth. The aim was to investigate the relationship between perisurgical pain and memory in youth.

**Methodology**: Following REB approval and informed assent/consent, 38 youth (10–18 years) scheduled for spinal fusion at Alberta Children’s Hospital rated their pain intensity using a 0–10 point NRS for 7 consecutive days before and 3 consecutive days after surgery. Memory interviews were conducted 3–4 weeks postsurgery when patients recalled their experiences on the first day after surgery. Interviews were coded for specificity. Details were further categorized into content categories of pain; medical; body; coping; anxiety and fear; meaning-making details; and positive, negative, and neutral emotion.

**Results**: Multiple linear regression analyses were conducted using postsurgical pain as the independent variable, presurgical NRS pain as a covariate, and episodic/semantic memory content categories as the dependent variable. Pain was not a significant predictor of memory specificity overall (*p*>.1). Presurgical pain significantly predicted the proportion of episodic coping details β = −0.01, *SE* = 0.01, *t*(33) = −2.10, *p*= .04 and anxiety/fear recalled 3–4 weeks after surgery, β = 0.02, *SE* = 0.01, *t*(33) = 2.31, *p*= .03. Postsurgical pain significantly predicted the proportion of episodic meaning-making details 3–4 weeks after surgery, β = −0.03, *SE* = 0.02, *t*(33) = −2.21, *p*= .03.

**Discussion/Conclusions**: Findings indicate that pre- and postsurgical pain intensity predicts youth’s surgery-related memory content 3–4 weeks later. Perisurgical pain predicts certain episodic but not semantic content.

**Comfort level of Quebec prescribers with dispensing and adjusting prescriptions for the treatment of chronic pain: A cross-sectional study about associated factors**

Usra Naeem^a, b^, Gwenaelle De Clifford-Faugère^a^, Marimée Godbout-Parent^a^, Hermine Lore Nguena Nguefack^a^ and Anaïs Lacasse^a^

^a^The Université du Québec en Abitibi-Témiscamingue, ^b^The University of Lahore, Lahore, Pakistan

**Introduction/Aim**: Identifying factors associated with the comfort level to prescribe medications is important for tailoring education and training. The objective of this study was to explore factors associated with the comfort level of healthcare professionals regarding dispensing and adjusting prescriptions for the treatment of chronic pain (CP).

**Methodology**: A cross-sectional survey was conducted among licensed physicians, pharmacists,

and nurse practitioners across the province of Quebec. Comfort level regarding dispensing and/or adjusting prescriptions for the treatment of CP was measured on a 0–10 rating scale (0 =very uncomfortable, 10 =very comfortable).

**Results**: 207 prescribers participated (83 physicians, 58 pharmacists, 66 nurse practitioners). Mean comfort level score was 6.10 ± 1.8 (median: 6). Differences in scores were found between physicians (6.4 ± 1.8), pharmacists (6.6 ± 1.8) and nurses (5.1 ± 1.8; *p* < .001). Multivariable logistic regression revealed that factors associated with an increased likelihood of reporting high comfort level (≥ 6/10) where being a pharmacist, having a relative living with CP, a greater percentage of past-year continuing educational activities about CP management, and higher perception of short-acting opioids risks. Factors associated with lower comfort levels where being a nurse practitioner, fewer years of experience, living in a remote region, living with CP, and higher perception of long-acting opioids risks. Practice setting was also associated with comfort level, but not sex at birth nor gender identity.

**Discussion/Conclusions**: Comfort level regarding prescribing in chronic pain varies according to socioeconomic/professional factors, which can lead to disparities in the quality of care and outcomes for patients. Our results reinforce the importance of investing in initial training and continuing education for prescribers.

**Codesigning a Canadian adaptation of a lifestyle-oriented intervention aimed to improve daily functioning of individuals living with chronic pain: identifying knowledge-to-practice gaps**

Julie Masse^a^, Svetlana Solgaard Nielsen^b^, Jeanette Reffstrup Christensen^b^, Søren T. Skou^b^, José Côté^c^, Sara Saunders^d^, Émilie Lagueux^e^, Aline Boulanger^f^, Mélanie Lussier^f^, Marc O. Martel^d^, Mark Ware^d^ and M. Gabrielle Pagé^c^

^a^Université de Montréal, ^b^University of Southern Denmark, ^c^Centre de recherche du Center hospitalier de l’Université de Montréal, ^d^McGill University, ^e^Université de Sherbrooke, ^f^Centre hospitalier de l’Université de Montréal

**Introduction/Aim**: Living with chronic pain implies major lifestyle changes. Surprisingly few interventions target occupational outcomes rather than focussing only on reducing pain. Redesign your Everyday Activities and Lifestyle with Occupational Therapy (REVEAL(OT)) is a lifestyle-oriented intervention initially developed and studied in Danish multidisciplinary specialized pain clinics that facilitates more effective activity pacing strategies in real-life daily routines and improves satisfaction and well-being for individuals affected by chronic pain. We are coconstructing and evaluating a Canadian adaptation of REVEAL(OT).

**Methodology**: This participatory action research involves the original authors of REVEAL(OT), people with lived chronic pain experience, clinicians and managers, and is taking place in two Montreal specialized pain clinics. In Phase 1 of the project, the identification of current practice gaps will be presented. Using a structured template based on Proctor et al.’s taxonomy (2011), we identified gaps between lifestyle-oriented intervention evidence and guidelines versus existing practices within these two clinical settings.

**Results**: Our preliminary results show that a number of contextual factors need to be considered in order to improve the fit between REVEAL(OT) and its new Canadian context of delivery. These include (1) occupational therapists’ knowledge and skills about strategies to facilitate lifestyle changes, (2) patient pain care trajectories and the timing of interdisciplinary treatments offered, and (3) the availability of organizational support to combine on-line with in-person interventions.

**Discussion/Conclusions**: This first step will inform upcoming focus groups with partners and supports the pertinence of developing a Canadian version of REVEAL(OT) manual. These efforts promote the use of lifestyle-oriented interventions in conjunction with current care.

**Painful procedures in hospitalized infants – what has changed over the past three decades?**

Mariana Bueno^a^, Megha Rao^b^, Prabhlin Aujla^c^, Charles Victor^d^ and Bonnie Stevens^a^

^a^The Hospital for Sick Children, ^b^Mount Sinai Hospital, ^c^McMaster University, ^d^Institute for Clinical Evaluative Sciences (ICES)

**Introduction/Aim**: Infants in the Neonatal Intensive Care Unit (NICU) undergo multiple painful procedures daily. Minimizing the frequency of painful procedures is critical to reduce pain exposure and its effects. Cruz (2016) established the frequency of painful procedures in the NICU; however, knowledge mobilization and practice trends were not identified. We aimed to determine if the frequency of painful procedures for hospitalized infants has changed over time.

**Methodology**: A scoping review was conducted using methodology by Arskey and O’Malley (2005) and Tricco (2018).  Frequency of painful procedures was determined by (a) chronologic period (1995–2012 and 2013–2023) and (b) data observation period (1 week, 2 weeks, >2 weeks). Standard inverse-variance random-effects meta-analyses were used to combine studies. Heterogeneity between studies was quantified using the I^2^ statistic.

**Results**: Of 2,622 unique citations, 64 full-text articles were reviewed; 23 were included. Six studies identified by Cruz (2016) and six publications from reference lists were included, accounting for 35 studies in this review. These studies, from 16 countries, were published from 1995–2023. The mean number of painful procedures/neonate /day was 7.38 (95%CI 5.60, 9.17) ranging from <2 to 17 painful procedures/infant/ day. There was no statistically significant difference by chronologic period (*p* = .16) but there was a higher number of painful procedures in studies that observed frequency of painful procedures for 2 weeks (*p* = .01). There was high heterogeneity between the studies for all analyses. 

**Discussion/Conclusions**: Frequency of procedural pain in the NICU has not significantly changed in the past three decades leaving infants vulnerable to its consequences.

**Power Over Pain Portal: A window of opportunity feasibility study at the Ottawa Hospital Pain Clinic**

Amin Zahrai^a^, Alesha King^a, b^, Etienne Bisson^a^, Yaad Shergill^a^, Lynn Cooper^a^, Natalie Zur Nedden^a^, Rachael Bosma^c, d^, Daniel James^a, e, f^ and Patricia Poulin^a, e, f^

^1^Ottawa Hospital Research Institute,^2^Memorial University of Newfoundland, ^3^University of Toronto, ^4^Women’s College Hospital, ^5^The Ottawa Hospital Pain Clinic, ^6^University of Ottawa

**Introduction/Aim**: The Power Over Pain (POP) Portal provides free access to a range of pain self-management resources, including education, self-directed courses, peer support, and interactive workshops. In preparation for a window-of-opportunity multisite implementation-effectiveness study, we completed a single-site feasibility study at The Ottawa Hospital Pain Clinic.

**Methodology**: Prospective participants were adults living with chronic pain newly referred to the pain clinic. Patients were contacted by a pain clinic clerk and referred to the POP team for screening and study consent procedures. Participants who consented attended a 1:1 orientation delivered by a POP team member and were invited to use the POP Portal for 3 months. Feasibility and adoption measures were collected. Feasibility thresholds were set as: (1) Proceed with protocol if we recruit 80 participants and achieve 80% completion rate; (2) Modify and reevaluate if we recruit 40 participants and achieve at least 30% completion rate; (3) Stop if we didn’t meet either condition of threshold 2.

**Results**: Over a 4-month period (late March to early August 2023), 114 patients were referred to the POP team (61% female, *mean_age_* (*SD*) = 52.0 (15.5) years). 80 patients consented (70.2%; recruitment rate = 5.0/week) and 40 (50%) completed all study procedures at 3-month follow-ups.

**Discussion/Conclusions**: The implementation-effectiveness study may be feasible with modifications to the protocol. This could include providing a demonstration of POP prior to the consent procedures and reducing burden on participants (e.g., length of consent form, number of questionnaires).

**Evaluating myoActivation as an intervention for chronic pain in a population living with social and health inequities: quantitative findings from a mixed methods pilot study**

Barb L. Eddy^a^, Nicholas West^b^, Ly Nguyen^a^, Leanne M. Currie^c^, Hattie Daumann^a^ and Gillian Lauder^c^

^a^Vancouver Coastal Health, ^b^BC Children’s Hospital Research Institute, ^c^University of British Columbia

**Introduction/Aim**: Vancouver’s Downtown Eastside (DTES) is home to one of the most structurally vulnerable populations in Canada, living with substance use, trauma, and limited access to pain care. myoActivation® is an innovative nonpharmacological process that enables expedient structured assessment and treatment of myofascial dysfunction and pain in primary care. Our aim was to analyze quantitative data collected during a longitudinal mixed methods pilot study to determine the impact of myoActivation on pain intensity, physical function, and quality of life in the DTES population.

**Methodology**: Following ethical approval, we conducted a prospective observational study of patients from the DTES receiving myoActivation assessment (includes structured movement tests) and treatment (a needling technique) for chronic pain. Data were collected at baseline, 4, 12 and 24 weeks using validated tools, including the PEG scale (Pain, Enjoyment of life, General activity) and self-reports of substance and analgesic use.

**Results**: Thirty-five people participated, including 14/35 (40%) with opioid use disorder. The PEG score was median (interquartile range) 7.7 (6.7–8.7) at baseline. PEG scores improved at each follow-up, with a mean difference from baseline of −2.5 (95%CI −3.4 to −1.5, *p* < .001) at week 24, corresponding to a clinically significant (>30%) improvement. At week 24, 9/27 (33%) participants also reported reduced substance use.

**Discussion/Conclusions**: This pilot study suggests that myoActivation can be an effective tool in managing myofascial pain and dysfunction in an inner-city population, with a lasting impact and a concurrent positive impact on drug and medication use. A larger study is required to confirm these findings.

**Understanding the impacts of myoActivation dry needling pain care in a population living with social and health inequities: qualitative findings from a mixed methods pilot study**

Barb L. Eddy^a^, Hattie Daumann^a^, Ly Nguyen^a^, Nicholas West^b^, Gillian Lauder^c^ and Leanne M. Currie^c^

^a^Vancouver Coastal Health, ^b^BC Children’s Hospital Research Institute, ^c^University of British Columbia

**Introduction/Aim**: myoActivation is a unique systematic approach to dry needling that addresses chronic pain and dysfunction associated with myofascial injuries and scars. Assessment includes a lifetime chronology of physical injuries, noting emotional trauma, and functional movement. Our aim was to analyze qualitative data collected during a mixed methods longitudinal pilot study to determine the impacts of myoActivation treatments on people living with social and health inequities in the inner city of Vancouver.

**Methodology**: Patients were followed for 24 weeks and received at least one myoActivation treatment. Semi-structured interviews were conducted with some participants by phone between 12–24 weeks to explore personal experiences of myoActivation in relation to physical and emotional aspects of their life as well as drug and analgesic use. Coding was completed using thematic content analysis.

**Results**: Thirty-five people participated; 18/35 (51%) completed an interview. At baseline, 16/18 (89%) reported a history of physical or sexual assaults, 14/18 (77%) had a mental health diagnosis, and 13/18 (72%) were using at least one substance. Qualitative themes included, “Keep trying to find ways to feel better,” “Changing the landscape of suffering,” “Stepping back into life despite uncertainties,” “Relief without drugs,” and “Location matters.” Risks included surfacing of emotions and memories related to past trauma. At 24 weeks, most participants self-reported less pain and mental/social health improvements, and some reported fewer analgesics and reduced drug use.

**Discussion/Conclusions**: myoActivation may be an effective and acceptable treatment for people with chronic pain embodied with trauma and living with social and health inequalities.

**Guiding the future of pediatric pain research toward an intersectional approach**

Kathryn Birnie^a, b^, Christine Chambers^b, c^, Cate Creede^d^, Marsha Campbell-Yeo^c^, Isabel Jordan^b, c^, Melanie Noel^a^, Jennifer Parker^c^, Jennifer Stinson^b, e^ and White Point Pediatric Pain meeting delegates^f^

^a^University of Calgary, ^b^Solutions for Kids in Pain, ^c^Dalhousie University, ^d^The Potential Group, ^e^The Hospital for Sick Children, ^f^various

**Introduction/Aim**: The field of pediatric pain has rapidly expanded since the early 1980s, culminating in key recent contributions including a 2021 Child and Adolescent Lancet Commission; however, relevant criticism exists about the lack of intersectionality in pediatric pain research, care, policy, and training. A collaborative, exploratory meeting was organized to co-develop a strategy to guide the future of pediatric pain research that incorporated foundational work and engaged deeply with intersectional equity and social justice perspectives.

**Methodology**: 41 invited delegates attended a 1.5-day facilitated meeting at White Point, NS in October 2023. The delegates purposefully represented diverse roles (researcher, health professional, decisionmaker, lived experience, philanthropic), career stage (trainee to senior career), race, sex and gender, country (Canada, Australia, Belgium, Spain, United Kingdom, United States), and discipline (psychology, nursing, medicine, pharmacy, physical therapy, neuroscience, basic science). Delegates mapped the current landscape and discussed opportunities for bold impact, increasing intersectionality, strengthening collaborations, and accelerating knowledge mobilization.

**Results**: The developed acceleration agenda for pediatric pain prioritized: (a) creation of an acute pain task force; (b) addressing the Top 10 priorities for chronic pain; (c) systematizing patient partnership; d) expanding structural collaboration capacity; (e), diversifying and globalizing research participation; (f) supporting diverse trainees and early career researchers; and (g) building community capacity for culturally integrated, anti-oppressive research. Evaluation data from 33 (80%) delegates indicated “excellent” meeting conversations, networking and collaboration, and overall satisfaction.

**Discussion/Conclusions**: This work provides a roadmap for the field of pediatric pain to value different perspectives and share a commitment toward intersectionality and impact.

**The impact of morphine on nerve injury recovery and lipid metabolism: sexually dimorphic behavior and emerging molecular targets**

Sierra Stokes-Heck^a, b, c^, Julia Canet-Pons^a,b, c^ and Tuan Trang^a, b, c^

^a^Hotchkiss Brain Institute, ^b^Cumming School of Medicine, ^c^University of Calgary

**Introduction/Aim**: Neuropathic pain is among the most debilitating types of chronic pain conditions. Opioid medications are often used despite their poor efficacy in treating neuropathic pain symptoms. Notably, emerging evidence suggests that rather than alleviating neuropathic pain, opioids may worsen mechanical allodynia. Morphine has been shown to exacerbate nerve injury-induced mechanical allodynia, but the cause is not understood. Both morphine and nerve injury have been implicated in perturbations of myelin. As myelin is primarily composed of lipids, here we determine whether lipid metabolism alterations are a potential mechanism underlying morphine exacerbated neuropathic pain.

**Methodology**: Chronic constriction injury (CCI) was performed on eight-week-old female and male C57/B6J mice. Development of mechanical allodynia was assessed until return to baseline paw withdrawal threshold using the von Frey filament test. RNA extracted from lumbar spinal cord tissue was profiled using lipid metabolism arrays. Validation of genetic targets were performed at the RNA and protein level.

**Results**: We found that morphine treatment of CCI prolonged mechanical allodynia and recovery from nerve injury. This effect of morphine prolonged pain is further delayed by two weeks in female mice. RNA profiling arrays for lipid metabolism revealed gene candidates in male mice that were further validated at the RNA and protein levels in both sexes.

**Discussion/Conclusions**: These results suggest that morphine delays recovery from CCI by dysregulating spinal cord lipid metabolism. As oligodendrocytes are the myelinating cells of the CNS, we will next explore whether alterations in lipid metabolism are linked to changes in this cell type.

**Assessing the quality of referrals to a community-based chronic pain clinic: Enhancing effectiveness and patient outcomes**

Angela Mailis^a^, Amna Rafiq^b^, Amol Deshpande^a^ and S. Fatima Lakha^a^

^a^Pain and Wellness Center, ^b^Queen’s University

**Introduction/Aim**: Since chronic pain patients are complex with significant medical and psychiatric comorbidities, referrals to specialty pain clinics are often necessary. The present study explores the quality of information submitted and the profile of referring physicians associated with rejected patient referrals by a community pain clinic.

**Methodology**: A retrospective cross-sectional study was conducted on a series of consecutive new patient referrals, rejected by a community pain clinic (November 2021- June 2022). Data were collected on the reasons for rejected referrals and physicians responsible for these referrals using the public database of CPSO.

**Results**: During the study period 120 new referrals made by 99 physicians (88% primary care providers or PCPs; male/female ratio 1:1.2; 53% Canadian university graduates) were rejected because of inadequate information (62%) or because they were inappropriate (38%). Only 29% of referring physicians (all PCPs) used the clinic’s referral form, and despite this, 74% of the referrals were rejected due to inadequate information. Just 38% of rejected referrals were resubmitted within 13 ±18 days and accepted.

**Discussion/Conclusions**: A significant number of referrals to our pain clinic (primarily from PCPs) are rejected for mainly avoidable reasons. The process requires 81–110 hrs of staff time/year. Rejected referrals create significant amount of administrative work adding to factors promoting burn-out of health providers. Without additional healthcare resources, our study will highlight simple but effective improvements in the referral process on behalf of the referring physicians that could facilitate patient care, avoid unnecessary delays, and decrease possible sources of patient complaints.

**A pilot window-of-opportunity study of the Power over Pain Portal at The Ottawa Hospital Pain Clinic: A summary of qualitative findings**

Alesha King^a, b^, Amin Zahrai^b^, Etienne Bisson^b, c^, Yaadwinder Shergill^b^, Lynn Cooper^b^, Natalie Zur Nedden^b^, Rachael Bosma^d, e^, Joshua Rash^a^, Daniel James^b, f, g^ and Patricia Poulin^b, f, g^

^a^Memorial University of Newfoundland, ^b^Ottawa Hospital Research Institute, ^c^Kingston Health Sciences Center, ^d^University of Toronto, ^e^Women’s College Hospital, ^f^The Ottawa Hospital, ^g^University of Ottawa

**Introduction/Aim**: The Power Over Pain (POP) Portal offers free access to pain self-management resources across a Stepped Care 2.0 continuum for people living with pain (PLWP). We aimed to evaluate participants’ experience of POP with attention to: (1) the usability and acceptability of the POP Portal; and (2) facilitators and barriers of implementing POP among PLWP referred to a tertiary pain clinic.

**Methodology**: PLWP referred to The Ottawa Hospital Pain Clinic who used the POP Portal for 3-months were invited to participate in semi-structured interviews to assess Portal acceptability, usability, barriers and facilitators of use, and impact on pain symptoms. Recruitment occurred until saturation was reached. Interviews with 9 PLWP were recorded, transcribed and thematically analyzed by two coders.

**Results**: The POP Portal was widely accepted across participants; patients reported they enjoyed using the Portal (*N*= 5) and recommended use of POP to other PLWP (*N*= 4). The POP Portal was found beneficial for mental health concerns (*N*= 4), understanding pain (*N*= 4), and offered a variety of resources that matched the participants interests, concerns, and readiness (*N*= 5). Some participants indicated the quantity of resources on POP can be overwhelming (*N*= 4), and several participants suggested that a 1:1 interactive service with a healthcare professional be included in future iterations of the POP Portal to support the use of POP for chronic pain management (*N*= 6).

**Discussion/Conclusions**: The POP Portal was found to be accepted among PLWP; however, improvements may be made to improve portal usability and encourage use across a broader continuum of care.

**Development of a presurgical chronic postsurgical pain risk stratification tool for cardiothoracic surgeries in adults**

Andrew Walker^a^, Brittany Rosenbloom^b^, Beate Sydora^a^, Kayla Denness^a^, Dorothy Wong^b^, Hance Clarke^c^, Sanjay Beesoon^a^ and Nivez Rasic^d^

^a^Alberta Health Services, ^b^Women’s College Hospital, ^c^University Health Network at Toronto General Hospital, ^d^University of Calgary

**Introduction/Aim**: Chronic postsurgical pain (CPSP) affects 10–50% of the surgical population. Identification of patients at risk for developing CPSP is important to ensure appropriate peri-operative care. We sought to develop a presurgical risk stratification predictive tool for the development of CPSP using cardiothoracic surgical patients. We present our model with internal validation metrics.

**Methodology**: We employed a database of 920 patients from the University Health Network (Toronto, ON). Systematic literature review and expert consensus identified 15 candidate variables of interest. We used adaptive least absolute shrinkage and selection operator (lasso) regression to identify the suite of variables presenting with the lowest prediction error. Internal validation was performed using 2000 bootstrapped replicates.

**Results**: 30% of our cohort reported CPSP at three months. Our predictive model included age [odds ratio 0.96 (95%CI: 0.95–0.98)], female sex [1.67 (1.16–2.38)], preoperative chronic pain (yes/no) [1.45 (1.01 to 2.09)], somatization score [1.05 (1.03–1.08)], and HADS-D score [1.10 (1.04–1.16)] with a c-statistic of 0.73 (0.69–0.77). Sensitivity and specificities at the Youden index were 65% (58%–71%) and 65% (61%–69%), respectively. Internal validation reported a corrected c-statistic of 0.72. Calibration curves presented corrected slope and intercept values of 0.97 and −0.03.

**Discussion/Conclusions**: Our corrected c-statistic remained stable after internal validation. Such values suggest good discriminatory ability and align with prior results. Apparent and bias-corrected calibration curves were in close agreement across all predicted probabilities. As such, we should expect similar model diagnostics upon external validation. Our model shows promise as a useful presurgical risk stratification tool to identify patients at-risk of CPSP.

**Pharmacological and nonpharmacological interventions for cancer-related pain management: An overview of Cochrane reviews**

Roxanna Wang^a^, Dorisa Meng^a^, Zhaoxia Li^b^, Lucas Lorimer^c^, Serena Wei^d^, Fan Wang^e^, Henry Kwon^f^, Chunming Wang^g^, Jason W. Busse^h^ and Li Wang^i^

^a^McMaster University, Michael G. DeGroote School of Medicine, ^b^The Second Hospital of Gansu Province, Lanzhou, ^c^Western University, Schulich School of Medicine and Dentistry, ^d^McMaster University, Faculty of Health Sciences, ^r^Minzu University, School of Clinical Medicine, ^f^Wayne State University School of Medicine, ^g^Guangdong Academy of Sciences, Guangdong Science and Technology Library, ^h^McMaster University, Department of Anesthesia, ^i^McMaster University, Michael G. DeGroote Institute for Pain Research and Care/ Department of Anesthesia

**Introduction/Aim**: Cancer-related pain affects 40% to 70% of cancer patients and is associated with disability and reduced quality of life. Cancer-related pain management is complicated due to differences in cancer treatments, cancer stages, pain causes, and comorbidities. We aimed to conduct an overview of Cochrane reviews, one of the best evidence sources, to assess and summarize the evidence of cancer-related pain management.

**Methodology**: We searched the Cochrane Database of Systematic Reviews on October 5, 2023, for systematic reviews of RCTs reporting any pharmacological and nonpharmacological interventions for cancer-related pain management. Paired reviewers independently screened the title/abstracts and full texts for eligibility, extracted data, and assessed quality of systematic reviews and quality of evidence using AMSTAR 2 and GRADE approach.

**Results**: Thirty-two Cochrane reviews (CSRs) including 672 RCTs and 88,868 cancer patients proved eligible. Of 32 CSRs, 23 (72%) included adult cancer patients only, but 9 CSRs included both pediatric and adult patients. Interventions for cancer pain management included pharmacological interventions (21 CSRs) and nonpharmacological interventions (11 CSRs). We are in the process of data analyses and will be prepared to present our findings at the Canadian Pain Society 2024 Annual Scientific Meeting.

**Discussion/Conclusions**: Our overview will be the first to systematically evaluate the interventions for cancer-related pain management and associated quality of evidence. In the future, we will conduct a network meta-analysis to rank the interventions for optimizing cancer pain management.

**Demographic and clinical factors impacting chronic pain among teachers seeking care at a community pain clinic**

Shehnaz Fatima Lakha^a^, Philip Amoabeng^b^ and Angela Mailis^a^

^a^Pain and Wellness Center, ^b^University of Toronto

**Introduction/Aim**: Research indicates a link between chronic pain and occupational factors, with certain worker groups, including teachers, being more susceptible due to job-related features. Therefore, this study aims to investigate the impact of demographics and associated factors on chronic pain among schoolteachers.

**Methodology**: This was a cross-sectional retrospective descriptive study. We included the data of all teachers (*N* = 20, elementary or high school, special needs, special education) with chronic pain from 2019–2023 admitted to an interdisciplinary pain program. Data collected included demographic information, pain characteristics, emotional/ functional status obtained by validated instruments, psychiatric and medical diagnosis, and Global Impression of Change (GIC).

**Results**: Female/male ratio was 9:1 (*p* < .5); mean age 46 ± 10 years (26–62 yrs.); 75% Canadian-born (*p* < .05), 70% (*p* < .05) married, 50% with university degree, 60% employed at time of admission, with 1/3 were unable to work due to pain. Mean pain rating was 6 ± 1.7, and commonest pain site low back (*n* = 17). However, 70% had >5 sites or chronic widespread pain. Significant biomedical pathology was observed in 15%, and formal DSM-5 diagnosis by a psychologist was rendered to 70%. About 1/3 had received multiple injections, and all reported utilization of mental health services before admission to the pain program. Data on emotional and functional status outcome batteries will be presented in detail.

**Discussion/Conclusions**: This study adds to the limited research on the unique demographic and pain characteristics of teachers with chronic pain, pointing to overwhelming prevalence of women in our sample, and offers essential demographic and clinical benchmark data valuable for planning and policy.

**Constructing authentic vignettes for intersectional insights in communicating needs concerning complex chronic pain**

Shehnaz Fatima Lakha^a^, Peter Pennefather^b^ and Deborah Fels^a^

^a^Toronto Metropolitan University, ^b^University of Toronto

**Introduction/Aim**: Vignettes enhance understanding and reflection in healthcare research by framing complex subjects. The purpose of this study is to explore the feasibility of summarizing qualitative results in the form of vignettes composed of short, pain-related, unstructured video-based commentary from an individual experiencing chronic pain over time. The objective is to explore if that medium is suitable for constructing a qualitative presentation of the incapacities associated with the lived experience of this chronic condition.

**Methodology**: Using our experimental MyHealthMyRecord (MHMR) mobile app, a person with chronic pain recorded 65 short video commentaries over 3 months. The videos, averaging 36 seconds, covered unprompted commentary on diverse aspects of the person’s pain-related experiences and challenges. Thematic analysis sorted these clips into eight themes related to physical pain, difficulties or successes in daily activities, overall concerns, frustrations, or other pain-related events.

**Results**: This poster presents the feasibility of creating a summary digital vignette based on this thematic analysis that can communicate insights from the qualitative findings. We describe how the MHMR technology could be used to generate a form of “vignette didactics” for recognizing patterns associated with positive or negative patient-centered care outputs from the patients’ perspective.

**Discussion/Conclusions**: Together, the themes and vignettes augment understanding of the care recipient’s experiences, challenges, and painful moments in the context of providing greater insight into intersectional factors that shape their pain-related experiences over time and between healthcare provider visits. Moreover, these vignettes can function as practical case studies that can shed light on issues related to person-centered.

**The CircaMS Study: Circadian rhythmicity as a biomarker for fatigue and pain phenotypes in MS**

Doriana Taccardi^a^, Hailey Gowdy^a^, Ana Wing^a^, Moogeh Baharnoori^a^, Marcia Finlayson^a^ and Nader Ghasemlou^a^

^a^Queen’s University

**Introduction/Aim**: There are 16 approved treatments for multiple sclerosis (MS) in Canada, helping reduce the risk of clinical relapse and the formation of new or active lesions. These medications generally do not reduce key symptoms like fatigue and pain. A greater understanding of symptomatic phenotypes and experiences is needed to better manage MS. Circadian (24-hour) rhythms control most physiological systems and are the focus of our multi-disciplinary approach. Identifying how these rhythms affect daily fluctuations of fatigue and pain and immune biomarkers in the blood cells of people with MS (pwMS) could identify new therapeutic opportunities.

**Methodology**: We hypothesize that pwMS exhibits altered circadian rhythmicity in fatigue and pain, and this variability is associated with immune biomarkers and self-reported symptoms. We document ongoing intradaily fluctuations in fatigue and pain by ecological momentary assessment (10-day e-diary) in a large cohort across Canada. Blood samples are collected two times a day in a smaller cohort of pwMS in Kingston.

**Results**: Despite a limited sample size, our preliminary analysis showed that pwMS exhibited some variation in pain profiles. When clustered according to fatigue and pain intensity, we observed variation in the mean fatigue score from morning to evening in the rhythmic pain group and a prevalence of a ‘mixed’ or unpredictable pain phenotype.

**Discussion/Conclusions**: Determining whether immune cell clocks are affected in MS may help to identify new biomarkers of this disease. This is crucial to understanding variability in MS symptomatology; identifying new biomarkers of symptomatic phenotypes in MS; and designing bespoke interventions focused on symptomatology rhythmicity in MS.

**Is inflammation the solution for chronic neuropathic pain?**

Lucas Vasconcelos Lima^a^, Marc-Antoine Johnson^a^, Melanie Di Maria^a^, Mia Dominguez-Dahan^a^, Oakley Morgan^a^, Luda Diatchenko^a^ and Jeffrey Mogil^a^

^a^McGill University

**Introduction/Aim**: Growing evidence suggest that acute inflammatory response play a critical role in triggering the resolution of acute pain, preventing chronification. Recent findings also demonstrate that inflammatory agents can promote axonal regrowth in neuronal injury models in mice via recruitment of specific immune cells. Based on that, we investigate whether an inflammatory insult could trigger the resolution of chronic neuropathic pain.

**Methodology**: First, mice received a spared nerve injury (SNI) (or sham), a model known to result in long-lasting (over 6 months) allodynia. 30 days later, when chronic neuropathic pain is stablished, mice received an inflammatory insult in the form of an injection of complete Freund’s adjuvant (CFA) into the hind paw ipsilateral to SNI/sham. Control groups (for both sham and SNI) received saline injections. Their pain behavior was followed over time by measurement of mechanical withdrawal thresholds, via Von Frey filaments, at multiple relevant timepoints.

**Results**: Mice with chronic SNI who received an injection of CFA experienced a significant increase in mechanical thresholds (hypoalgesia) starting 9 days after CFA and lasting between 40 and 60 days. Ongoing experiments look at different timings for the CFA injection (3 days after SNI, 3 days prior to SNI and 90 days after SNI) and different locations (ipsilateral leg, cheek, contralateral paw).

**Discussion/Conclusions**: Our preliminary data shows that inflammation induced by CFA triggered a delayed, but long-lasting, hypoalgesic response. This new, surprising finding suggest immune cells related to acute inflammatory response have the potential to resolve chronic neuropathic pain.

**Pain severity, pain interference, and health-related quality of life for adults using authorized medical cannabis products: preliminary findings from the Medical Cannabis Real World Evidence study**

Calvin Diep^a, b^, Karim Ladha^a, b, c^, Praveen Ganty^d^, Sonalben Thaker^d^ and Hance Clarke^a, d^

^a^Department of Anesthesiology and Pain Medicine, University of Toronto, ^b^Institute of Health Policy, Management and Evaluation, Dalla Lana School of Public Health, University of Toronto, ^d^Department of Anesthesia, St. Michael’s Hospital, Toronto, ON, ^e^Department of Anesthesia and Pain Management, Toronto General Hospital, Toronto, ON

**Introduction/Aim**: Longitudinal real-world data from medical cannabis users may complement efficacy data from clinical trials to generate evidence of effectiveness in the broader population. We report preliminary findings from the Medical Cannabis Real World Evidence (MCRWE) study.

**Methodology**: MCRWE is an ongoing national observational study of adults (≥18 years) using authorized medical cannabis products by physician prescription. Purchases of medical cannabis products are recorded during the study. Outcomes of interest were the Numeric Rating Scale for pain severity, PROMIS Pain Interference total score, and a health-related quality of life index score (iQoL), each measured at baseline and 6, 12, and 24 weeks. We fitted generalized estimating equations to assess changes over time for each outcome. We then assessed if age and sex modified changes over time using interaction terms.

**Results**: Since July 2020, 215 adults (mean age 49 years, 69% female, 73% prior cannabis use) from 8 provinces enrolled to use medical cannabis for pain relief. Mean baseline pain severity was 5.5 ± 2.4, pain interference 23.0 ± 6.0, and iQoL 54.3 ± 19.0. All outcomes improved over time; pain severity and pain interference were lowest at 24 weeks (β = −1.1, 95% CI −1.5 to −0.7 and β = −4.4, 95% CI −5.4 to −3.4, respectively) while iQoL was greatest at 6 weeks (β = 6.4, 95% CI 1.9 to 10.9). Age and sex did not modify improvements over time.

**Discussion/Conclusions**: Adults using medical cannabis products guided by their physicians reported improvements in pain severity, pain interference, and quality of life. Future MCRWE investigations will explore associations of different cannabis formulations with outcomes.

**Intolerance of uncertainty and chronic pain in adult survivors of childhood cancer**

Tori Langmuir^a^, Wendy M. Leisenring^b^, Kayla L. Stratton^b^, Élisabeth Lamoureux^a^, Alex Pizzo^a^, Kevin Alschuler^c^, Kevin R. Krull^d^, Lindsay A. Jibb^e^, Paul C. Nathan^e^, Jennifer N. Stinson^e^, Gregory T. Armstrong^d^ and Nicole M. Alberts^a^

^a^Concordia University, Montréal, Canada, ^b^Fred Hutchinson Cancer Center, Seattle, USA, ^c^University of Washington, Seattle, USA, ^d^St. Jude Children’s Research Hospital, Memphis, USA, ^e^Hospital for Sick Children, Toronto, Canada

**Introduction/Aim**: Intolerance of uncertainty (IU) is a dispositional tendency to perceive uncertainty as unacceptable or threatening. Despite the uncertain nature of childhood cancer survivorship *and* chronic pain, no studies have examined IU and chronic pain in this population.

**Methodology**: Adult survivors of childhood cancer (*N* = 228, mean[SD] age =39.6[9.9] years, 50.4% female, 31.7 years since diagnosis) from the Childhood Cancer Survivor Study completed IU (12-item mean), chronic pain (lasting 3 months), pain intensity (1-item), pain interference (7-item mean), pain catastrophizing (i.e., catastrophic thoughts and feelings about pain; 13-item mean), and psychosocial measures. Independent-sample t-tests compared mean levels of IU between survivors with and without chronic pain. Multivariable regression adjusted for sex and age and estimated mean effects (B) with 95% confidence intervals (CI) for associations of pain-related variables with IU among survivors with chronic pain (*N* = 93).

**Results**: The mean level of IU among all survivors was 26.2 (95%CI[24.9–27.5). Higher levels of IU were observed in survivors with chronic pain (*M* = 29.23, *SD* =12.13) compared to survivors without (*M* = 23.70, *SD* =8.78) (*t*[226] = −4.27, *p*<.001). After adjusting for anxiety and depression, higher IU was associated with elevated pain catastrophizing (B[95% CI]; 0.3[0.1–0.5]), but not elevated pain intensity (B[95% CI]; 1.2[−3.0–5.4]) or pain interference (B[95% CI]; 0.1[−0.1–0.2]).

**Discussion/Conclusions**: These findings provide cross-sectional evidence that elevated IU is associated with increased chronic pain as well as pain catastrophizing in childhood cancer survivors. Further longitudinal research is needed to elucidate the nature of the relationship between IU and these pain variables, which will help inform psychosocial screening and intervention for survivors.

**Sensory modulation disorder in patients with autism spectrum disorder treated as chronic pain: a clinical hypothesis**

Rodrigo Deamo Assis^a^, Stéphanie Jacques^a^, Genevieve Duclos ^a^, Zoe Jobin-Chayer^a^ and Eva-Flore Bui-Xuan ^b^

^a^Centré Intégré de Santé et Services Sociaux d’Abitibi-Témiscamingue, ^b^Montreal Children’s Hospital

**Introduction/Aim**: Sensory modulation disorder (SMD) affects sensory processing across single or multiple sensory systems. It is a condition in which nonpainful stimuli are perceived as abnormally irritating, unpleasant, or even painful which leads to sensory overresponsivity. Patients with SMD may demonstrate several nonadaptive behaviors to everyday sensory stimuli such as overreaction to noise or tactile input. We infer that patients with autism spectrum disorder (ASD) and SMD would have a sensory experience like chronic pain. There are currently very few treatments proven to be effective in improving symptoms of sensory hypo- or hypersensitivity. Literature suggests an imbalance between the cortical excitation and inhibition systems. Our hypothesis is this imbalance is a type of nonadaptative neuroplasticity of the central nervous system that may cause a central sensitization.

**Methodology**: Since 2022, our team treats patients with ASD and CSM as chronic pain patients. We already treated 10 patients. Our nonpharmacological treatment program consists of pain education for parents and patients, change in lifestyle habits, nonpain increasing exercises, mindfulness meditation program and sensitive rehabilitation program and the use of noninvasive brain stimulation (transcranial direct current stimulation).

**Results**: As a result, we see an improvement in the motor skills and the cognitive function of some patients.

**Discussion/Conclusions**: Patients with ASD and CSM may be treated as a chronic pain condition. To our knowledge, we are the only Quebec’s team to treat ASD with CSM as a chronic pain condition.

**Applying implementation science for improved uptake and sustainment of innovations in chronic pain care**

Jenny Olson^a^, Megan MacNeil^b^, Kathryn Birnie^b^, Alex Haagaard^c^, Norm Buckley^d^, Brandon Van Dam^d^ and Justin Presseau^a^

^a^Ottawa Hospital Research Institute, ^b^University of Calgary, ^c^Chronic Pain Network, ^d^McMaster University

**Introduction/Aim**: Canadian chronic pain research has advanced considerably in recent years, driven by initiatives like the Chronic Pain Network (CPN), funded through CIHR’s Strategy for Patient Oriented Research, and Health Canada’s Canadian Pain Task force. These initiatives have galvanized production of evidence-based innovations (EBIs) for preventing, assessing, and treating chronic pain. As promising EBIs are developed and practice change is necessitated, it is important to ensure innovations are implemented and to avoid research-to-practice gaps common in other settings. Implementation Science can facilitate supporting the systematic uptake of EBIs into routine practice, through the development of generalizable empirical evidence for optimizing implementation activities.

**Methodology**: We are applying a programmatic Implementation Science-informed approach, in collaboration with people with lived experience, to optimize the integration of CPN-generated EBIs into standard clinical practice. Our approach uses established theories, models, and frameworks across three steps to: (1) Identify who needs to do what differently (e.g., Action, Actor, Context, Target Time); (2) Identify barriers/enablers of implementing EBIs (e.g., Theoretical Domains Framework/Consolidated Framework for Implementation Research); and (3) Identify and codevelop evidence-informed strategies to implement EBIs into clinical care (e.g., Behavior Change Techniques/Expert Recommendations for Implementing Change).

**Results**: This work provides the foundation for delivering and evaluating implementation strategies tailored to optimize the uptake and sustained use of promising EBIs into clinical chronic pain care in Canada.

**Discussion/Conclusions**: Facilitating and evaluating the implementation of EBIs into clinical practice allows Canadians living with chronic pain to realize the benefits of innovations emerging from Canadian chronic pain research.

**Knowledge, attitudes, and beliefs related to persistent pain: A descriptive study among employees of a Quebec-based insurer**

Yannick Tousignant-LaFlamme^a, b^, Frédéric Olivia, Thomas Gerard^a, b^, Anne-Marie Pinard^c^, Hélène Beaudry^a, d^ and Kadija Perrault^c, e, f^

^a^School of Rehabilitation, Université de Sherbrooke, Sherbrooke, Quebec, Canada, ^b^Research Center of the Center Hospitalier Universitaire de Sherbrooke (CRCHUS), Sherbrooke, Quebec, Canada, ^c^École des sciences de la réadaptation, Université Laval, Québec, Canada, ^d^Réseau Québécois de recherche sur la douleur, ^e^Centre interdisciplinaire de recherche en réadaptation et en intégration sociale (Cirris), ^f^Centre intégré universitaire de santé et de services sociaux (CIUSSS) de la Capitale-Nationale

**Introduction/Aim**: The lack of knowledge, misconceptions, false beliefs and negative attitudes related to persistent pain are prevalent among healthcare professionals and the general population. These deficits may result in a misguided attitude toward the people living with chronic pain and potentially impede clinical outcomes. One potential solution to address these issues is to provide tailored training to these stakeholders. However, no study has explored these aspects among case managers in the insurance industry. Considering their role as intermediaries between insured persons and clinicians, this study aims to assess their knowledge, attitudes, and beliefs regarding pain.

**Methodology**: Using a descriptive design, a 58-question online survey was sent to the 558 employees of a Quebec-based insurer. The response frequencies for each question were calculated. The percentage of correct responses for each question was collected, with a response considered “correct” if >60% of the respondents had the correct answer.

**Results**: The survey response rate was 46.2% (255/558). Among these respondents, 188 (74%) never received any pain-related training. For general pain knowledge, the participants answered only 8 out of 17 questions (47%) correctly. Regarding specific knowledge about chronic pain, 10 out of 15 questions (66%) were correctly answered. Lastly, for beliefs and attitudes about pain, 22 out of 26 questions (85%) were answered correctly by the participants.

**Discussion/Conclusions**: Employees of a Quebec-based insurer demonstrated significant gaps in pain-specific knowledge as well as inadequate beliefs and attitudes about pain. These findings may serve as a target for future training development for these stakeholders.

**Prognostic factors of pain, disability, and poor outcomes in persons with neck pain – An umbrella review**

Thomas Gerard^a, b^, Florian Naye^a, b^, Pierre Langevin^c, d^, Simon DeCary^, b^, Chad Cook^e, f, g^, Nathan Hutting, Marylie Martel^a^, Yannick Tousignant-LaFlamme^a, b^

^a^School of Rehabilitation, Université de Sherbrooke, Sherbrooke, Quebec, Canada, ^b^Research Center of the Center Hospitalier Universitaire de Sherbrooke (CRCHUS), Sherbrooke, Quebec, Canada, ^c^Centre interdisciplinaire de recherche en réadaptation et intégration sociale (Cirris), Université Laval, Quebec City, Quebec, Canada, ^d^PhysioInteractive/Cortex, Quebec City, Quebec, Canada , ^e^Department of Orthopedics, Division of Physical Therapy, Duke University, Durham, NC, USA, ^f^Department of Population Health Sciences, Duke University, Durham, NC, USA , ^g^Duke Clinical Research Institute, Duke University, Durham, NC, USA, ^h^Research Group Occupation and Health, HAN University of Applied Sciences, Nijmegen, the Netherlands

**Introduction/Aim**: Neck pain is a prevalent and disabling condition. Current treatments approaches have shown moderate effects at best in reducing the burden of neck pain. However, targeting prognostic factors is a promising avenue to enhance the management of neck pain. For this purpose, high quality prognostic factors are needed. As the latest umbrella review was published over a decade ago, the aim of this study was to identify, from systematic reviews, prognostic factors pertaining to neck pain.

**Methodology**: We conducted an umbrella review reporting the prognostic factors associated with nonspecific, trauma-related neck pain and cervical radiculopathy. Prognostic factors were synthesized according to the outcome predicted, the direction of the predicted outcome (poorer, better, inconsistent), and the grade of evidence (A to D). Risk of bias analysis was performed with the ROBIS tool.

**Results**: We retrieved 805 citations from three databases, read 25 full texts, and 16 studies met all selection criteria. The predicted outcomes were synthetized into five categories: pain, disability, work-related outcomes, quality of life and poor outcomes. From these studies, we extracted 46 prognostic factors restricted to nonspecific neck pain, 48 for trauma-related neck pain and one for cervical radiculopathy.

**Discussion/Conclusions**: This study synthesized several prognostic factors from people with neck pain that could be useful for clinicians and researchers. We found multiple negative prognostic factors and very few positive prognostic factors – thus the literature informs us on the risk of “poor” outcomes much more than risk of favorable outcomes.

**Depicting the current state of pain management competencies across graduating Canadian physiotherapy students**

Alaanah Bhanji^a^, Nathan Augeard^a^, Fatima Amari^a^, Geoffrey Bostick^b^, Lynn Cooper^a^, Anne Hudon^c^, Jordan Miller^d^, Lesley Singer^a^, Yannick Tousignant-Laflamme^e^, David Walton^f^ andTimothy Wideman^a^

^a^McGill University, ^b^University of Alberta, ^c^University of Montreal, ^d^Queen’s University, ^e^University of Sherbrooke, ^f^Western University

**Introduction/Aim**: In 2021, the Canadian Pain Task Force presented an Action Plan for Pain that called for improved pain education across health professional programs, including physiotherapy (PT). To inform evidenced-based improvements for PT pain education, our team developed a 15-item competency profile that achieved stakeholder consensus, outlining necessary abilities required by PTs to adequately manage pain. We do not yet understand how students are performing in relation to the goals outlined by the competencies.

**Methodology**: We implemented a 28 multiple-choice case-based assessment, that measures 6 competencies. This online assessment was designed to be integrated within curricular activities and administered to Canadian PT students completing their entry-to-practice program in 2022 or 2023.

**Results**: PT students from across 14 Canadian PT programs (*n* = 718) performed best on questions assessing their ability to *use appropriate tools and strategies to evaluate the effectiveness of treatment*; questions were answered correctly 92.0% (*SD* = 25.9) of the time. Students performed worst on questions assessing their ability to *perform a comprehensive assessment using appropriate tools and strategies* and *synthesize and interpret assessment findings*; these questions were answered correctly 61.8% (*SD* = 48.7) and 70.0% (*SD* = 45.8) of the time, respectively.

**Discussion/Conclusions**: These findings depict the current state of PT pain education and targets for improvement. Understanding these targets will facilitate PT educators to modify their program for students to achieve crucial knowledge and skills. Working toward a standard of education among Canadian PT programs will lead to more widely accessible high-quality care for Canadians living with pain.

**Building capacity in primary care for chronic pain and somatic symptom disorder management through education and program needs assessment**

Orit Zamir^a, b, c^, Carrie Schram^a, c^ and Blanca Bolea^a, c^

^a^Women’s College Hospital, ^b^Mount Sinai Hospital, ^c^University of Toronto

**Introduction/Aim**: Chronic pain and other somatic symptom disorder presentations in family practice are common, utilize a disproportionate amount of time and cost and result in considerable distress for providers and patients. Many chronic pain presentations cluster with functional somatic symptoms, which are persistent physical symptoms that often have a complex psychosocial interplay such as irritable bowel syndrome and functional neuronal disorders. There remains a gap in psychosocial education and treatment options within primary care. The aim of this clinical initiative is to assess program needs and confidence in provider care after providing education workshops that reviewed background physiology, mind-body interplay, resources, assessment and management skills to improve care for this population in primary care.

**Methodology**: Family physicians and nurse practitioners (*n* = 58) were surveyed on attitudes, confidence and needs at the start and end of a workshop. Data was collected from two workshops: one for an academic hospital clinic and one for community care providers.

**Results**: Workshops were found to improve level of provider comfort working with this population overall (*t* = 3.10, *p*< .05) including providing education and assessment. There was also more likelihood to make psychological referrals (*t* = 2.33, *p* <.05) and provide brief counseling. The top priority reported for program development included psychological referral options (96%).

**Discussion/Conclusions**: A comprehensive workshop targeting psychosocial aspects of chronic pain and somatic symptom disorders under the umbrella of functional somatic disorders improves several aspects of the encounter and increases likelihood to refer to specialized psychological programs.

**Initiation of an opioid prescription for chronic musculoskeletal pain and associated factors: a retrospective cohort study using the Quebec Integrated Chronic Disease Surveillance System**

Eugene Attisso^a^, Clermont E. Dionne^b^, Edeltraut Kröger^c^, Line Guenette^c^, Sébastien Tessier^a^ and Sonia Jean^a^

^a^Institut national de santé publique du Québec, ^b^Centre de recherche du CHU de Québec et Département de médecine sociale et préventive, Faculté de médecine, Université Laval, ^c^Centre de recherche du CHU de Québec et Faculté de pharmacie, Université Laval

**Introduction/Aim**: The current study aimed at (1) describing the trend of opioid initiation for Chronic musculoskeletal pain (CMP) among adults covered by the Quebec Public Drug Insurance Plan between 2006 and 2022, and (2) identifying factors associated with these prescriptions.

**Methodology**: We performed a retrospective cohort study. The opioid naïve population was defined for each fiscal year between 2006–2007 and 2021–2022. The clinical indication of their new opioid prescription was first identified using a hierarchical algorithm. CMP was identified using administrative data codes, according to the literature or using expert opinion. The age-standardized incidence of this conditions was estimated. Temporal trend analyses were done. Factors associated with this condition were identified using negative binomial Poisson regression.

**Results**: The age-standardized incidence of opioid initiation for CMP decreased from 70 to 9 per 10,000 persons between 2006–2007 and 2021–2022, with an average annual percent change of −12.6 [95%CI: −18.7 to −6.2]. The most important decrease was observed between 2015 and 2018: −25.0 [95 CI%: −27.1 to −24.3]. The factors statistically associated with opioid initiation for CMP were being female (RR = 1.17 [95%CI: 1.15 to 1.19]), age ≥75 years (RR =1.39 [95%CI: 1.34 to 1.44]) and prescriber’s specialty (RR = 2.05 [95%CI: 2.00 to 2.09] for family physicians and RR = 2.71 [95%CI: 2.64 to 2.79] for medical specialists).

**Discussion/Conclusions**: This study shows that opioid initiation for CMP decreased substantially between 2006 and 2022 in Quebec, and that these drugs were more frequently prescribed by medical specialists, among women and those aged ≥75 years.

**Patient values and preferences on medical use of cannabis for chronic pain: A mixed-methods systematic review**

Andrea Darzi^a^, Holly Crandon^b^, Samer Karam^c^, Annie George^d^, Xiaoqin Wang^e^, Rachel Couban^b^ and Jason Busse^a^

^a^Department of Anesthesia, Faculty of Health Sciences, McMaster University, Hamilton, Ontario, Canada, ^b^Michael G. DeGroote National Pain Center, McMaster University, Hamilton, Ontario, Canada, ^c^Department of Health Research Methods, Evidence, and Impact, McMaster University, Hamilton, ON, Canada, ^d^Department of Orthopedic Surgery, McMaster University, Hamilton, ON, Canada, ^e^Clinical Epidemiology Program, Ottawa Hospital Research Institute, Ottawa, Ontario, Canada

**Introduction/Aim**: Evidence alone is insufficient for clinical decision-making, which also requires knowledge of how patients’ value benefits and harms of interventions. We conducted a systematic review of values and preferences studies on medical cannabis in people with chronic pain.

**Methodology**: MEDLINE, EMBASE and PsycINFO were searched till 9 May 2022 for relevant studies. We used a meta-narrative synthesis approach and assessed certainty of the evidence with GRADE CERQual.

**Results**: We included 35 studies with 12,905 participants. High certainty evidence showed that many patients valued benefits of cannabis for chronic pain, even when experiencing adverse events such as fatigue or reduced concentration. Moderate to high certainty evidence found that patient’s decision to use cannabis was influenced by social stigma, cost and accessibility, and support from healthcare providers. Low certainty evidence suggested highly variable values toward medical cannabis among people with chronic pain.

High certainty evidence showed that many patients preferred medical use of cannabis for chronic pain to other medications, in particular opioids, due to perceptions of lower risk. Moderate certainty evidence also showed that patients were motivated to use cannabis to reduce other prescription medications. Moderate certainty evidence suggested that most prefer balanced ratios of tetrahydrocannabinol (THC): cannabidiol (CBD, or high CBD formulations, vs. high THC products. Also, males, experienced users, and patients who used higher THC products or used cannabis recreationally and medically, preferred smoking or vaporizing vs. oral or topical administration.

**Discussion/Conclusions**: Our review identified factors that should be considered when engaging patients in shared decision-making regarding medical use of cannabis for chronic pain.

**Chronic pain self-management among university employees and students: The Pain Hacks U Study**

Andréanne Bernier^a^, Claudie Audet^a^, Sylvie Beaudoin^a^, Nancy Ménard^a^, Christian Bertrand^a^, Adriana Angarita-Fonseca^a^, Gwenaëlle De Clifford-Faugère^a^, Joséanne Desrosiers^a^, Marimée Godbout-Parent^a^, Hermine Lore Nguena Nguefack^a^, Ghita Zahlan^a^, Meriem Zerriouh^a^, Nancy Julien^a^, Abir El-Haouly^a^, Nabiha Benyamina Douma, PhD^a^, and Anaïs Lacasse^a^

^a^Département des sciences de la santé, Université du Québec en Abitibi-Témiscamingue (UQAT), Rouyn-Noranda, Québec, Canada

**Introduction/Aim**: In universities, employees and students primarily engage in office work and often face work-related pain, demanding the need for improved workplace pain management support. In order to plan educational activities and interventions effectively, this study aimed to describe pain self-management strategies, along with barriers and facilitators, among university community members living with chronic pain.

**Methodology**: A web-based cross-sectional data collection, namely the Pain Hacks U Study, was carried out among individuals who work or study at the Université du Québec en Abitibi-Témiscamingue (UQAT) in Quebec, Canada, and live with chronic pain.

**Results**: 261 participants with an average age of 39 years completed the questionnaire; most identified as women (79.2%) and 40.0% as students. The prevalence of use of self-management strategies was 88.7% (women: 90.6%, men: 84.6%, gender-diverse persons: 83.3%), with the three most frequent being exercise (96.6%), relaxation techniques/mindfulness meditation (87.3%), and dietary behavior (74.8%). Self-management strategies were judged by a great majority as moderately/quite/very effective (vs. not/slightly effective) in reducing pain intensity (83.2%), decreasing pain unpleasantness (77.6%), and enhancing the quality of life (83.1%). Only 54.9% adopted self-management strategies based on recommendations from healthcare professionals. Pharmacists (80.6%), physicians (76.7%), physiotherapists (52.4%), and nurses (41.3%) were perceived as the most frequent professionals for assistance with self-management. Barriers to the use of self-management strategies included not having informed loved ones about their chronic pain and kinesiophobia.

**Discussion/Conclusions**: Our study demonstrates that workplace education and interventions are an important opportunity that could help chronic pain self-management and prevent work disability.

**Educational needs and preferences of adult patients with acute pain: a mixed-methods systematic review**

Mélanie Bérubé^a^, Caroline Côté^a^, Michael Verret^a^, Line Guénette^a^, Simon Ouellet^a^, Lesley Singer^b^, Andréane Richard-Denis^c^, Lynn Gauthier^a^, Anne Hudon^c^, Laurence Bourque^a^, Marc-Aurèle Gagnon^a^ and Geraldine Martorella^d^

^a^Université Laval, ^b^McGill University, ^c^Université de Montréal, ^d^Florida State University

**Introduction/Aim**: Many patients experience acute pain that can hamper their recovery. Pain education has been proposed as a strategy to improve acute pain management. However, studies on educational interventions for adults with acute pain are inconclusive. This is mainly due to the need to adopt a more patient-centered approach.

To systematically review and synthesize current evidence from quantitative, qualitative and mixed-methods studies describing patients’ needs and preferences for acute pain (< 3 months) education in adults aged ≥ 18 years.

**Methodology**: We conducted a mixed methods systematic review according to JBI guidelines. We searched seven databases from January 1990 to October 2023. Methodological quality was assessed with the Mixed Methods Appraisal Tool.

**Results**: A total of 32 studies were included (*n* =1880 patients), two-thirds of which were qualitative studies of high methodological quality. Most of the studies were conducted in patients with postsurgical and posttraumatic pain, identified as white with a low level of education. Patients expressed the greatest need for education when it came to what to expect in terms of pain, pain medication and how to take it, and the prevention of opioid use disorders. The preferred methods to receive educational content were: in-person, supplemented with written or online education, and from physicians and multidisciplinary team professionals throughout the acute pain period.

**Discussion/Conclusions**: This review has highlighted aspects to be taken into account to promote a person-centered approach in pain education interventions, mainly for patients with acute pain secondary to surgical procedures or traumatic injuries. The results still need to be confirmed with different patient populations.

**A pilot study on the use of graphic pain scales by pain practitioners and for chronic pain patients**

Gabi Schaffzin^a^

^a^York University

**Introduction/Aim**: There has been much written from the medical and behavioral sciences on the validity, efficiency, and overall utility of graphic pain scales (VAS, body maps, face-based scales). There has not been a major project undertaken, however, to speak with both pain practitioners and patients to connect the design and histories of these scales to the experiences of the people that use them.

**Methodology**: For this pilot project, 24 participants took part in 90-minute online focus groups, made up of up to 6 individuals each, during which researchers showed a series of graphic pain scales and were asked about their experiences with the scales in clinical contexts. No medical records were collected for this study.

**Results**: Most practitioners and patients are unaware of the critical histories accompanying the most popular graphic pain scales in use today. There were a variety of insightful takeaways regarding patients’ attitudes toward the scales, however.

**Discussion/Conclusions**: These scales are often taken for granted, used to answer the brief and rote “how is your pain?” or “where is your pain?” when visiting a doctor’s office, going through emergency triage, or taking part in a clinical study. But the experience of the individual in pain and their interlocutor is inherently shaped by the design of the tool used to facilitate this communication. Based on ethnographic research with the subjects, I gained critical insight into these experiences. Eventually, I hope to understand if conveying these histories in the context of the scales’ use can create a more empowered patient.

**Association of parental acculturation with perceptions of toddler immunization pain**

Lojain Hamwi^a^, Estreya Cohen^a^ and Rebecca Pillai Riddell^b^

^a^York University – Faculty of Graduate Studies, ^b^York University – Faculty of Health

**Introduction/Aim**: Cultural background is one of many important mechanisms that impact pain perception. Previous research suggests that parental ethnic, cultural and/or racial background affects beliefs pertaining to pain expression in children, yet its role in toddler immunization pain is unclear. This study examined the relationship between parental acculturation and their concern for their toddlers’ pain responses.

**Methodology**: During routine immunization visits, parents (*N* = 369) of infants aged 12, 18, and 24 months answered questions about their alignment with North American/Canadian culture and their heritage. They rated their worry about their child’s needle pain before and after the procedure and assessed the pain level experienced by their child.

**Results**: Pearson correlation analyses revealed a small negative correlation was found between parents’ identification with Canadian culture and their children’s perceived needle pain, *r*(359) = −.151, *p* = .004, with no significant link to the parents’ worry. Conversely, a small positive correlation was observed between parents’ connection to their heritage culture and their postneedle worry, *r*(232) = .158, *p* = .016, but this did not significantly relate to the needle or assessment of their child’s pain postprocedure.

**Discussion/Conclusions**: This study reveals that parents who identify more closely with Canadian culture tend to perceive their children as experiencing less pain from immunization needles. Conversely, a stronger identification with their heritage culture correlates with increased parental worry after the procedure. These findings underscore the subtle yet significant influence of cultural identity on parental perceptions and concerns regarding their child’s pain during routine immunizations, suggesting that cultural awareness could enhance pain management strategies in healthcare settings.

**Chronic pain, prescriptions, and peril: Predicting risk trajectories**

Hermine Lore Nguena Nguefack^a^, Nancy Ménard^a^, Sylvie Beaudoin^a^, M. Gabrielle Pagé^b^, Line Guénette^c^, Catherine Hudon^d^, Oumar Mallé Samb^a^ and Anaïs Lacasse^a^

^a^Université du Québec en Abitibi-Témiscamingue, ^b^Centre de recherche du Center Hospitalier de l’Université de Montréal, ^c^Centre hospitalier universitaire de Québec – Université Laval, ^d^Centre de recherche du Center hospitalier universitaire de Sherbrooke (CRCHUS)

**Introduction/Aim**: Quantifying medication-related risk is important for enhancing patient safety, informed decision making, and responsible medication use awareness. This study aimed to describe risk trajectories associated with medication use for chronic pain.

**Methodology**: A cohort study was conducted using the TorSaDE Cohort, which links 2007–2016 cycles of the Canadian Community Health Survey with Quebec health administrative data; 8,760 adults reporting chronic pain and covered by the public prescription drug insurance two years postsurvey completion were selected. The monthly risk associated with medication use was calculated, using the Medication Quantification Scale 4.0, a single score obtained by assigning risk weights to each analgesic/coanalgesic used. Growth Mixture Modeling was applied to identify subgroups (trajectories) of individuals with similar patterns of risk over time.

**Results**: Five risk trajectories were obtained: 1) low-stable risk over time (*n* = 2,988;34.1%), 2) moderate-stable risk (*n* =2,801;32.0%), 3) moderate risk with a marked increase over time (*n* = 601;6.9%), 4) high risk with a marked decrease over time (*n* =597;6.8%), 5) high-stable risk (*n* =1,773;20.2%). The “high-stable risk” group was the one with the highest proportion of females (73.0%) and excessive polypharmacy (≥10 medications: 37.9%). The “moderate-stable risk” group showed the highest proportion of ≥65-year-old participants (62.7%) and the “high-risk-decreasing” group showed the highest proportion of participants with some/most activities prevented because of pain (49.6%) and fair/poor perceived general health (56.9%).

**Discussion/Conclusions**: Different subgroups of people experience varied risk patterns associated with their use of pain medications. It is essential to better understand the particularities and determinants of adverse trajectories to effectively prevent them.

**Exploring the impact of sensitivity to pain traumatization on trauma-related symptoms in Individuals with chronic pain**

Khush-Bakht Zaidi^a^ and Eleni Hapidou^a, b, c^

^a^Michael G. DeGroote Pain Clinic, Hamilton Health Sciences, ^b^Department of Psychiatry and Behavioral Neurosciences, ^c^Department of Psychology, Neuroscience and Behavior, McMaster University

**Introduction/Aim**: Chronic pain has been linked to anxiety and trauma-related symptoms. The Sensitivity to Pain Traumatization Scale (SPTS) is a 12-item measure designed to measure the propensity of developing anxiety-related somatic, cognitive, emotional, and behavioral responses to pain that resemble a traumatic-stress reaction. We aimed to validate the SPTS with participants with chronic pain who completed a pain program.

**Methodology**: Participants completed a 5-week intensive pain program with admission and discharge questionnaires. Individuals participated in education classes, structured activities, and set goals on physical fitness, social re-integration, recreation, nutrition, and family. Only individuals who completed the SPTS and Posttraumatic Stress Disorder Checklist for DSM-5 (PCL-5) at two time points were included in the analyses (*N* = 55).

**Results**: Analyses revealed that the SPTS significantly predicted the PCL-5 (*R*^2^ = .09, *F*= 5.28, *p* = .03) both at admission (= .52) into the program and at discharge (= .60). However, after controlling for depression and anxiety, this relationship was no longer significant. After completing the program, significant reductions were reported in the SPTS (*t* = 3.58, *p* <.001) and the PCL-5 (*t* =3.02, *p* = .002) at discharge compared to admission scores.

**Discussion/Conclusions**: Our findings conclude that there is a strong relationship between chronic pain and a trauma-related responses. Findings suggest that the SPTS has a unique relationship with depression and anxiety, thus future interventions focusing on reducing pain, as well as depression and anxiety symptoms may be beneficial.

**The rise in the mental health complexity among chronic patients seeking tertiary chronic pain care**

Rachael Bosma^a^, Emeralda Burke^a^, Cara Stanley^a^, Christian Aquino^a^, Kimberly Coombs^a^, Brittany N. Rosenbloom^a^, Adriano Nella^a^, Shamalla James^a^ and Tania Di Renna^a^

^a^Women’s College Hospital

**Introduction/Aim**: Approximately 60% of people living with chronic pain experience a mental health disorder. Despite the prevalence of this co-occurrence, access to health services for mental health and chronic pain are often not integrated, resulting in fragmented care or being denied access to the services they need to address these often-complex psychosocial issues. The aim of this study was to examine the number of chronic pain referrals for patients with complex mental health needs that precluded them from pain services and to determine whether there has been a post pandemic increase in the complexity of pain referrals post pandemic.

**Methodology**: We conducted a retrospective chart review of all declined referrals received in 2018 and 2022. The reason for decline was extracted from each chart and were illustrated descriptively (percentages). The proportion of the number of declined referrals between years was compared using chi square tests.

**Results**: From 2018 to 2022, there was an increase (11%-18%) in the proportion of declined referrals due to complexities in mental health that there were unable to be addressed through the pain clinic services/capacity (e.g., need for 1 on 1 psychiatric support) χ^2^ = 10.9, *P* = .0009. Other reasons for declined referrals included patients seeking duplicate services and a lack of primary care provider.

**Discussion/Conclusions**: Results from this study highlight gaps in chronic pain services for people who have co-occurring mental health needs and point to the urgent need for closer integration between mental health and chronic pain care.

**Development of vignettes for qualitative research on physical therapists’ recovery expectations of work-disabled individuals with musculoskeletal pain conditions**

Laury Montemurro^a^, Marie-France Coutu^a^ and Junie Carrière^a^

^a^Université de Sherbrooke

**Introduction/Aim**: This study describes the development and review of vignettes used in research on work disability due to a work-related musculoskeletal pain condition. The vignettes were created to assess the recovery expectations of physical therapists toward workers aged 55 and older who were work-disabled due to a musculoskeletal pain condition.

**Methodology**: A developmental approach was used, following the steps outlined in a study on the development and use of research vignettes with healthcare providers (Cazale et al., 2006) which were evaluated positively in a scoping review by Tremblay et al. (2021). The vignettes contained elements from the personal, healthcare, compensation, and workplace systems described in the Work Disability Paradigm that have a known influence on work disability. Factors that have a known influence on clinicians’ recovery expectations were also considered (Perrot et al., 2009). The structure of the vignettes followed the recommendations on vignette methodology for studying clinician’s decision making (Evans et al., 2015). A comparison table was created to insure elements were equivalent across vignettes. The final vignettes were approved by an expert in the field of work rehabilitation and a rehabilitation physical therapist.

**Results**: The vignettes described female workers in physical therapy 4 to 5 weeks after a work disabling musculoskeletal injury.

**Discussion/Conclusion**s: Future studies may use the vignettes described in this study to assess healthcare providers’ recovery expectations toward work-disabled individuals with a musculoskeletal pain condition. This study may also be used as a guideline for the development of vignettes in rehabilitation research.

**Pain Hacks U: Self-efficacy and perceived effectiveness of pain self-management strategies in a Quebec university community**

Claudie Audet^a^, Andréanne Bernier^a^, Sylvie Beaudoin^a^, Nancy Ménard^a^, Christian Bertrand ^a^, Adriana Angarita-Fonseca^a^, Gwenaëlle De Clifford-Faugère ^a^, Joséanne Desrosiers ^a^, Marimée Godbout-Parent^a^, Hermine Lore Nguena Nguefack^a^, Ghita Zahlan^a^, Meriem Zerriouh^a^, Nancy Julien^a^, Nabiha Benyamina Douma ^a^, Abir El-Haouly^a^ and Anaïs Lacasse^a^

^a^Département des sciences de la santé, Université du Québec en Abitibi-Témiscamingue (UQAT), Rouyn-Noranda, Québec, Canada

**Introduction/Aim**: Self-efficacy, defined as belief in our capabilities to achieve goals, is recognized as a predictor of better functioning in persons living with chronic pain (CP). This study aimed to assess the association between self-efficacy and the perceived effectiveness of pain self-management strategies.

**Methodology**: A web-based cross-sectional survey was performed among employees and students in the community of the University of Québec in Abitibi-Témiscamingue (UQAT) living with CP (Pain Hacks U Study). Self-efficacy was assessed using the 6-item French-Canadian Chronic Pain Self-Efficacy Scale. Participants were asked to indicate whether they used self-management strategies (definition and examples were provided), and the perceived effectiveness of those strategies regarding pain intensity, pain unpleasantness and quality of life (using 5-point Likert scales, recoded into not/slightly effective vs. moderately/quite/very effective). Univariable logistic regressions were performed.

**Results**: 225 members of the university community participated and completed the self-efficacy scale (females: 82.1%, mean age: 38.8 ± 10.6 years, students: 40.1%). Self-efficacy scores were not associated with the use (yes/no) of self-management strategies (*p* >.05). Among participants using self-management strategies (*n* = 201), those with higher self-efficacy were more likely to report better effectiveness of those strategies regarding pain intensity (OR: 1.44;95%CI: 1.21–1.72), pain unpleasantness (OR: 1.40;95%CI: 1.18–1.67) and quality of life (OR: 1.70;95%CI 1.39–2.06). Multivariable regression models will be developed until the presentation.

**Discussion/Conclusions**: Although our study does not establish causal relationships, self-efficacy appears to be a potentially modifiable factor worth targeting. This factor can enhance the likelihood of self-management strategies being effective in improving the quality of life for individuals living with CP.

**Success in pain management: Identifying pivotal moments and the support needed to make them happen**

Mélanie Bérubé^a^, Lesley Singer^b^, Line Guénette^a^, Suzy Ngomo^c^, Laurence Bourque^s^ and Anne Hudon^d^

^a^Université Laval, ^b^McGill University, ^c^Université du Québec à Chicoutimi, ^d^Université de Montréal

**Introduction/Aim**: Approaches aimed at helping people to live well with chronic pain have so far focused on the psychological domain, with modest effect. Describing the pivotal moments for reaching this state by further examining the social perspective can help bring innovative solutions to chronic pain management.

To describe, from the perspective of adults with chronic pain: 1) the pivotal moments in their journey to living well with pain; and 2) the support needed from healthcare professionals and their social network to facilitate these turning points.

**Methodology**: We conducted a qualitative study using a life‐history methodology. We performed semi-structured interviews with persons who considered themselves to be living well with chronic pain, until informational redundancy was achieved. The interview guide aimed to uncover the persons’ experiences of pivotal moments in their journey and what surrounded them. We analyzed data through a narrative analysis.

**Results**: We conducted 25 interviews. Two-thirds of the participants were women (64%), and most were living with chronic pain for an average of 12 years. We identified two main themes that characterize pivotal moments: 1) precursors and stabilizers and 2) processes of redefining the self. The precursor and stabilizing moments support being a partner in care, building a toolbox and having access to facilitating social conditions. The self-defining processes comprise having time and space for reflection, getting involved in the community and breaking away from performance.

**Discussion/Conclusions**: This study identified essential conditions and processes for living well with chronic pain, which could inform future healthcare and social interventions.

**Feasibility of Transcranial Direct Current Stimulation (tDCS) to treat central sensitization in a clinical practice environment**

Rodrigo Deamo Assis^a^, Marie-Philippe Harvey^b^ and Guillaume Leonard^b^

^a^Centre intégré de santé et services sociaux Abitibi-Témiscamingue (CISSSAT), ^b^Université de Sherbrooke

**Introduction/Aim**: Although tDCS is very known at the research field, it is not used at the clinical practice. Since 2018 our clinic uses tDCS to treat chronic pain patients where central sensitization (CS) is present. Our aim is to describe the feasibility of tDCS to manage CS in a clinical practice environment.

**Methodology**: 5-consecutive-days of active tDCS, 20 minutes combined with mindfulness meditation; anode electrode over the M1 region; cathode electrode over contralateral supraorbital region; and intensity of 2 mA. Inclusion criteria’s: to be a patient of CISSS-AT clinic of pain; to have signs of CS; and do not progress with the exercise’s program. Exclusion criteria: to have a neurodegenerative disease or a cognitive impairment. The Visual Analog Scale of Pain (VAS), McGill Pain Questionnaire – short version (MPQ) and the Central Sensibilization Inventory (CSI) were administered during three periods: first day of tDCS (T1), last day of tDCS (T2) and 1 month after the last day of tDCS (T3). For the analysis, we calculated the medium and the standard deviation.

**Results**: 106 patients made the protocol. Decrease of the score in all the tests were observed between T1/T2 andT1/T3. VAS: T1 6.1 ± 2.1; T2 3.6 ± 2.1 and T3 4.5 ± 2.1. MPQ: T1 21.7 ± 9.1; T2 9.5 ± 6.9 and T3 12.2 ± 6.9. CSI: T1 54.3 ± 15.5; T2 25.3 ± 12.4 and T3 29 ±12.2. Side effects: fatigue, headache and tingling sensation.

**Discussion/Conclusions**: tDCS seems to be safe and effective to treat CS in the clinical practice environment.

**Efficacy of a quality improvement project in pediatric procedural pain management during needle procedures**

Raphaëlle Pelc^a^, Julie Paquette^a^, Patricia Laforce^a^, Marie-Joëlle Doré-Bergeron^a^, Jocelyn Gravel^a^, Nathalie Gaucher^a^, Kaitlen Gattuso^a^, Marie-France Langlet^a^, Annie Lacroix^a^, Céline Thémelin^a^ and Evelyne D. Trottier^a^

^a^CHU Sainte-Justine

**Introduction/Aim**: Evaluate the impact of the first 2 years of an institutional QI initiative on managing procedural pain and distress in children undergoing needle procedures. Primary objective was to evaluate the use of multimodal strategies to reduce needle-related procedural pain/distress following the QI initiative. The secondary objective was to compare pain scores for needle procedures before and after the QI initiative.

**Methodology**: Observational study conducted in a university-affiliated pediatric hospital. The QI initiative consisted of mandatory training of healthcare providers (HCP) on procedural pain/distress strategies in order to increase the use of a multimodal approach. Pain scores were measured during needle procedures pre and post implementation of the initiative. Standardized questionnaires were used by a program nurse during needle procedures to document pain scales and pain/distress strategies used by the HCP.

**Results**: From June 2021 to June 2023, 363 audits were conducted on needle procedures for 363 children. Audits took place in 14 hospital services. Common procedures were IV insertions (34%) and venous blood draws (27%). Parental presence and distraction techniques were frequently used (90% and 80% of procedures, respectively).221 audits were pre-implementation and 142 postimplementation. Post QI initiative, the number of procedures realized using multimodal strategies (4 or more strategies) to reduce pain/distress increased (from 60% to 78%). Pain scores recorded for 203/221 audits pre and 134/142 post demonstrated a reduction in severe pain from 25% to 5% postimplementation.

**Discussion/Conclusions**: This QI initiative demonstrated an increase in the use of multimodal strategies and a subsequent reduction in severe pain associated with procedures.

**Contemporary prescription opioid use for pain among Canadian Armed Forces Veterans in Ontario**

Lyndsay D. Harrison^a^, Sophie Kitchen^b^, Marlo Whitehead^b^, Alyson L. Mahar^b^, Jason W. Busse^c^ and Tara Gomes^b^

^a^University of Manitoba, Manitoba Center for Health Policy, ^b^Institute for Clinical and Evaluative Sciences (ICES), ^c^McMaster University, Department of Anesthesia

**Introduction/Aim**: Approximately 40% of Canadian veterans live with chronic pain, which is double the rate in the general population. Although opioids are commonly prescribed for chronic noncancer pain, they provide only modest improvements in pain and are associated with important dose-dependent harms. This study summarizes trends in opioid prescriptions for pain, treatment for opioid use disorder, and opioid-related overdoses between 2013 and 2019 among Canadian Armed Forces veterans in Ontario compared with a matched cohort of Ontario nonveterans.

**Methodology**: Routinely collected Ontario data were linked using unique encoded identifiers.

**Results**: Veterans had a slightly higher prevalence of opioid analgesic use with rates declining from 16% in 2016 to 12% by 2019. Rates of new opioid use declined in both groups and were similar in 2019 (7%). Indicators of potentially unsafe opioid prescribing (high doses; co-prescribing benzodiazepines) declined over time among both groups, although ~10% of veterans were still receiving high doses (≥90 MED), and/or co-prescribed benzodiazepines in 2019. Over time, treatment of opioid use disorder climbed more rapidly among veterans (43% for veterans vs. 23% for nonveterans, from 2016 to 2019). Rates of opioid overdose rose in both cohorts but were higher in veterans.

**Discussion/Conclusions**: Rates of opioid analgesic use are higher in Ontario veterans than matched nonveterans, although the differences are small. These findings suggest an ongoing need to optimize pain management services for Ontario veterans, and to ensure the accessibility of multifaceted treatment and harm reduction services to those at higher risk of opioid-related adverse events.

**Remote versus in-person cognitive behavioral therapy: A systematic review and meta-analysis of randomized trials**

Sara Zandieh^a^, Maryam Abdollahzadeh^b^, Behnam Sadeghirad^c^, Li Wang^d^, Randi E. McCabe^e^, Liam Yao^a^, Briar E. Innes^e^, Annaya Pathak^a^, Rachel J. Couban^d^, Holly Crandon^f^, Kian Torabiardakani^d^, Peter Bieling^e^ and Jason W. Busse^c^

^a^McMaster University, Department of Health Research Methods, Evidence and Impact, ^b^McMaster University, Nutrition Research Center, ^c^McMaster University, Departments of Anesthesia and Health Research Methods, Evidence and Impact (HEI), ^d^McMaster University Michael G. DeGroote National Pain Center, ^e^McMaster University, Department of Psychiatry and Behavioral Neurosciences, ^f^University of Toronto, Institute for Management and Innovation

**Introduction/Aim**: Cognitive behavior therapy (CBT) has been shown effective for several conditions; however, most trials have administered treatment in-person, and it remains uncertain if remote delivery is similarly effective.

**Methodology**: We searched MEDLINE, EMBASE, PsycINFO, CINAHL, Cochrane, and Web of Science from inception to July 4, 2023, for trials that: (1) enrolled adults (≥18 years) presenting with any psychiatric or somatic disorder, and (2) randomized them to therapist-guided remote CBT or in-person CBT. Paired reviewers independently assessed risk of bias and extracted data. We performed random-effects model meta-analyses to pool primary outcomes across eligible trials as the standardized mean difference (SMD), used GRADE to assess the certainty of evidence, and ICEMAN to rate the credibility of subgroup effects.

**Results**: A total of 54 trials that enrolled 5,463 patients were eligible for review. Seventeen studies focused on treatment of anxiety and related disorders, 14 on depressive symptoms, 7 on insomnia, 6 on chronic pain or fatigue syndromes, 5 on body image/eating disorders, 3 on tinnitus, and 1 each on alcohol use disorder and mood and anxiety disorders. High certainty evidence showed little to no difference in effectiveness on primary outcomes between therapist-guided remote and in-person CBT (SMD −0.02, 95% CI: −0.12 to 0.07). We found no credible subgroup effects based on clinical condition or individual vs. group in-person CBT.

**Discussion/Conclusions**: High certainty evidence showed little to no difference in effectiveness between in-person and therapist-guided remote CBT across a range of psychological and somatic disorders.

**Prevalence of chronic noncancer pain among military Veterans: A systematic review and meta-analysis of observational studies**

Abdul Rehman Qureshi^a^, Mansi Patel^b^, Sam Neumark^c^, Li Wang^d^, Rachel J. Couban^d^, Behnam Sadeghirad^d^, Alla Bengizi^b^ and Jason W. Busse^d^

^a^Khalifa University, College of Medicine and Health Sciences, ^b^McMaster University, Department of Health Research Methods, Evidence and Impact, ^c^University of Toronto, Department of Laboratory Medicine and Pathobiology, ^d^McMaster University, Department of Anesthesia

**Introduction/Aim**: Chronic noncancer pain is common among military veterans; however, the prevalence is uncertain. This information gap complicates policy decisions and resource planning to ensure veterans have access to healthcare services that align with their needs.

**Methodology**: We searched MEDLINE, EMBASE, PsycINFO, CINAHL, and Web of Science from inception to February 9, 2023, for observational studies reporting the prevalence of chronic noncancer pain among military veterans. We performed random-effects meta-analysis to pool pain prevalence data across studies and used the GRADE approach to evaluate the certainty of evidence.

**Results**: Forty-two studies that included 14,305,129 veterans were eligible for review, of which 28 studies (*n* = 5,011,634) contributed to our meta-analysis. Most studies (90%; 38 of 42) enrolled U.S. veterans, the median of the mean age among study participants was 55 years (IQR 45 to 62), and 93% were male. The pooled prevalence of chronic noncancer pain was 45%; however, we found evidence of a credible subgroup effect based on representativeness of the study population. Moderate certainty evidence found the prevalence of chronic pain among studies enrolling military veterans from the general population was 30% (95% CI: 23% to 37%) compared to 51% (95% CI: 38% to 64%) among military veterans sampled from populations with high rates of conditions associated with chronic pain (*p* = .005).

**Discussion/Conclusions**: We found moderate certainty evidence that three in every ten military veterans from the general population live with chronic noncancer pain. These findings underscore the importance of ensuring access to evidence-based care for chronic pain for veterans.

**The GRADE Working Group and CINeMA approaches provided inconsistent certainty of evidence ratings for a network meta-analysis of opioids for chronic noncancer pain**

Atefeh Noori^a^, Behnam Sadeghirad^a^, Lehana Thabane^a^, Mohit Bhandari^b^, Gordon H. Guyatt^a^ and Jason W. Busse^c^

^a^McMaster University, Department of Research Methods, Evidence and Impact, ^b^McMaster University, Department of Surgery, ^c^McMaster University, Department of Anesthesia

**Introduction/Aim**: Assessment of the certainty of evidence (CoE) from network meta-analysis is critical to convey the strength of inferences from clinical decision making. Both the GRADE Working Group (GWG) and the CINeMA framework have been designed to assess the CoE of treatment effects informed by network meta-anlysis; however, the concordance of results is uncertain.

**Methodology**: We assessed the CoE for treatment effects of individual opioids on pain relief and physical functioning from a network meta-analysis for chronic noncancer pain using the GWG approach and the CINeMA framework. Both approaches evaluate the CoE as high, moderate, low, or very low. We quantified the number of discrepant CoE ratings between approaches, and the magnitude of the difference (i.e., 1-level, 2-levels, or 3-levels).

**Results**: Across 105 comparisons among individual opioids for pain relief, the GWG and CINeMA approaches provided different CoE ratings in 34% of cases (36 of 105). Across 66 comparisons for physical functioning, there was discordance in 17% of cases (11 of 66). All discrepancies were separated by 1-level. The CINeMA framework typically provided lower CoE ratings compared to the GWG approach, predominantly because of differences in the assessment of transitivity and heterogeneity.

**Discussion/Conclusions**: Our findings suggest there are differences between the CoE ratings provided by the GWG and CINeMA approaches when applied to network meta-analyses. Further research is needed to replicate or refute our findings in other network meta-analyses and assess the implications for clinical decision-making.

**Patient perspectives on recovery from myalgic encephalomyelitis/chronic fatigue syndrome**

Zara Hasan^a^, Cassandra Kuyvenhoven^a^, Mehreen Chowdhury^a^, Lana Amoudi^a^, Dena Zeraatkar^b^, Jason W. Busse^b^, Marina Sadik^a^ and Meredith Vanstone^a^

^a^McMaster University, Department of Family Medicine, ^b^McMaster University, Department of Anesthesia

**Introduction/Aim**: Myalgic encephalomyelitis (ME), also called chronic fatigue syndrome (CFS), is characterized by persistent fatigue, post exertional malaise, and cognitive dysfunction. It is a complex, long‐term, and debilitating illness without widely effective treatments. This study describes the treatment choices and experiences of ME/CFS patients who have experienced variable levels of recovery.

**Methodology**: Interpretive description study consisting of semistructured qualitative interviews with 33 people who met the U.S. Centers for Disease Control (2015) diagnostic criteria for ME/CFS and report recovery or symptom improvement.

**Results**: Twenty‐six participants endorsed partial recovery, and seven reported full recovery from ME/CFS. Participants reported expending significant time and energy to identify, implement, and adapt therapeutic interventions, often without the guidance of a medical practitioner. They formulated individualized treatment plans reflecting their understanding of their illness and personal resources. Most fully recovered participants attributed their success to mind‐body approaches.

**Discussion/Conclusion**: Patients with ME/CFS describe independently constructing and managing treatment plans, due to a lack of health system support. Stigmatized and dismissive responses from clinicians precipitated disengagement from the medical system and prompted use of other forms of treatment

**Best practice for transition from pediatric complex pain care: A scoping review – Implications for pain clinics**

Monica Ouellet^a^, Amanjot Kaur^a^ and Krista Baerg^a^

^a^University of Saskatchewan

**Introduction/Aim**: Many adolescent and young adults (AYA) experiencing chronic pain often continue to have pain in adulthood and thus must make the transition from pediatric care to adult care. However, only few studies have described the specific transition protocol from pediatric to adult care that AYA with chronic pain must make. To improve understanding of transitional care needs (quality of care), this scoping review will determine known barriers and factors associated with successful transition from pediatric multidisciplinary chronic pain care to adult care.

**Methodology**: This scoping review used previously published Arksey and O’Malley framework and followed the PRISMA guidelines. A literature search was carried on Ovid MEDLINE and 12 studies (11 research articles and 1 dissertation) published from 2015 to 2022 were included in the review, with only one theoretical transition framework that may be useful in the Canadian healthcare setting.

**Results**: In this scoping review, interpersonal, organizational, and systemic barriers and enablers for successful transition were identified. The most frequently identified barriers to successful transition include lack of self-efficacy, trust, communication, coordination of care between pediatric and adult care. A need of early transition, an individualized approach, fluid and dynamic transition process, and age-appropriate resources were identified as primary patient care needs during transition. Four frameworks supporting transitional care for AYA with chronic health conditions were identified.

**Discussion/Conclusions**: Limited evidence suggests that there is lack of standardized transition protocol for clinical use. There is an urgent need for an evidence-based transition tools for clinical use for AYA with chronic pain.

**Physicians’ and patients’ perceived risk of chronic pain medications**

Gwenaelle De Clifford-Faugère^a^, Anaïs Lacasse^a^, Aline Boulanger^b^, Marimée Godbout-Parent^a^, Hermine Lore Nguena Nguefack^a^ and Nancy Julien^a^

^a^Université du Québec en Abitibi-Témiscamingue (UQAT), ^b^Centre hospitalier de l’Université de Montréal (CHUM); Université de Montréal

**Introduction/Aim**: The risk associated with pain medications influence both the physician and the patient. It can influence a physician’s choice of molecule and dosage and can also influence a patient’s adherence to the medication and quality of life. Given that involving patients in medication decisions is integral to delivering high-quality care, our aim was to assess whether physicians and patients were aligned in their perceptions of the risks associated with chronic pain (CP) medications.

**Methodology**: A total of 83 physicians and 141 CP patients from the province of Quebec (Canada) responded to a survey measuring the perceived risk with 0–10 numerical scales (0 = no risk, 10 = very high risk). Physicians scored the 36 medication subclasses of the Medication Quantification Scale 4.0 (MQS 4.0) (through online surveys), and CP patients only scored the medication subclasses they were taking (through telephone interviews).

**Results**: Differences of at least 2 points out of 10 were found for 12 medication subclasses, with physicians consistently perceiving a higher level of risk compared to patients. For instance, mean perceived risk scores (physicians vs patients) were 7.54 vs 2.50 for benzodiazepines, 7.20 vs 3.62 for long-acting opioids, 5.87 vs 2.10 for medical/therapeutic cannabis (all *p* < .001).

**Discussion/Conclusions**: CP patients perceived medication use as less risky than physicians. Divergent risk perceptions can lead to challenges in communication and patient understanding, potentially affecting deprescription and self-management strategies. The MQS 4.0 may serve as a valuable tool for physicians to address such discrepancies, thereby promoting shared decision making.

**Unpacking polypharmacy patterns: Chronic pain patients in Quebec**

Gwenaelle De Clifford-Faugère^a^, Hermine Lore Nguena Nguefack^a^, Nancy Ménard^a^, Sylvie Beaudoin^a^, M. Gabrielle Pagè^b^, Line Guénette^c^, Catherine Hudon^d^, Oumar Mallé Samb^a^ and Anaïs Lacasse^a^

^a^Université du Québec en Abitibi-Témiscamingue (UQAT), ^b^Centre de recherche du Center Hospitalier de l’Université de Montréal, ^c^Centre de recherche du Center hospitalier universitaire de Québec – Université Laval, ^d^Centre de recherche du Center hospitalier universitaire de Sherbrooke (CRCHUS)

**Introduction/Aim**: Excessive polypharmacy, the concurrent use of ≥10 medications, is prevalent among persons with chronic pain, but it remains unclear how it may vary within groups of individuals or over time. This study aimed to describe and identify determinants of trajectories of excessive polypharmacy.

**Methodology**: This retrospective cohort study was conducted using the TorSaDE Cohort, which links the Canadian Community Health Survey (2007–2016 cycles) and Quebec health administrative databases (longitudinal claims). Among 9,156 adults living with chronic pain (self-reported) and covered by the public prescription drug insurance, the presence of excessive polypharmacy (yes/no) was assessed monthly for one year postsurvey completion, totaling 12 time points. Group-based trajectory modeling (GBTM) was applied to identify subgroups (trajectories) of individuals with similar patterns over time. Multivariable logistic regression was used to identify the determinants of trajectory membership.

**Results**: A four-trajectory model was obtained: 1) “Sometimes in excessive polypharmacy” (8.6%); 2) “No excessive polypharmacy” (74.8%); 3) “Often in excessive polypharmacy” (6.1%); 4) “Always in excessive polypharmacy” (10.5%). Determinants of the “always in excessive polypharmacy” trajectory membership were: being older, born in Canada, having a lower household income, a high comorbidity index score, moderate/severe pain intensity, some/most activities prevented by pain, arthritis or back pain, fair/bad perceived general health, and a regular physician. They also included using opioids or benzodiazepines, no/occasional alcohol consumption, rare physical activity, and a high number of prescribers and visits to a family physician (*p* < .05).

**Discussion/Conclusions**: The risk of experiencing excessive polypharmacy among individuals living with chronic pain varies depending on various characteristics.

**Surgical timing shows no influence on postoperative pain in a mouse model of incisional wound**

Eleri McEachern^a^, Maria Zilic^a^, Nader Ghasemlou^b^ and Jeffrey Mogil^a^

^a^McGill University, ^b^Queen’s University

**Introduction/Aim**: Postoperative pain affects over 80% of surgical patients and is typically managed with NSAIDs, opioids, and acetaminophen, which have associated side effects. To complement these options, there is growing interest in chronotherapy, which focuses on timing interventions. Circadian rhythms governed by core molecular clocks significantly influence physiological functions, including the immune response and pain mechanisms. Although the impact of time of day on pain in various conditions has been studied, whether time of injury affects subsequent pain behaviors has not been well characterized. We aimed to assess postoperative pain outcomes in a mouse model of hind paw incision in a circadian-dependent manner.

**Methodology**: Plantar incisions were made at four times of day (ZT2, ZT8, ZT14, ZT20; wherein ZT0 signifies lights-on and ZT12 lights-off). Pain behaviors were evaluated using mechanical sensitivity tests, radiant heat paw-withdrawal assays, and the Mouse Grimace Scale (MGS) at ZT8. Assessments occurred at 1, 3-, 5-, 7-, and 10-days postincision, followed by weekly evaluations until pain resolution.

**Results**: Our results revealed no significant differences in peak allodynia or pain resolution when taking into account time of injury or sex.

**Discussion/Conclusions**: This suggests that surgery time during resting or active phase does not substantially affect postoperative pain. Better understanding of whether surgical recovery would benefit from chronotherapy could lead to more biologically informed postoperative care and improved experimental control in research settings.

**Opioids for chronic noncancer pain: an updated systematic review and meta-analysis**

Li Wang^a^, Alireza Malektojari^b^, Sara Ghazizadeh^b^, Yaping Chang^c^, Jhalok Ronjan Talukdar^d^, Lucas Lorimer^e^, Zaineb Hamza^f^, Rachel J. Couban^d^, Mark Jeddi^c^, Atefeh Noori^c^, Wenjun Jiang^f^, Dorisa Meng^g^ and Jason W. Busse^h^

^a^McMaster University, Michael G. DeGroote Institute for Pain Research and Care/ Department of Anesthesia, ^b^Hormozgan University of Medical Sciences, ^c^McMaster University, Department of Health Research Methods, Evidence and Impact (HEI), ^d^McMaster University, Michael G. DeGroote Institute for Pain Research and Care, ^e^Western University, Schulich School of Medicine and Dentistry, ^f^McMaster University, Faculty of Health Sciences, ^g^McMaster University, Michael G. DeGroote School of Medicine, ^h^McMaster University, Departments of Anesthesia and Health Research Methods, Evidence and Impact (HEI); Michael G. DeGroote Institute for Pain Research and Care; Michael G. DeGroote Center for Medicinal Cannabis Research

**Introduction/Aim**: Our previous meta-analysis in 2018 showed that opioids used for chronic noncancer pain (CNCP) resulted in statistically significant but small improvements in pain and physical functioning, and increased risk of vomiting compared with placebo. A number of new randomized clinical trials (RCT) have been published since 2018. We aimed to update our previous systematic review and meta-analysis to provide the most recent evidence.

**Methodology**: We updated our search for CENTRAL, CINAHL, EMBASE, MEDLINE, AMED, PsycINFO and trial registries to identify RCTs of opioids for chronic noncancer pain vs any nonopioid control. Using standardized forms, paired reviewers independently screened literature, assessed risk of bias, and extracted study data. We converted each continuous outcome into the most common instrument. We performed random-effects meta-analysis and explored possible sources of heterogeneity with a priori subgroup analyses and meta-regression. We used GRADE to evaluate the certainty of evidence for each outcome

**Results**: Our updated literature search identified 14,271 citations, of which 20 new RCTs provided eligible. Totally, we included 116 RCTs and 30,703 patients with CNCP. There were 29 trials for neuropathic pain, 39 trials for nociceptive pain, 41 trials for nociplastic pain and 7 trials of mixed types of pain. 

We are in the process of data cleaning and analyses and will complete and be prepared to present our findings at the Canadian Pain Society 2024 Annual Scientific Meeting.

**Discussion/Conclusions**: Our systematic review will provide the most recent evidence for the update of 2017 Canadian opioid guideline for chronic noncancer pain management.

**Impact of cannabis use during adolescence and young adulthood on academic achievements: a systematic review and meta-analysis of observational studies**

Olsen Chan^a^, Ahad Daudi^b^, David Ji^b^, Mathias Wang^b^, Jeremy P. Steen^c^, Parsia Parnian^b^, Crystal Li^b^, Wei Zhang^d^, Luciane Cruz Lopes^e^, James MacKillop^f^, Jason W. Busse^g^ and Li Wang^h^

^a^University of Toronto, Temerty Faculty of Medicine, ^b^McMaster University, Faculty of Health Sciences, ^c^University of Toronto, Institute of Health Policy, Management and Evaluation, ^d^McMaster University, Library, ^e^University of Sorocaba, Pharmaceutical Science Graduate Course, ^f^McMaster University, Department of psychiatry and Behavioral Neurosciences, Michael G. DeGroote Center for Medicinal Cannabis Research, ^g^McMaster University, Departments of Anesthesia and Health Research, Methods, Evidence and Impact (HEI); Michael G. DeGroote Institute for Pain Research and Care; Michael G. DeGroote Center for Medicinal Cannabis Research, ^h^McMaster University, Michael G. DeGroote Institute for Pain Research and Care/ Department of Anesthesia

**Introduction/Aim**: Cannabis use during adolescence and young adulthood is has been associated with lower academic achievement, but with varying findings and the magnitude of associations remains uncertain. We aimed to conduct a systematic review and meta-analysis to assess the association of cannabis use on academic achievement.

**Methodology**: We searched CINAHL, EMBASE, MEDLINE, PsycINFO, PubMed, Scopus, and Web of Science up to April 23, 2023, for observational studies examining the associations of cannabis use with academic outcomes. We used random-effects model for meta-analyses of three or more studies, and fixed effects models for meta-analyses of two studies.

**Results**: Forty-nine studies proved eligible, including 42 primary studies (*n* = 343,692) and seven studies with nine overlapping cohorts (*n* = 23,944). Moderate-certainty evidence showed cannabis use during adolescence and young adulthood was associated with lower school grades (Odds ratio [OR] for ≥grade B 0.64, 95%CI [0.56–0.74]), high school graduation (OR 0.50 [0.33–0.76]), university enrollment (OR 0.67 [0.58–0.78]), postsecondary degree attainment (OR 0.67 [0.61–0.74]); and, conversely, higher school dropout rate (OR 1.63 [1.63–1.94]), school absenteeism (OR 2.35 [1.69–3.27]), and unemployment (OR 1.76 [1.23–2.53]). The absolute risk changes ranged from 5% to 19%. Low certainty evidence showed no significant association with grade retention (OR 1.41, 95%CI [0.97 to 2.03]). Subgroup analyses with moderate credibility showed worse academic outcomes for frequent users than infrequent users.

**Discussion/Conclusions**: Cannabis use during adolescence and young adulthood, particularly more frequent use, is associated with worse academic outcomes in terms of school grades, high school graduation, university enrollment, educational attainment, school dropout, absenteeism, and unemployment.

**“You’re there, but your brain is not”: A qualitative descriptive study on the impacts of brain fog in Veterans with chronic pain**

Ronessa Dass^a^, Zeal Kadakia^a^, Nancy Paris Rosen^a^, Susan Clarke-Tizzard^a^, Jocelyn Harris^a^, Diana Velikonja^a^ and Tara Packham^a^

^a^McMaster University

**Introduction/Aim**: Brain fog is a common manifestation of chronic pain described to cause challenges with memory, attention, and executive function, thus resulting in reduced participation in daily activities. Canadian Veterans are twice as likely as their civilian counterparts to experience chronic pain and cognitive disruptions, linked to a higher likelihood of physical and psychological conditions. The impact of chronic pain related brain fog has not been qualitatively explored in Veterans. There is a need to understand the specific impacts for Veterans to help health care professionals adapt care according to the unique characteristics of Veterans.

**Methodology**: The present study used a qualitative descriptive approach, guided by a constructivist theoretical lens. Focus groups of 1–4 participants were used for data generation and were analyzed using a qualitative descriptive and gender matrix analysis.

**Results**: A total of 25 Veterans (F = 6, M = 19) across Canada participated in this study. Three categories were identified: 1) brain fog experiences, 2) linear and cyclic relationships of the triggers, impacts, and management strategies of brain fog, and 3) barriers and solutions for brain fog management. Gender differences on the cognitive and emotional impacts and management strategies were identified in the matrix analysis.

**Discussion/Conclusions**: Brain fog was described as a variable experience which could create cyclic cognitive and emotional impacts, limiting meaningful engagement and goal achievement in Veterans with chronic pain. To reduce the burden of this experience and restore quality of life, Veterans emphasized the need for awareness, measurement tools, and modifications to existing interventions.

**Investigating the relationship between phantom limb pain, telescoping, and psychosocial experience: A case study**

Andrea Aternali^a^, Heather Lumsden-Ruegg^a^, Sander L. Hitzig^b^, Amanda L. Mayo^c^ and Joel Katz^a^

^a^York University, Department of Psychology, Toronto, ON, Canada, ^b^Sunnybrook Research Institute, St. John’s Rehab Research Program, Toronto, ON, Canada, ^c^Physical Medicine and Rehabilitation, Sunnybrook Health Sciences Center, Toronto, ON, Canada

**Introduction/Aim**: Approximately 80% of individuals who undergo limb amputation experience phantom limb pain (PLP), yet effective treatments are lacking. A better understanding of phantom limb “telescoping,” the experience of one’s phantom hand or foot gradually approaching the residual limb, may assist in developing effective interventions for PLP. This single case report explores the relationships between PLP, telescoping, and psychosocial experience.

**Methodology**: The participant was a 36-year-old male who underwent a right transfemoral amputation due to a traumatic injury approximately 4 years ago. He responded to online questionnaires evaluating demographic and health-related information, pain experience, psychological variables, and telescoping. He also completed a semi-structured interview that was analyzed for patterns in PLP and telescoping experiences.

**Results**: The participant rated his average PLP as 10 on an 11-point Numeric Rating Scale (NRS) shortly after amputation. Approximately 12 months later, the participant noticed a shortening of his phantom limb, with a concurrent decrease in PLP. At present, his average NRS pain intensity is a 5/10. His score of 2 on the ID Pain Questionnaire suggests that he does not experience symptoms of neuropathic pain. His mean score of 4.29/10 on the Brief Pain Inventory (Short Form) indicates moderate levels of pain interference. His score of 4 on the four-item Patient Health Questionnaire indicates he has mild symptoms of anxiety and depression.

**Discussion/Conclusions**: In the present case, telescoping of the phantom limb was accompanied by a reduction in PLP intensity. This study provides insight into factors that may maintain PLP, generating targets for further investigation.

**Nurses’ evaluations of the feasibility and clinical utility of the use of the nociception level (NOL™) index in critically ill adults**

Shiva Shahiri T. ^a^, Patrick Lavoie^b^, Marc O. Martel^c^, Philippe Richebé^d^ and Céline Gélinas^a^

^a^Ingram School of Nursing, McGill University, Montréal, Canada, ^b^Faculty of Nursing, Université de Montréal, Montreal, Canada, ^c^Faculty of Dental Medicine and Department of Anesthesiology, McGill University, Montreal, Quebec, Canada, ^d^Department of Anesthesiology and Pain Medicine, University of Montreal, Montreal, Canada

**Introduction/Aim**: The Nociception Level (NOL™) index monitors nociception and pain using multiple physiological parameters through a noninvasive finger probe and is currently undergoing validation for ICU pain assessment. Evaluating its feasibility and clinical utility from nurses’ perspectives during this process is crucial for its potential adoption into clinical practice, which is the aim of this study.

**Methodology**: This descriptive study involved ICU nurses who received a brief NOL training (i.e., 5-minute video and handout). Trained nurses who used the NOL during validation at the ICU bedside completed a self-administered questionnaire on NOL’s feasibility and clinical utility.

**Results**: Out of the 22 trained nurses, 8 (36%) used the NOL on enrolled patients and completed the questionnaire. Their average age was 34 years, and 63% were female. *Feasibility*: All nurses found the NOL’s application, installation, and calibration easy and quick, with clear usage instructions. They all agreed that the training improved their understanding of NOL. However, 50% of them found it too brief. They recommended prioritizing hands-on practice and multiple exposures to the NOL’s use at the bedside over video recordings and handouts. *Clinical Utility*: 50% of the nurses agreed that the NOL is useful and could influence their practice if found valid. Others (50%) disagreed, suggesting it should remain an assistive, not a diagnostic tool, due to numerous limitations concerning its use in the ICU, including patient’s cardiac instability and movements.

**Discussion/Conclusions**: Nurses perceive the NOL as a potential alternative for ICU pain assessment in mechanically ventilated and sedated ICU patients unable to self-report.

**Astrocyte-Neuron Dynamics in Chronic Neuropathic Pain: Sex-Dependent Mechanisms in the Anterior Cingulate Cortex**

Ana Leticia Simal^a^, Jaime Tuling^a^ and Giannina Descalzi^a^

^a^University of Guelph

**Introduction:/Aim**: Chronic pain impacts 25% of Canadians aged 15 and above, especially marginalized groups, with women making up 67% of those affected. Preclinical research has predominantly prioritized male rodent models, leaving a knowledge gap regarding female chronic pain mechanisms. Neuroimaging studies have identified neuroplastic changes within the anterior cingulate cortex (ACC) being critical for chronic pain development. Responding to neuronal activity, the astrocyte-neuronal lactate shuttling (ANLS) can rapidly provide lactate to neurons to meet large metabolic demands caused by neuroplasticity. The ANLS is necessary for pain-mediated, fear-learning induced neuroplasticity in the rat hippocampus, however its role in chronic pain-induced neuroplasticity remains unknown. This study explored the ANLS in chronic neuropathic pain development in the ACC of female and male mice, seeking new molecular insights for improved treatment efficacy.

**Methodology**: Adult (8 wks) female and male C57BL/6 mice underwent the spared nerve injury (SNI) model to induce neuropathic pain. The expression of genes involved in the ANLS in the ACC were assessed through RT-qPCR at 5, 14, 30, and, 60 days postsurgery. Mechanical allodynia was confirmed using the Von Frey Test prior to sample collection.

**Results**: Long-term SNI resulted in elevated ANLS-related gene expression in male mice, with no significant difference observed in female mice. Female and male mice displayed comparable levels of mechanical allodynia across different time points.

**Discussion/Conclusions**: Neuropathic pain engages ANLS in the mouse ACC in a sexually dimorphic manner. These observed sex differences emphasize the importance of including both sexes when investigating novel molecular targets for chronic pain treatment, contributing our understanding of pain chronification.

**The health administrative data puzzle: Unraveling insurance disparities**

Marimée Godbout-Parent^a^, Nancy Julien^a^, Hermine Lore Nguena Nguefack^a^ and Anaïs Lacasse^a^

^a^Université du Québec en Abitibi-Témiscamingue

**Introduction/Aim**: Approximately 46% of the Quebec population is covered by the public prescription drug insurance plan, which includes persons aged ≥65, without access to a private plan through an employer, or recipients of financial assistance.

To better appraise the external validity of studies conducted with public prescription claims databases, this study compared the characteristics of persons living with chronic pain who are publicly insured versus privately insured.

**Methodology**: The COPE Cohort was used, formed by linking a web-based survey with Quebec prescription claims. Insurance status was determined based on coverage in the year following survey completion. Characteristics were compared using chi-square and *t*-tests.

**Results**: Out of 895 participants, 48.3% were publicly insured. When examining the most clinically important (≥10%) and statistically significant (*p*< .05) differences, publicly insured participants were more likely to be unemployed (80.3% vs. 51.7%), exhibit pain catastrophizing (66.4% vs. 55.0%), or report using ≥10 medications (34.9% vs. 23.5%). They were less likely to have completed postsecondary education (72.9% vs. 84.5%) and to be never smokers (37.3% vs. 49%). Publicly insured participants were older (54.2 ± 14.1 vs. 46.6 ± 11.1) and had lower general health (34.4 ± 12.49 vs. 37.6 ± 13.1) and physical functioning SF-12 scores (30.0 ± 9.5 vs. 33.3 ± 11.1). Although statistically significant, differences in terms of sex, gender identity, and pain characteristics (intensity, interference, duration, frequency) did not appear to be clinically important.

**Discussion/Conclusions**: Sociodemographic and clinical differences exist based on medication insurance status. The external validity of studies conducted with public prescription claims should be assessed on a case-by-case basis, considering these results.

**Test stimulus intensity-dependent conditioned pain modulation in patients with fibromyalgia**

Laila A. Chaudhry^a^, Isabel Aboud^a^, Mathilde Ferland^a^, Simon Carrier^a^, Marc O. Martel^a^ and Jeffrey S. Mogil^a^

^a^McGill University

**Introduction:/Aim**: Conditioned pain modulation (CPM) is a well-known psychophysical phenomenon considered to be a biomarker of endogenous descending pain mechanisms. Previous mouse and human data from our lab (Tansley et al., 2019; Chaudhry et al., unpublished data) demonstrates that test stimulus intensity affects CPM’s direction, with higher-intensity stimuli leading to hypoalgesia (i.e., CPM) and lower-intensity stimuli leading to hyperalgesia (i.e., “anti-CPM”). Deficits in CPM, or “weaker” CPM, suggest low capacity to inhibit pain, predicting the development of chronic pain (Edwards et al., 2003; Lewis et al., 2012). Because of this, our aim was to see how this “anti-CPM” phenomenon presents itself in chronic pain patients with fibromyalgia, with a hypothesis that the CPM and anti-CPM effects would either be reversed or replaced entirely with hyperalgesia.

**Methodology**: Patient participants (*N* = 20 of 60) underwent an individual heat pain threshold (HPTh) assessment, followed by one CPM trial, at −1, +1, or +3°C below/above their HPTh. Each CPM trial consisted of two baseline sub/suprathreshold heat pain stimulations (the test stimulus), a 30-s cold pressor test (CPT; 4°C), and a final heat pain stimulation at the same temperature, with pain ratings (0–100 VAS) provided throughout.

**Results**: A two-way repeated measures ANOVA was conducted to assess changes in pain ratings from pre- to post-CPT within and across temperature groups. Preliminary results indicate a trend toward the reversal of both CPM and anti-CPM effects in pain patients, further emphasizing the importance of test stimulus intensity in CPM experiments, and the role of CPM as a diagnostic assessment tool for chronic pain.

**Opioid craving in patients with chronic pain on long-term opioid therapy: Insights from an ecological momentary assessment (EMA) study**

Alice Bruneau^a^, Amanda Sirois^b^, Sarah Petkau^c^, Alexandra Gavrilescu^c^, Yami-Louise Djoudi^c^, Jordi Perez^d,e^ and Marc O. Martel^b, d, e^

^a^Faculty of Medicine and Health Sciences, McGill University, ^b^Faculty of Dental Medicine and Oral Health Sciences, McGill University, ^c^Department of Psychology, McGill University, ^d^Alan Edwards Pain Management Unit, McGill University Health Center, ^e^Department of Anesthesia, McGill University

**Introduction/Aim**: Despite the potential benefits of opioids, their use can lead to problems such as opioid misuse and opioid use disorder in a subset of patients. Evidence indicates that opioid craving (i.e., the desires and/or urges to use opioids) can increase patients’ propensity to misuse opioids, but the factors contributing to opioid craving in patients with pain are not clearly understood. Patients’ physical dependence to opioids, manifested by transient opioid withdrawal symptoms, can contribute to opioid craving. Increases in pain intensity and psychological distress can also potentially lead to opioid craving. Research is needed to better understand how pain symptoms, psychological states, and opioid-related problems interrelate in everyday life of patients with chronic pain.

The main objective was to examine the intra-day associations between pain intensity, negative affect (NA), opioid withdrawal symptoms, and opioid craving in patients with chronic pain.

**Methodology**: The study received ethical approval and consent was obtained from all study participants (*n* = 60). Ecological momentary assessment (EMA) procedures were used for 10 consecutive days to assess patients’ levels of pain, NA, and opioid-related variables such as opioid withdrawal symptoms and opioid craving.

**Results**: Intra-day elevations in pain intensity, NA, and withdrawal symptoms were associated with higher levels of opioid craving (all *p* < .05). The association between opioid withdrawal symptoms and craving was mediated by concurrent elevations in pain and negative affect (*p* < .05).

**Discussion/Conclusions**: Our study provides new insights into our understanding of factors that may contribute to opioid craving and opioid misuse among patients with chronic pain.

**The relationship between pupillary light reflex and clinical measures of nociplastic pain in chronic WAD? An observational study**

Colin Wasylynuk^a^ and Ashley Smith^b^

^a^Department of Physical Therapy, University of British Columbia, Vancouver, Canada, ^b^Department of Clinical Neurosciences, Cumming School of Medicine, University of Calgary, Canada

**Introduction/Aim**: People with chronic whiplash-associated disorders (WAD) may demonstrate features of nociplastic pain, such as increased light sensitivity as measured via the Pupillary Light Reflex (PLR).  It is unknown if light sensitivity is a feature of chronic WAD and if it is associated with other clinical measures of nociplastic pain.  The aims of this study were to determine if: (1) there was a significant difference in PLR measures between people with chronic WAD and healthy controls (HCs), and (2) PLR measures were associated with clinical measures of nociplastic pain in chronic WAD. 

**Methodology**: 26 people with chronic WAD were age/sex-matched to HCs. PLR measures were collected using a validated iPhone app.  Clinical measures of nociplastic pain collected included: pain intensity, Central Sensitization Inventory – CSI, Pressure Pain Thresholds – PPTs, Temporal Summation – TS and Conditioned Pain Modulation – CPM.  Independent *t*-tests evaluated group differences and Pearson’s correlation analyses measured the relationship between PLR and clinical measures of nociplastic pain. 

**Results**: Participants with chronic WAD demonstrated elevated CSI, reduced local and distal PPTs, and facilitated TS when compared to HCs.  There were no differences in CPM.  Chronic WAD demonstrated significantly reduced maximum constriction amplitude and quicker time to return to 75% of resting pupil diameter.  PLR measures were not correlated to measures of nociplastic pain in chronic WAD. 

**Discussion/Conclusions**: People with chronic WAD demonstrated sympathetic and parasympathetic autonomic nervous system dysfunction. There was no relationship between PLR and clinical measures of nociplastic pain, suggesting that altered nociceptive input is not driving this aberrant autonomic response in chronic WAD. 

**Transition needs among Veterans living with chronic pain: A systematic review**

Mansi Patel^a^, Jane Jomy^b^, Rachel Couban^a^, Hélène Le Scelleur^c^ and Jason Busse^a^

^a^McMaster University, ^b^University of Toronto, ^c^University of Ottawa

**Introduction/Aim**: An estimated 40% of Canadian veterans live with chronic pain, which is likely associated with greater needs during the transition from military to civilian life. This review explores challenges and transition needs among military personnel living with chronic pain as they return to civilian life.

**Methodology**: We searched MEDLINE, EMBASE, CINAHL, Scopus, and Web of Science from inception to July 2022, for qualitative, observational, and mixed-method studies exploring transition needs among military veterans released with chronic pain. Reviewers, independently and in duplicate, screened studies and extracted data from included studies using a standardized data collection form. Content analysis was used to identify patterns in challenges and unmet needs of veterans transitioning to civilian life. We summarized our findings in a descriptive manner.

**Results**: Of 10,532 unique citations, we identified 43 studies that reported transition challenges and needs of military personnel; however, none were specific to individuals released with chronic pain. Most studies (41 of 43; 95%) focused on military personnel in general, with one study enrolling individuals with traumatic brain injury and another including homeless veterans. We identified military-to-civilian challenges in seven areas: (1) identity, (2) interpersonal interactions/relationships, (3) employment, (4) education, (5) finances, (6) self-care and mental health, and (7) accessing services and care.

**Discussion/Conclusions**: Military personnel who transition to civilian life report several important challenges; however, the generalizability to individuals released with chronic pain is uncertain. Further research is needed to better understand the transition experiences of veterans with chronic pain to best address their needs and enhance their well-being.

**Perceptions on opioid prescribing after total joint arthroplasty among orthopedic surgeons practising in Canada, Japan, and the Netherlands: A qualitative description study**

Mansi Patel^a^, Parsia Parnian^a^, Kim Madden^a^, Sheila Sprague^a^, Anita Acai^a^, Ydo Kleinlugtenbelt^b^, Natsumi Saka^c^, Ellie Landman^b^, Harsha Shanthanna^a^, Vickas Khanna^a^ and Jason Busse^a^

^a^McMaster University, ^b^Deventer Ziekenhuis, ^c^Teikyo University School of Medicine

**Introduction/Aim**: Opioid analgesics are commonly prescribed after total knee and hip arthroplasty to manage pain. Rates of opioid prescribing after arthroplasty differ by country suggesting differences in policies or surgeons’ perceptions.

**Methodology**: We adopted a qualitative description design to compare the perceptions of Canadian, Dutch, and Japanese orthopedic surgeons on postoperative opioid prescribing and to explore facilitators and barriers to opioid reduction. We used a combination of convenience and purposive sampling, and a snowball recruitment technique to facilitate 27 semistructured interviews online or via a phone call. We concurrently collected and analyzed data using conventional (inductive) content analysis.

**Results**: Surgeons in Canada prescribed opioids to 99–100% of their arthroplasty patients postoperatively, compared to 60–100% of patients in the Netherlands, and 0–100% in Japan. Japanese surgeons believed that opioids were often unnecessary for managing postoperative pain. Although all Dutch surgeons utilized an institutional standard pain management protocol, Canadian and Japanese surgeons exhibited high variability in opioid prescribing, even within the same institution. Orthopedic surgeons in each country identified challenges and facilitators to reduced postoperative opioid use in six key areas: (1) opioid prescribing practices, (2) patient factors, (3) collaborative care, (4) opioid prescribing policies/guidelines, (5) surgeon education, and (6) personal perceptions/beliefs.

**Discussion/Conclusions**: Canadian and Dutch surgeons are more likely to prescribe opioids after arthroplasty compared to Japanese surgeons. Canadian surgeons report high variability in the duration and dose of opioids prescribed compared to surgeons from other countries. Our findings suggest opportunities for standardization of opioid prescribing after joint replacement surgery.

**Mind over matter: Exploring behavioral responses in pain management during COVID-19**

Brenda Lau^a,b^, Emmanuel Abreu^a^, Neha Singh^a^, Tameus Venkataraman^a^, Aidan McParland^a,c^, Kai Xin Zhang^a^, Justin Brass^a,d^ and Dorina Sluka^a^

^a^CHANGEpain Clinic, ^b^University of British Columbia, ^c^University of Toronto, ^d^Yorkville University

**Introduction:/Aim**: During the COVID-19 pandemic, the CHANGEpain Clinic pivoted to digital platforms, epitomizing the rapid evolution in chronic pain management. This study reviewed the broad implications of this shift, concentrating on the clinic’s transition and its effect on healthcare delivery, with particular attention to increased reach, patient access, and the behavioral changes essential for successful digital healthcare adoption.

**Methodology**: Employing a mixed method approach, our study consolidated data from 2019 to 2021 to assess the CHANGEpain Clinic’s transition to virtual care. This included quantitative analysis of care encounters and qualitative evaluation of patient and provider satisfaction. With support from the Chronic Pain Center of Excellence for Canadian Veterans, the research aimed to comprehend the intricacies of adopting digital health platforms for pain management.

**Results**: The clinic experienced a 300% surge in virtual appointments, notably in Group Medical Visits and consultations. This transition not only continued care during the pandemic but extended the clinic’s reach, with patients accessing services from an average distance of 64.3 km, including remote regions. Providers reported high satisfaction and a preference for the permanence of digital health services, recognizing the benefits of increased accessibility and efficiency.

**Discussion/Conclusions**: The CHANGEpain Clinic’s shift to online services illuminated digital health’s potential in democratizing healthcare access, particularly in pain management. Although the transition presented challenges, such as technological barriers and the need for digital literacy, it also offered a roadmap for future healthcare delivery. This study advocates for continued digital health integration, emphasizing investment in infrastructure, training, and patient engagement to optimize the digital healthcare landscape.

**Can’t wait any longer: How uncertainty affects our pain choices across time**

Éliane Rochelet^a^, Taryn Berman^a^ and Mathieu Roy^a^

^a^McGill University

**Introduction/Aim**: How does temporal uncertainty affect our decision-making processes? Past literature has demonstrated that when timing of a reward is unpredictable, people prefer to expedite its experience to reduce unpleasant feelings of uncertainty associated with the waiting period (Bixter and Luhmann, 2015; McGuire and Kable, 2013). However, little is known about how uncertainty impacts the way we make inter-temporal decisions regarding pain. In the present study, we investigated this very topic.

**Methodology**: We recruited 80 participants who partook in an inter-temporal choice task where they had to decide between receiving a certain level of electrical stimulation (i.e., 60, 70, 80, or 90% of pain tolerance) immediately or at a delay (i.e., 15s, 30s, 1-hr, or 1-month). Temporal uncertainty was manipulated by having half of the participants see a count-up when selecting the 15- or 30-seconds option (i.e., count group), and half see a fixation cross (i.e., no-count group), thus making the timing of the shock unpredictable.

**Results**: We found that all participants generally preferred immediate over delayed pain, particularly those in the no-count group. This pattern was found for all delays and was present even when immediate pain was greater than delayed pain, indicating that people are willing to accept more pain than needed to avoid uncertainty.

**Discussion/Conclusions**: Our findings imply that some people, when faced with real-life situations of temporal uncertainty regarding pain (e.g., waiting in the emergency room for an injury), may decide to escape the situation to avoid unpleasant feelings associated with uncertainty, preventing essential care and thus allowing the pain to worsen.

**More than a romantic connection: Partner emotion co-regulation in the context of chronic pain**

Anastasia Mekhael^a^, Michelle Gagnon^b^ and Meghan McMurtry^a,c^

^a^University of Guelph, ^b^University of Saskatchewan, ^c^McMaster Children’s Hospital

**Introduction/Aim**: Effective caring for individuals with chronic pain (ICP) requires caregivers to accurately perceive the individuals’ pain, and *then* manage their own emotional response.^1^ This process requires emotion regulation (ER), defined as the strategies used to influence how one’s emotions are felt and expressed.^2^ Research with romantic dyads illustrates that emotions of partners affect individuals with chronic illness.^3,4^ To date, we do not have a comprehensive understanding of the construct of ER in partners of individuals with Chronic Pain (CP). We will answer: 1) What do we know about ER in spouses of ICPs, including conceptualization and measurement? 2) What relationship does spousal ER have with CP-related outcomes?

**Methodology**: A scoping review is being conducted according JBI^9^ methodology. *Inclusion*: English, peer-reviewed papers using any design type with participants in romantic relationships and CP. *Exclusion*: Cancer-related CP; conference presentations. OVID Medline, PsycINFO, Web of Science and CINAHL were searched from database inception supplemented by hand searches.^5^

**Results**: 3811 records were identified of which 2176 have undergone screening (57%) with 126 moving to full text review; of these, 6 met inclusion criteria (*N* = 252 dyads). Results suggest: 1) the construct of ER is not well operationalized in the CP literature and 2) bidirectional relationships exist between emotional experiences of caregivers and their partners with CP (e.g., synchrony between spousal and ICP’s reports of the ICP’s anger regulation and its effects). This poster will present results from the full sample.

**Discussion/Conclusions**: We will highlight gaps in the present research to guide future research and potential intervention targets for CP.

**Triage decision-making in interdisciplinary pediatric chronic pain programs**

Megan Greenough^a^, Krystina Lewis^b^, Tracy Bucknall^c^, Lindsay Jibb^d^, Jennifer Leese^e^, Christine Lamontagne^a^ and Janet Squires^b^

^a^CHEO, ^b^Unviersity of Ottawa, ^c^Deakin University, ^d^SickKids, ^e^University of Ottawa

**Introduction/Aim**: Interdisciplinary pediatric chronic pain programs are ideal treatment settings for youth with chronic pain who are complex from a biopsychosocial perspective. There is currently no evidence-based clinical decision support to guide nurses triaging patients to such programs, which increases the risk for haphazard triage decisions. The aim of this study was to explore and describe the decision-making practices of and contextual influences on nurses triaging patients to interdisciplinary pediatric chronic pain programs.

**Methodology**: A qualitative exploratory descriptive design was undertaken that involved individual, semi-structured interviews with 12 nurses across 11 different interdisciplinary pediatric chronic pain programs. Interviews were transcribed verbatim and analyzed using concurrent content analysis, guided by the Cognitive Continuum Theory and the Theoretical Domains Framework.

**Results**: Analysis generated three prominent themes: 1) Nurse-led triage determinants, 2) Process of triage decision-making, and 3) External influences on triage decision-making.

**Discussion/Conclusions**: Triage decision making in the setting of interdisciplinary pediatric chronic pain programs is complex and often led by nurses. There is a desire amongst nurses to adopt an evidence-based Clinical Decision Support triage tool (CDS), which may streamline the referral and triage process and foster a system whereby patients in highest need for interdisciplinary care are best prioritized. The following recommendations could be considered in developing clinical decision triage support: 1) Solidify expectations, 2) Enhance diagnostic clarity, 3) Incorporate Patient Reported Outcome Measures, 4) Accept that triage is an evolving decision and prepare for change, and 5) Optimize communication and collaboration.

**A systematic review of the biopsychosocial dimensions impacted by chronic pain in children and adolescents: Identifying reliable and valid pediatric multidimensional chronic pain assessment tools**

Megan Greenough^a^, Lindsay Jibb^b^, Krystina Lewis^c^, Tracey Bucknall^d^, Christine Lamontagne^a^, Melissa Demery Varin^a^, Ashley Sokalski^a^ and Janet Squires^c^

^a^CHEO, ^b^SickKids, ^c^University of Ottawa, ^d^Deakin University

**Introduction/Aim**: Pediatric chronic pain is a complex experience that is often challenging to describe and measure. Multidimensional tools that evaluate the biopsychosocial impact of chronic pain in pediatrics can help clinicians to prioritize and tailor interdisciplinary pain care; yet the psychometric value and clinical utility of such tools has not yet been systematically studied in the literature. The purpose of this review was to identify multidimensional biopsychosocial tools used in pediatric chronic pain, synthesize their reliability and validity evidence, and draw on this evidence to describe the relationships between chronic pain and biopsychosocial domains.

**Methodology**: The search involved two phases to 1) identify eligible tools and 2) conduct a measured forward citation search of tool development articles. Tool eligibility was guided by the *Multidimensional Biobehavioral Model of Pediatric Pain* and study eligibility was focused on primary chronic pain diagnoses unrelated to disease. Data extraction was focused on reliability and validity evidence of eligible tools, guided by the *Standards for Educational and Psychological Testing*.

**Results**: Results yielded 6 tools that included 64 eligible studies, highlighting 84 significant relationships between pain and functional interference across 11 biopsychosocial variables. All tools were shown to have good internal consistency and evidence of validity, primarily through relationships to other variables.

**Discussion/Conclusions**: Of the 6 tools, the most brief and easy to use were the most under studied. Further psychometric research is warranted for these tools to investigate their clinical utility and psychometric properties in guiding and prioritizing pain care for children and adolescents.

**Attaining Expert Consensus on Diagnostic Expectations of Primary Chronic Pain Diagnoses for Patients Referred to Interdisciplinary Pediatric Chronic Pain Programs: A Delphi Study with Pediatric Chronic Pain Physicians and Advanced Practice Nurses**

Megan Greenough^a^, Tracey Bucknall^b^, Lindsay Jibb^c^, Krystina Lewis^d^, Christine Lamontagne^a^ and Janet Squires^d^

^a^CHEO, ^b^Deakin University, ^c^SickKids, ^d^University of Ottawa

**Introduction/Aim**: Pediatric primary chronic pain disorders come with diagnostic uncertainty, which may obscure diagnostic expectations for referring providers and the decision to accept or re-direct patients into interdisciplinary pediatric chronic pain programs based on diagnostic completeness. We aimed to attain expert consensus on diagnostic expectations for patients who are referred to interdisciplinary pediatric chronic pain programs with 6 common primary chronic pain diagnoses.

**Methodology**: We conducted a modified Delphi study with pediatric chronic pain physicians, nurse practitioners and clinical nurse specialists to determine degree of importance on significant clinical indicators and diagnostic items relevant to each of the 6 primary chronic pain diagnoses. Items were identified through point of care databases and complimentary literature and were rated by participants on a 5-point Likert scale. Our consensus threshold was set at 70%.

**Results**: Amongst 22 experts across 14 interdisciplinary programs in round one and 16 experts across 12 interdisciplinary programs in round two, consensus was reached on 84% of diagnostic items, where the highest degree of agreement was with Complex Regional Pain Syndrome (CRPS), Type 1 (100%) and the lowest with chronic pelvic pain (67%).

**Discussion/Conclusions**: This study demonstrated a general agreement amongst pediatric chronic pain experts regarding diagnostic expectations of patients referred to interdisciplinary chronic pain programs with primary chronic pain diagnoses. Study findings may help to clarify referral expectations and the decision to accept or redirect patients into such programs based on diagnostic completeness while reducing the occurrence of unnecessary diagnostic tests and subsequent delays in accessing specialized care.

**Utilization patterns and impact of the chronic pain self-management program in Eastern Ontario**

Emily Hum^a^, Sathya Karunananthan^a^, Areej Adil^b^, Isabella Moroz^c^, Rachel Davidson^d^ and Clare Liddy^e^

^a^School of Interdisciplinary Health Sciences, University of Ottawa, ^b^Chatham Kent Health Alliance, ^c^Research Associate, Bruyère Research Institute, ^d^Regional Program Coordinator, Chronic Disease Self-Management, Living Healthy Champlain, ^e^Chair, Department of Family Medicine, University of Ottawa

**Introduction/Aim**: Healthcare providers often struggle to treat patients with chronic pain. One potential solution is to facilitate patient access to programs that develop skills and confidence in managing their own care. In this study, we aimed to describe patterns of utilization of a Chronic Pain Self-Management Program (CPSMP) in Eastern Ontario and evaluate the impact of the program on the Patient Activation Measure (PAM), a measure of participants’ involvement in their care, their health behaviors, and their knowledge of the condition.

**Methodology**: Using data routinely collected through the CPSMP between December 2017 and May 2023, we conducted descriptive analysis of the number of participants each year, their gender and age distributions. We conducted longitudinal analyses of change in PAM score between participants’ enrollment in the program (baseline) and the end of the program (follow-up).

**Results**: 1,023 individuals participated in the CPSMP during the study period. The number of participants peaked in 2018 and remained stable thereafter. There was a higher proportion of female (69%, *n* = 709) compared to male participants and a higher proportion of 50–59-year-olds, compared to other age groups. Among the 151 participants (15% of the total sample) who completed a PAM survey at baseline and follow-up, 69% (104/151) experienced a clinically meaningful increase of 4 points on the PAM scale.

**Discussion/Conclusions**: Participation in the CPSMP resulted in a clinically meaningful increase in patient activation among patients with chronic pain. As only 15% of CPSMP participants completed the PAM survey at baseline and follow-up, replication in a larger sample is warranted.

**Treatment preferences among Canadian military Veterans with chronic low back pain: a mixed methods cross-sectional survey**

Peter C.` Emary ^a^, Carla Ciraco^b^, Jenna DiDonato^b^, Branden Deschambault^c^, Andrew Garas^d^, Sheila Sprague^e^ and Jason W. Busse^c^

^a^McMaster University, Micheal G. DeGroote Institute for Pain Research and Care, ^b^Private Practice, ^c^McMaster University, Department of Anesthesia, ^d^McMaster University, Faculty of Health Sciences, ^e^McMaster University, Department of Surgery

**Introduction/Aim**: Chronic low back pain is prevalent among military Veterans. The purpose of our study was to examine Canadian military Veterans’ use and preferences toward specific health care disciplines and treatment approaches for the management of chronic low back pain.

**Methodology**: From February to May 2023, we e-mailed a 33-item survey, in English and French, to a list of Canadian Armed Forces (CAF) Veterans living with chronic low back pain that asked about demographic variables, military service, chronic low back pain-related characteristics, and experiences and attitudes toward health care providers and therapeutic approaches for chronic low back pain.

**Results**: Overall, 290 of 1,632 CAF Veterans returned a completed survey (18% response rate). Almost all (98%) respondents reported living with chronic low back pain for more than five years, and 91% indicated first experiencing low back pain during military service. Respondents most preferred registered massage therapy, physiotherapy, family medicine, and chiropractic care for managing their chronic low back pain. Most respondents endorsed that registered massage therapy (70%), physiotherapy (60%), chiropractic care (51%), and occupational therapy (50%) should be available on-base for active-duty military personnel.

**Discussion/Conclusions** Our findings suggest there may be an opportunity to better align on-base health care with treatment preferences of actively serving CAF members. Treatment preferences of military personnel should inform future research and policies to optimize management of low back pain among CAF members.

**Treating myofascial low back pain with dry needling: a systematic review**

Fabiola Dach^a^ and Karen Ferreira^b^

^a^University of Sao Paulo, Medical School of Ribeirao Preto, Ribeirao Preto, Sao Paulo, Brazil.,^b^Department of Neurology, Suroit Hospital, Salaberry-de-Valleyfield, Quebec, Canada

**Introduction/Aim**: Myofascial Pain Syndrome (MPS) is a common source of pain in primary care or pain clinics. There are many different ways to manage and treat MPS, such as physical exercise, trigger points massage, and dry needling. The objective of this overview is to highlight and discuss the evidence-based treatment of myofascial pain by dry needling in patients with low back pain.

**Methodology**: A systematic review was performed based on meta-analysis (MA) and randomized controlled trials (RCTs) related to dry needling treatment for myofascial pain in patients with lumbar pain, published from 2000 to 2023.

**Results**: A total of 509 records were identified at first. Seventy were published before 2000, so they were excluded. From the remaining 439 studies, ninety-two were RCTs or MA, of which 86 additional studies were excluded for the following reasons: not related to dry needling treatment (*n* = 79), not published in English (*n* = 4), duplicated (*n* = 1), project protocol (*n*= 1), and not related to myofascial pain (*n* = 1). So, this review was based on 4 RCTs and two MA. These studies compared dry needling efficacy to other treatments, such as acupuncture, sham dry needling, laser therapy, physical therapy, local anesthetic injection, ischemic compression and neuroscience education. Despite outcomes and follow-up period varied between them, they showed that dry needling can decrease postintervention pain intensity and pain disability.

**Discussion/Conclusions**: Dry needling is an effective procedure for the treatment of myofascial pain in patients with acute and chronic low back pain. Further high-quality studies are needed to clarify the long-term outcomes.

**Causalgia: a review of nerve resection, amputation, immunotherapy and amputated limb CRPS II pathology**

Peter Watson^a^, Rajiv Midha^b^ and Denise W. Ng^c^

^a^University of Toronto, ^b^University of Calgary, Department of Clinical Neurosciences, Section of Neurosurgery, ^c^University of Calgary, Department of Pathology and Laboratory Medicine

**Introduction/Aim**: Causalgia and complex regional pain syndrome (CRPS) type II with nerve injury can be difficult to treat. Surgical peripheral nerve denervation for causalgia has been largely abandoned by pain clinicians because of a perception that this may aggravate a central component (anesthesia dolorosa).

**Methodology**: We selectively searched Pubmed, Cochrane, MEDLINE, EMBASE, CINAHL Plus, Scopus from 1947 for articles, books, and book chapters for evidence of surgical treatments (nerve resection, amputation) and treatment related to autoimmunity and immune deficiency with CRPS.

**Results**: Reviews were found for the treatment of causalgia or CRPS type II (*n* = 6), causalgia relieved by nerve resection (*n* = 6), and causalgia and CRPS II treated by amputation (*n*= 8). Twelve reports were found of autoimmunity with CRPS, one paper of these on associated immune deficiency and autoimmunity, and two were chosen for discussion regarding treatment with immunoglobulin and one by plasma exchange. We document a report of a detailed, and unique pathological examination of a CRPS type II affected amputated limb and related successful treatment with immunoglobulin.

**Discussion/Conclusions**: Nerve resection, with grafting, and relocation may relieve uncomplicated causalgia and CRPS type II in some patients in the long term. However, an unrecognized and treatable immunological condition may underly some CRPS II cases and can lead to the ultimate failure of surgical treatments.

**Providing a pain app to support patients undergoing spine surgery and their surgeons: uptake, utilization, and participant experiences**

Kayla Denness^a^, Sara Mallinson^a, b^, Carla Vetland^a^, Gurpreet Brar^a^, Asma Khan^a^, Nivez Rasic^a, b^ and Sanjay Beesoon^a, c^

^a^Alberta Health Services, ^b^University of Calgary, ^c^University of Alberta

**Introduction/Aim**: As part of a Health Canada Substance Use and Addictions Program (SUAP) grant, Alberta Health Services (AHS) aims to improve supports for people at risk of chronic postsurgical pain. Eleven participating spine surgeons and their patients undergoing surgery in Alberta were offered free access to a digital health application: Manage My Pain (MMP). Patients were invited to self-monitor their pain pre- and postsurgery using the app. Surgeons were able to view MMP data entered by their patients in a clinical portal.

**Methodology**:

The project uses a mixed-methods approach.

1. App uptake and utilization are evaluated using background app data and the clinical portal.
2. User feedback on the experience of using the app and whether it supports better pain communication and management is assessed using an online survey and qualitative interviews with a sample of patient and surgeon participants.
3. Online surveys containing standardized health and quality of life questionnaires are collected at baseline, 30 days, and 90 days to explore health impacts.

**Results**: 85 spine surgery patients enrolled in the study between February 2023 and September 2023 and 61 (71%) downloaded MMP. Application, survey, and interview data collection is ongoing until Dec 22^nd^. The poster will present descriptive analysis of quantitative data and key themes from qualitative interviews.

**Discussion/Conclusions**: The utility of a self-completed app to record pain experiences over time in spine surgery patients will be discussed along with recommendations for AHS and other health systems testing innovative digital strategies to improve patient-focused transitional pain management.

**Excitatory N-methyl-D-aspartate receptor subunits are asymmetrically distributed across the mediolateral axis of the rodent but not human dorsal horn**

Katherine Griffiths^a,b^, Jennifer Armstrong^a^, Clare Murray Lawson^a,b^, Laurence David^a^, Santa Temi^a^, Jessica Parnell^a,b^, Annemarie Dedek^a,b^, Eve C. Tsai^b^ and Michael E. Hildebrand^a,b^

^a^Carleton University, Department of Neuroscience, Ottawa, Ontario, Canada, ^b^The Ottawa Hospital, Ottawa Hospital Research Institute, University of Ottawa, Ottawa, Ontario, Canada

**Introduction/Aim**: Despite chronic pain being highly prevalent, there are limited treatments that are both safe and effective. Glutamatergic N-methyl-D-aspartate receptors (NMDARs) are found across the central nervous system, with GluN2 subunit variants in the spinal cord consisting of GluN2A, GluN2B, and GluN2D. The superficial dorsal horn (SDH) is a critical site for nociceptive signaling, where NMDARs are essential regulators of plasticity and excitability. The relative expression and localization of GluN2 NMDAR subunits within the SDH across sex and species remains unknown.

**Methodology**: To investigate GluN2 subunit localization across the dorsal horn, we used immunohistochemistry techniques on rat and human lumbar and thoracic spinal cord tissue for both sexes, in conjunction with antigen retrieval and tyramide signal amplification. Additionally, we used costaining with neuronal (NeuN) and presynaptic afferent (CGRP) markers to test whether the GluN2 expression is specific to differences of NMDAR distribution and not cellular density or synaptic innervation.

**Results**: GluN2 subunits are preferentially expressed in the SDH of rat and human spinal cord tissue in both sexes. However, in both thoracic and lumbar spinal segments, there is a heightened localization of GluN2 subunits to the lateral SDH of rats compared to a uniform GluN2 distribution across the mediolateral SDH axis for humans. This GluN2 asymmetry in rodents is driven by differences in NMDAR distribution as both NeuN and CGRP are uniformly distributed across the dorsal horn mediolateral axis.

**Discussion/Conclusions**: These findings highlight potential differential NMDAR signaling and plasticity across the mediolateral SDH somatotopic map, which differs across species.

**Combined intraosseous and intra-articular allogeneic stem cell therapy for knee osteoarthritis: A case report**

Alex Gleave^a^, Adeel Khan^b^ and Shahnawaz Towheed^a^

^a^Michael G. Degroote Faculty of Medicine, McMaster University, ^b^Temerty Faculty of Medicine,University of Toronto

**Introduction/Aim**: Knee osteoarthritis (OA) poses a major health challenge, causing pain and functional limitations. Traditional treatment approaches often fail to provide adequate relief. This case report investigates injecting allogeneic culture-expanded adipose-derived stem cells (ADSCs) into both the intra-articular and intraosseous regions of the knee of a middle-aged female patient with medial compartment knee OA. The objective is to evaluate the effectiveness of this combined approach for pain relief and functional improvement.

**Methodology**: 25 million allogeneic culture-expanded fat stem cells were injected into the interosseous part of the medial femur and medial tibia, with 15 M cells intra-articular and 10 M intraosseous.

**Results**: Before treatment: the left knee had 90 degrees of flexion (compared to 120 degrees in the right), and the patient reported 10/10 on the Visual Analogue Scale (VAS) during activity. Two months posttreatment, the left knee reached 110° flexion, VAS pain score decreased to 5/10, and self-rated recovery was at 50%. At the 3-month follow-up, the left knee’s motion remained at 110°, VAS decreased to 2/10, and recovery was at 90%. By the fourth month, the left knee’s flexion improved to 115°, VAS pain score was 1/10, and recovery was at 95%. At the 8-month follow-up, the left knee achieved 120° range of motion, VAS pain score at 0/10, and recovery reached 99%.

**Discussion/Conclusions**: The combination of intra-osseous and intra-articular allogeneic stem cell injections conferred substantial pain relief and improved functionality in this middle-aged female patient with knee OA. This combined approach may revolutionize OA management, but more research is needed.

**Empowered Management for Pelvic Pain: The experiences of women with persistent pelvic pain participating in an online self-directed self-management program while they wait for interprofessional care**

Rachael Bosma^a^, Emeralda Burke^a^, Celeste Corkery^a^, Wendy Carter^a^, Nida Mustafa^a^, Sarah Sheffe^a^,Carleen Ginter^a^, Rosemary Wilson^b^, Nucelio Lemos^a^ and Tania Di Renna^a^

^a^Women’s College Hospital, ^b^Queen’s University

**Introduction/Aim**: The lack of interprofessional chronic pelvic pain (CPP) management programs that incorporate the complex interplay of biopsychosocial factors impacting patients with CPP into treatment often results in extended wait times and negative health outcomes. Offering patients CPP self-management education may enhance pain coping skills and provide additional support while they wait for care. The aim of this study is to understand the experience of women with CPP participating in an online, self-management education program (“Pelvic Pain Empowered Management” program) while awaiting interprofessional care.

**Methodology**: A descriptive qualitative approach was used to explore the experiences of women participating in an online educational program designed for cis women with CPP. Eleven women participated in semistructured interviews, which were transcribed verbatim using NVivo software and analyzed inductively using established methods for thematic analysis.

**Results**: Women shared their perceptions with the Empowered Management Program and discussed how it impacted their experience while waiting for interprofessional CPP care. Patients revealed that access to a self-directed management program tailored for pelvic pain enhanced their understanding of their disease in ways that were relevant to them, resonated with their own experiences, and helped them develop skills and strategies to better manage their pain.

**Discussion/Conclusions**: Our study identified the need to support women living with CPP by utilizing online tailored educational program to enhance care while they await a medical consultation. Provision of self-directed online patient education can support patients to better understand their disease, set expectations around care, and develop self-management strategies and skills.

**“Pain invalidation shakes your entire construction as a human being”: A qualitative analysis of causes and consequences of epistemic injustices by healthcare providers in chronic pain testimony**

Catherine Côté^a^ and Pascale Devette^a^

^a^Université de Montréal

**Introduction/Aim**: An extensive body of literature discusses the frequent invalidation of patient’s chronic pain by healthcare professionals. Among these studies, only a few refer to epistemic injustices as a conceptual framework to understand invalidation. Previous studies have addressed testimonial and hermeneutic injustices but have overlooked epistemic violence, namely testimonial silencing, and smothering, as well as epistemic oppression and exploitation. Another important gap is that most research on epistemic injustices in this context is theoretical or based on general literature on chronic illnesses and not on patients’ testimonies. Previous literature only addressed epistemic injustices in an interpersonal context (i.e., relationship between patient and provider), and neglected how epistemic injustices and violence can be intricated in systems such as the healthcare system or oppression systems.

**Methodology**: Narrative interviews (*N*= 17) were conducted with chronic pain patients in Quebec to better understand encounters in which they experienced validation and/or invalidation. Data was analyzed using Braun et al. (2019) reflexive thematic analysis to identify accounts of invalidation/validation, possible causes to epistemic injustices and the consequences they produce.

**Results**: Results showed accounts of testimonial and hermeneutic injustices, as well as epistemic violence, oppression, and exploitation. Analyses revealed that invalidation can be produced by healthcare system’s pressure on practitioners to see more patients, more quickly. This can lead to poorer quality listening and to inadequate care.

**Discussion/Conclusions**: As testimonial injustices are rooted in oppression systems such as sexism, transphobia, and fatphobia, implications for social health inequities are discussed, including difficulties in accessing adequate healthcare. Gendered differences are also discussed.

**Reliability and validity of a single-item measure of recovery expectations in rehabilitation research and practice**

Junie Carrière^a^, Djamal Berbiche^a^, Laury Montemurro^a^ and Michael Sullivan^a^

^a^Université de Sherbrooke

**Introduction/Aim**: The objective of this study was to investigate the reliability and validity of a single-item measure of recovery expectations in the context of work disability.

**Methodology**: Two independent samples of individuals with musculoskeletal pain conditions were used to evaluate the reliability and validity of a single-item measure of recovery expectations (“How likely is it that within the next month you will have resumed some form of employment?”). Sample 1 consisted of 109 individuals with work-related musculoskeletal injuries and Sample 2 consisted of 152 individuals with whiplash injury. Participants completed measures of demographics, recovery expectations and pain-related psychological variables (depressive symptoms, catastrophizing, fear of movement and perceptions of injustice). At 1-year follow-up, participants reported their work status. Analyses examined the test-retest reliability, parallel-form reliability, construct validity and predictive validity of a single-item measure of recovery expectations on work status at 1-year follow-up.

**Results**: Findings reveal good test-retest reliability, parallel-form reliability, construct validity and predictive validity. Together, these results support the use of single-item measures of recovery expectations to predict work disability following musculoskeletal pain conditions.

**Discussion/Conclusions**: This study represents a step toward practice-based evidence by providing a brief, low burden, low-cost measure of recovery expectations that can be seamlessly integrated into clinical workflow and research protocols.

**A systematic review of perceived injustice and pain-related outcomes in youth with pain conditions**

Naz Yagmur Alpdogan^a^, Megan M. Miller^b^, Larbi Bennalal^c^, Marie-Pier Royer^a^, Marie-France Coutu^a^ and Junie S. Carrière^a^

^a^University of Sherbrooke, ^b^Cincinnati Children’s Hospital Medical Center, ^c^McGill University

**Introduction/Aim**: A growing body of literature shows that perceived injustice is a significant determinant of pain-related outcomes and prolonged trajectories of recovery in adults. We conducted a systematic review of the literature assessing the relationship between perceived injustice and pain-related outcomes in youth with pain conditions.

**Methodology**: A search of published studies in English in PubMed, PsychInfo and Cochrane Database of Systematic Reviews from database inception through December 2022 was performed. The search protocol identified research articles that included measures of perceived injustice and pain-related outcomes in youth with pain conditions. A total of 56 articles were screened and 8 met inclusion criteria.

**Results**: Data for a total of 1240 youth with pain conditions were extracted. The mean age for patients across all studies was of 14.12 (*SD* = 2.25), and 68.2% of participants were female. There is strong evidence that perceived injustice is associated with pain intensity, disability, mental health outcomes, and emotional, social and school functioning.

**Discussion/Conclusions**: There were several overlapping samples from the United States of America, which limits the evidence included in this review. However, the results underscore the need for screening and treatments targeting injustice appraisals in pediatric populations with pain conditions. Clinical implications and directions for future research are discussed.

